# Exploratory N‐Protecting Group Manipulation for the Total Synthesis of Zwitterionic *Shigella sonnei* Oligosaccharides

**DOI:** 10.1002/chem.202003480

**Published:** 2021-03-01

**Authors:** Debashis Dhara, Laurence A. Mulard

**Affiliations:** ^1^ Unité de Chimie des Biomolécules UMR 3523 CNRS, Institut Pasteur 28 rue du Dr Roux 75015 Paris France

**Keywords:** AAT, altrose, glycosylation, protecting groups, zwitterionic polysaccharides

## Abstract

*Shigella sonnei* surface polysaccharides are well‐established protective antigens against this major cause of diarrhoeal disease. They also qualify as unique zwitterionic polysaccharides (ZPSs) featuring a disaccharide repeating unit made of two 1,2‐*trans* linked rare aminodeoxy sugars, a 2‐acetamido‐2‐deoxy‐l‐altruronic acid (l‐Alt*p*NAcA) and a 2‐acetamido‐4‐amino‐2,4,6‐trideoxy‐d‐galactopyranose (AAT). Herein, the stereoselective synthesis of *S. sonnei* oligosaccharides comprising two, three and four repeating units is reported for the first time. Several sets of up to seven protecting groups were explored, shedding light on the singular conformational behavior of protected altrosamine and altruronic residues. A disaccharide building block equipped with three distinct *N*‐protecting groups and featuring the uronate moiety already in place was designed to accomplish the iterative high yielding glycosylation at the axial 4‐OH of the altruronate component and achieve the challenging full deprotection step. Key to the successful route was the use of a diacetyl strategy whereby the *N*‐acetamido group of the l‐Alt*p*NAcA is masked in the form of an imide.

## Graphical abstract

**Fine‐tuned** ***N*** **‐protecting** group selection and manipulation to control the ^1^C_4_⇌^2^S_O_⇌^4^C_1_ conformational equilibrium of l‐Alt*p*NAcA within a disaccharide building block and to accomplish high yielding iterative glycosylation and full deprotection. The synthesis of di‐, tetra‐, hexa‐ and octasaccharide segments from the *S sonnei* zwitterionic polysaccharides was achieved.

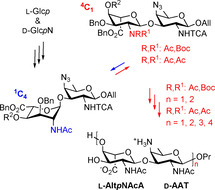

## Introduction

Diarrheal diseases are a major public health burden worldwide and the second leading‐cause of death in children under 5 years of age. Two recent keynote studies—the GEMS^[1]^ and the MAL‐ED^[2]^—have identified *Shigella* as one of the top agents causing moderate‐to‐severe diarrhea in this population. Still, the global burden of shigellosis is thought to be underestimated and the emergence of multidrug‐resistant strains goes against antibiotic treatment as being the sole answer to *Shigella* burden.^[3]^ Fighting shigellosis by means of vaccines was recommended decades ago by WHO and vaccination is still viewed as a valuable preventive intervention. However, no broadly licensed *Shigella* vaccine is available despite a diversity of vaccine candidates tested in clinical trials.^[4]^ *Shigella sonnei*, as a single serotype, causes an estimated 25 % of all shigellosis episodes. It is the second most common *Shigella* species in low and middle income countries and the predominant species in high income and transitional countries.^[5]^ High incidence in traveler's diarrhea and increasing antibiotic resistance also contribute to concern for this Gram negative enteroinvasive bacterium.^[6]^ Evidence point to *S. sonnei* surface lipopolysaccharide as being the major protective antigen against reinfection,^[7]^ and among the many strategies under investigation toward a *S. sonnei* vaccine, polysaccharide conjugates have emerged as a promising route.^[8]^ Otherwise, exploring the feasibility of using synthetic carbohydrate haptens as surrogates of the *S. sonnei* natural polysaccharide antigens is envisioned as a promising alternative. It is noteworthy that this strategy was originally investigated in our group to tackle a *Shigella flexneri* 2a infection.^[9]^ A vaccine candidate featuring a 15‐mer oligosaccharide hapten—a trimer analogue of the biological repeating unit of the *S. flexneri* 2a O‐antigen (O‐Ag)—linked to a protein carrier via single point attachment was proposed,^[10]^ and more recently demonstrated to be safe and strongly immunogenic in adult volunteers.^[11]^ Now paying attention to the second most prevalent *Shigella* serotype, we report herein our exploratory work and successful achievements on the chemical synthesis of oligomers of the repeating unit from the *S. sonnei* O‐Ag, a unique zwitterionic polysaccharide (ZPS). *S. sonnei* is to our knowledge, the only *Shigella* surrounded by a capsular polysaccharide (CPS). As recently disclosed, the two *S. sonnei* surface polysaccharides display the same zwitterionic repeating unit.^[12]^ Therefore, our effort aims in the long run at a *S. sonnei* vaccine candidate capable at inducing both an anti‐LPS and an anti‐CPS antibody‐mediated protective response.

**Figure 1:**
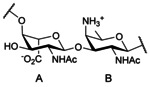
Biological repeat of the O‐Ag and CPS from *S. sonnei*: [4)‐α‐l‐Alt*p*NAcA‐(1→3)‐β‐d‐Fuc*p*NAc4N‐(→].[^12^, ^13^]

As for other ZPSs, the zwitterionic character of the surface polysaccharides from *S. sonnei* stems from adjacent monosaccharide units harboring alternating charges within the repeating unit. But to our knowledge, the *S. sonnei* ZPSs are the sole as of to date featuring a disaccharide repeating unit. The latter is made of two uncommon amino sugars, a 2‐acetamido‐2‐deoxy‐l‐altruronic acid (l‐Alt*p*NAcA, A) and a 2‐acetamido‐4‐amino‐2,4,6‐trideoxy‐d‐galactopyranose (d‐Fuc*p*NAc4N, AAT, B) 1,2‐*trans*‐linked to one another (Figure 1).[^12^, ^13^] Despite being an unusual component within the whole glycome, AAT has been identified in several other bacterial ZPSs, most often as an α‐linked residue as exemplified in the CPS from *Streptococcus pneumoniae* serotype 1 (Sp1)^[14]^ and *Bacteroides fragilis* (PS A1).^[15]^ It was less frequently found in its β‐form as present in *S. sonnei* and *Plesiomonas shigelloides* O17, which expresses an O‐Ag identical to that of *S. sonnei*,^[16]^ and more recently identified in the LPS from *Providencia alcalifaciens* O22, another cause of diarrheal disease,^[17]^ and in the lipoteichoic acid of *Streptococcus oralis* Uo5.^[18]^ Owing to their characteristic immunomodulatory properties,^[19]^ ZPSs—especially Sp1 and PS A1—and synthetic fragments thereof have attracted a lot of interest in recent years whether aiming at developing vaccine haptens^[20]^ or for use as vaccine carrier.^[21]^ In that context, AAT has qualified as an attractive synthetic target.^[22]^ In contrast, l‐Alt*p*NAcA is barely encountered, being to our knowledge originally reported as a key component of the *S. sonnei* and *P. shigelloides* O17 ZPSs. Besides its exceptional zwitterionic nature, a distinctive feature of the *S. sonnei* O‐Ag is the occurrence of three amino groups, two of which present as acetamide, within a disaccharide repeat. Following pioneering work from V. Pozsgay and collaborators two decades ago,^[23]^ we have reported the chemical synthesis of the biological repeat (AB) of the *S. sonnei* O‐Ag, its frame‐shifted analogue (BA), and trisaccharides ABA and BAB in the form of propyl glycosides.^[24]^ More recently, we also described an alternative synthesis of a ready‐for‐chemical‐oligomerization AB disaccharide building block disclosed in our original report.^[25]^ However, achieving the [AB]_*n*_ oligosaccharides (n>2) was more demanding.

**Scheme 1:**
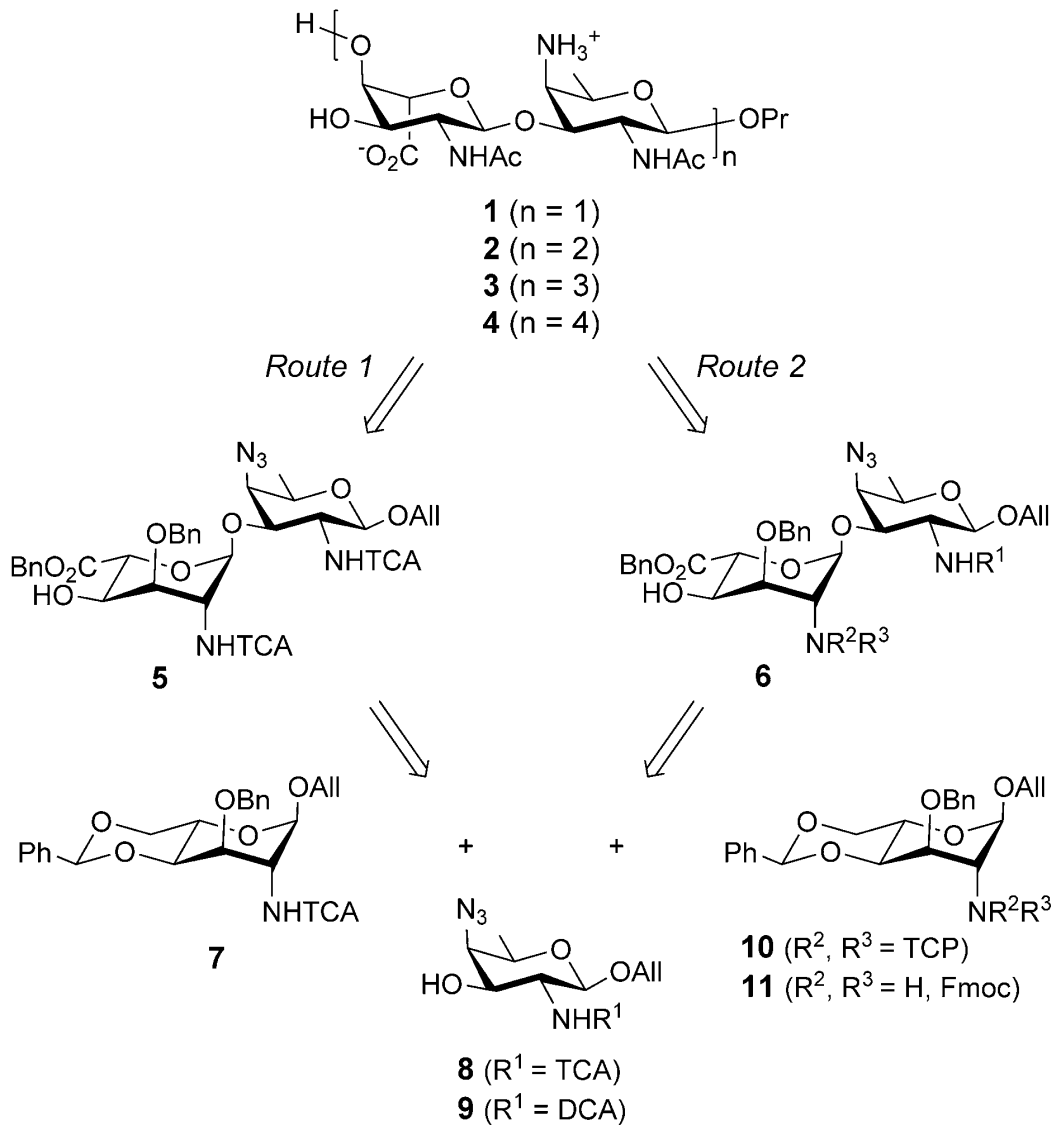
Routes to di‐, tetra‐, hexa‐ and octasaccharides **1**, **2**, **3** and **4**, respectively, and potential monosaccharide precursors thereof. All: allyl, Fmoc: 9‐fluorenylmethoxycarbonyl, DCA: dichloroacetyl, TCA: trichloroacetyl, TCP: tetrachlorophtaloyl, R^n^: protecting groups.

As a key feature of our original strategy^[24]^ (Scheme 1, Route 1), both the 2_A_‐ and 2_B_‐acetamides were masked as trichloroacetamides. In support to this selection are the excellent neighboring group participating properties of the *N*‐trichloroacetyl (TCA) moiety and the large diversity of conditions enabling its exchange into the natural acetamide.^[26]^ The successful application of TCA in the synthesis of large oligosaccharides encompassing multiple 2‐acetamido sugars was exemplified on several occasions,^[27]^ including in the field of *Shigella*.^[28]^ The recently reported automated synthesis of a β‐(1→6)‐linked glucosamine dodecamer, whereby the corresponding 12 acetamido moieties were revealed post assembly by exposure to a large excess of tin hydride, illustrates the most striking achievement.^[29]^ As an attempt to avoid the use of this toxic reagent at a late stage of the synthesis of a potential vaccine component, we have favored the *N*‐TCA palladium‐mediated reductive hydrodechlorination concomitant to benzyl hydrogenolysis, plus azide and allyl reduction.^[24]^ However, conditions used with success in the synthesis of oligosaccharides representative of the *S. flexneri* type 3a O‐Ag,^[28b]^ resulted in complex mixtures in the *S. sonnei* context. For instance, whereas conversion of the *N*‐TCA group into the corresponding *N*‐chloroacetyl moiety often proceeds smoothly, further conversion of the latter into the expected acetamide may be sluggish.^[30]^ The risk of incomplete conversion increases with the number of *N*‐TCA groups, especially for those located at internal residues as the chain grows larger.^[31]^ These concerns led us to reinvestigate the protecting group pattern of a suitable ready‐for‐oligomerization AB building block to achieve the synthesis of [AB]_*n*_ oligomers.

As an attempt to overcome issues met at the latest stage in the synthesis of large *S. sonnei* oligosaccharides by means of the key intermediate **5** (Scheme 1, Route 1),^[24]^ we reasoned that the final palladium‐catalyzed step should be avoided or at least that the number of acetamido groups to unmask by palladium‐mediated hydrodechlorination should be reduced. We report herein on a detailed exploration of diverse routes to AB building blocks featuring up to seven orthogonal *O*‐ and *N*‐protecting groups and fulfilling this criterion. We discuss the relevance of various protecting group combinations in the context of conformation, glycosylation, oligomerization and final deprotection. Finally, we describe an effective convergent route to generate *S. sonnei* oligosaccharides encompassing several repeating units.

Similarly to the strategy implemented to achieve the first synthetic glycan‐based *Shigella* vaccine candidate that has reached clinical evaluation,^[11]^ the availability of the di‐, tetra‐, hexa‐ and octasaccharides (**1**–**4**)—herein synthesized as their propyl glycoside—and the feasibility of larger well‐defined fragments of the *S. sonnei* ZPSs pave the way to detailed molecular investigation. Epitope mapping,[^31^, ^32^] supported by thorough conformational and structural analysis,[^31^, ^32b^, ^32c^, ^33^] will contribute to unravel the molecular attributes governing antigenic mimicry of the bacterial polysaccharide antigens by short synthetic oligosaccharides as a step forward to a structure‐guided design of a *S. sonnei* synthetic glycan conjugate vaccine.^[34]^

## Results and Discussion

The last two decades have witnessed a number of reports on the successful step‐saver synthesis of 2‐acetamido‐2‐deoxy‐glycopyranosides.^[35]^ Yet, direct chemical methods whether in the form of electrophilic routes involving 2‐acetamido glycosyl donors or based on the anomeric O‐alkylation of 2‐acetamido hemiacetals are still rarely applied to achieve complex oligosaccharides. In this context, we engaged in a strategy aimed at a suitable AB disaccharide building block in which the 4_B_‐amino group and 2_B_‐acetamido group were masked as a 4_B_‐azide and 2_B_‐trichloroacetamide as previously described.^[24]^ However, instead of the original 2_A_‐trichloroacetamide moiety, we thought to consider a 2_A_‐*N*‐protecting group orthogonal to TCA to reach a 2_A_‐NAc/2_B_‐NTCA AB brick. As an attempt to avoid the manipulation of altruronate‐bearing intermediates under harsh acidic or basic conditions, *N*‐protecting groups cleavable under mild basic conditions, and therefore a priori compatible with the altruronic moiety in the generic disaccharide **6**, were found attractive. In this context, the 2_A_‐*N*‐tetrachlorophthaloyl (TCP)^[36]^ altrosaminide **10** and its 2_A_‐*N‐(*9‐fluorenylmethoxycarbonyl) (Fmoc)^[37]^ analogue **11** were investigated as precursors to the AB intermediate **6** (Scheme 1, Route 2). Otherwise, as an attempt to overcome further hydrodechlorination‐related issues, the AAT precursor **9** featuring a 2_B_‐acetamide masked in the form of an *N*‐dichloroacetyl (DCA)^[38]^ was considered as a possible improvement to the known *N*‐TCA acceptor **8**.^[24]^

### The 4_A_,6_A_‐*O*‐benzylidene route to the AB building block

**Scheme 2:**
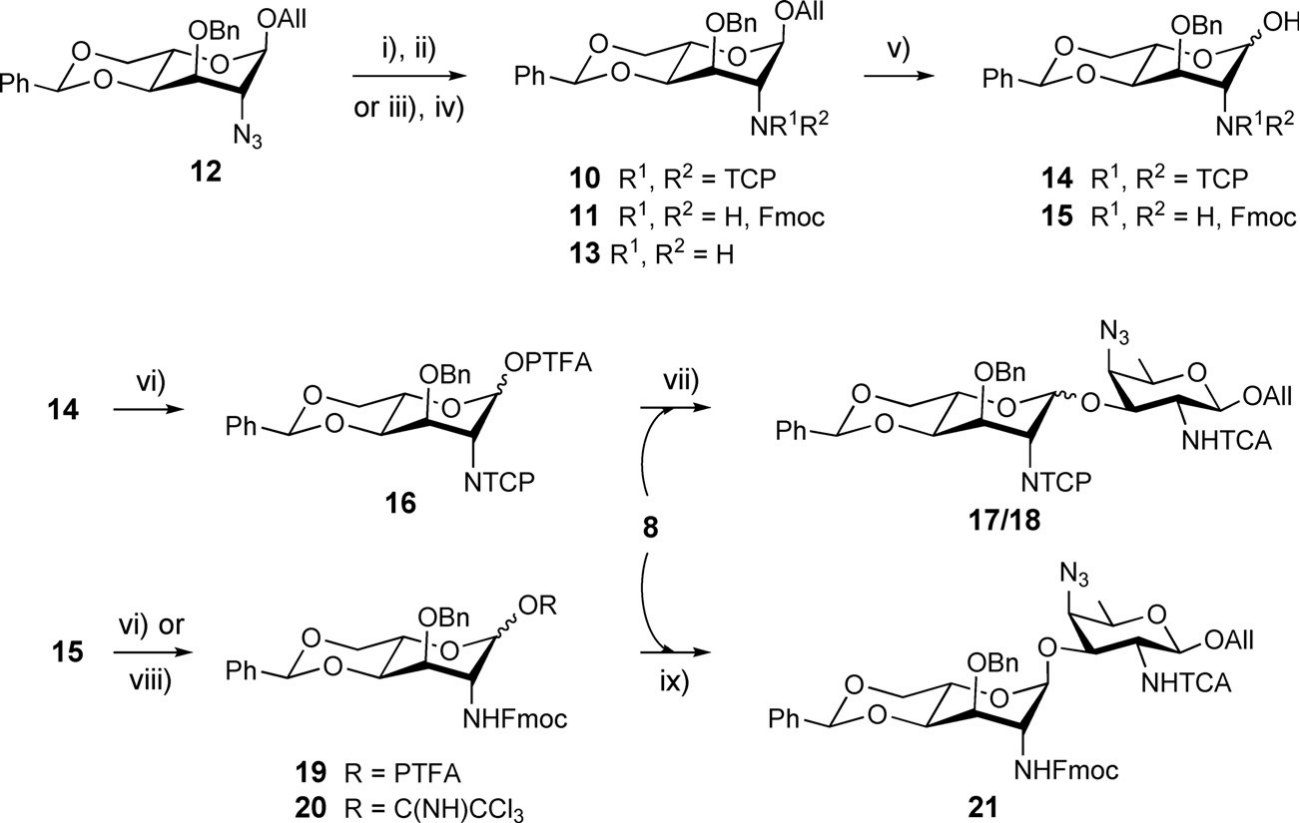
[A+B] glycosylation by use of a 4,6‐*O*‐benzylidene A donor. (i) Zn, AcOH, THF, (ii) *a*. TCPO, Et_3_N, DCE, 50 °C, *b*. Ac_2_O, py, 90 °C, 83 % over 2 steps, (iii) PPh_3_, H_2_O, THF, 60 °C, (iv) FmocCl, NaHCO_3_, DMAP, DCM, 0 °C, 88 % over 2 steps, (v) *a*. H_2_‐activated Ir‐cat, THF, *b*. I_2_, NaHCO_3_, THF/H_2_O, 43 % for **14**, and NIS, THF/H_2_O, 90 % for **15**, (vi) PTFA‐Cl, Cs_2_CO_3_, Acet, 80 % for **16**, 90 % for **19**, (vii) TMSOTf, MS 4 Å, DCE, −30 °C, 80 %, (viii) CCl_3_CN, K_2_CO_3_, Acet, quant., (ix) TMSOTf, MS 4 Å, DCE, −15 °C, 62 % (from crude donor **20**). Acet: acetone, Ir‐cat: [Ir(COD)(PMePh_2_)_2_]PF_6_. PTFA: (*N*‐phenyl)trifluoroacetimidoyl.

The synthesis of the A residue building blocks was achieved from the known 2‐azido‐l‐altroside derivative **12** (Scheme 2).^[24]^ Reduction of the azido group under Staudinger condition as described,^[24]^ provided the amine **13**, which was reacted with 9‐fluorenylmethoxycarbonyl chloride to give the *N*‐Fmoc protected analogue **11** (88 %). Analogously, the *N*‐TCP derivative **10** was isolated upon treatment of the crude **13** with tetrachlorophtalic anhydride in pyridine albeit at best in 57 % yield from the azide **12** (Scheme S1 in the Supporting Information). We reasoned that the slight excess of triphenylphosphine and the triphenylphosphine oxide formed concomitantly to amine **13** could hamper TCP installment. To our satisfaction, when changing the Staudinger conditions for a Zn/AcOH‐mediated azide reduction, the two‐step sequence gave the *N*‐TCP intermediate **10** in an improved 83 % yield (Scheme 2). Allyl glycosides **10** and **11** were submitted to Ir‐cat mediated allyl to propen‐1‐yl isomerization.^[39]^ Subsequent propenyl hydrolysis promoted by iodine in the presence of sodium bicarbonate^[31]^ furnished hemiacetal **14** in an acceptable yield. Replacing I_2_/NaHCO_3_ by *N*‐iodosuccinimide^[40]^ (NIS) enabled a smoother hydrolysis step in the case of the Fmoc analogue to furnish hemiacetal **15** in a fairly improved yield. Conventional reaction of the former with (*N*‐phenyl)trifluoroacetimidoyl chloride^[41]^ in the presence of cesium carbonate gave the (*N*‐phenyl)trifluoroacetimidate donor **16**. TMSOTf‐mediated glycosylation of the latter with the known AAT acceptor **8** at −30 °C proceeded in high yield (95 %) albeit furnishing a ≈5:1 mix of two glycosylation products in addition to the elimination product (15 %) (Scheme S2, Entry 1). Running the reaction on the gram scale resulted in lower acceptor conversion and seemingly a better stereoselectivity to furnish only one glycosylation product, while aminoglycal formation was amplified (Scheme S2, Entry 2). The elimination side‐reaction was further enhanced when the reaction temperature was increased. In contrast, the condensation reaction was not affected (Scheme S2, Entry 3). Assuming that the main glycosylation product **17** was the desired α‐linked disaccharide was tempting. Nevertheless, the sole chemical shifts of the signal of the anomeric carbons (**17**: C‐1_A_, *δ* 96.0 ppm and **18**: C‐1_A_, *δ* 97.3 ppm) did not allow to unambiguously determine the configuration at the newly established glycosidic linkages in disaccharides **17** and **18**. The ^1^ *J*_C1,H1_ at the anomeric carbons (**17**: C‐1_A_, ^1^ *J*_C,H_=173 Hz and **18**: C‐1_A_, ^1^ *J*_C,H_=173 Hz) did not permit any clear‐cut determination either, a well‐established phenomenon for compounds bearing an axial substitution at C‐2.^[42]^ The bulky TCP group at position 2 induced a distortion of the A ring in both coupling products, none of which adopts the standard ^1^ *C*_4_ chair conformation^[43]^ as attested from the vicinal ^3^ *J*_H,H_ coupling constants (Table 1 Entries 5 and 6).^[44]^ Instead, information extracted from the corresponding NOESY spectra (**17**: H‐1_A_/H‐5_A_, H‐3_B_, H‐4_B_ and **18**: H‐1_A_/H‐2_A_, H‐3_A_, H‐4_A_, H‐3_B_, H‐4_B_) permitted unambiguous characterization. The configuration at C‐1_A_ was assigned as β and α in disaccharides **17** and **18**, respectively. The chemical shifts of the anomeric protons (**17**: *δ* 6.20 ppm, *J*_1,2_=8.5 Hz and **18**: H‐1_A_, *δ* 5.35 ppm, *J*_1,2_=5.6 Hz) are misleading in this particular case. Obviously, the observed glycosylation outcome suggested that the neighboring group participation effect of the tetrachlorophtalimide moiety was far outweighed by other factors.^[45]^ Such a phenomenon is not without precedent. It was previously ascribed to solvent effect,^[46]^ steric factors,^[47]^ 3‐*O*‐protecting group directing effect^[48]^ or S_N_2‐type displacement of a less sterically demanding intermediate triflate.^[49]^

**Table 1:** Coupling constants ^3^ *J*_H,H_ and ^1^ *J*_C,H_ for the altropyranose residue (A) as monosaccharides and present in selected AB disaccharides.

Entry	Compound	^1^ *J*_C1,H1_ (Hz)	^3^ *J*_H,H_ (Hz)			
			*J*_1,2_	*J*_2,3_	*J*_3,4_	*J*_4,5_
1^[24]^	**12**	170	<1	2.9	2.9	9.4
2	**10**	172	4.0	4.0	4.4	9.6
3^a^	**11**	170	<1	–	–	8.8
4	**23**	169	7.2	11.2	3.6	3.6
5	**17** (βA)	173	8.7	3.1	2.3	9.6
6	**18** (αA)	173	5.6	4.8	4.8	8.8
7^a^	**21**	169	<1	–	–	8.0
8	**26**	171	7.6	11.2	3.9	2.8

[a] Poorly resolved spectra.

The corresponding *N*‐Fmoc donor **19** was not an option. It was found inert when treated under glycosylation conditions found suitable for its *N*‐TCP counterpart whereas harsher acidic conditions resulted in partial benzylidene loss (HRMS (ESI^+^): *m*/*z* [*M*+H]^+^: Calcd for C_36_H_34_F_3_N_2_O_7_ 663.2318; found 663.2318) before glycosylation occurred (not described). In contrast, the more reactive trichloroacetimidate **20** reacted with acceptor **8** in the presence of a catalytic amount of TMSOTf (0.05 equiv) to give the expected α‐linked glycosylation product **21** with the A ring in a conformation close to the standard ^1^ *C*_4_ chair (Table 1, Entry 7), albeit in lower yield than when using donor **16** (Scheme 2).

Having previously achieved the high yielding glycosylation of acceptor **8** ^[24]^ and others^[25]^ with the less hindered *N*‐TCA analogue of donor **16**, we reasoned that the poor glycosylation potential of altropyranosyl donors **16**, **19** and **20** could stem from the combination of conformational restriction, stereoelectronic effect^[50]^ and steric hindrance or poor anchimeric assistance owing to the superimposed influence of the 4,6‐*O*‐benzylidene acetal and 2_A_‐*N*‐protecting group. While the good propensity of the 2_A_‐NTCA for anchimeric assistance could obviously counterbalance the limitations imposed by the 4,6‐acetal moiety,^[24]^ the later may be detrimental to the glycosylation outcome.^[51]^ Altropyranose residues have a high propensity for conformational flexibility, which in the l‐series translates into a conformational equilibrium along the ^1^ *C*_4_⇌^2^ *S*_O_⇌^4^ *C*_1_ pseudo rotational itinerary. This phenomenon is noteworthy under the influence of substitutions and protecting groups.^[52]^ Yet, NMR data strongly support the assumption that when protected in the form of a 4,6‐*O*‐benzylidene acetal, altropyranosides appear to exist in a predominant conformation,^[53]^ previously identified as the ^1^ *C*_4_ conformation (Table 1, Entry 1).[^24^, ^25^] NMR data revealed that monosaccharides **10** and **11**, which carry different *N*‐protecting groups at C‐2, do not necessarily obey this trend (Table 1, Entries 2 and 3). Besides, the accessible *J* couplings for disaccharides **18** and **21** resembled those of allyl glycosides **10** and **11**, respectively (Table 1, Entries 2, 3, 6, and 7). It suggests that the conformational behavior of the 4,6‐*O*‐benzylidene‐l‐altropyranose ring is governed by the protecting group at position 2_A_ rather than by the nature of the aglycon. We reasoned that strain release through benzylidene removal would confer to the A donor properties required to achieve the desired high yielding 1,2‐*trans* glycosylation. The 4,6‐di‐*O*‐acetyl donor **25** was conceived from the 4,6‐*O*‐benzylidene precursor **12** to probe this assumption.

### Strain release at the A donor to achieve high yielding 1,2‐*trans* glycosylation

**Scheme 3:**
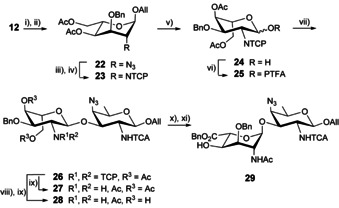
[A+B] glycosylation by use of a model 4,6‐di‐*O*‐acetyl donor **25**. (i) 80 % aq. 80 °C, (ii) Ac_2_O, py, 88 % over 2 steps, (iii) Zn, AcOH, THF, (iv) *a*. TCPO, py, DCE, 50 °C, *b*. Ac_2_O, py, 90 °C, 85 % over 2 steps, (v) *a*. H_2_‐activated Ir‐cat, THF, *b*. NIS, THF/H_2_O, 86 %, (vi) PTFA‐Cl, Cs_2_CO_3_, Acet, 86 % over 2 steps, (vii) **8**, TMSOTf, DCM, −20 °C, 88 %, (viii) NaOMe, MeOH, (ix) *a*. Ethylenediamine, MeOH, 50 °C, *b*. Ac_2_O, MeOH, 68 % over 2 steps, (x) TEMPO, BAIB, DCM/H_2_O, (xi) BnBr, K_2_CO_3_, DMF, 36 % over 2 steps. BAIB: [bis(acetoxy)iodo] benzene, TEMPO: 2,2,6,6‐tetramethylpiperidine‐1‐oxy.

Acid‐mediated 4,6‐*O*‐benzylidene hydrolysis of altroside **12** and acetylation furnished the 4,6‐diacetate **22** (Scheme S3). As described for the transformation of the precursor **12** to the corresponding PTFA donor **16**, a four‐step conversion furnished the *N*‐TCP donor **25** (Scheme 3). Glycosylation with acceptor **8** under the exact same conditions as those used with donor **16** delivered the α‐linked disaccharide **26** in 88 % yield, whether the reaction was run on 200 mg or multigram amounts. Neither the elimination product nor the β‐linked isomer was isolated. Removal of the benzylidene acetal resulted in a major switch of the A ring into a ^4^C_1_ conformation in both the monosaccharide **23** and disaccharide **26** (Table 1, Entries 4 and 8). As for the 2_A_‐NTCP and 2_A_‐NFmoc analogues, the influence of the aglycon was only minor. In spite of precedents,^[36b]^ the unmasking of the amino group of the fully protected **26** by action of ethylenediamine faced issues due to O→N acetyl group transfer providing the *N*‐acetylated side‐product (HRMS (ESI^+^): *m*/*z* [*M*+H]^+^: Calcd for C_40_H_45_Cl_7_N_7_O_13_ 1076.0895; found 1076.0918), in addition to the target **27** (Scheme S4). Instead, disaccharide **26** was subjected to Zemplén transesterification followed by treatment with ethylenediamine and subsequent selective *N*‐acetylation to give the desired diol **28**. In contrast to the high yielding conversion achieved in the case of its 2_A_‐NTCA analogue,^[24]^ the selective 2,2,6,6‐tetramethylpiperidine‐1‐oxy (TEMPO) radical/[bis(acetoxy)iodo] benzene (BAIB)^[54]^ oxidation of the primary alcohol of diol **28** and subsequent benzyl esterification into the uronate acceptor **29** was low yielding (Scheme 3). We hypothesized that the poor solubility of the 2_A_‐acetamide **28** in organic solvents was a major limiting factor, impairing smooth transformations.

### The 4_A_,6_A_‐orthogonally protected A route to a 2_A_‐acetamido AB building block

**Scheme 4:**
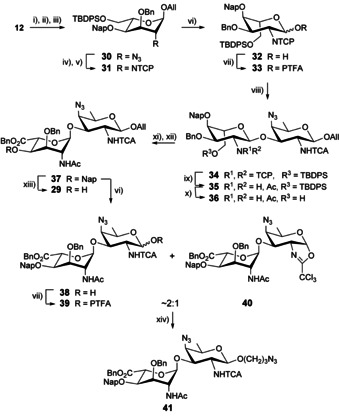
Synthesis of the AB azidopropyl glycoside **41** by means of a [A+B] glycosylation using the orthogonally protected A donor **33**. (i) CSA, MeOH/DCM (4:1, v/v), (ii) TBDPSCl, Imidazole, DMF, (iii) NapBr, NaH, DMF, 0 °C, 85 % over 3 steps, (iv) Zn, AcOH, THF, (v) *a*. TCPO, Et_3_N, DCM, *b*. Ac_2_O, Py, 80 °C, 82 % over 2 steps, (vi) *a*. H_2_‐activated Ir‐cat, THF, *b*. NIS, THF/H_2_O, 82 % for **32**, 92 % for **38**, (vii) PTFA‐Cl, Cs_2_CO_3_, Acet, quant. for **33**, 89 % for **39/40** (∼2:1), (viii) **8**, TMSOTf, DCM, −15 °C, 96 %, (ix) *a*. Ethylenediamine, MeOH/THF (1:1, v/v), 50 °C, *b*. Ac_2_O, MeOH, 94 %, (x) TBAF, THF, 86 %, (xi) TEMPO, BAIB, DCM/H_2_O, (xii) BnBr, K_2_CO_3_, DMF, 85 % over 2 steps, (xiii) DDQ, DCM/Phosphate buffer pH 7 (6:1, v/v), 0 °C to rt, 87 %, (xiv) 3‐Azidopropanol, Yb(OTf)_3_, DCM, 0 °C, 78 %.

The use of the 4,6‐diacetate **25** had facilitated a high yielding [A+B] glycosylation. Yet, difficulties met during the conversion of the model coupling product **26** into the desired uronate **29** encouraged the investigation of a more advanced protecting group strategy. Toward this aim, we set to design a novel orthogonally protected donor A fulfilling stability and solubility criteria as well as orthogonality requirement post glycosylation with acceptor **8** (Scheme 4). Thus, allyl altropyranoside **12** was subjected to sequential acidic benzylidene hydrolysis, masking of the liberated primary hydroxyl of the resulting diol in the form of a *tert*‐butyldiphenylsilyl (TBDPS) ether and 2‐naphthylmethyl (Nap) alkylation of the remaining free hydroxyl group, which afforded the orthogonally protected **30** in 85 % yield over three steps. Zn/AcOH‐mediated azide reduction of the latter and conventional *N*‐protection furnished the NTCP derivative **31** in a yield comparable to that of the 4,6‐diacetate **23** following a similar sequence of reduction/protection reaction. The corresponding hemiacetal **32** (82 %) was obtained as described for the preparation of the 4,6‐diacetate **24**. TMSOTf‐promoted glycosylation of acceptor **8** and the crude PTFA donor **33** issued from hemiacetal **32** (1.1 equiv) furnished the α‐linked disaccharide **34** as the sole product. This outcome supported our original assumption that strain release at the A residue governed the glycosylation outcome in spite of the excellent neighboring group potential of the TCP group.

The next step consisted in fashioning disaccharide **34** into ready‐for‐oligomerization AB donor and acceptor. Treatment with ethylenediamine in a mix of methanol and THF selectively liberated the 2‐amino analogue, which was smoothly *N*‐acetylated into derivative **35** (94 %). The TBDPS group was cleaved upon reaction with excess TBAF in THF to give alcohol **36**,^[55]^ which was subjected to TEMPO/BAIB oxidation and benzyl esterification. The key benzyl ester **37** was isolated from the intermediate **35** (73 %). Introducing orthogonality at the level of monosaccharide A resulted in a notably improved overall yield for the synthesis of the AB building block **37** from the AAT acceptor **8** and the 4,6‐*O*‐benzylidene altropyranoside **12**, 39 % and 12 steps via donor **33** instead of 14 % and 11 steps to intermediate **29** via donor **25**, respectively. Exposure of the orthogonally protected **37** to oxidative cleavage of the 4‐*O*‐Nap produced alcohol **29** in good yield. Alternatively, disaccharide **37** was deallylated into hemiacetal **38**, itself smoothly converted into a ∼2:1 mix of PTFA **39** and oxazoline **40** (82 % over two steps). Yb(OTf)_3_‐promoted glycosylation of the latter with a simple model acceptor—3‐azidopropanol—in DCM at 0 °C delivered the AB disaccharide **41** in a good 78 % yield (Scheme 4), suggesting that the **39**/**40** mix fulfilled donor criteria.

**Scheme 5:**
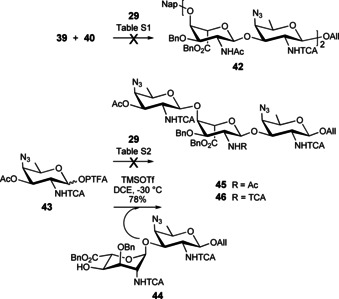
Investigation on the acceptor properties of the AB disaccharide **29**.

In contrast, attempts at AB oligomerization by use of disaccharide **29** as the glycosyl acceptor in combination with donor **39**/**40** failed repeatedly (Scheme 5, Table S1). At best, traces of the desired glycosylation product **41** were formed despite the large number of promoters and range of temperatures being tested, including heating at 70 °C in DCE as found previously appropriate for the glycosylation of poorly reactive acceptors.^[9a]^ As an attempt to understand the origin of the poor [**39**/**40**+**29**] glycosylation outcome, the AB acceptor **29** was treated with the known AAT donor **43**.^[24]^ Glycosylation in the presence of various triflate promoters resulted exclusively in the formation of the oxazoline (not described) instead of the expected **45** (Scheme 5, Table S2). This contrasted with our precedent observations whereby PTFA **43** proved to be a suitable donor in the synthesis of the ABA trisaccharide **46** involving the 2_A_‐NTCA/2_B_‐NTCA analogue (**44**) of disaccharide **29** as acceptor.^[24]^ We reasoned that the donor properties of the AB precursor **39**/**40** were not to be questioned. In contrast, our data suggest that alcohol **29**, which only differs from the previously used **44** by the 2_A_‐acetamido moiety, is a poor acceptor.

### From a 2_A_‐acetamido AB intermediate to a ready‐for‐oligomerization 2_A_‐(*N*,*N*‐diacetyl)amino AB building block: conformational distortion into play

Though in the altruronic configuration, the reactive glycosylation site at C‐4_A_ and the acetamido substitution at C‐2_A_ obey a 1,3‐*trans* relationship. We hypothesized that as for the most studied 2‐*N*‐acetyl‐glucosaminides, the 2_A_‐NAc moiety could be responsible for the lower reactivity of OH‐4 in disaccharide **29**. Despite some successful achievements,^[56]^ the poor glycosyl acceptor properties of hydroxyl groups, especially OH‐4, of partially protected *N*‐acetyl‐glucosamine were often underlined. In many instances, they could be ascribed to the amide N‐H capacity to enter intra‐ and intermolecular hydrogen bonding.^[57]^ While an appropriate selection of the protecting groups at the vicinal position of the 2‐acetamido moiety can disrupt the former to some extent, the latter phenomenon is favored at low temperature and higher concentration.^[57]^ The propensity of residues featuring a 2‐acetamido moiety distal to the reactive acceptor center to impair glycosylation has also been noted repeatedly. In particular, the formation of the 2‐*N*‐glycosyl imidate side‐products, whether stable or subsequently hydrolyzed during column chromatography, was underlined as a major interfering process.^[58]^ With this in mind, the outcome of the [**39**/**40**+**29**] glycosylation encouraged the investigation of novel orthogonally protected AB building blocks fulfilling oligomerization criteria. As masking the 2_A_‐acetamido moiety in disaccharide **29** was mandatory, we favored imide‐type protection enabling the direct 2_A_‐acetamide recovery under mild conditions.

In line with previous achievements in the synthesis of neuraminic acid containing oligosaccharides,^[59]^ the masking of the acetamide moiety of glucosamine in the form of the corresponding *bis*‐acetylated imide was adopted successfully on several occasions,[^57^, ^58b^, ^60^] including in the synthesis of an undecasaccharide featuring a Kdo_2_GlcNAc_2_ backbone.^[58d]^ The acetamido function is readily recovered upon treatment of the elongated intermediate under mild alkaline conditions, most often Zemplén conditions,^[58b]^ without any manipulation of the amine. This path was thought to be consistent with our original vision.^[24]^

**Scheme 6:**
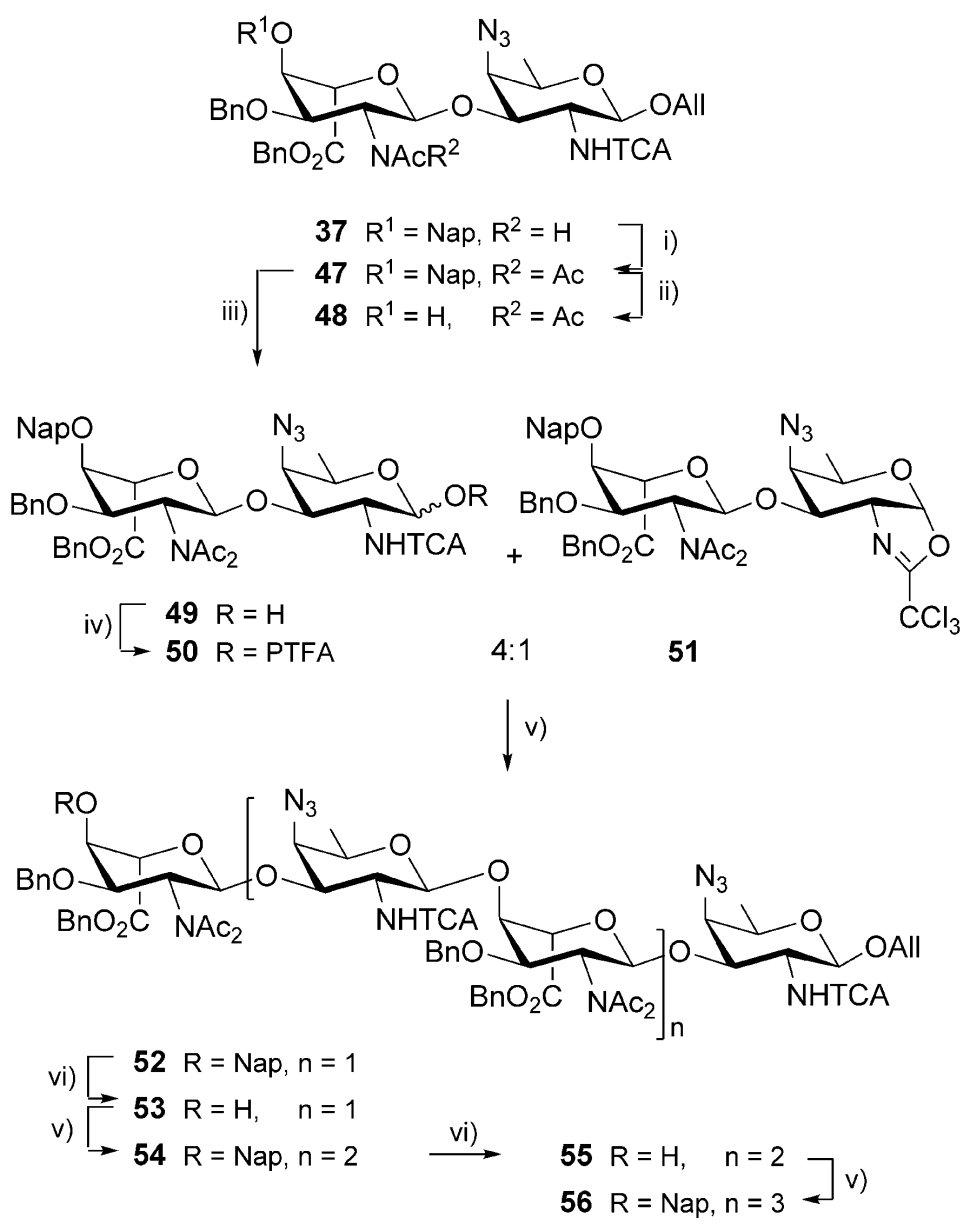
Synthesis of AB oligomers by means of the key 2_A_‐NAc_2_/2_B_‐NTCA disaccharide **47**. (i) AcCl, *i*Pr_2_NEt, DCM, 90 %, (ii) DDQ, DCM/Phosphate buffer pH 7 (10:1, v/v), 93 %, (iii) *a*. H_2_‐activated Ir‐cat, THF, *b*. NIS, THF/H_2_O, 90 %, (iv) PTFA‐Cl, Cs_2_CO_3_, Acet, 80 %, (v) Crude **50**/**51**, TfOH, DCM, 0 °C, 62 % (corrected 85 %) for **52** from **48**, 71 % (corrected 98 %) for **54**, 33 % (corrected 54 %) for **56**, (vi) DDQ, DCM/Phosphate buffer pH 7 (8:1, v/v), 0–10 °C, 72 % for **53**, 64 % for **55**. TfOH: trifluoromethanesulfonic acid.

To increase the acceptor potential of disaccharide **29** towards glycosylation at O‐4_A_, the fully protected **37** was selectively bis‐*N*‐acetylated at position 2_A_ in the presence of Hünig base and a controlled excess of acetyl chloride to afford the key intermediate **47** in over 90 % yield (Scheme 6). Next, the Nap group was oxidatively cleaved in a buffered system to efficiently liberate OH‐4_A_ and provide alcohol **48**. Available NMR data revealed that the A residue present in the bis‐*N*‐acetyl AB disaccharide, whether the fully protected **47** or acceptor **48**, adopted predominantly a ^4^ *C*_1_ conformation (Table 2, Entries 4 and 5). In contrast, the corresponding 2_A_‐acetamido derivatives **37** and **29** exist in a distorted conformation varying as a function of the protecting pattern at O‐3_A_ (Table 2, Entries 2 and 3). As a result, glycosylation with acceptor **48** involves an axial 4_A_‐OH. Nevertheless and confirming the improved acceptor properties of the latter over the 2_A_‐acetamido acceptor **29**, reaction with the available 3_A_‐*O*‐benzyl analogue of donor **39/40** gave the expected glycosylation product in a non‐optimized 34 % yield (Scheme S5). In spite of its impressive influence on the glycosylation potential of OH‐4_A_, the nature of the 2_A_‐acetamide (NAc versus NTCA) seems to have only a minor conformational impact (Table 2, Entries 1 and 3), suggesting that other parameters come into play.

**Table 2:** Coupling constants ^3^ *J*_H,H_ and ^1^ *J*_C,H_ for the 2‐amino‐2‐deoxy‐Alt*p*A unit (A) present in selected AB disaccharides.

Entry	AB disaccharide	^1^ *J*_C1,H1_ (Hz)	^3^ *J*_H,H_ (Hz)			
			*J*_1,2_	*J*_2,3_	*J*_3,4_	*J*_4,5_
1^[24]^	**44**	170	3.3	5.4	3.6	7.6
2	**37**	169	5.6	–	2.7	4.6
3	**29**	170	3.2	5.0	3.5	7.8
4	**47**	176	7.8	10.5	2.9	2.2
5	**48**	174	7.9	10.3	3.4	2.3

Disaccharide **47** was subjected to a two‐step anomeric deallylation process to achieve hemiacetal **49** (90 %), which was converted into a 2_A_‐NAc_2_,2_B_‐NTCA AB donor. The latter was isolated as a ∼4:1 mix of PTFA **50** and oxazoline **51** in 80 % yield, which suggested a high sensitivity to purification conditions and encouraged its use as a crude material. The trifluoromethanesulfonic acid (TfOH)‐promoted [**48**+**50**/**51**] glycosylation was performed in DCM at 0 °C. As expected, the 2_A_‐NAc_2_ acceptor **48** showed a higher reactivity than its 2_A_‐NAc counterpart **29**. These non‐optimized conditions delivered the desired tetrasaccharide **52** in a good yield. As only a minimal amount of the crude donor—issued from stoichiometric amounts of hemiacetal **49**—was used, some unreacted acceptor was recovered (37 %).

Whereas the ^3^ *J*_1,2_ coupling constant characterizing the newly formed B^1^‐A glycosidic linkage (^3^ *J*_1,2_ (B^1^)=8.0 Hz) corroborated the β‐anomeric configuration of the intrachain AAT unit, the corresponding heteronuclear ^1^ *J*_C,H_ coupling constant had an unusual high value (^1^ *J*_C1,H1_ (B^1^)=168 Hz), superior to the ^1^ *J*_C,H_ coupling constant of the AAT residue at the reducing end (^1^ *J*_C1,H1_ (B)=163 Hz), revealing a more constrained environment at the B^1^‐A linkage. Moreover, the high values measured at the A^1^‐B^1^ and A‐B linkages (^1^ *J*_C1,H1_ (A^1^)=176.5 Hz, ^1^ *J*_C1,H1_ (A)=177 Hz) also supported a highly constrained protected [AB]_2_ tetrasaccharide whereby the internal pyranose rings adopted a distorted conformation. Data portraying the fully protected 2_A_‐NAc_2_ AB building block **47** (Table 2, Entry 4) revealed a similar propensity for conformational distortion, which was also visible albeit to a lesser extent in acceptor **48** (Table 2, Entry 5). In contrast, the phenomenon is absent in the 2_A_‐acetamido precursor **29** (Table 2, Entry 3) or its azidopropyl analogue **41** (^1^ *J*_C1,H1_ (A)=171 Hz, Table S3, Entry 19), suggesting that the bis‐*N*‐acetyl protecting pattern is the sole responsible for this phenomenon.

### The 2_A_‐(*N*,*N*‐diacetyl)amino AB route to [AB]_*n*_ oligomers

The unmasking of the acceptor center at the terminal residue and subsequent glycosylation of the obtained **53** with the crude **50**/**51** in the presence of catalytic TfOH as described above, furnished hexasaccharide **54** in a good 71 % yield (Scheme 6) together with some unreacted **53** (28 %). The use of a slight excess of donor **50**/**51** (1.25 equiv instead of 1.0 equiv) based on hemiacetal **49** contributed to an improved yield of the [**50/51**+**53**] glycosylation in comparison to the [**50/51**+**48**] coupling, 71 % and 62 %, respectively. An additional round of Nap cleavage and subsequent glycosylation of hexasaccharide **55** with disaccharide **50**/**51** provided octasaccharide **56**. Insights from the ^1^H NMR spectra for compounds **52**–**56** revealed a clear propensity of signals from internal residues toward overlap as the number of repeating units within the oligosaccharides increased. Chemical shifts nicely picture this trend (**52**: *δ* (ppm) 5.78 (H‐1_A1_), 5.65 (H‐1_A_), 4.76 (H‐1_B_), 5.00 (H‐1_B1_), **54**: *δ* (ppm) 5.78 (H‐1_A2_), 5.65/5.64 (H‐1_A_/H‐1_A1_), 4.76 (H‐1_B_), 5.03/4.99 (H‐1_B1_/H‐1_B2_), **56**: *δ* (ppm) 5.78 (H‐1_A3_), 5.66–5.62 (H‐1_A_/H‐1_A1_/H‐1_A2_), 4.77 (H‐1_B_), 5.03–5.01/4.96 (H‐1_B1,_ H‐1_B2_, H‐1_B3_)) suggesting that the anomeric protons evolve within a similar environment distinct from that of the end chain residues.

Having identified disaccharide **47** as a suitable building block for oligomerization, we set up to investigate deprotection conditions. Octasaccharide **56** features eight acetamido moieties, half of which are masked as *N*‐TCA while the other half appear in the form of bis‐*N*‐acetyl. Selective recovery of the 2_A_‐acetamido moieties from the 2_A_‐NAc_2_ precursors was attempted under a diversity of mild basic conditions using disaccharides **47** and **48**, and tetrasaccharide **52** as model systems. The altruronate units did not resist any of the assayed conditions. Transesterification of the benzyl esters into the corresponding methyl esters was observed repeatedly while formation of the 4_A_:5_A_ unsaturated side‐product resulting from glycosidic cleavage at position 4_A_, could not be avoided at the tetrasaccharide level (Scheme S6). The A ring in the 2_A_‐NAc_2_ methyl esters derivative tended toward a ^4^ *C*_1_ chair conformation (Table S3, Entry 23), whereas the 2_A_‐acetamido derivative adopted a more distorted conformation (Table S3, Entry 20) as also observed for the benzyl ester analogues whether protected at position 3_A_ as in disaccharides **65**, **37**, **41** or in the form of acceptor **29** (Table S3, Entries 18, 19, Table 2, Entries 2 and 3).

### Use of an acid‐sensitive acetamide camouflage as a possible alternative to the 2_A_‐(*N*,*N*‐diacetyl)amino AB strategy

**Scheme 7:**
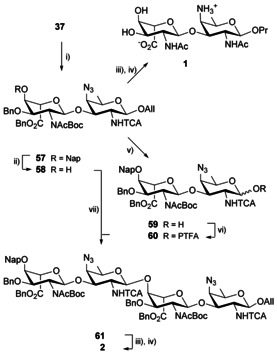
Synthesis, full deprotection and attempted oligomerization of the 2_A_‐NAcBoc/2_B_‐NTCA disaccharide **57**. (i) Boc_2_O, DMAP, THF, 73 %, (ii) DDQ, DCM/Phosphate buffer pH 7 (10:1, v/v), 79 %, (iii) TFA, DCM, (iv) H_2_, Pd(OH)_2_, *t*BuOH/DCM/H_2_O, 71 % over two steps from **57** for **1** and 37 % from **61** over two steps for **2**, (v) *a*. H_2_‐activated Ir‐cat, THF, *b*. NIS, THF/H_2_O, 87 %, (vi) PTFA‐Cl, Cs_2_CO_3_, Acet, 86 %, (vii) Crude **60**, TfOH, DCM, 0 °C, 41 %. Boc: *tert*‐butyloxycarbonyl.

The observed high sensitivity of the altruronate residue to mild basic treatment encouraged further investigation on the masking of the 2_A_‐acetamido moiety in disaccharide **37**. Our concern was that side‐reactions around the uronate moiety would increase as the oligomers grew larger. Of interest was the *tert*‐butyloxycarbonyl (Boc) group. In contrast to most other *N*‐protecting groups, *N*‐Boc is sensitive to acid hydrolysis. Glucosamine derivatives encompassing a 2‐*N*‐benzyloxycarbonylacetamido moiety have been described previously,^[61]^ but to our knowledge were barely involved in glycosylation reactions. Rather, they serve as transient intermediates from the 2‐acetamide precursors to the 2‐amino targets,^[61]^ the latter being eventually converted into a versatile 2‐amino‐glycosyl donor.^[62]^ Herein, treatment of disaccharide **37** with di‐*tert*‐butyldicarbonate and catalytic DMAP in THF led to the selective 2_A_‐*N*‐carbamoylation to produce imide **57** (Scheme 7). The conversion was characterized by a characteristic concomitant switch of residue A from a distorted conformation in the 2_A_‐acetamido disaccharide **37** toward a ^4^ *C*_1_ chair in the obtained 2_A_‐NAcBoc‐bearing disaccharide **57**, as also observed for the corresponding 2_A_‐NAc_2_ imide **47** (Table 2, Entries 2 and 4, Table S3, Entry 21). As anticipated, the selective recovery of the 2_A_‐acetamide by trifluoroacetic acid (TFA) cleavage of the Boc group from imide **57** to release intermediate **37** was high yielding. Hydrogenation of the latter in the presence of Pearlman's catalyst^[63]^ achieved the concomitant cleavage of the Bn and Nap ether, reduction of the azide and anomeric allyl moiety, and hydrodechlorination of the 2_B_‐trichloroacetamide to produce the desired zwitterionic disaccharide **1** in a rewarding 71 % yield over two steps post RP‐HPLC purification (Scheme 7). This notable increase over our initial report, whereby the propyl glycoside **1** stemmed from the 2_A_‐NTCA/2_B_‐NTCA intermediate **44** (46 %),^[24]^ supported our hypothesis that variation around the 2_A_‐*N*‐protecting group selection would facilitate the recovery of the free oligosaccharides.

Alternatively, the key intermediate **57** was converted in good yields to the corresponding alcohol **58** and PTFA analogue **60** by means of hemiacetal **59** as described for imide **47**. Yet, in contrast to the [**50**/**51**+**48**] glycosylation, the [**58**+**60**] coupling was low‐yielding, providing the desired tetrasaccharide **61** in at best 41 % yield. Otherwise, the latter was engaged in the two‐step deprotection process successfully experimented on the disaccharide analogue **57** to give the targeted tetrasaccharide **2** (Scheme 7) in an underestimated 37 % yield. NMR analysis revealed a trend for the ^1^ *J*_C1,H1_ (A) coupling constants of the product of glycosylation **57** and acceptor **58**–176 Hz and 174 Hz (Table S3, Entries 21 and 22), respectively—similar to that measured for the 2_A_‐NAc_2_ AB disaccharides **47** and **48** (Table 2). Moreover, missing (**57**: C‐2_A_, **55**: C‐2_A_), barely seen (**57**: CO_Ac_, CO_Boc_) and unusually broad (**57**: C‐3_A_, C‐3_B_, **58**: CO_Ac_, CO_Boc_, C‐3_A_) ^13^C signals provided additional support to the assumption that in solution at room temperature, AB building blocks comprising a 2_A_‐NAcBoc moiety exhibit a highly restricted conformational flexibility in the vicinity of the A‐B glycosidic linkage. We hypothesized that conformational restrictions could in part explain the poor glycosylation outcome. Owing to anticipated similar issues at each glycosylation step, this route was left aside despite the high yielding final conversion of disaccharide **57** into the known propyl glycoside **1**.

### Attempt at overcoming further hydrodechlorination issues: the 2_B_‐NDCA AB strategy

**Scheme 8:**
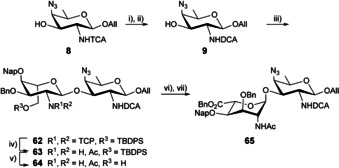
Synthesis of the AB building block **65** by means of a [A+B] glycosylation using the orthogonally protected A donor **33** and acceptor **9**. (i) LiOH.H_2_O, Acet/H_2_O (2:1, v/v), 50 °C, (ii) DCA‐Cl, Et_3_N, ACN, 68 % over two steps, (iii) Crude **33**, TMSOTf, ACN, −15 °C, 67 %, (iv) *a*. Ethylenediamine, MeOH/THF (1:1, v/v), 50 °C, *b*. Ac_2_O, MeOH, 87 %, (v) TBAF, THF, 80 %, (vi) TEMPO, BAIB, DCM/H_2_O, (vii) BnBr, K_2_CO_3_, DMF, 61 % over 2 steps. ACN: acetonitrile.

Otherwise, as an additional attempt to improve the 2_A_‐imide route disclosed in Scheme 6, we considered diminishing further the number of chlorine atoms to be exchanged as part of the late‐stage conversion into the free oligosaccharides. Toward this aim, we envisioned the use of the AAT alcohol **9** featuring a 2‐NDCA moiety in place of the closely related trichloroacetamide **8**. Owing to its feasible direct conversion into an acetyl group by catalytic hydrodechlorination, the DCA group was adopted for the protection of the amine function in the synthesis of alkali labile aminosugar disaccharides.^[64]^ While being less popular than other *N*‐protecting groups encountered in oligosaccharide synthesis,^[26]^ including TCA, it is also well‐suited for anchimeric assistance.^[65]^ The DCA group fulfilled all criteria relevant to our original strategy featuring, as a fundamental concept, a final catalytic hydrogenolysis to unmask all amine, acetamide, alcohol and uronic acid moieties in addition to aglycon reduction, and provide the desired propyl glycosides in a single deprotection step. We thus set out to target the 2_B_‐NDCA AB building block **65** (Scheme 8).

The *N*‐TCA moiety in acceptor **8** was cleaved by treatment with lithium hydroxide and the released free amine was in turn set to react with dichloroacetyl chloride,^[64]^ which furnished dichloroacetamide **9** (Scheme 8). The properties of the *N*‐DCA acceptor **9** differ somewhat from that of its *N*‐TCA counterpart. Owing to its significant reduced solubility in chlorinated solvents and toluene, glycosylation of alcohol **9** with donor **33** was performed in acetonitrile in the presence of catalytic TMSOTf to give the sole α‐isomer **62** (67 %). The smooth *N*‐TCP to acetamide exchange furnished intermediate **63**. The latter was subjected to TBDPS removal with TBAF to give alcohol **64**. Conversion into the benzyl uronate **65** was carried out as for its analogue **37**, albeit in a lower yield over three steps, 49 % and 73 %, respectively. The drastic difference in the solubility of the DCA intermediates with respect to their TCA analogues noticeably impaired the easy manipulation of the former and building block **65** was not investigated further.

**Scheme 9:**
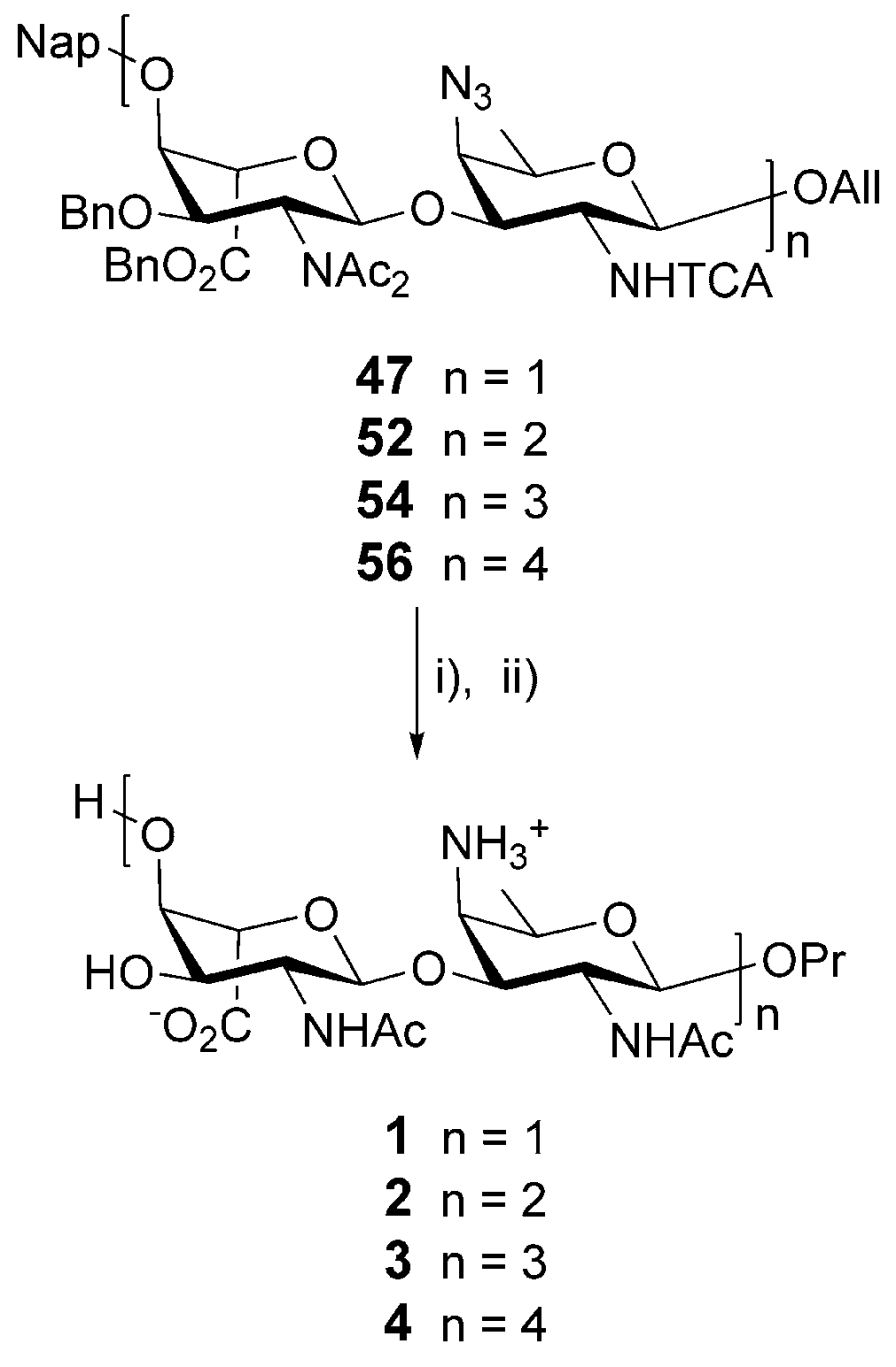
Synthesis of the *S. sonnei* zwitterionic di‐ to octasaccharides **1**–**4**. (i) Pd(OH)_2_/C, H_2_, *t*BuOH/DCM/H_2_O, (ii) NH_2_OH, MeOH, 57 % for **1**, 39 % for **2**, 31 % for **3**, and 16 % for **4** over two steps.

This outcome led us to focus on the strategy featuring the more promising 2_A_‐NAc_2_/2_B_‐NTCA AB building block **47**. Issues met when attempting the 2_A_‐NAc_2_ to 2_A_‐NAc conversion under mild basic conditions (Scheme S6) urged us to reconsider our leading “single step full deprotection” concept. We set to investigate a two‐step deprotection strategy whereby *N*‐deacetylation at position 2_A_ would only occur post benzyl ester cleavage (Scheme 9). As a follow up of our previous achievements,[^24^, ^28b^] focus was on the Pd(OH)_2_/C‐catalyzed hydrogenolysis of the benzyl, naphthyl, and TCA groups and concomitant reduction of the azido and allyl moieties from the fully protected intermediates. A two‐step transformation supported by LC‐MS/HRMS monitoring to ensure full hydrodechlorination, and subsequent *N*‐deacetylation of the crude intermediate gave the desired AB propyl glycoside **1** in a good 57 % yield from disaccharide **47**. The same conditions applied to tetrasaccharide **52** and hexasaccharide **54** furnished the more complex [AB]_2_ and [AB]_3_ zwitterionic targets in 6 to 12 mg amount, in a moderate 39 % and 31 % yield post RP‐HPLC purification, respectively. Extrapolation to the fully protected **56** also exemplified the feasible two‐step conversion to the more demanding [AB]_4_ zwitterionic octasaccharide. These achievements open for the first time the way to large oligosaccharides representative of the unique *S. sonnei* and *P. shigelloides* O17 ZPS antigens.

## Conclusions

In this study, we have embarked on the block wise synthesis of oligosaccharides encompassing multiple repeating units of the ZPSs from *S. sonnei*. To reach our objective, we have reinvestigated the protecting group pattern of a suitable ready‐for‐oligomerization disaccharide building block, corresponding to the AB repeating unit. Aiming at avoiding the extensive hydrodechlorination associated to the necessary conversion of multiple trichloroacetamides into acetamides featured in the originally designed 2_A_‐NTCA,2_B_‐NTCA AB brick,^[24]^ alternative routes were explored, whilst a final hydrogenation/hydrogenolysis step guided the overall strategy. Owing to a strong associated risk of 4:5‐elimination,^[24]^ the repeated occurrence of the 4‐*O*‐glycosylated Alt*p*NAcA moiety in the targeted [AB]_*n*_ oligomers—one in two glycosidic linkages—also governed our reasoning. AB bricks featuring up to seven different *O*‐ and *N*‐protecting groups were evaluated in the context of glycosylation, oligomerization, and full deprotection. Issues such as ring strain, conformational switch, steric hindrance, inadequate solubility, and poor acceptor reactivity met while the work was in progress shed light on the complex balance between criteria governing protecting group selection in the synthesis of oligosaccharides featuring multiple aminodeoxy sugars. For example, changing the 2‐NTCA protecting group to the 2‐NDCA equivalent resulted in a significantly reduced solubility of acceptor **9** versus **8**, which was not compensated at the disaccharide stage. Otherwise, fine‐tuning focused on the A residue. A lightly protected 2_A_‐NAc,2_B_‐NTCA AB acceptor was primarily considered. This route was unsuccessful owing to the poor acceptor capability of the 4_A_‐OH in the simplest disaccharide **29**. This was tentatively correlated to the 2_A_‐NAc moiety and called for alternatives. We favored the implementation of a 2_A_‐NAcR,2_B_‐NTCA AB building block featuring an acid‐ or mild base‐sensitive protecting group R to ensure compatibility with the uronate moiety while enabling the direct recovery of the 2_A_‐acetamide without the need for *N*‐acetylation of the free amine. With the orthogonally protected **37**—the precursor to acceptor **29**—serving as the key AB intermediate, divergence occurred at an advanced stage. Two routes were undertaken, involving either the key disaccharide **57** (R=Boc) or the corresponding **47** (R=Ac). *N*‐Masking of the 2_A_‐acetamide in **37** caused a significant change in the conformation adopted by the A residue to reach predominantly a ^4^ *C*_1_ conformation in the 2_A_‐NAcR,2_B_‐NTCA intermediates **57** and **47**. While the two routes provided the [AB]_2_ tetrasaccharide **2**, the second option that engaged the 2_A_‐(*N*,*N*‐diacetyl)amino AB **47** was found superior. *N*‐deacetylation was performed post hydrogenolysis. Donor A was revised to comply with the unique conformational behavior of altropyranose residues and produce **37** in high yield. Selection was mostly governed by the 2_A_‐*N*‐substitution, which had to be orthogonal to the 2_B_‐NTCA moiety. The orthogonally protected 2‐NTCP donor **33** fulfilled both orthogonality and glycosylation criteria, in contrast to its 4,6‐*O*‐Bzl equivalent **16**. On several occasions, NMR data revealed the strong influence of the substitution pattern on the ^1^C_4_⇌^2^S_O_⇌^4^C_1_ equilibrium characterizing altropyranose residues. While introducing bulkiness at C2 had the most striking impact, glycosylation also had a meaningful effect, as underlined upon chain elongation. These strategic improvements resulted in an effective route to *S. sonnei* oligosaccharides encompassing several repeating units. For the first time, a tetra‐, a hexa‐, and an octasaccharide, featuring two, three, and four biological repeating units, respectively, were achieved in addition to an improved synthesis of the AB repeating unit. Those and the feasibility of larger fragments of the *S. sonnei* ZPSs add to the available arsenal of synthetic well‐defined zwitterionic oligosaccharides. They open the way to a detailed molecular investigation of the propensity of short oligosaccharides to act as antigenic, conformational, and structural mimic of the full length *S. sonnei* ZPSs.^[34a]^ The gained knowledge will strengthen the exploration of the immunological properties of these puzzling ZPSs and the structure‐guided design of a *S. sonnei* synthetic glycan conjugate vaccine.

## Experimental Section

**Allyl 2‐azido‐3‐*O*‐benzyl‐6‐*O*‐*tert*‐butyldiphenylsilyl‐2‐deoxy‐4‐*O*‐(2‐naphthylmethyl)‐α‐l‐altropyranoside (30)**: CSA (4.1 g, 17.7 mmol, 0.5 equiv) was added to acetal **12** (15.0 g, 35.4 mmol, 1.0 equiv) in MeOH/DCM (4:1, 170 mL). After stirring at rt for 2 h, a TLC follow up (Tol/EtOAc 4:1) indicated reaction completion as shown by the absence of the starting **12** (*R*_f_ 0.65) and the presence of a very polar spot (*R*_f_ 0.0). 5 % Aq. NaHCO_3_ (300 mL) was added followed by EtOAc (500 mL). The organic phase was separated and washed with brine (500 mL). The organic phase was dried over Na_2_SO_4_ and concentrated under reduced pressure. The crude product was dried under high vacuum. *tert*Butyldiphenylchlorosilane (10.1 mL, 38.9 mmol, 1.1 equiv) and imidazole (3.1 g, 46.0 mmol, 1.3 equiv) were added to the crude diol in anhyd. DMF (180 mL) at 0 °C. The reaction mixture was allowed to reach rt slowly and stirred overnight at this temperature. Methanol (10.0 mL) was added and after 30 min, volatiles were evaporated under reduced pressure. The residue was dissolved in EtOAc (500 mL) and the organic layer was washed with 90 % aq. brine (500 mL), separated, dried over Na_2_SO_4_, and concentrated. 2‐(Bromomethyl)naphthalene (10.9 g, 49.6 mmol, 1.4 equiv) was added to the crude intermediate in DMF (230 mL). The solution was cooled to 0 °C and NaH (60 % in mineral oil, 1.7 g, 70.8 mmol, 2.0 equiv) was added portionwise. After stirring vigorously for 2 h while the bath temperature slowly reached rt, a TLC follow up indicated reaction completion. The reaction mixture was diluted with DCM (1 L) and 5 % aq. NH_4_Cl (500 mL) was added. The organic layer was washed with water (1.5 L) and brine (1 L), dried over Na_2_SO_4_ and concentrated. The crude product was purified by flash chromatography (cHex/EtOAc 12:1→10:1) to give the fully protected **30** (21.6 g, 30.2 mmol, 85 %) as a light yellow oil. Allyl glycoside **30** had *R*_f_ 0.8 (Tol/EtOAc 10:1). ^1^H NMR (CDCl_3_) *δ* 7.84–7.30 (m, 22 H, H_Ar_), 5.98–5.88 (m, 1 H, CH_All_), 5.34–5.28 (m, 1 H, CH_2All_), 5.21–5.17 (m, 1 H, CH_2All_), 4.79 (d, 1 H, *J=*12.2 Hz, CH_2Nap_), 4.79 (d, 1 H, CH_2Nap_), 4.73 (d, 1 H, *J*_1,2_=4.7 Hz, H‐1), 4.67 (d, 1 H, *J=*11.9 Hz, CH_2Bn_), 4.61 (d, 1 H, CH_2Bn_), 4.31–4.25 (m, 1 H, CH_2All_), 4.19 (pq, 1 H, H‐5), 4.07–4.01 (m, 1 H, CH_2All_), 3.98–3.94 3.97 (dd_po_, 1 H, H‐2), 3.95 (dd_po_, 1 H, *J*_4,5_=5.3 Hz, H‐4), 3.77 (brd, 2 H, *J*_5,6a_=4.5 Hz, *J*_5,6b_=4.5 Hz, H‐6a, H‐6b), 3.74 (dd_po_, 1 H, *J*_3,4_=3.5 Hz, *J*_2,3_=8.0 Hz, H‐3), 1.00 (s, 9 H, CH_3, TBDPS_). ^13^C NMR (CDCl_3_), *δ* 137.8, 135.5, 133.2, 133.0 (C_q, Ar_), 133.8 (CH_All_), 135.6, 135.6, 129.7, 128.3, 128.1, 127.9, 127.7 (2C), 126.6 (2C), 126.0, 125.9, 125.8 (C_Ar_), 117.2 (CH_2All_), 98.7 (C‐1, ^1^ *J*_C,H_=170 Hz), 76.2 (C‐3), 72.9 (C‐5), 72.3 (CH_2Nap_, CH_2Bn_), 72.1 (C‐4), 68.7 (CH_2All_), 63.7 (C‐6), 61.8 (C‐2), 26.9, 26.7 (3C, CH_3,TBDPS_), 19.1 (C_TBDPS_). HRMS (ESI^+^): *m*/*z* [M+Na]^+^ calcd for C_34_H_47_N_3_O_5_SiNa 736.3783; found 736.3777.

**Allyl 3‐*O*‐benzyl‐6‐*O*‐*tert*‐butyldiphenylsilyl‐2‐deoxy‐4‐*O*‐(2‐naphthylmethyl)‐2‐tetrachlorophthalimido‐α‐l‐altropyranoside (31)**: Zn dust (8.2 g, 126 mmol, 10.0 equiv) and AcOH (7.2 mL, 126 mmol, 10.0 equiv) were added to azide **30** (9.0 g, 12.6 mmol, 1.0 equiv) in anhyd. THF (85 mL). After stirring for 1 h, a TLC analysis (Tol/EtOAc 10:1) showed the absence of azide **30** (*R*_f_ 0.8) and the presence of a more polar spot. The suspension was filtered over a pad of Celite and washed with DCM. The DCM layer was washed with satd aq. NaHCO_3_, water, and brine, dried over Na_2_SO_4_, concentrated under reduced pressure, and dried under high vacuum. The crude amine was dissolved in DCM and tetrachlorophthalic anhydride (2.2 g, 7.5 mmol, 0.6 equiv) was added. The mixture was stirred at rt for 30 min. Et_3_N (2.1 mL, 15.1 mmol, 1.2 equiv) was added followed by more tetrachlorophthalic anhydride (2.2 g, 7.5 mmol, 0.6 equiv). The reaction was stirred for another 30 min at rt, at which time a TLC follow up (EtOAc) indicated reaction completion. Volatiles were evaporated and dried under high vacuum. The crude was dissolved in pyridine (60 mL) and Ac_2_O (5.9 mL, 63.0 mmol, 5.0 equiv) was added. After heating to 80 °C for 10 min, a TLC analysis (cHex/EtOAc 9:1) showed full consumption of the intermediate and the presence of a less polar spot. At completion, the mixture was concentrated under reduced pressure and coevaporated with toluene (30 mL) twice. The crude was taken in DCM (300 mL) and the DCM layer was washed with water (300 mL) and brine (300 mL), dried over Na_2_SO_4_, filtered, and concentrated under reduced pressure. The residue was purified by flash chromatography (cHex/EtOAc 93:7→88:12) to give the fully protected **31** (9.54 g, 10.4 mmol, 82 %) as a yellowish dense oil. Allyl glycoside 31 had *R*_f_ 0.7 (cHex/EtOAc, 10:1). ^1^H NMR (CDCl_3_) *δ* 7.83–7.35 (m, 18 H, H_Ar_), 7.04 (brs, 4 H, H_Ar_), 5.83–5.73 (m, 1 H, CH_All_), 5.27 (d, 1 H, *J*_1,2_=7.0 Hz, H‐1), 5.19–5.14 (m, 1 H, CH_2All_), 5.08–5.05 (m, 1 H, CH_2All_), 4.94 (d, 1 H, *J=*12.7 Hz, CH_2Nap_), 4.85 (d_po_, 1 H, CH_2Nap_), 4.85 (dd_po_, 1 H, *J*_2,3_=11.2 Hz, H‐2), 4.60 (d, 1 H, *J=*12.3 Hz, CH_2Bn_), 4.3.6 (dd, 1 H, *J*_3,4_=3.6 Hz, H‐3), 4.30 (ddd_po_, 1 H, *J*_4,5_=3.3 Hz, H‐5), 4.25–4.19 (m, 1 H, CH_2All_), 4.16 (pt, 1 H, H‐4), 4.10 (d, 1 H, CH_2Bn_), 3.98–3.93 (m, 1 H, CH_2All_), 3.83 (brd, 2 H, *J*_5,6a_=6.1 Hz, *J*_5,6b_=6.1 Hz, H‐6a, H‐6b), 1.04 (s, 9 H, CH_3,TBDPS_). ^13^C NMR (CDCl_3_) *δ* 163.2 (CO_NTCP_), 157.4, 142.4, 139.7, 138.0, 135.9, 133.2, 133.1, 133.0 (2C), 129.5, 127.7, 127.2 (C_q,Ar_), 133.9 (CH_All_), 135.6 (2C), 129.8, 128.0, 127.9, 127.8, 127.6, 127.5, 127.3, 126.5, 126.0 (2C), 125.8 (C_Ar_), 117.1 (CH_2All_), 96.1 (C‐1, ^1^ *J*_C,H_=169 Hz), 75.6 (C‐5), 74.3 (C‐3), 72.7 (CH_2Nap_), 72.1 (CH_2Bn_), 71.8 (C‐4), 68.6 (CH_2All_), 63.1 (C‐6), 53.4 (C‐2), 26.9, 26.7 (3C, CH_3,TBDPS_), 19.2 (C_TBDPS_). HRMS (ESI^+^): *m*/*z* [M+NH_4_]^+^ calcd for C_51_H_51_Cl_4_N_2_O_7_Si 971.2220; found 971.2213.

**3‐*O*‐Benzyl‐6‐*O*‐*tert*‐butyldiphenylsilyl‐2‐deoxy‐4‐*O*‐(2‐naphthylmethyl)‐2‐tetrachlorophthalimido‐α/β‐l‐altropyranose (32)**: Ir(COD)(PMePh_2_)_2_]PF_6_ (115 mg, 0.13 mmol, 0.02 equiv) was dissolved in anhyd. THF (8.0 mL) and stirred for 30 min under an H_2_ atmosphere. The resulting yellow solution was degassed repeatedly with Ar and transferred by means of a cannula into a solution of allyl glycoside **31** (6.5 g, 6.8 mmol, 1.0 equiv) in anhyd. THF (60 mL). The reaction mixture was stirred for 1 h at rt, at which time a solution of NIS (1.68 g, 7.5 mmol, 1.1 equiv) in H_2_O (15 mL) was added. After stirring for 1 h at rt, a TLC analysis (cHex/EtOAc 8:1) revealed the full consumption of the isomerization product (*R*_f_ 0.65) and the presence of a more polar spot (*R*_f_ 0.1). 10 % Aq. Na_2_SO_3_ was added and volatiles were evaporated. The aq. phase was extracted with DCM (200 mL) twice. The organic layers were combined, washed with water and brine, dried over Na_2_SO_4_, filtered, and concentrated under vacuum. Purification of the residue by flash chromatography (Tol/EtOAc 10:1→8:1) yielded the expected hemiacetal **32** (5.2 g, 5.6 mmol, 82 %) as a white floppy solid. Hemiacetal **32** (α/*β* 5:1) had *R*_f_ 0.6 (cHex/EtOAc 9:1). The α anomer had ^1^H NMR (CDCl_3_) *δ* 7.84–6.99 (m, 22 H, H_Ar_), 5.25 (dd, 1 H, *J*_1,OH_=9.4 Hz, *J*_1,2_=6.8 Hz, H‐1), 4.81 (d, 1 H, *J=*12.4 Hz, CH_2Nap_), 4.76 (d, 1 H, CH_2Nap_), 4.67 (dd, 1 H, *J*_2,3_=10.8 Hz, H‐2), 4.53 (d, 1 H, *J=*12.4 Hz, CH_2Bn_), 4.43–4.33 (dd, 1 H, *J*_3,4_=3.0 Hz, H‐3), 4.30 (ddd, *J*_4,5_=1.8 Hz, 1 H, H‐5), 4.19 (dd, 1 H, H‐4), 4.03 (d, 1 H, CH_2Bn_), 3.91 (dd, 1 H, *J*_5,6a_=5.6 Hz, *J*_6a,6b_=10.6 Hz, H‐6a), 3.86 (dd,1 H, *J*_6a,6b_=8.2 Hz, H‐6b), 3.06 (d, 1 H, OH), 1.03 (s, 9 H, CH_3TBDPS_). ^13^C NMR (CDCl_3_) *δ* 163.2 (CO_NTCP_), 139.8, 137.8, 137.9, 137.8, 135.6, 135.5, 135.4, 133.2, 133.1, 133.0, 132.8 (C_q,Ar_), 135.6, 135.5, 129.9 (2C), 129.0, 128.2 (2C), 128.1 (2C), 127.9, 127.8 (2C), 127.7, 127.4, 126.8, 126.6, 126.0 (2C), 125.9, 125.2 (C_q,Ar_), 91.5 (C‐1, ^1^ *J*_C,H_=171 Hz), 76.1 (C‐3), 73.3 (C‐5), 72.7 (CH_2Nap_), 71.7 (CH_2Bn_), 71.7 (C‐4), 62.2 (C‐6), 56.1 (C‐2), 26.8 (CH_3TBDPS_), 21.4 (C_TBDPS_). HRMS (ESI^+^): *m*/*z* [M+NH_4_]^+^ calcd for C_48_H_47_Cl_4_N_2_O_7_Si 931.1907; found 931.1880.

The β anomer had ^1^H NMR (CDCl_3_) *δ* 7.84–6.99 (m, 22 H, H_Ar_), 5.37 (dd, 1 H, *J*_1,2_=4.1 Hz, *J*_1,OH_=5.6 Hz, H‐1), 4.95 (dd, 1 H, *J*_2,3_=10.8 Hz, H‐2), 4.95 (dd_po_, 1 H, *J*_3,4_=2.7 Hz, H‐3), 4.90 (d_po_, 1 H, CH_2Nap_), 4.85 (d, 1 H, *J=*12.6 Hz, CH_2Nap_), 4.41 (d, 1 H, *J=*11.6 Hz, CH_2Bn_), 4.28 (bs_o_, 1 H, H‐4), 4.28–4.25 (m, 1 H, H‐5), 4.21 (d_po_, 1 H, CH_2Bn_), 3.99 (d_po_, 1 H, H‐6a), 3.93–3.84 (m_o_, 1 H, H‐6b), 3.48 (brs, 1 H, OH), 1.08 (s, 9 H, CH_3TBDPS_). ^13^C NMR (CDCl_3_) *δ* 163.9 (CO_NTCP_), 139.8–125.3 (C_Ar_), 92.6 (C‐1, ^1^ *J*_C,H_=175 Hz), 77.4 (C‐3), 72.2 (CH_2Nap_), 72.4 (C‐4), 71.4 (CH_2Bn_), 64.6 (C‐6), 53.6 (C‐2), 26.8 (CH_3TBDPS_), 21.4 (C_TBDPS_). HRMS (ESI^+^): *m*/*z* [M+NH_4_]^+^ calcd for C_48_H_47_Cl_4_N_2_O_7_Si 931.1907; found 931.1880.

**Allyl 3‐*O*‐benzyl‐6‐*O*‐*tert*‐butyldiphenylsilyl‐2‐deoxy‐4‐*O*‐(2‐napthylmethyl)‐2‐tetrachlorophthalimido‐α‐l‐altropyranosyl‐(1→3)‐4‐azido‐2‐trichloroacetamido‐2,4,6‐trideoxy‐β‐d‐galactopyranoside (34)**: PTFACl (1.47 mL, 7.1 mmol, 1.3 equiv) and Cs_2_CO_3_ (1.9 g, 6.0 mmol, 1.1 equiv) were added to hemiacetal **32** (5.0 g, 5.4 mmol, 1.0 equiv) in acetone (40 mL). After stirring for 2 h at rt, the reaction mixture was filtered through a pad of Celite and washed with DCM (50 mL) twice. The filtrate was concentrated under reduced pressure and dried under vacuum to give the crude donor **33** (6.0 g, 5.4 mmol, quant.), which was used as such in the next step. The PTFA donor **33** had *R*_f_ 0.85 (Tol/EtOAc 10:1). HRMS (ESI^+^): *m*/*z* [M+Na]^+^ calcd for C_56_H_47_Cl_4_F_3_N_2_O_7_SiNa 1107.1757; found 1107.1755.

A mix of the crude PTFA donor **33** (6.0 g, 5.4 mmol, 1.1 equiv theo.) and acceptor **8** (1.83 g, 4.9 mmol, 1.0 equiv) were co‐evaporated with anhyd. toluene (30 mL) and then dried under vacuum. Freshly activated MS 4 Å (4.0 g) was added to the starting materials in anhyd. DCM (90 mL) and the suspension was stirred for 1 h under an Ar atmosphere at rt. After cooling to −15 °C, TMSOTf (49 μL, 0.05 equiv) was added slowly and stirring went on for 40 min during which the bath temperature kept at −15 °C. A TLC analysis (Tol/EtOAc 10:1) showed the absence of donor **33** and the presence of a new spot (*R*_f_ 0.5) in addition to a slight amount of hemiacetal **32** (*R*_f_ 0.4). At completion, Et_3_N (80 μL) was added. The suspension was filtered through a fitted funnel and washed with DCM (50 mL) twice. Volatiles were evaporated and the residue was purified by flash chromatography (cHex/EtOAc 10:1→8:1) to give disaccharide **34** as a white solid (6.0 g, 4.7 mmol, 96 %). The coupling product **34** had ^1^H NMR (CDCl_3_) *δ* 7.85–7.81 (m, 4 H, H_Ar_), 7.67–7.63 (m, 3 H, H_Ar_), 7.53–7.16 (m, 10 H, H_Ar_), 7.04–6.98 (m, 5 H, H_Ar_), 6.67 (m, 1 H, *J*_2,NH_=6.8 Hz, NH_B_), 5.85–5.75 (m, 1 H, CH_All_), 5.43 (d, 1 H, *J*_1,2_=7.2 Hz, H‐1_A_), 5.23–5.17 (m, 1 H, CH_2All_), 5.14–5.11 (m, 1 H, CH_2All_), 4.94 (d, 1 H, *J=*12.4 Hz, CH_2Nap_), 4.87 (dd_po_, 1 H, *J*_2,3_=11.1 Hz, H‐2_A_), 4.81 (d, 1 H, CH_2Nap_), 4.73 (d, 1 H, *J*_1,2_=8.4 Hz, H‐1_B_), 4.61 (d, 1 H, *J=*12.0 Hz, CH_2Bn_), 4.50 (dd, 2 H, *J*_3,4_=3.5 Hz, *J*_2,3_=10.7 Hz, H‐3_B_), 4.39 (ddd_po_, 1 H, *J*_4,5_=3.3 Hz, H‐5_A_), 4.33 (dd_po_, 1 H, *J*_3,4_=3.5 Hz, H‐3_A_), 4.29–4.24 (m, 1 H, CH_2All_), 4.11 (pt_po_, 1 H, H‐4_A_), 4.09 (d_po_, 1 H, CH_2Bn_), 4.00–3.94 (m, 1 H, CH_2All_), 3.86 (d_po_, 1 H, *J*_3,4_=3.8 Hz, H‐4_B_), 3.85 (d_po_, 1 H, *J*_5,6a_=6.4 Hz, H‐6a_A_), 3.79 (dd, 1 H, *J*_5,6b_=5.8 Hz, *J*_6a,6b_=11.0 Hz, H‐6b_A_), 3.51 (dd, 1 H, H‐2_B_,), 3.47 (dq, *J*_4,5_=1.3 Hz, H‐5_B_), 1.82 (d, 3 H, *J*_5,6_=6.4 Hz, H‐6_B_), 1.05 (s, 9 H, CH_3TBDPS_). ^13^C NMR (CDCl_3_) *δ* 163.1 (CO_NHTCA_), 161.5 (CO_NTCP_), 139.8, 137.8, 137.7, 135.7, 133.2, 133.0 (2C), 132.9, 129.6, 127.5 (C_q, Ar_), 133.5 (CH_All_), 135.6, 135.2, 129.9, 129.0, 128.2, 128.1, 128.0, 127.9, 127.8, 127.7, 127.6, 127.4, 126.5, 126.0, 125.9, 125.2 (C_Ar_), 117.8 (CH_2All_), 98.5 (C‐1_A_, ^1^ *J*_C,H_=171 Hz), 97.6 (C‐1_B_, ^1^ *J*_C,H_=163 Hz), 92.2 (CCl_3_), 75.9 (C‐5_A_), 75.5 (C‐3_B_), 73.7 (C‐3_A_), 72.6 (CH_2Nap_), 72.1 (CH_2Bn_), 71.4 (C‐4_A_), 70.0 (CH_2All_), 69.2 (C‐5_B_), 65.4 (C‐4_B_), 63.1 (C‐6_A_), 53.3 (C‐2_B_), 53.1 (C‐2_A_), 26.9 (CH_3TBDPS_), 19.3 (C_TBDPS_), 17.2 (C‐6_B_). HRMS (ESI^+^): *m*/*z* [M+NH_4_]^+^ calcd for C_59_H_60_Cl_7_N_6_O_10_Si 1285.1960; found 1285.1948.

**Allyl 2‐acetamido‐3‐*O*‐benzyl‐6‐*O*‐*tert*‐butyldiphenylsilyl‐2‐deoxy‐4‐*O*‐(2‐naphthylmethyl)‐α‐l‐altropyranosyl‐(1→3)‐4‐azido‐2‐trichloroacetamido‐2,4,6‐trideoxy‐β‐d‐galactopyranoside (35)**: Ethylenediamine (1.3 mL, 19.3 mmol, 4.0 equiv) was added to disaccharide **34** (6.2 g, 4.8 mmol, 1.0 equiv) in THF/MeOH (1:1, 100 mL) at rt and the reaction mixture was stirred at 50 °C for 72 h under an Ar atmosphere. A TLC analysis (Tol/EtOAc 7:3) revealed the absence of the starting **34** (*R*_f_ 1.0) and the presence of a new spot (*R*_f_ 0.55). The mixture was allowed to reach rt and Et_3_N (2.0 mL) was added, followed by acetic anhydride (4.6 mL, 48.9 mmol, 10.0 equiv). After stirring for 3 h at rt, a TLC analysis (Tol/EtOAc 7:3) showed the presence of a new spot (*R*_f_ 0.65) whereas the intermediate amine had been fully consumed. The suspension was filtered by passing through a pad of Celite, washed with DCM (15 mL) thrice and the filtrate was concentrated under reduced pressure. The residue was purified by column chromatography (cHex/EtOAc 10:1→7:1). Acetamide **35** was obtained as a white solid (4.8 g, 4.6 mmol, 94 %). Disaccharide 35 had ^1^H NMR (CDCl_3_) *δ* 7.84–7.81 (m, 1 H, H_Ar_), 7.75–7.62 (m, 7 H, H_Ar_), 7.50–7.26 (m, 15 H, H_Ar_), 6.67 (d, 1 H, *J*_2,NH_=7.2 Hz, NH_B_), 5.90–5.80 (m, 1 H, CH_All_), 5.28–5.23 (m_po_, 1 H, CH_2All_), 5.23 (d_po_, 1 H, *J*_2,NH_=8.8 Hz, NH_A_), 5.19–5.16 (m, 1 H, CH_2All_), 4.89 (d, 1 H, *J*_1,2_=8.0 Hz, H‐1_B_), 4.78 (d, 1 H, *J=*12.7 Hz, CH_2Nap_), 4.74 (d, 1 H, CH_2Nap_), 4.71 (d_po_, 1 H, CH_2Bn_), 4.69 (brs_po_, 1 H, *J*_1,2_=1.8 Hz, H‐1_A_), 4.52 (d, 1 H, *J=*12.1 Hz, CH_2Bn_), 4.48 (dd_po_, 1 H, *J*_3,4_=3.7 Hz, *J*_2,3_=10.9 Hz, H‐3_B_), 4.47–4.43 (m_o_, 2 H, H‐5_A_), 4.41 (ddd, *J*_2,3_=4.3 Hz, H‐2_A_), 4.34–4.29 (m, 1 H, CH_2All_), 4.06–4.01 (m_po_, 1 H, CH_2All_), 4.00 (dd_po_, 1 H, *J*_5,6_ *a*_=_2.6 Hz, *J*_6_ a_,6_ *b*_=_11.2 Hz, H‐6 a_A_), 3.95 (dd_po_, 1 H, *J*_5,6_ *a*_=_2.6 Hz, *J*_6_ a_,6_ *b*_=_11.1 Hz, H‐6 b_A_), 3.93 (pt_po_, 1 H, H‐3_A_), 3.63 (dd, 1 H, *J*_3,4_=3.0 Hz, *J*_4,5_=8.9 Hz, H‐4_A_), 3.57 (brd, 1 H, H‐4_B_), 3.53 (brq, 1 H, H‐5_B_), 3.41 (ddd, 1 H, H‐2_B_), 1.74 (s, 3 H, CH_3NHAc_), 1.17 (d, 3 H, *J*_5,6_=6.3 Hz, H‐6_B_), 1.08 (s, 9 H, CH_3TBDPS_). ^13^C NMR (CDCl_3_) *δ* 168.8 (CO_NHTA_), 162.1 (CO_NHAc_), 138.6, 135.1, 133.5, 133.0 (2C, C_q,Ar_), 133.6 (CH_All_), 135.7, 135.6, 129.7 (2C), 128.2 (2C), 127.8, 127.7, 127.6, 127.5, 126.8, 126.1, 125.9 (2C, C_Ar_), 117.7 (CH_2All_), 101.5 (C‐1_A_, ^1^ *J*_C,H_=168 Hz), 97.5 (C‐1_B_, ^1^ *J*_C,H_=163 Hz), 92.2 (CCl_3_), 76.0 (C‐3_B_), 72.5 (C‐3_A_), 71.5 (CH_2Nap_), 70.5 (CH_2Bn_), 70.4 (C‐4_A_), 70.1 (CH_2All_), 69.7 (C‐5_A_), 69.7 (C‐5_B_), 65.2 (C‐4_B_), 63.7 (C‐6_A_), 55.8 (C‐2_B_), 49.6 (C‐2_A_), 27.0 (CH_3TBDPS_), 23.0 (CH_3NHAc_), 19.4 (C_TBDPS_), 17.1 (C‐6_B_). HRMS (ESI^+^): *m*/*z* [*M*+H]^+^ calcd for C_53_H_61_Cl_3_N_5_O_9_Si 1044.3304; found 1044.3325.

**Allyl 2‐acetamido‐3‐*O*‐benzyl‐2‐deoxy‐4‐*O*‐(2‐naphthylmethyl)‐α‐l‐altropyranosyl‐(1→3)‐4‐azido‐2‐trichloroacetamido‐2,4,6‐trideoxy‐β‐d‐galactopyranoside (36)**: TBAF (1.8 g, 5.8 mmol, 1.2 equiv) was added to disaccharide **35** (4.8 g, 4.8 mmol, 1.0 equiv) in THF (98 mL) and the reaction mixture was stirred at rt for 4 h. A TLC analysis (Tol/EtOAc 7:3) showed the consumption of the fully protected **35** (*R*_f_ 0.65) and the presence of a polar spot. Acetic acid (0.34 mL, 5.8 mmol, 1.2 equiv) was added and after stirring for 10 min, volatiles were evaporated. The residue was purified by flash chromatography (EtOAc/MeOH 100:0→95:5) to give alcohol **33** (3.2 g, 3.9 mmol, 86 %) as a white solid. Disaccharide **36** had *R*_f_ 0.15 (EtOAc). ^1^H NMR ([D_6_]DMSO) *δ* 8.87 (d, 1 H, *J*_2,NH_=9.2 Hz, NH_B_), 7.93–7.82 (m, 4 H, NH_A_, H_Ar_), 7.75 (brs, 1 H, H_Ar_), 7.53–7.47 (m, 2 H, H_Ar_), 7.43–7.39 (m, 3 H, H_Ar_), 7.32–7.24 (m, 3 H, H_Ar_), 5.85–5.76 (m, 1 H, CH_All_), 5.26–5.20 (m, 1 H, CH_2All_), 5.12–5.09 (m, 1 H, CH_2All_), 4.81 (d, 1 H, *J*_1,2_=1.6 Hz, H‐1_A_), 4.69 (d_po_, 3 H, *J=*11.6 Hz, CH_2Nap_), 4.67–4.62 (m, 2 H, *J=*11.8 Hz, OH, CH_2Bn_), 4.53 (d_po_, 1 H, CH_2Bn_), 4.51 (d_po_, 1 H, *J*_1,2_=8.9 Hz, H‐1_B_), 4.49 (d_po_, 1 H, CH_2Nap_), 4.32 (ddd, 1 H, *J*_2,NH_=8.4 Hz, *J*_2,3_=4.5 Hz, H‐2_A_), 4.24–4.16 (m, 2 H, H‐5_A_, CH_2All_), 4.12 (dd_po_, 1 H, *J*_3,4_=3.6 Hz, *J*_2,3_=10.8 Hz, H‐3_B_), 4.05 (brd, 1 H, H‐4_B_), 4.00–3.95 (m, 1 H, CH_2All_), 3.90 (ddd_po_, 1 H, H‐2_B_), 3.79 (dd_po_, 1 H, H‐3_A_), 3.78–3.74 (m_o_, 1 H, H‐6 a_A_), 3.72 (bq, 1 H, H‐5_B_), 3.68 (dd, 1 H, *J*_3,4_=3.1 Hz, *J*_4,5_=8.9 Hz, H‐4_A_), 3.53 (ddd, 1 H, H‐6 b_B_), 1.74 (s, 3 H, CH_3NHAc_), 1.24 (s, 9 H, CH_3TBDPS_). ^13^C NMR ([D_6_]DMSO) *δ* 169.1 (CO_NHTCA_), 162.0 (CO_NHAc_), 139.3, 136.4, 133.1, 132.9 (C_q,Ar_), 134.8 (CH_All_), 128.3, 128.1 (2C), 128.0, 127.6, 126.5, 126.4, 126.3 (C_Ar_), 116.8 (CH_2All_), 101.9 (C‐1_A_, ^1^ *J*_C,H_=171 Hz), 100.2 (C‐1_B_, ^1^ *J*_C,H_=162 Hz), 93.5 (CCl_3_), 77.5 (C‐3_B_), 73.8 (C‐3_A_), 72.5 (C‐4_A_), 70.9 (CH_2Nap_), 70.4 (CH_2Bn_), 70.2 (C‐5_B_), 69.6 (C‐5_A_), 69.3 (CH_2All_), 65.3 (C‐4_B_), 61.6 (C‐6_A_), 53.4 (C‐2_B_), 49.4 (C‐2_A_), 22.9 (CH_3NHAc_), 17.6 (C‐6_B_). HRMS (ESI^+^): *m*/*z* [*M*+H]^+^ calcd for C_37_H_43_Cl_3_N_5_O_9_ 806.2126; found 806.2117.

**Allyl (benzyl 2‐acetamido‐3‐*O*‐benzyl‐2‐deoxy‐4‐*O*‐(2‐naphthylmethyl)‐α‐l‐altropyranosyluronate)‐(1→3)‐4‐azido‐2‐trichloroacetamido‐2,4,6‐trideoxy‐β‐d‐galactopyranoside (37)**: TEMPO (116 mg, 0.74 mmol, 0.2 equiv) was added, followed by BAIB (3.0 g, 9.3 mmol, 2.5 equiv), to a suspension of alcohol **36** (3.0 g, 3.7 mmol, 1.0 equiv) in DCM/H_2_O (2:1, 120 mL). The biphasic mixture stirred vigorously for 2 h at rt, at which point a TLC analysis (EtOAc) revealed the absence of alcohol **36** (*R*_f_ 0.15) and the presence of a polar product (*R*_f_ 0.0). 10 % Aq. Na_2_SO_3_ was added followed by DCM (80 mL). The DCM layer was separated, and the aq. phase was extracted with DCM (100 mL) twice. The combined organic phases were dried by passing through a phase separator filter and concentrated to dryness. The residue was dissolved in anhyd. DMF (40 mL). Benzyl bromide (1.3 mL, 11.1 mmol, 3.0 equiv) and K_2_CO_3_ (670 mg, 4.8 mmol, 1.3 equiv) were added and the suspension was stirred at rt for 2 h. At completion, satd aq. NH_4_Cl was added and the aq. layer was washed with DCM (100 mL) thrice. The organic phases were combined, washed with brine (300 mL), dried over Na_2_SO_4_, filtered, and concentrated under reduced pressure. The residue was purified by flash chromatography (Tol/EtOAc 7:3→6:4) to give the desired benzyl ester **37** (2.8 g, 3.0 mmol, 85 %) as a brown‐white solid. Ester **37** had *R*_f_ 0.3 (Tol/EtOAc, 4:1). ^1^H NMR (CDCl_3_) *δ* 7.84–7.26 (m, 17 H, H_Ar_), 6.94 (d, *J*_2,NH_=7.2 Hz, NH_B_), 5.90–5.80 (m, 1 H, CH_All_), 5.73 (d, *J*_2,NH_=6.8 Hz, NH_A_), 5.33 (d, 1 H, *J*_1,2_=5.6 Hz, H‐1_A_), 5.24–5.20 (m_po_, 1 H, CH_2All_), 5.22 (d_po_, 1 H, CH_2Bn‐6_), 5.19–5.15 (m_po_, 1 H, CH_2All_), 5.17 (d, 1 H, *J=*12.0 Hz, CH_2Bn‐6_), 4.80 (d, 1 H, *J*_4,5_=4.6 Hz, H‐5_A_), 4.77 (d, 1 H, *J*_1,2_=8.3 Hz, H‐1_B_), 4.73 (d, 1 H, *J=*12.7 Hz, CH_2Nap_), 4.71 (d, 1 H, CH_2Nap_), 4.52 (d, 1 H, *J=*12.0 Hz, CH_2Bn_), 4.48 (dd_po_, 1 H, *J*_3,4_=3.8 Hz, H‐3_B_), 4.48 (d, 1 H, CH_2Bn_), 4.33–4.28 (m, 1 H, CH_2All_), 4.10 (dd_po_, *J*_3,4_=2.7 Hz, H‐4_A_), 4.07–3.98 (m, 3 H, H‐3_A_, H‐2_A_, CH_2All_), 3.93 (brd, 1 H, *J*_3,4_=3.2 Hz, H‐4_B_), 3.57 (d_t_, 1 H, *J*_2,3_=10.5 Hz, H‐2_B_), 3.47 (brq, 1 H, H‐5_B_), 1.86 (s, 3 H, CH_3Ac_), 1.23 (d, 3 H, *J*_5,6_=6.3 Hz, H‐6_B_). ^13^C NMR (CDCl_3_) *δ* 170.4 (CO_NTCA_), 169.3 (C‐6_A_), 161.9 (CO_NAc_), 137.9, 134.9, 134.8, 133.1, 133.0 (C_q, Ar_), 133.5 (CH_All_), 128.7 (2C), 128.6, 128.3, 128.1, 127.9, 127.8, 127.6, 126.9, 126.1, 126.0, 125.9 (C_Ar_), 117.9 (CH_2All_), 99.5 (C‐1_A_, ^1^ *J*_C,H_=169 Hz), 97.6 (C‐1_B_, ^1^ *J*_C,H_=162 Hz), 92.4 (CCl_3_), 76.6 (C‐3_B_), 73.4 (C‐3_A_), 72.9 (C‐4_A_), 71.9 (C‐5_A_), 71.8 (2C, CH_2Bn,_ CH_2Nap_), 70.1 (CH_2All_), 69.3 (C‐5_B_), 67.5 (CH_2Bn‐6_), 65.1 (C‐4_B_), 55.0 (C‐2_B_), 52.1 (C‐2_A_), 23.4 (CH_3Ac_), 17.3 (C‐6_B_). HRMS (ESI^+^): *m*/*z* [*M*+H]^+^ calcd for C_44_H_47_Cl_3_N_5_O_10_ 910.2388; found 910.2380.

**Allyl (benzyl 3‐*O*‐benzyl‐2‐deoxy‐2‐(*N***,***N*** **‐diacetyl)amino‐4‐*O*‐(2‐naphthylmethyl)‐α‐l‐altropyranosyluronate)‐(1→3)‐4‐azido‐2‐trichloroacetamido‐2,4,6‐trideoxy‐β‐d‐galactopyranoside (47)**: DIPEA (9.5 mL, 54.9 mmol, 20.0 equiv) and acetyl chloride (3.9 mL, 21.9 mmol, 20 equiv) were added successively to a solution of disaccharide **37** (2.5 g, 2.75 mmol, 1.0 equiv) in anhyd. DCM (90 mL) at 0 °C. The mixture was allowed to reach rt slowly and was stirred overnight. A TLC follow up (Tol/EtOAc 4:1) showed the complete conversion of acetamido **37** (*R*_f_ 0.25) to a less polar product (*R*_f_ 0.8). 10 % Aq. NaHCO_3_ (50 mL) was added and the biphasic mixture was diluted with DCM (20 mL). The organic layer was separated, dried over Na_2_SO_4_, and concentrated under reduced pressure. The residue was purified by flash chromatography (Tol/EtOAc 90:20→88:12) to give the fully protected **47** (2.36 g, 2.48 mmol, 90 %) as an off‐white solid. The *N*‐acetylacetamido derivative **47** had *R*_f_ 0.65 (Tol/EtOAc 7:3). ^1^H NMR (CDCl_3_) *δ* 7.85–7.83 (m, 3 H, H_Ar_), 7.52–7.45 (m, 3 H, H_Ar_), 7.42–7.36 (m, 4 H, H_Ar_), 7.30–7.26 (m, 4 H, H_Ar_), 7.21–7.12 (m, 3 H, H_Ar_), 6.75 (d, *J*_2,NH_=7.6 Hz, NH_B_), 5.92–5.82 (m, 1 H, CH_All_), 5.80 (d, 1 H, *J*_1,2_=7.8 Hz, H‐1_A_), 5.29–5.23 (m, 1 H, CH_2All_), 5.26 (d, H, *J=*11.9 Hz, CH_2Bn‐6_), 5.21 (d, H, CH_2Bn‐6_), 5.20–5.17 (m, 1 H, CH_2All_), 4.85 (d, 1 H, *J=*12.9 Hz, CH_2Nap_), 4.81 (d, 1 H, CH_2Nap_), 4.76 (d, 1 H, *J*_1,2_=8.3 Hz, H‐1_B_), 4.73 (d, 1 H, *J*_4,5_=2.2 Hz, H‐5_A_), 4.48 (dd, 1 H, *J*_2,3_=10.8 Hz, *J*_3,4_=3.8 Hz, H‐3_B_), 4.39–4.34 (m_po_, 3 H, H‐4_A_, CH_2Bn_), 4.35–4.30 (m_po_, 1 H, CH_2All_), 4.27 (dd_po_, 1 H, *J*_2,3_=10.5 Hz, *J*_3,4_=2.9 Hz, H‐3_A_), 4.26 (d_po_, 1 H, *J=*11.6 Hz, CH_2Bn_), 4.06–4.00 (m_po_, 1 H, CH_2All_), 4.05 (bd_o_, 1 H, H‐4_B_), 3.59 (ddd, 1 H, H‐2_B_), 3.45 (dq, 1 H, *J*_5,6_=1.0 Hz, H‐5_B_), 2.38 (s, 6 H, CH_3NAc_), 1.27 (d, 3 H, *J*_5,6_=6.3 Hz, H‐6_B_). ^13^C NMR (CDCl_3_) *δ* 175.1 (2C, CO_NAc_), 168.7 (C‐6_A_), 161.9 (CO_NTCA_), 137.8, 137.4, 135.2, 134.9, 133.2, 133.0 (C_q,Ar_), 133.5 (CH_All_), 128.7–125.2 (C_Ar_), 117.9 (CH_2All_), 98.8 (C‐1_A_, ^1^ *J*_C,H_=176 Hz), 97.6 (C‐1_B_, ^1^ *J*_C,H_=162 Hz), 92.3 (CCl_3_), 77.4 (C‐3_B_), 73.9 (C‐5_A_), 73.7 (C‐3_A_), 73.0 (C‐4_A_), 72.5 (CH_2Nap_), 71.9 (CH_2Bn_), 70.2 (CH_2All_), 68.7 (C‐5_B_), 62.6 (CH_2Bn‐6_), 65.2 (C‐4_B_), 59.1 (C‐2_A_), 55.2 (C‐2_B_), 21.4 (CH_3Ac_), 17.4 (C‐6_B_). HRMS (ESI^+^): *m*/*z* [M+NH_4_]^+^ calcd for C_46_H_52_Cl_3_N_6_O_11_ 969.2759; found 969.2751.

**Allyl (benzyl 3‐*O*‐benzyl‐2‐(*N***,***N*** **‐diacetyl)amino‐2‐deoxy‐α‐l‐altropyranosyluronate)‐(1→3)‐4‐azido‐2‐trichloroacetamido‐2,4,6‐trideoxy‐β‐d‐galactopyranoside (48)**: Disaccharide **47** (1.0 g, 1.0 mmol, 1.0 equiv) was dissolved in DCM (10 mL) and phosphate buffer pH 7 (1.0 mL) was added. The biphasic mixture was cooled to 0 °C and DDQ (477 mg, 2.1 mmol, 2.0 equiv) was added. The reaction was slowly allowed to reach rt and stirred for 3 h at this temperature. At completion, 5 % aq. NaHCO_3_ (30 mL) was added and the biphasic mixture was diluted with DCM (50 mL). The DCM layer was separated, washed with brine (100 mL), dried over Na_2_SO_4_, filtered, and concentrated under reduced pressure. The residue was purified by flash chromatography (Tol/EtOAc 5:1→4:1) to give alcohol **48** (800 mg, 0.93 mmol, 93 %) as a white solid. Disaccharide **48** had *R*_f_ 0.45 (Tol/EtOAc, 7:3). ^1^H NMR (CDCl_3_) *δ* 7.45–7.39 (m, 5 H, H_Ar_), 7.35–7.25 (m, 3 H, H_Ar_), 7.20–7.17 (m, 2 H, H_Ar_), 6.69 (d, *J*_2,NH_=7.4 Hz, NH_B_), 5.90–5.80 (m, 1 H, CH_All_), 5.68 (d, 1 H, *J*_1,2_=7.9 Hz, H‐1_A_), 5.27 (s, 2 H, CH_2Bn‐6_), 5.28–5.22 (m, 1 H, CH_2All_), 5.19–5.15 (m, 1 H, CH_2All_), 4.73 (d, 1 H, *J*_4,5_=2.3 Hz, H‐5_A_), 4.67 (d, 1 H, *J*_1,2_=8.3 Hz, H‐1_B_), 4.53–4.52 (m_po_, 1 H, H‐4_A_), 4.50 (d_po_, 1 H, CH_2Bn_), 4.45 (dd_po_, 1 H, *J*_2,3_=10.7 Hz, *J*_3,4_=3.8 Hz, H‐3_B_), 4.24 (d_po_, *J=*11.6 Hz, CH_2Bn_), 4.36 (dd, 1 H, *J*_2,3_=10.3 Hz, *J*_3,4_=3.4 Hz, H‐3_A_), 4.33–4.28 (m, 1 H, CH_All_), 4.07–3.99 (m, 3 H, H‐2_A_, H‐4_B_, CH_2All_), 3.60 (pdt, 1 H, H‐2_B_), 3.39 (dq_po_, 1 H, *J*_4,5_=1.1 Hz, H‐5_B_), 2.57 (d, 1 H, *J*_4,OH_=2.0 Hz, OH), 2.37, 2.34 (2 s, 6 H, CH_3NAc_), 1.24 (d, 3 H, *J*_5,6_=6.3 Hz, H‐6_B_). ^13^C NMR (CDCl_3_) *δ* 174.9 (2C, CO_NAc_), 168.3 (C‐6_A_), 161.9 (CO_NTCA_), 136.8, 134.9 (C_q,Ar_), 133.5 (CH_All_), 129.0, 128.9, 128.8, 128.6, 128.3, 128.2, 128.0 (C_Ar_), 118.2 (CH_2All_), 98.3 (C‐1_A_, ^1^ *J*_C,H_=174 Hz), 97.7 (C‐1_B_, ^1^ *J*_C,H_=162 Hz), 92.3 (CCl_3_), 72.2 (C‐3_B_), 75.2 (C‐5_A_), 72.7 (C‐3_A_), 72.3 (CH_2Bn_), 70.1 (CH_2All_), 68.7 (C‐5_B_), 67.8 (CH_2Bn‐6_), 66.6 (C‐4_A_), 65.2 (C‐4_B_), 58.9 (C‐2_A_), 55.0 (C‐2_B_), 21.4 (CH_3Ac_), 17.3 (C‐6_B_). HRMS (ESI^+^): *m*/*z* [M+NH_4_]^+^ calcd for C_35_H_44_Cl_3_N_6_O_11_ 829.2134; found 829.2128.

**(Benzyl 3‐*O*‐benzyl‐2‐(*N***,***N*** **‐diacetyl)amino‐4‐*O*‐(2‐naphthylmethyl)‐2‐deoxy‐α‐l‐altropyranosyluronate)‐(1→3)‐4‐azido‐2‐trichloroacetamido‐2,4,6‐trideoxy‐α/β‐d‐galactopyranose (49)**: [Ir(COD)(PMePh_2_)_2_]PF_6_ (18 mg, 0.02 mmol, 0.02 equiv) in anhyd. THF (3.0 mL) was degassed and stirred for 20 min under an H_2_ atmosphere. The resulting yellow solution was degassed repeatedly with Ar and poured into a solution of allyl glycoside **47** (1.0 g, 1.05 mmol, 1.0 equiv) in anhyd. THF (20 mL). After stirring for 1 h at rt, a TCL follow up (cHex/EtOAc 10:1, 2 runs) revealed that the starting 47 (*R*_f_ 0.6) had been converted to a closely migrating product (*R*_f_ 0.65). NIS (260 mg, 1.1 mmol, 1.1 equiv) and H_2_O (12 mL) were added and after stirring for another 1 h at rt, 10 % aq. Na_2_SO_3_ was added. The reaction mixture was concentrated and the aq. phase was extracted with DCM (30 mL) thrice. The combined organic layers were washed with brine (50 mL), dried over anhyd. Na_2_SO_4_, and concentrated under reduced pressure. The residue was purified by flash chromatography with cHex/EtOAc (80:20→75:25) to give the expected hemiacetal **49** (870 mg, 0.95 mmol, 90 %) as a white solid. The α/β hemiacetal **49** had *R*_f_ 0.4, 0.45 (Tol/EtOAc, 4:1). The α isomer had ^1^H NMR (CDCl_3_) *δ* 7.85–7.75 (m, 4 H, H_Ar_), 7.52–7.46 (m, 3 H, H_Ar_), 7.39–7.26 (m, 8 H, H_Ar_), 7.21–7.10 (m, 2 H, H_Ar_), 6.70 (d, *J*_2,NH_=9.2 Hz, NH_B_), 5.80 (d, 1 H, *J*_1,2_=7.6 Hz, H‐1_A_), 5.24 (t, 1 H, *J*_1,2_=3.6 Hz, H‐1_B_), 5.19 (brs, 2 H, CH_2Bn‐6_), 4.83 (brs, 2 H, CH_2Nap_), 4.74 (d, 1 H, *J*_4,5_=2.0 Hz, H‐5_A_), 4.42–4.33 (m, 3 H, H‐2_A_, H‐4_A_, H‐2_B_), 4.34 (d_po_, 1 H, *J=*12.0 Hz, CH_2Bn_), 4.25 (d_po_, 1 H, *J=*12.0 Hz, CH_2Bn_), 4.23 (dd_po_, *J*_3,4_=3.2 Hz, *J*_2,3_=10.4 Hz, H‐3_A_), 4.15 (dd_po_, *J*_3,4_=2.4 Hz, H‐4_B_), 4.09–4.03 (m, 2 H, H‐3_B_, H‐5_B_), 3.15 (d, 1 H, *J*_1,OH_=3.6 Hz, OH), 2.39 (s, 3 H, CH_3Ac_), 2.38 (s, 3 H, CH_3Ac_), 1.21 (d, 3 H, *J*_5,6_=6.4 Hz, H‐6_B_). ^13^C NMR (CDCl_3_), *δ* 175.1 (2C, CO_NAc_), 168.8 (C‐6_A_), 161.9 (CO_NTCA_), 135.1, 134.8, 133.2, 133.0 (C_q,Ar_), 129.0, 128.8, 128.5, 128.4, 128.2, 127.9, 127.7 (2C), 126.4, 126.1, 125.6, 125.3 (C_Ar_), 98.8 (C‐1_A_, ^1^ *J*_C,H_=175 Hz), 92.4 (CCl_3_), 91.2 (C‐1_B_, ^1^ *J*_C,H_=176 Hz), 76.9 (C‐3_B_), 73.9 (C‐5_A_), 73.6 (C‐4_A_), 73.0 (C‐3_A_), 72.6 (CH_2Nap_), 71.8 (CH_2Bn_), 67.5 (CH_2Bn‐6_), 65.5 (C‐4_B_), 64.7 (C‐5_B_), 59.1 (C‐2_A_), 50.6 (C‐2_B_), 21.4 (CH_3Ac_), 17.3 (C‐6_B_). HRMS (ESI^+^): *m*/*z* [M+NH_4_]^+^ calcd for C_35_H_44_Cl_3_N_6_O_11_ 1615.3589; found 1615.3596.

**(Benzyl 3‐*O*‐benzyl‐2‐(*N***,***N*** **‐diacetyl)amino‐4‐*O*‐(2‐naphthylmethyl)‐2‐deoxy‐α‐l‐altropyranosyluronate)‐(1→3)‐4‐azido‐2‐trichloroacetamido‐2,4,6‐trideoxy‐α/β‐d‐galactopyranosyl (*N*‐phenyl)trifluoroacetimidate (50) and 2‐Trichloromethyl‐[(Benzyl 3‐*O*‐benzyl‐4‐*O*‐(2‐naphthylmethyl)‐2‐deoxy‐2‐(*N***,***N*** **‐diacetyl)amino‐α‐l‐altropyranosyluronate)‐(1→3)‐4‐azido‐1,2,4,6‐tetradeoxy‐α‐d‐galactopyrano]‐[2,1,d]‐oxazoline (51)**: Hemiacetal **49** was dissolved in acetone (12 mL) and PTFACl (113 μL, 713 μmol, 1.3 equiv) was added followed by Cs_2_CO_3_ (197 mg, 604 μmol, 1.1 equiv). After stirring at rt for 2 h, a TLC follow up (Tol/EtOAc 4:1) showed the complete conversion of the hemiacetal (*R*_f_ 0.4) into a less polar compound (*R*_f_ 0.9). The suspension was filtered over a pad of Celite, washed with acetone (5 mL) twice, and the filtrate was concentrated. The residue was purified by column chromatography (cHex/EtOAc 90:10→85:15, +1 % Et_3_N) to give a 4:1 mix of the expected PTFA donor **50** and oxazoline **51** (480 mg, 281 μmol, 80 %) as a white solid. The isolated mix of **50** and **51** had *R*_f_ 0.9 (Tol/EtOAc 4:1). ^1^H NMR (major compound, CDCl_3_) *δ* 7.85–6.70 (m, 21 H, H_Ar_), 6.59 (d, 1 H, *J=*8.4 Hz, NH), 5.94 (bs, 1 H, H‐1_B_), 6.37 (d, 1 H, *J*_1,2_=8.0 Hz, H‐1_A_), 5.21 (bs, 2 H, CH_2Bn‐6_), 6.37 (d, 1 H, CH_2Nap_), 6.37 (d, 1 H, *J=*12.1 Hz, CH_2Nap_), 4.88–4.81 (m_po_, 2.5 H), 4.75 (d, 1 H, *J=*2.0 Hz, H‐5_A_), 4.55 (ddd, 1 H, H‐2_B_), 4.41 (dd_po_, 1 H, *J*_2,3_=10.5 Hz, H‐2_A_), 4.39–4.33 (m, 2 H, H‐4_A_, CH_2Bn_), 4.25 (d, 1 H, *J=*11.8 Hz, CH_2Bn_), 4.21 (dd_po_, 1 H, *J*_2,3_=2.8 Hz, H‐3_A_), 4.19 (d_o_, 1 H, H‐4_B_), 4.13 (d, 1 H, *J*_2,3_=11.0 Hz, *J*_3,4_=3.2 Hz, H‐3_B_), 3.90 (brq, 1 H, H‐5_B_), 2.38 (s, 6 H, CH_3Ac_), 1.25 (d, 3 H, *J*_5,6_=6.2 Hz, H‐6_B_). ^13^C NMR (major isomer, CDCl_3_) *δ* 175.0 (CO_NAc_), 168.8, 168.7 (C‐6_A_), 162.0 (CO_NTCA_), 142.9, 137.2, 135.0, 134.7, 133.2, 133.0 (C_q,Ar_), 128.9, 128.8, 128.7 (2C), 128.6, 128.4 (2C), 128.2 (2C), 128.0, 127.8, 127.7 (2C), 126.5, 126.4, 126.3, 126.2, 126.1, 126.0 (2C), 125.7, 124.9, 120.4, 119.2 (C_Ar_), 98.5 (C‐1_A_), 93.7 (br, C‐1_B_), 92.0 (CCl_3_), 76.3 (C‐3_B_), 74.1 (C‐5_A_), 73.5 (C‐4_A_), 73.0 (C‐3_A_), 72.6 (CH_2Nap_), 71.8 (CH_2Bn_), 67.6 (CH_2Bn‐6_), 67.5 (C‐5_B_), 64.6 (C‐4_B_), 59.0 (C‐2_A_), 49.9 (C‐2_B_), 29.6 (CH_3Ac_), 17.3 (C‐6_B_). HRMS (ESI^+^): *m*/*z* [M+NH_4_]^+^ calcd for C_51_H_48_Cl_3_F_3_N_6_O_11_ 1100.2737; found 1100.2729.

**Allyl (benzyl 3‐*O*‐benzyl‐2‐(*N***,***N*** **‐diacetyl)amino‐2‐deoxy‐4‐*O*‐(2‐naphthylmethyl)‐α‐l‐altropyranosyluronate)‐(1→3)‐(4‐azido‐2‐trichloroacetamido‐2,4,6‐trideoxy‐β‐d‐galactopyranosyl)‐(1→3)‐(benzyl 3‐*O*‐benzyl‐2‐(*N***,***N*** **‐diacetyl)amino‐2‐deoxy‐α‐l‐altropyranosyluronate)‐(1→3)‐4‐azido‐2‐trichloroacetamido‐2,4,6‐trideoxy‐β‐d‐galactopyranoside (52)**: PTFACl (102 μL, 642 μmol, 1.3 equiv) and Cs_2_CO_3_ (117 mg, 543 μmol, 1.1 equiv) were added to a solution of hemiacetal **49** (230 mg, 252 μmol, 1.0 equiv) in acetone (8.0 mL). After stirring for 2 h at rt, the suspension was filtered over a pad of Celite and solids were washed with acetone (5 mL) thrice. The filtrate was concentrated under reduced pressure and the crude product was subjected to the next step.

The crude mix of glycosyl donors **50** and **51** (252 μmol theo., 1.1 equiv) and acceptor **48** (184 mg, 227 μmol, 1.0 equiv) were co‐evaporated with anhyd. toluene (5 mL) and then dried under high vacuum for 1 h. The dried mixture was dissolved in anhyd. DCM (8.0 mL) and stirred for 1 h with freshly activated MS 4 Å (500 mg) under an Ar atmosphere. The reaction mixture was cooled to 0 °C and TfOH (1.1 μL, 13 μmol, 0.05 equiv) was added. After stirring for 30 min at this temperature, a TLC analysis (Tol/EtOAc, 4:1) showed no further evolution while donor **50**/**51** (*R*_f_ 0.9) had reacted and a more polar spot (*R*_f_ 0.35) was visible. Et_3_N (2.0 μL) was added and the suspension was filtered over a fitted funnel. Solids were washed with DCM (5 mL) twice and the filtrate was concentrated under reduced pressure. The residue was purified by flash chromatography (Tol/EtOAc 80:20→60:40) to give firstly the condensation product **52** (240 mg, 141 μmol, 62 %; corr. yield 85 %) as a white solid and then some unreacted **48** (50 mg, 7 %). Tetrasaccharide **52** had ^1^H NMR (CDCl_3_) *δ* 7.82–7.74 (m, 4 H, H_Ar_), 7.51–7.10 (m, 23 H, H_Ar_), 6.99 (d, *J*_2,NH_=6.8 Hz, NH_B1_), 6.74 (d, *J*_2,NH_=7.2 Hz, NH_B_), 5.90 (m, 1 H, CH_All_), 5.78 (d, 1 H, *J*_1,2_=8.0 Hz, H‐1_A1_), 5.65 (d, 1 H, *J*_1,2_=8.0 Hz, H‐1_A_), 5.29–5.15 (m, 6 H, CH_2All_, CH_2Bn‐6_), 5.00 (d, 1 H, *J*_1,2_=8.0 Hz, H‐1_B1_), 4.85–4.79 (m, 3 H, CH_2Nap_, H‐5_A1_), 4.76 (d, 1 H, *J*_1,2_=8.0 Hz, H‐1_B_), 4.72 (d, 1 H, *J*_4,5_=2.4 Hz, H‐5_A_), 4.64 (dd, 1 H, *J*_3,4_=4.0 Hz, *J*_2,3_=10.8 Hz, H‐3_B_), 4.45–4.22 (m, 11 H, H‐2_A1_, H‐3_B1_, H‐4_A_, H‐4_A1_, H‐3_A_, H‐3_A1_, CH_2All_, CH_2Bn_), 4.07–3.99 (m, 4 H, CH_2All_, H‐2_A_, H‐4_B1_, H‐4_B_), 3.54–3.41 (m, 4 H, H‐2_B,_ H‐2_B1_, H‐5_B_, H‐5_B1_), 2.38 (brs, 12 H, CH_3Ac_), 2.23 (brs, 3 H, CH_3Ac_), 1.28 (d, 3 H, *J*_5,6_=6.0 Hz, H‐6_B_), 1.19 (d, 3 H, *J*_5,6_=6.4 Hz, H‐6_B1_). ^13^C NMR (CDCl_3_) *δ* 175.3–174.8 (br, 4C, CO_NAc_), 168.7, 168.3 (C‐6_A_, C‐6_A1_), 161.9, 161.8 (2C, CO_NTCA_), 137.4, 135.2, 135.1, 135.0, 133.2, 133.0 (C_q,Ar_), 133.5 (CH_All_), 129.0, 128.9, 128.8, 128.7, 128.6, 128.5, 128.4, 128.2, 128.1, 128.0, 127.9, 127.8, 127.7, 127.6, 126.3, 126.1, 125.9, 125.6, 125.2 (C_Ar_), 117.9 (CH_2All_), 98.9 (C‐1_A1_^*^, ^1^ *J*_C,H_=175 Hz), 98.8 (C‐1_B1_, ^1^ *J*_C,H_=167 Hz), 98.3 (C‐1_A_^*^, ^1^ *J*_C,H_=175 Hz), 97.6 (C‐1_B_, ^1^ *J*_C,H_=163 Hz), 92.2, 91.8 (2C, CCl_3_), 76.7 (C‐3_B1_), 76.2 (C‐5_A1_), 75.9 (C‐3_B_), 73.4 (C‐5_A_), 73.6 (C‐4_A_), 72.8, 72.6 (C‐3_A_, C‐3_A1_), 72.5 (CH_2Nap_), 72.0, 71.8 (CH_2Bn_), 71.4 (C‐4_A1_), 70.1 (CH_2All_), 68.7, 68.6 (C‐5_B_, C‐5_B1_), 67.5 (2C, CH_2Bn‐6_), 65.3 (C‐4_B_), 65.2 (C‐4_B1_), 59.3 (C‐2_A_), 59.1 (C‐2_A1_), 58.8 (C‐2_B1_), 55.3 (C‐2_B_), 27.7, 25.3, 21.4 (4C, CH_3Ac_), 17.4, 17.2 (C‐6_B_, C‐6_B1_). HRMS (ESI^+^): *m*/*z* [M+NH_4_]^+^ calcd for C_78_H_86_Cl_6_N_6_O_21_ 1722.4131; found 1722.4110.

**Allyl (benzyl 3‐*O*‐benzyl‐2‐(*N***,***N*** **‐diacetyl)amino‐2‐deoxy‐α‐l‐altropyranosyluronate)‐(1→3)‐(4‐azido‐2‐trichloroacetamido‐2,4,6‐trideoxy‐β‐d‐galactopyranosyl)‐(1→3)‐(benzyl 3‐*O*‐benzyl‐2‐(*N***,***N*** **‐diacetyl)amino‐2‐deoxy‐4‐*O*‐(2‐naphthylmethyl)‐α‐l‐altropyranosyluronate)‐(1→3)‐4‐azido‐2‐trichloroacetamido‐2,4,6‐trideoxy‐β‐d‐galactopyranoside (53)**: DDQ (108 mg, 475 μmol, 3.0 equiv) was added to tetrasaccharide **52** (270 mg, 158 μmol, 1.0 equiv) in DCM/phosphate buffer pH 7 (8:1, 18 mL) cooled to 0 °C. The biphasic mixture was stirred vigorously for 4 h while allowing the bath to slowly warm to rt. At completion, 10 % aq. NaHCO_3_ (10 mL) was added followed by DCM (20 mL). The DCM layer was separated, washed with water and brine, dried over Na_2_SO_4_, filtered, and concentrated under reduced pressure. The crude was purified by flash chromatography with Tol/EtOAc (75:25→70:30). Alcohol **53** (180 mg, 115 μmol, 73 %), obtained as a white solid, had *R*_f_ 0.3 (Tol/EtOAc, 6:4). ^1^H NMR (CDCl_3_) *δ* 7.44–7.13 (m, 20 H, H_Ar_), 6.97 (d, *J*_2,NH_=6.8 Hz, NH_B1_), 6.80 (d, *J*_2,NH_=7.2 Hz, NH_B_), 5.88 (m, 1 H, CH_All_), 5.68 (d, 1 H, *J*_1,2_=7.8 Hz, H‐1_A1_), 5.64 (d, 1 H, *J*_1,2_=8.0 Hz, H‐1_A_), 5.34–5.15 (m, 6 H, CH_2All_, CH_2Bn‐6_), 4.95 (d, 1 H, *J*_1,2_=8.0 Hz, H‐1_B1_), 4.79 (d, 1 H, *J*_4,5_=2.0 Hz, H‐5_A1_), 4.76 (d, 1 H, *J*_1,2_=8.4 Hz, H‐1_B_), 4.72 (d, 1 H, *J*_4,5_=2.4 Hz, H‐5_A_), 4.64 (dd, 1 H, *J*_3,4_=4.0 Hz, *J*_2,3_=10.4 Hz, H‐3_B_), 4.50–4.37 (m, 7 H, H‐3_B1_, H‐4_A_, H‐4_A1_, H‐3_A_, H‐3_A1_, CH_2Bn_), 4.32–4.24 (m, 3 H, CH_2Bn_, CH_2All_), 4.13–3.99 (m, 4 H, CH_2All_, H‐2_A1_, H‐2_A_, H‐4_B1_, H‐4_B_), 3.54–3.41 (m, 4 H, H‐2_B,_ H‐2_B1_, H‐5_B_, H‐5_B1_), 2.61 (d, 1 H, *J*_4,OH_=2.0 Hz, OH), 2.38–2.32 (brs, 9 H, CH_3Ac_), 2.22 (brs, 3 H, CH_3Ac_), 1.28 (d, 3 H, *J*_5,6_=6.0 Hz, H‐6_B_), 1.19 (d, 3 H, *J*_5,6_=6.4 Hz, H‐6_B1_). ^13^C NMR (CDCl_3_) *δ* 175.3, 174.7 (2C, CO_NAc_), 168.3, 168.2 (2C, C‐6_A1_), 161.9, 161.8 (2C, CO_NTCA_)_,_ 137.4, 136.9, 135.0 (2C) (C_q,Ar_), 133.5 (CH_All_), 129.0, 128.9, 128.7 (2C), 128.6 (2C), 128.5, 128.4, 128.3, 128.2, 128.0 (2C), 125.2 (C_Ar_), 117.9 (CH_2All_), 98.9 (C‐1_B1_, ^1^ *J*_C,H_=168 Hz), 98.4 (C‐1_A_, ^1^ *J*_C,H_=176 Hz), 98.4 (C‐1_A1_, ^1^ *J*_C,H_=177 Hz), 97.6 (C‐1_B_, ^1^ *J*_C,H_=162 Hz), 92.2, 91.9 (2C, CCl_3_), 76.7 (C‐3_B1_), 76.2 (C‐5_A1_), 75.7 (C‐3_B_), 75.2 (C‐5_A_), 72.6 (C‐4_A_), 72.5, 71.4 (C‐3_A_, C‐3_A1_), 72.3, 72.0 (2C, CH_2Bn_), 70.1 (CH_2All_), 68.7, 68.6 (C‐5_B_, C‐5_B1_), 67.5, 67.5 (2C, CH_2Bn‐6_), 66.5 (C‐4_A1_), 65.3 (C‐4_B_, C‐4_B1_), 59.3 (C‐2_A_), 59.1 (C‐2_A1_), 55.7 (C‐2_B1_), 55.2 (C‐2_B_), 27.7, 25.3, 21.4 (4C, CH_3Ac_), 17.4, 17.2 (C‐6_B_, C‐6_B1_). HRMS (ESI^+^): *m*/*z* [M+NH_4_]^+^ calcd for C_67_H_78_Cl_6_N_11_O_21_ 1582.3505; found 1582.3503.

**Allyl (benzyl 3‐*O*‐benzyl‐2‐(*N***,***N*** **‐diacetyl)amino‐2‐deoxy‐4‐*O*‐(2‐naphthylmethyl)‐α‐l‐altropyranosyluronate)‐(1→3)‐4‐azido‐2‐trichloroacetamido‐2,4,6‐trideoxy‐β‐d‐galactopyranosyl‐(1→3)‐(benzyl 3‐*O*‐benzyl‐2‐(*N***,***N*** **‐diacetyl)amino‐2‐deoxy‐α‐l‐altropyranosyluronate)‐(1→3)‐4‐azido‐2‐trichloroacetamido‐2,4,6‐trideoxy‐β‐d‐galactopyranosyl‐(1→3)‐(benzyl 3‐*O*‐benzyl‐2‐(*N***,***N*** **‐diacetyl)amino‐2‐deoxy‐α‐l‐altropyranosyluronate)‐(1→3)‐4‐azido‐2‐trichloroacetamido‐2,4,6‐trideoxy‐β‐d‐galactopyranoside (54)**: Hemiacetal **49** (131 mg, 144 μmol, 1.0 equiv) was dissolved in acetone (7.0 mL). PTFACl (30 μL, 187 μmol, 1.3 equiv) and Cs_2_CO_3_ (52 mg, 158 μmol, 1.1 equiv) were added and the mixture stirred at rt for 2 h. Solids were filtered off over a pad of Celite and washed with acetone (5 mL) twice. The filtrate was concentrated under reduced pressure and the crude donor, isolated as a 4:1 mix of PTFA **50** and oxazoline **51**, was subjected to the next step without further purification.

The crude mix of donors **50**/**51** (144 μmol theo., 1.25 equiv) and acceptor **53** (180 mg, 115 μmol, 1.0 equiv) were coevaporated with anhyd. toluene (5 mL) twice and then dried extensively under high vacuum. The mixture, dissolved in anhyd. DCM (6.0 mL), was stirred with freshly activated MS 4 Å (200 mg) for 30 min at rt under an Ar atmosphere, and cooled to 0 °C. TfOH (1.0 μL, 0.05 equiv) was added and after stirring for 30 min at this temperature, a TLC analysis indicated donor consumption and the presence of a major new product together with some unreacted acceptor. Et_3_N (2.0 μL) was added and after 10 min, solids were filtered off and washed with DCM (5 mL) twice. Volatiles were evaporated and the residue was purified by flash chromatography (Tol/EA 75:25→60:40) to give first the condensation product **54** (200 mg, 81 μmol, 71 %; corr. yield 98 %) as a white solid, followed by some unreacted **53** (50 mg, 28 %). Hexasaccharide **54** had *R*_f_ 0.25 (Tol/EtOAc 4:1). ^1^H NMR (CDCl_3_) *δ* 7.85–7.11 (m, 37 H, H_Ar_), 6.94 (d, *J*_2,NH_=6.8 Hz, NH_B2*_), 6.90 (d, *J*_2,NH_=6.8 Hz, NH_B1*_), 6.74 (d, *J*_2,NH_=7.2 Hz, NH_B_), 5.91–5.81 (m, 1 H, CH_All_), 5.78 (d, 1 H, *J*_1,2_=7.6 Hz, H‐1_A2_), 5.65 (d, 1 H, *J*_1,2_=7.8 Hz, H‐1_A1*_), 5.64 (d, 1 H, *J*_1,2_=7.8 Hz, H‐1_A*_), 5.31–5.16 (m, 8 H, CH_2All_, 3CH_2Bn‐6_), 5.03 (d, 1 H, *J*_1,2_=8.1 Hz, H‐1_B2*_), 4.99 (d, 1 H, *J*_1,2_=8.1 Hz, H‐1_B1*_), 4.82–4.80 (m, 2 H, CH_2Nap_), 4.80 (d_po_, 1 H, *J*_4,5_=2.2 Hz, H‐5_A*_) 4.78 (d_po_, 1 H, *J*_4,5_=2.4 Hz, H‐5_A1*_), 4.76 (d_po_, 1 H, *J*_1,2_=8.3 Hz, H‐1_B_), 4.72 (d, 1 H, *J*_4,5_=2.3 Hz, H‐5_A2*_), 4.68 (dd, 1 H, *J*_3,4_=4.0 Hz, *J*_2,3_=10.7 Hz, H‐3_B2*_), 4.59 (dd, 1 H, *J*_3,4_=4.0 Hz, *J*_2,3_=10.6 Hz, H‐3_B1*_), 4.44 (dd, 1 H, *J*_3,4_=3.8 Hz, *J*_2,3_=10.7 Hz, H‐3_B_), 4.41–4.22 (m, 15 H, H‐2_A2_, H‐3_B_, H‐4_A_, H‐4_A1_, H‐4_A2_, H‐3_A_, H‐3_A1_, H‐3_A2_, CH_2All_, 3CH_2Bn_), 4.09 (bd_po_, 1 H, H‐4_B1_*), 4.07 (bd_po_, 1 H, H‐4_B_), 4.05–3.99 (m, 4 H, CH_2All_, H‐2_A_, H‐2_A1_, H‐4_B2*_), 3.52 (dt, 1 H, H‐2_B_), 3.50–3.43 (m, 3 H, H‐5_B_, H‐5_B1_, H‐5_B2_), 3.42–3.36 (m, 2 H, H‐2_B1_, H‐2_B2_), 2.41 (brs, 12 H, 4CH_3Ac_), 2.23 (brs, 6 H, 2CH_3Ac_), 1.30 (d, 3 H, *J*_5,6_=6.0 Hz, H‐6_B*_), 1.23 (d, 3 H, *J*_5,6_=6.4 Hz, H‐6_B1*_) 1.12 (d, 3 H, *J*_5,6_=6.4 Hz, H‐6_B2*_). ^13^C NMR (CDCl_3_) *δ* 175.6, 175.3, 175.0, 174.7 (br, 6C, CO_NAc_), 168.9, 168.8, 168.7 (3C, C‐6_A_), 162.3, 161.8, 161.7 (3C, CO_NTCA_), 137.5, 137.4, 135.2, 135.1, 135.0 (C_q,Ar_), 133.5 (CH_All_), 133.2, 133.0 (C_q,Ar_), 129.0, 128.9, 128.8, 128.7, 128.6, 128.5, 128.4, 128.2, 128.1, 128.0, 127.8, 127.7, 126.4, 126.1, 125.9, 125.6, 125.2 (27C, C_Ar_), 117.9 (CH_2All_), 98.9 (C‐1_B2*_, ^1^ *J*_C,H_=167 Hz), 98.8 (C‐1_A2_, ^1^ *J*_C,H_=175 Hz), 98.7 (C‐1_B1*_, ^1^ *J*_C,H_=167 Hz), 98.4 (C‐1_A1*_, ^1^ *J*_C,H_=177 Hz), 98.3 (C‐1_A*_, ^1^ *J*_C,H_=177 Hz), 97.6 (C‐1_B_, ^1^ *J*_C,H_=163 Hz), 76.7, 75.7, 75.1 (C‐3_B_, C‐3_B1_, C‐3_B2_), 76.2, 74.0 (C‐5_A_, C‐5_A1_, C‐5_A2_), 73.7, 71.3 (C‐4_A,_ C‐4_A1_, C‐4_A2_), 72.8, 72.4, 70.9 (C‐3_A_, C‐3_A1_, C‐3_A2_), 72.5 (CH_2Nap_), 72.0, 71.9 (3C, CH_2Bn_), 70.1 (CH_2All_), 68.6, 68.5 (C‐5_B_, C‐5_B1,_ C‐5_B2_), 67.5 (3C, CH_2Bn‐6_), 65.3, 65.2 (C‐4_B_, C‐4_B1_, C‐4_B2_), 59.5, 59.4, 59.1 (C‐2_A_, C‐2_A1_, C‐2_A2_), 55.9, 55.2 (C‐2_B_, C‐2_B1_, C‐2_B2_), 27.7, 25.4 (3C, CH_3Ac_), 17.4, 17.3 (C‐6_B_, C‐6_B1_, C‐6_B2_). HRMS (ESI^+^): *m*/*z* [M+2NH_4_]^2+^ calcd for C_110_H_124_Cl_9_N_17_O_31_ 1246.7922; found 1246.7922.

**Allyl (benzyl 3‐*O*‐benzyl‐2‐deoxy‐2‐(*N***,***N*** **‐diacetyl)amino‐α‐l‐altropyranosyluronate)‐(1→3)‐(4‐azido‐2‐trichloroacetamido‐2,4,6‐trideoxy‐β‐d‐galactopyranosyl)‐(1→3)‐(benzyl 3‐*O*‐benzyl‐2‐deoxy‐2‐(*N***,***N*** **‐diacetyl)amino‐α‐l‐altropyranosyluronate)‐(1→3)‐(4‐azido‐2‐trichloroacetamido‐2,4,6‐trideoxy‐β‐d‐galactopyranosyl)‐(1→3)‐(benzyl 3‐*O*‐benzyl‐2‐deoxy‐2‐(*N***,***N*** **‐diacetyl)amino‐α‐l‐altropyranosyluronate)‐(1→3)‐4‐azido‐2‐trichloroacetamido‐2,4,6‐trideoxy‐β‐d‐galactopyranoside (55)**: DDQ (55 mg, 244 μmol, 3.0 equiv) was added to hexasaccharide **54** (200 mg, 81 μmol, 1.0 equiv) in DCM (8.0 mL) and phosphate buffer pH 7 (1.0 mL). The biphasic mixture was cooled to 0 °C and stirred for 2 h. Additional DDQ (200 mg, 81 μmol, 1.0 equiv) was added and stirring was pursued for another 4 h while the bath temperature reached rt. A TLC analysis (Tol/EtOAC 3:1) showed the absence of the fully protected **54** (*R*_f_ 0.6) and the presence of a more polar spot (*R*_f_ 0.4). 10 % Aq. NaHCO_3_ (5 mL) was added followed by DCM (15 mL). The DCM layer was separated, washed with water and brine, dried over Na_2_SO_4_, filtered, and concentrated under reduced pressure. The residue was purified by flash chromatography (Tol/EtOAc 4:1→3:1) to give alcohol **55** (120 mg, 51 μmol, 64 %) as a white solid. Hexasaccharide **55** had ^1^H NMR (CDCl_3_) *δ* 7.36–7.13 (m, 30 H, H_Ar_), 6.89 (d_po_, *J*_2,NH_=6.8 Hz, NH_B*_), 6.87 (d, *J*_2,NH_=6.8 Hz, NH_B1*_), 6.72 (d, *J*_2,NH_=7.6 Hz, NH_B2*_), 5.90–5.80 (m, 1 H, CH_All_), 5.68 (d, 1 H, *J*_1,2_=8.0 Hz, H‐1_A*_), 5.65 (d_po_, 1 H, *J*_1,2_=7.8 Hz, H‐1_A1*_), 5.65 (d_po_, 1 H, *J*_1,2_=7.8 Hz, H‐1_A2*_), 5.35–5.15 (m, 8 H, CH_2All_, 3CH_2Bn‐6_), 5.01 (d_po_, 1 H, *J*_1,2_=8.0 Hz, H‐1_B*_), 4.94 (d, 1 H, *J*_1,2_=8.4 Hz, H‐1_B1*_), 4.79–4.75 (m, 6 H, CH_2Nap_, H‐5_A*_, H‐5_A1*_, H‐3_B*_, H‐1_B2*_), 4.72 (d, 1 H, *J*_4,5_=2.4 Hz, H‐5_A2*_), 4.69 (dd, 1 H, *J*_3,4_=4.0 Hz, *J*_2,3_=10.8 Hz, H‐3_B1*_), 4.58 (dd, 1 H, *J*_3,4_=4.0 Hz, *J*_2,3_=10.4 Hz, H‐3_B2*_), 4.45–4.23 (m, 15 H, H‐3_B_, H‐4_A_, H‐4_A1_, H‐4_A2_, H‐3_A_, H‐3_A1_, H‐3_A2_, CH_2All_, 3CH_2Bn_), 4.08–3.99 (m, 7 H, H‐2_A_, H‐2_A1_, H‐2_A2_, H‐4_B_, H‐4_B1_, H‐4_B2_, CH_2All_), 3.54–3.35 (m, 6 H, H‐2_B,_ H‐2_B1_, H‐2_B2_, H‐5_B_, H‐5_B1_, H‐5_B2_), 2.52 (d, 1 H, *J*_4,OH_=2.4 Hz, OH), 2.40–2.17 (brm, 18 H, CH_3Ac_), 1.29–1.12 (m, 12 H, H‐6_B_, H‐6_B1_, H‐6_B2_). ^13^C NMR (CDCl_3_) *δ* 175.3 (6C, CO_NAc_), 168.3 (2C), 168.1 (3C, C‐6_A_), 161.8 (2C), 161.7 (3C, CO_NTCA_), 133.5, 133.5 (CH_All_), 137.4, 136.8, 135.0 (2C), 127.8, 127.0 (C_q,Ar_), 129.0, 129.4, 128.8 (2C), 128.7 (2C), 128.6 (2C), 128.5 (2C), 128.4 (2C), 128.3, 128.2, 128.0 (2C), 125.2 (30C, C_Ar_), 117.9 (CH_2All_), 98.9 (C‐1_B*_, ^1^ *J*_C,H_=166 Hz), 98.7 (C‐1_B1*_, ^1^ *J*_C,H_=167 Hz), 98.4 (C‐1_A_, ^1^ *J*_C,H_=175 Hz), 98.3 (2C, C‐1_A1*_, C‐1_A2*_, ^1^ *J*_C,H_=175 Hz), 97.6 (C‐1_B2*_, ^1^ *J*_C,H_=164 Hz), 92.6, 91.8 (3C, CCl_3_), 76.6, 75.5, 75.1 (C‐3_B_, C‐3_B1_, C‐3_B2_), 76.2 (2C), 75.2 (C‐5_A_, C‐5_A1_, C‐5_A2_), 72.8, 72.6, 72.4 (C‐3_A_, C‐3_A1_, C‐3_A2_), 72.3, 72.0 (3C, 3CH_2Bn_), 71.3, 71.0 (C‐4_A,_ C‐4_A1_), 70.1 (CH_2All_), 68.6 (2C), 68.5 (C‐5_B_, C‐5_B1,_ C‐5_B2_), 67.7, 67.5 (3C, CH_2Bn‐6_), 66.5 (C‐4_A2_), 65.4, 65.3, 65.2 (C‐4_B_, C‐4_B1_, C‐4_B2_), 59.5, 59.3, 59.0 (C‐2_A_, C‐2_A1_, C‐2_A2_), 55.8 (2C), 55.3 (C‐2_B_, C‐2_B1_, C‐2_B2_), 27.7 (6C, CH_3Ac_), 17.4, 17.3, 17.2 (3C, C‐6_B_). HRMS (ESI^+^): *m*/*z* [M+NH_4_]^+^ calcd for C_99_H_112_Cl_9_N_16_O_31_ 2335.4876; found 2335.4871.

**Allyl (benzyl 3‐*O*‐benzyl‐2‐deoxy‐2‐(*N***,***N*** **‐diacetyl)amino‐4‐*O*‐(2‐naphthylmethyl)‐α‐l‐altropyranosyluronate)‐(1→3)‐(4‐azido‐2‐trichloroacetamido‐2,4,6‐trideoxy‐β‐d‐galactopyranosyl)‐(1→3)‐(benzyl 3‐*O*‐benzyl‐2‐deoxy‐2‐(*N***,***N*** **‐diacetyl)amino‐α‐l‐altropyranosyluronate)‐(1→3)‐(4‐azido‐2‐trichloroacetamido‐2,4,6‐trideoxy‐β‐d‐galactopyranosyl)‐(1→3)‐(benzyl 3‐*O*‐benzyl‐2‐deoxy‐2‐(*N***,***N*** **‐diacetyl)amino‐α‐l‐altropyranosyluronate)‐(1→3)‐(4‐azido‐2‐trichloroacetamido‐2,4,6‐trideoxy‐β‐d‐galactopyranosyl)‐(1→3)‐(benzyl 3‐*O*‐benzyl‐2‐deoxy‐2‐(*N***,***N*** **‐diacetyl)amino‐α‐l‐altropyranosyluronate)‐(1→3)‐4‐azido‐2‐trichloroacetamido‐2,4,6‐trideoxy‐β‐d‐galactopyranoside (56)**: A 4:1 mix of donors **50/51** (92 mg, 85 μmol, 1.5 equiv) and acceptor **55** (132 mg, 57 μmol, 1.0 equiv) was coevaporated with toluene (3 mL) twice and dried extensively under high vacuum. The mixture, dissolved in anhyd. DCM (4.0 mL), was stirred for 45 min at rt with freshly activated MS 4Å and cooled to 0 °C. TfOH (3.8 μL, 0.05 equiv) was added and the reaction mixture was stirred at this temperature. At completion, as revealed by TLC analysis (Tol/EtOAc 7:3), Et_3_N (5.0 μL) was added and after 10 min, solids were filtered over a fitted funnel. The filtrate was concentrated to dryness and the residue was purified by flash chromatography (Tol/EtOAc 80:20→70:30) to give first the glycosylation product **56** (60 mg, 19 μmol, 33 %, corr. yield, 54 % wrt acceptor) as a white solid followed by the remaining unreacted acceptor **55** (50 mg, 38 %). Octasaccharide **56** had *R*_f_ 0.35 (Tol/EtOAc 4:1). ^1^H NMR (CDCl_3_) *δ* 7.85–7.74 (m, 5 H, H_Ar_), 7.52–7.11 (m, 42 H, H_Ar_), 6.90–6.86 (m, 3 H, NH_B1_, NH_B3_, NH_B3_), 6.71 (d, 1 H, *J*_2,NH_=7.3 Hz, NH_B_), 5.90–5.75 (m, 1 H, CH_All_), 5.78 (d, 1 H, *J*_1,2_=7.6 Hz, H‐1_A3_), 5.66–5.62 (m, 3 H, H‐1_A_, H‐1_A1_, H‐1_A2_), 5.29–5.16 (m, 10 H, CH_2All_, 4CH_2Bn‐6_), 5.03–5.01 (m, 2 H, H‐1_B1_, H‐1_B2_), 4.96 (d, 1 H, *J*_1,2_=8.0 Hz, H‐1_B3_), 4.83–4.80 (m, 3 H, CH_2Nap_, H‐5_A_), 4.78–4.76 (m, 3 H, H‐1_B_, H‐5_A1*_, H‐5_A2*_), 4.72–4.55 (m, 4 H, H‐3_B_, H‐3_B1_, H‐3_B2_, H‐5_A3*_), 4.45–4.22 (m, 17 H, H‐2_A*_, H‐3_B3*_, H‐3_A_, H‐3_A1_, H‐3_A2_, H‐3_A3_, H‐4_A_, H‐4_A1_, H‐4_A2_, H‐4_A3_, CH_2All_, 3CH_2Bn_), 4.09–3.98 (m, 8 H, CH_2All_, H‐2_A1_, H‐2_A2_, H‐2_A3_, 4H‐4_B_), 3.55–3.32 (m, 8 H, 4H‐2_B,_ 4H‐5_B_), 2.40–2.19 (m_po_, 24 H, 8CH_3NAc_), 1.30–1.18 (d, 12 H, *J*_5,6_=6.4 Hz, H‐6_B_, H‐6_B1,_ H‐6_B2,_ H‐6_B3_). ^13^C NMR (Partial, CDCl_3_) *δ* 175.5, 175.0, 174.6 (4C, CO_NAc_), 168.7 (2C), 168.3, 168.2 (4C, C‐6_A_), 162.3, 161.8, 161.7 (4C, CO_NTCA_), 137.4 (2C) 137.3, 135.2 (2C), 135.1, 135.0 (2C), 133.2, 133.0, 126.0 (C_q,Ar_), 133.5 (CH_All_), 129.0, 128.9, 128.8, 128.7 (2C), 128.6, 128.5 (2C), 128.4 (2C), 128.2, 128.1, 128.0, 127.8, 127.7, 126.4, 126.1, 125.9, 125.6, 125.2 (C_Ar_), 117.9 (CH_2All_), 98.9 (C‐1_A3*_, ^1^ *J*_C,H_=178 Hz), 98.8 (C‐1_B3*_, ^1^ *J*_C,H_=166 Hz), 98.7 (C‐1_B1_, C‐1_B2_, ^1^ *J*_C,H_=168 Hz), 98.4 (C‐1_A1*_, C‐1_A2*_, ^1^ *J*_C,H_=178 Hz), 98.3 (C‐1_A*_, ^1^ *J*_C,H_=178 Hz), 97.6 (C‐1_B_, ^1^ *J*_C,H_=162 Hz), 92.2, 91.8 (4C, CCl_3_), 76.6, 75.8, 75.1, 74.9 (C‐3_B_, C‐3_B1_, C‐3_B2_, C‐3_B3_), 76.2, 73.7 (C‐5_A_, C‐5_A1_, C‐5_A2_, C‐5_A3_), 74.0, 72.9, 72.8 (C‐4_A_, C‐4_A1_, C‐4_A2_, C‐4_A3_), 72.5 (CH_2Nap_), 71.8, 70.9 (C‐3_A_, C‐3_A1_, C‐3_A2_), 72.0, 71.9 (4C, CH_2Bn_), 70.1 (CH_2All_), 68.6, 68.5 (C‐5_B_, C‐5_B1_, C‐5_B2_, C‐5_B3_), 67.5 (4C, CH_2Bn‐6_), 65.4 (C‐4_B,_ C‐4_B1_, C‐4_B2_, C‐4_B3_), 59.5, 59.4, 59.1 (C‐2_A_, C‐2_A1_, C‐2_A2_, C‐2_A3_), 55.8, 55.3 (C‐2_B_, C‐2_B1_, C‐2_B2_, C‐2_B3_), 27.6, 25.2, 12.4 (8C, CH_3Ac_), 17.4, 17.3, 17.2 (C‐6_B_, C‐6_B1_, C‐6_B2_, C‐6_B3_). HRMS (ESI^+^): *m*/*z* [M+NH_4_]^+^ calcd for C_142_H_154_Cl_12_N_21_O_41_ 3229.6899; found 3230.7006.

**Propyl (2‐acetamido‐2‐deoxy‐α‐l‐altropyranosyluronic acid)‐(1→3)‐(2‐acetamido‐4‐amino‐2,4,6‐trideoxy‐β‐d‐galactopyranosyl)‐(1→4)‐(2‐acetamido‐2‐deoxy‐α‐l‐altropyranosyluronic acid)‐(1→3)‐(2‐acetamido‐4‐amino‐2,4,6‐trideoxy‐β‐d‐galactopyranoside (2)**: Tetrasaccharide **54** (30 mg, 18 μmol, 1.0 equiv) was dissolved in *t*BuOH/DCM/H_2_O (10 mL, 20:5:2, v/v/v) and 20 % Pd(OH)_2_/C (100 mg) was added. The reaction mixture was degassed several times and stirred under a hydrogen atmosphere for 48 h. A follow up by HRMS revealed the presence of a major product corresponding the 2_A_‐NAc_2_,2_B_‐NAc product (HRMS: C_39_H_62_N_6_O_21_Na [M+Na]^+^ *m*/*z* 973.4270). The suspension was passed through a syringe filter (0.2 μm) and washed thoroughly with methanol. The filtrate was concentrated and the crude product was dried under vacuum. The resulting white powder was dissolved in methanol (3.0 mL) and hydroxylamine (2.2 mg, 36 μmol, 2.0 equiv) was added. LCMS monitoring revealed that after 4 h, no intermediate remained and the desired product (LCMS: [*M*+H]^+^ *m*/*z* 867.3) was present to a large extent. The reaction mixture was neutralized with phosphate buffer with frequent pH monitoring to achieve pH 7, then diluted with water (6.0 mL) and lyophilized. Purification of the crude material by semi‐preparative RP‐HPLC gave the propyl glycoside **2** as a white solid (5.9 mg, 6.8 μmol, 39 %). Tetrasaccharide **2** had RP‐HPLC (215 nm) Rt=12.3 min (conditions A), Rt=13.6 min (conditions B), ^1^H NMR (D_2_O) *δ* 4.87 (d, 1 H, *J*_1,2_=8.4 Hz, H‐1_A1_), 4.77 (d_po_, 1 H, *J*_1,2_=8.4 Hz, H‐1_A_), 4.74 (d_po_, 1 H, *J*_1,2_=8.4 Hz, H‐1_B1_), 4.66 (brs, 1 H, H‐5_A_), 4.59 (C‐5_A1_), 4.44 (d_po_, 2 H, *J=*8.4 Hz, H‐1_B1_, H‐4_A_), 4.36 (brs, 1 H, H‐4_A1_), 4.15–4.08 (m, 2 H, H‐3_B_, H‐3_B1_), 4.03–3.99 (m_po_, 2 H, H‐5_B_, H‐5_B1_), 3.96–3.90 (m, 2 H, H‐2_A_, H‐4_B_), 3.80–3.66 (m, 7 H, H‐3_A_, H‐3_A1_, H‐2_A1_, H‐2_B_, H‐2_B1_, H‐4_B1_, OCH_2Pr_), 3.51–3.49 (m_po_, 1 H, OCH_2Pr_), 1.99, 1.94 (2 s, 12 H, CH_3Ac_), 1.51–1.48 (m, 2 H, CH_2Pr_), 1.29 (d_po_, 6 H, H‐6_B_), 0.82 (t, 3 H, *J=*7.2 Hz, CH_3Pr_).^13^C NMR (D_2_O) *δ* 174.6, 174.5 (2C), 174.0 (4C, CO_NAc_), 172.7, 172.5 (C‐6_A_, C‐6_A1_), 102.9 (C‐1_B1_, ^1^ *J*_C,H_=168 Hz), 101.6 (C‐1_B_, ^1^ *J*_C,H_=166 Hz), 101.1 (C‐1_A_, C‐1_A1_, ^1^ *J*_C,H_=170 Hz, ^1^ *J*_C,H_=168 Hz), 76.7 (C‐4_A_), 76.5, 76.3 (C‐5_A_, C‐5_A1_), 76.0 (C‐3_B_, C‐3_B1_), 72.7 (OCH_2Pr_), 68.5 (C‐4_A1_), 67.8, 67.6 (C‐3_A_, C‐3_A1_), 67.4, 67.3 (C‐5_B_, C‐5_B1_), 54.8 (C‐4_B_), 54.7 (C‐4_B1_), 51.5 (C‐2_A1_), 51.3 (C‐2_A_), 51.0, 50.8 (C‐2_B_, C‐2_B1_), 23.3, 22.2 (4C, CH_3Ac_), 21.2 (CH_2Pr_), 15.6, 15.5 (C‐6_B_, C‐6_B1_), 9.5 (CH_3Pr_). HRMS (ESI^+^): *m*/*z* [M+Na]^+^ calcd for C_35_H_58_N_6_O_19_Na 889.3649; found 889.3636.

**Propyl (2‐acetamido‐2‐deoxy‐α‐l‐altropyranosyluronic acid)‐(1→3)‐(2‐acetamido‐4‐amino‐2,4,6‐trideoxy‐β‐d‐galactopyranosyl)‐(1→4)‐(2‐acetamido‐2‐deoxy‐α‐l‐altropyranosyluronic acid)‐(1→3)‐(2‐acetamido‐4‐amino‐2,4,6‐trideoxy‐β‐d‐galactopyranosyl)‐(1→4)‐(2‐acetamido‐2‐deoxy‐α‐l‐altropyranosyluronic acid)‐(1→3)‐2‐acetamido‐4‐amino‐2,4,6‐trideoxy‐β‐d‐galactopyranoside (3)**: Hexasaccharide **54** (70 mg, 31 μmol, 1.0 equiv) was dissolved in *t*BuOH/DCM/H_2_O (19 mL, 20:5:2, v/v/v). 20 % Pd(OH)_2_/C (120 mg) was added and the suspension was degassed repeatedly. After stirring under a hydrogen atmosphere for 48 h, monitoring by LCMS analysis showed the presence of the targeted intermediate (LCMS: [*M*+H]^+^ *m*/*z* 1396.4). The suspension was passed through a 0.2 μm filter and washed extensively with methanol. The filtrate was concentrated and the crude material was dried under vacuum for 2 h. The obtained white powder was dissolved in methanol (3.0 mL) and hydroxylamine (6.1 mg, 85 μmol, 3.0 equiv) was added. After stirring for 3 h, LCMS analysis showed the complete disappearance of the triacetate intermediate and the presence of the desired product (LCMS: [*M*+H]^2+^ *m*/*z* 635.2). Water (6.0 mL) was added and the reaction mixture was lyophilized. Purification of the crude material by semi‐preparative RP‐HPLC gave hexasaccharide **3** as a white solid (12 mg, 9.4 μmol, 31 %). The propyl glycoside **3** had RP‐HPLC (215 nm) Rt=11.3 min (conditions A), Rt=13.3 min (conditions B). ^1^H NMR (D_2_O) *δ* 4.88 (d, 1 H, *J*_1,2_=8.4 Hz, H‐1_A2_), 4.78 (d_po_, 2 H, *J*_1,2_=8.4 Hz, H‐1_A_, H‐1_A1_), 4.75 (d_po_, 1 H, *J*_1,2_=8.4 Hz, H‐1_B1_, H‐1_B2_), 4.70 (brs_po_, 2 H, H‐5_A_, H‐5_A1_), 4.61 (brs, 1 H, H‐5_A2_), 4.46–4.44 (m_po_, 2 H, H‐1_B_, H‐4_A_), 4.36 (brs, 1 H, H‐4_A1_), 4.16–4.09 (m, 3 H, H‐3_B_, H‐3_B1_, H‐3_B2_), 4.06–3.90 (m, 7 H, H‐5_B_, H‐5_B1_, H‐5_B2_, H‐2_A_, H‐2_A1_, H‐4_A,_ H‐4_B_), 3.86–3.72 (m, 10 H, H‐3_A_, H‐3_A1_, H‐3_A2_, H‐4_B1_, H‐4_B2_, H‐2_A_, H‐2_B_, H‐2_B1_, H‐2_B2_, OCH_2Pr_), 3.51–3.49 (m_po_, 1 H, OCH_2Pr_), 1.99, 1.98, 1.96, 1.95 (4 s, 18 H, CH_3Ac_), 1.54–1.45 (m, 2 H, CH_2Pr_), 1.30–1.29 (d_po_, 9 H, H‐6_B_), 0.82 (t, 3 H, *J=*7.2 Hz, CH_3Pr_).^13^C NMR (D_2_O) *δ* 174.6, 174.5, 174.4, 174.0 (6C, CO_NAc_), 172.6, 172.2 (C‐6_A_, C‐6_A1_, C‐6_A2_), 102.9, 102.8 (C‐1_A_, C‐1_A_, C‐1_A_, ^1^ *J*_C,H_=174.2 Hz, ^1^ *J*_C,H_=170.2 Hz), 101.6 (C‐1_B_, ^1^ *J*_C,H_=162.0 Hz), 101.2, 101.1 (C‐1_B1_, C‐1_B2_, ^1^ *J*_C,H_=166.2 Hz), 76.6 (C‐4_A_, C‐4_A1_), 76.3, 76.1 (C‐5_A_, C‐5_A1_, C‐5_A2_), 75.7 (C‐3_B_, C‐3_B1_, C‐3_B2_), 72.7 (OCH_2Pr_), 68.4 (C‐4_A2_), 68.2, 67.8 (C‐3_A_, C‐3_A1_, C‐3_A2_), 67.6, 67.3 (C‐5_B_, C‐5_B1_, C‐5_B2_), 54.8, 54.7 (C‐4_B_, C‐4_B1_, C‐4_B2_), 51.5, 51.3 (C‐2_A_, C‐2_A1_, C‐2_A2_), 50.9, 50.8 (C‐2_B_, C‐2_B1_, C‐2_B2_), 23.3, 22.2 (6C, CH_3Ac_), 21.2 (CH_2Pr_), 15.6 (C‐6_B_, C‐6_B1_, C‐6_B2_), 9.5 (CH_3Pr_). HRMS (ESI^+^): *m*/*z* [M+2H]^2+^ calcd for C_51_H_85_N_9_O_28_ 635.7747; found 635.7736.

**Propyl (2‐acetamido‐2‐deoxy‐α‐l‐altropyranosyluronic acid)‐(1→3)‐(2‐acetamido‐4‐amino‐2,4,6‐trideoxy‐β‐d‐galactopyranosyl)‐(1→4)‐(2‐acetamido‐2‐deoxy‐α‐l‐altropyranosyluronic acid)‐(1→3)‐(2‐acetamido‐4‐amino‐2,4,6‐trideoxy‐β‐d‐galactopyranosyl)‐(1→4)‐(2‐acetamido‐2‐deoxy‐α‐l‐altropyranosyluronic acid)‐(1→3)‐(2‐acetamido‐4‐amino‐2,4,6‐trideoxy‐β‐d‐galactopyranosyl)‐(1→4)‐(2‐acetamido‐2‐deoxy‐α‐l‐altropyranosyluronic acid)‐(1→3)‐2‐acetamido‐4‐amino‐2,4,6‐trideoxy‐β‐d‐galactopyranoside (4)**: Octasaccharide **56** (20 mg, 12 μmol, 1.0 equiv) was dissolved in *t*BuOH/DCM/H_2_O (16.5 mL, 20:5:2, v/v/v). 20 % Pd(OH)_2_/C (50 mg) was added and the suspension was degassed repeatedly. After stirring under a hydrogen atmosphere for 48 h, the suspension was passed through a 0.2 μm filter and washed extensively with methanol. The filtrate was concentrated and the crude material was dried under vacuum for 2 h. HRMS analysis of the crude material showed the presence of the tetra‐2‐*N*‐acetylacetamido product ([M+2H]^2+^ calculated for: C_75_H_118_N_12_O_41_ 921.3758, found 921.3753). The obtained white powder was dissolved in methanol (3.0 mL) and hydroxylamine (3.0 mg, 49 μmol, 4.0 equiv) was added. After stirring for 6 h, LCMS analysis revealed the presence of a product of the desired mass (LCMS: [*M*+H]^2+^ *m*/*z* 837.2). Water (6.0 mL) was added and the reaction mixture was lyophilized. Purification of the crude material by semi‐preparative RP‐HPLC gave octasaccharide **4** as a white solid (1.7 mg, 1.01 μmol, 16 %). The propyl glycoside **4** had RP‐HPLC (215 nm) Rt=11.3 min (conditions A’). ^1^H NMR (D_2_O, 800 MHz) *δ* 4.81–4.79 (2d_po_, 3 H, H‐1_A_), 4.67–4.65 (m_po_, 4 H, H‐1_A_, 3H‐1_B_), 4.54 (brd_po_, 2 H, H‐4_A_), 4.42–4.37 (brs, 4 H, 3H‐5_A_, H‐1_B_), 4.31–4.30 (m_po_, 3 H, H‐5_A_, 2H‐4_A_), 4.11–4.05 (m, 4 H, H‐3_B_), 4.01–3.99 (q, 4 H, H‐5_B_), 3.92–3.87 (m, 3 H, H‐2_B_, 2H‐4_B_), 3.82–3.68 (m, 9 H, 4H‐2_A_, 3H‐2_B_, 2H‐4_B_), 3.67–3.65 (m_po_, 3 H, 2H‐3_A_, OCH_2Pr_), 3.59–3.55 (m_po_, 3 H, 2H‐3_A_, OCH_2Pr_), 1.96–1.89 (m_po_, 24 H, CH_3Ac_), 1.47–1.44 (m, 2 H, CH_2Pr_), 1.26 (m_po_, 12 H, H‐6_B_), 0.78 (t, 3 H, *J=*7.2 Hz, CH_3Pr_). ^13^C NMR (D_2_O, 800 MHz) *δ* 174.8, 174.6, 174.4, 173.9 (8C, CO_NAc_), 163.1, 162.9 (4C, C‐6_A_), 103.0 (2C, C‐1_A_), 101.6 (C‐1_A,_ C‐1_B_), 101.1 (2C, C‐1_B_), 100.9 (C‐1_B_, C‐1_A_), 77.9, 77.4, 77.3 (3C, C‐4_A_), 76.0, 75.8, 75.7, 75.5 (4C, C‐3_B_), 72.6 (OCH_2Pr_), 72.0 (C‐4_A3_), 69.1, 68.1, 67.7 (4C, C‐3_A_), 67.4, 67.2 (4C, C‐5_B_), 54.9, 54.7 (4C, C‐4_B_), 51.4 (4C, C‐2_A_), 50.9, 50.8 (4C, C‐2_B_), 22.3, 22.1 (8C, CH_3Ac_), 21.2 (CH_2Pr_), 15.5 (4C, C‐6_B_), 9.4 (CH_3Pr_). HRMS (ESI^+^): *m*/*z* [M+2H]^2+^ calcd for C_61_H_110_N_12_O_37_ 837.3547; found 837.3542.

**Supporting Information**: The Supporting Information contains the following: Schemes S1–S6 and Tables S1–S3. General procedures. Experimental procedures and analytical data for compounds **1**, **2**, **9**–**11**, **14**–**19**, **21**–**29**, **38**–**41**, **57**–**65**, **S1**–**S4** and **S7**–**S10**. ^1^H and ^13^C NMR spectra of novel compounds.

## Conflict of interest

The authors declare no conflict of interest.

## Supporting information

As a service to our authors and readers, this journal provides supporting information supplied by the authors. Such materials are peer reviewed and may be re‐organized for online delivery, but are not copy‐edited or typeset. Technical support issues arising from supporting information (other than missing files) should be addressed to the authors.

Supplementary
