# Supplementary material for: Exploratory N‐Protecting Group Manipulation for the Total Synthesis of Zwitterionic Shigella sonnei Oligosaccharides
Source: Chemistry. 2021 Mar 1;27(18):5694–711. doi: 10.1002/chem.202003480 (PMC8048667; doi:10.1002/chem.202003480)
Supplement: Supplementary file 1 — Supplementary [file CHEM-27-5694-s001.pdf]

Chemistry–A European Journal

Supporting Information

Exploratory N-Protecting Group Manipulation for the Total Synthesis of Zwitterionic *Shigella sonnei* Oligosaccharides

Debashis Dhara and Laurence A. Mulard*[a]

Table of contents

I. Abbreviations p S2

II. Supplementary schemes p S2

III. Supplementary tables p S5

IV. Experimental protocols and analytical data p S9

IVa. General procedures p S9

IVb. Compounds 1, 2, 9-11, 14-19, 21-29, 38-41, 57-65, S1-S4, and S7-S10 p S10

V. References p S34

VI. ^{1}H and ^{13}C spectra for compounds 2-4, 9-11, 14-19, 21-32, 34-41, 47-65, S1-S4, S7-S10 p S34

I. Abbreviations

Ac: acetyl, ACN: acetonitrile, Acet: acetone, All: allyl, anhyd.: anhydrous, aq.: aqueous, Ar: argon, BAIB: [bis-(acetoxy)iodo]benzene, Bn: benzyl, Boc: *tert*-butyloxycarbonyl; ^{t}BuOH: *tert*-butanol, cHex: cyclohexane, CSA: camphorsulfonic acid, $Cu(OTf)_2$: copper(II) trifluoromethanesulfonate, DCE: 1,2-dichloroethane, DCM: dichloromethane, DMAP: 4-(dimethylamino)pyridine, DMF: *N*,*N*-dimethylformamide, DMSO-d_6: deuterated dimethylsulfoxide, equiv.: equivalent, EtOAc: ethyl acetate, Fmoc: 9-fluorenylmethoxycarbonyl, MeOH: methanol, MS: molecular sieves, Nap: 2-naphthylmethyl, Py: pyridine, PTFACl: (*N*-phenyl)trifluoroacetimidoyl chloride, rt: room temperature, recd.: recovered, satd.: saturated, TCA: trichloroacetyl, TEMPO: 2,2,6,6-tetramethyl-piperidine-1-oxyl, theo.: theoretical, THF: tetrahydrofuran, Tol: toluene, TLC: thin layer chromatography, TMSOTf: trimethylsilyl trifluoromethanesulfonate, TfOH: trifluoromethanesulfonic acid, TBDPS: *tert*-butyldiphenylsilyl, TCP: tetrachlorophthaloyl, TCPO: tetrachlorophthalic anhydride, wrt: with respect to, $Yb(OTf)_3$: ytterbium(III) triflate.

II. Supplementary schemes

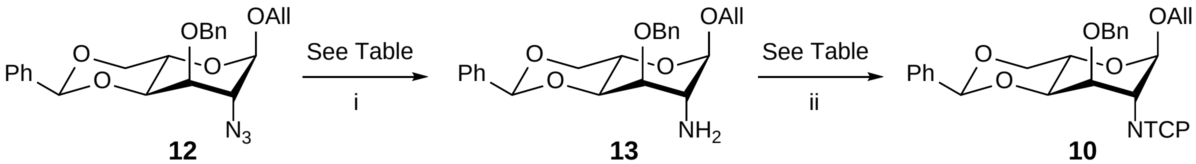

**Entry**[a]	**Conditions**	**Yield**[b] (%)
1	i. PPh_3 (1.1 equiv.), THF, H_2O, 60 °C<br>ii. TCPO (2.2 equiv.), Et_3N, DCM, rt	-
2	i. PPh_3 (1.1 equiv.), THF/H_2O, 60 °C<br>ii. TCPO (1.5 equiv.), Et_3N, DCE, 50 °C<br>then Ac_2O (5.0 equiv.), Py, rt, 10 h	30
3	i. PPh_3 (1.1 equiv.), THF/H_2O, 60 °C<br>ii. TCPO (1.5 equiv.), DCE, Et_3N, rt, 2 h<br>then Ac_2O (5.0 equiv.), Py, 90 °C, 10 min	57
4	i. Zn (12.0 equiv.), AcOH (12.0 equiv.), THF, rt<br>ii. TCPO (1.2 equiv.), DCE, Et_3N, rt, 2 h<br>then Ac_2O (5.0 equiv.), Py, 90 °C, 10 min	**83**

[a] Reactions were performed on the 2-10 g scale. Follow up was by TLC analysis. [b] Isolated yields.

Scheme S1. Conversion of the 2-azido intermediate **12** into its 2-*N*-TCP counterpart **10**.

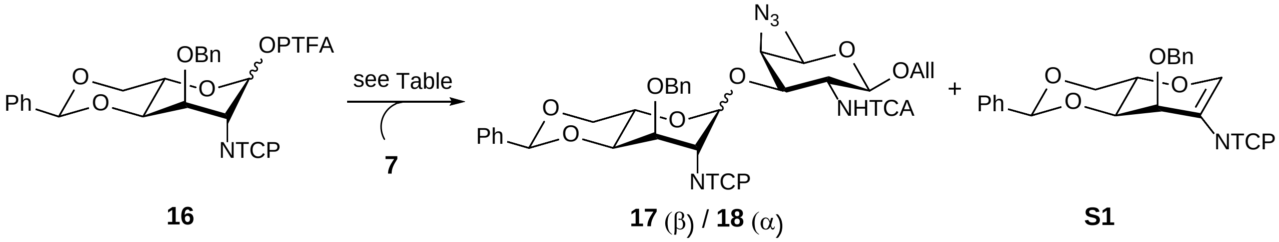

**Entries**[a]	**Scale (g)**	**Conditions**[b]	**17** (%)	**18** (%)	**S2** (%)
1	0.5	-30 °C	80	15	15
2	2.0	-30 °C	55	-	30
3	1.0	0 °C	56	-	56 (wrt **16**)

[a] Isolated yields. [b] **16** (1.25 equiv.), TMSOTf (0.05 equiv.), DCE 0.1 M, 30 min. wrt: with respect to.

Scheme S2. Glycosylation of PTFA donor **16** and acceptor **8**.

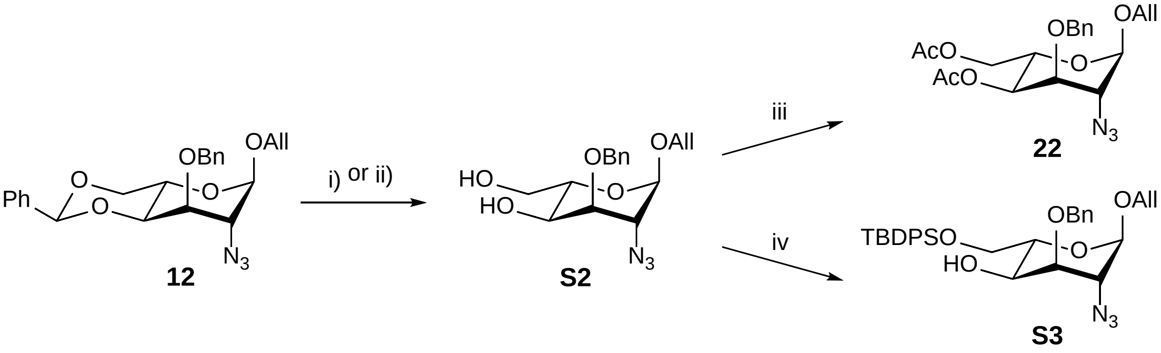

Scheme S3. Protecting group manipulation from altroside **12** by means of diol **S2**. i. 80% aq. AcOH, 80 °C, 2 h; ii. CSA, MeOH/DCM (4:1), rt, 2 h; iii) Ac_2O, Py, rt, 3 h, 88% from **12**; iv. TBDPSCl, Imidazole, DMF, rt, overnight.

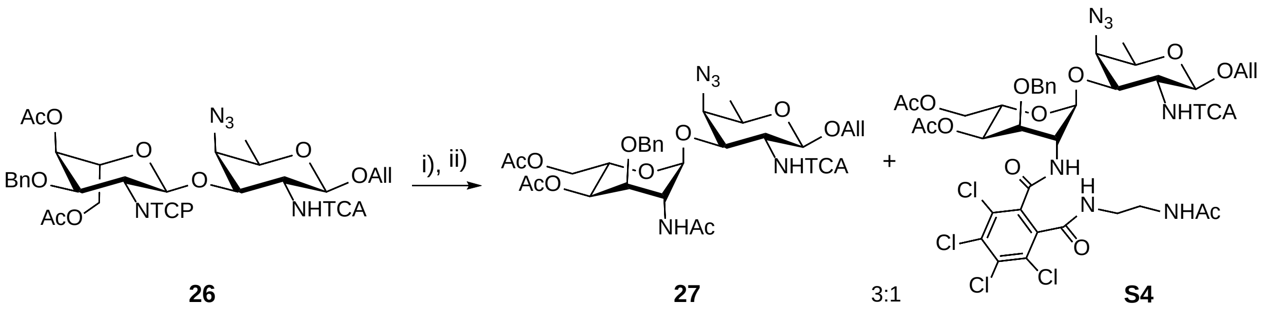

Scheme S4. Conversion of the 2A-*N*-TCP disaccharide **26** into the corresponding 2A-acetamido disaccharide **27**. i. $H_2NCH_2CH_2NH_2$, THF/MeOH, 72 h, 50 °C, ii. Ac_2O, Py, 3 h, 65% for **27** and 21% for **S4**.

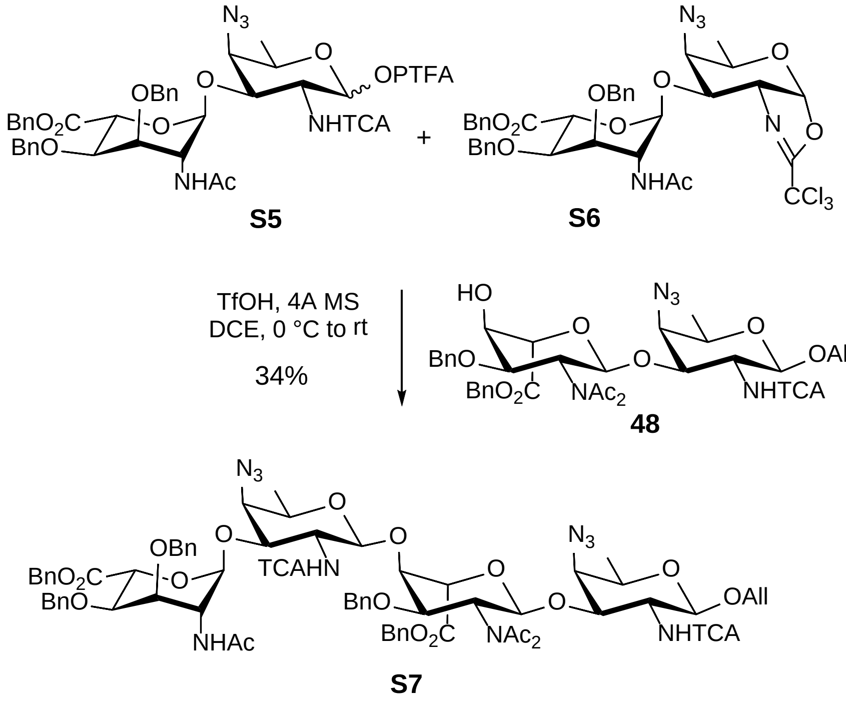

Scheme S5. Glycosylation of the 2A-NAc2 acceptor **48** and the 2A-NHAc donors **S5/S6**.

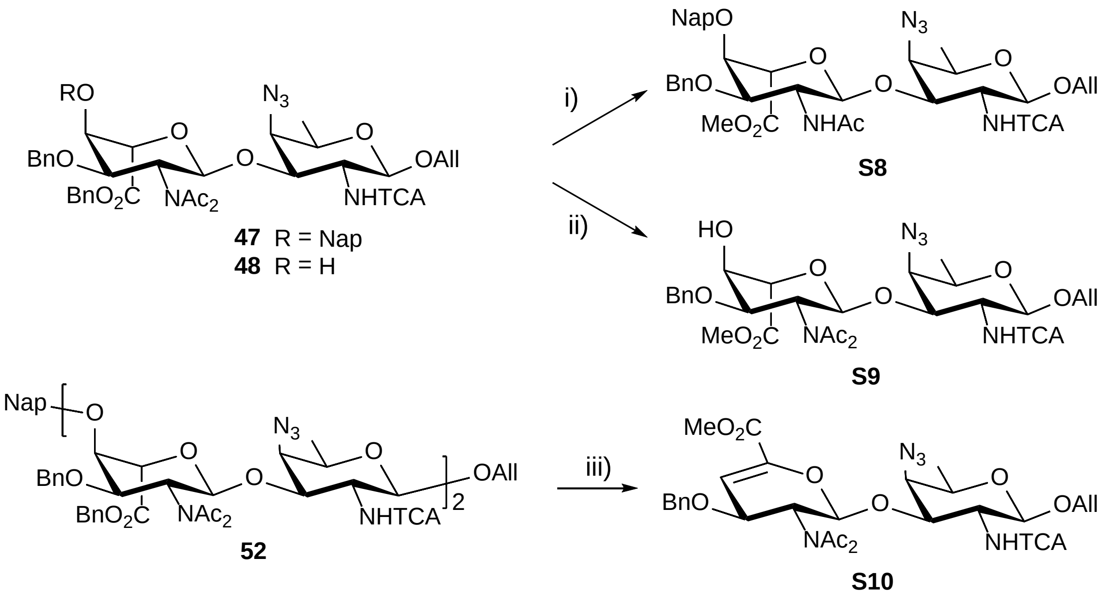

Scheme S6. Unmasking of the 2_A-NHAc in 2_A-NAc_2/2_B-NTCA oligosaccharides: model study on disaccharides **47**, and **48**, and on tetrasaccharide **52**. (i) from **47**: NaOMe, MeOH, rt, 74%, (ii) from **48**: K_2CO_3, MeOH, rt, 82%, (iii) K_2CO_3, MeOH, rt, 63%.

III. Supplementary tables

Table S1. Study on the glycosylation of donor **39/40** and acceptor **29** to give tetrasaccharide **42**.

Entry[a]	Glycosylation conditions	Products[b]
1	TMSOTf (0.05 equiv.), DCM, 0 °C to rt, 6 h	-
1	TfOH (0.1 equiv.), DCM, rt, 6 h	-
2	TfOH (0.1 equiv.), DCM, reflux, 2 h	**42** (Trace)
3	TfOH (0.2 equiv.), DCM, reflux, 2 h	degradation
4	TfOH (0.1 equiv.), DCE, ACN, 70 °C, 1 h	**38**
5	$Yb(OTf)_3$ (0.1 equiv.), DCM, rt, 8 h	-
6	$Yb(OTf)_3$ (0.2 equiv.), DCM, rt, 3 h	**42** (Trace)
7	$Yb(OTf)_3$ (0.2 equiv.), DCE, 70 °C, 1 h	**42** (Trace)
8	$Cu(OTf)_2$ (0.2 equiv.), DCE, 70 °C, 1 h	**42** (Trace)

[a] Reactions were performed on the 50 mg scale using **29** (1.1 equiv.). Follow up was by TLC analysis. [b] Based on LC-MS analysis.

Table S2. Investigation on the coupling of donor **43** and acceptor **29**.

**Entry**[a]	**29/43**	**Conditions**	**Observations**
1	1:2	TMSOTf (0.06 equiv.), DCM, -20 °C, 1 h	degradation
2	1:3	TfOH (0.1 equiv.), DCM, 0 °C, 1 h	
3	1:3	$Yb(OTf)_3$ (0.1 equiv.), DCM, rt, 1 h	

[a] Reactions were performed on the 50 mg scale. Follow up was by TLC and LC-MS analysis.

Table S3. $^{1}J_{C,H}$ and $^{3}J_{H,H}$ coupling constants (Hz) of the conformationally flexible A residue.

**Entry**	**Compound**	$^{3}J_{1,2}/^{1}J_{C,H}$	$^{3}J_{2,3}$	$^{3}J_{3,4}$	$^{3}J_{4,5}$
1[1]	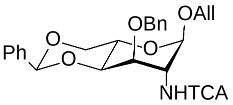	<1/169	2.7	3.0	9.9
2[1]	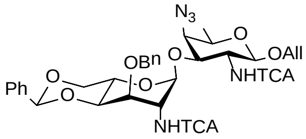	<1/169	2.7	2.9	9.6
3	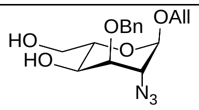 **S2**	2.4/170	3.5	-	8.4
4[1]	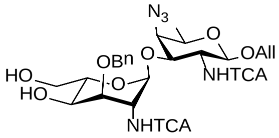	4.0/172	7.3	4.1	-
5	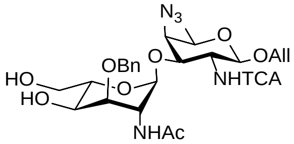 **28**	1.7/170	3.9	4.1	9.2
6	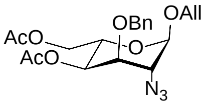 **22**	4.4/169	8.3	3.6	-

7	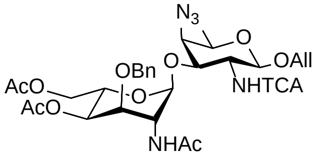 **27**	2.2/170	5.3	3.4	8.2
8	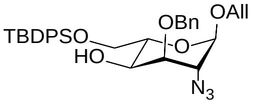 **S3**	4.2/169	7.3	3.9	6.7
9	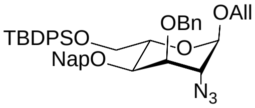 **30**	4.7/170	8.0	3.5	5.3
10	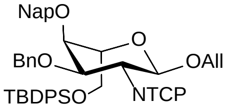 **31**	7.0/169	11.2	3.6	3.3
11	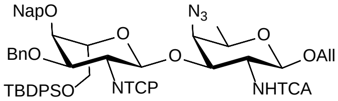 **34**	7.2/171	11.1	3.5	3.3
12	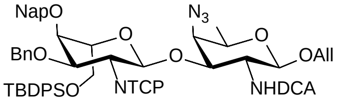 **62**	7.2/170	11.1	3.5	3.2
13	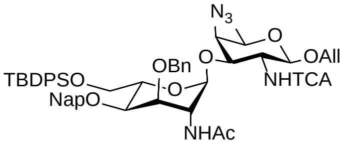 **35**	1.8/168	4.3	3.0	8.9
14	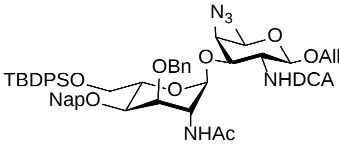 **63**	3.8/170	3.6	3.6	8.8
15	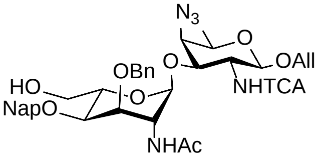 **36**	1.6/171	4.5	3.4	8.9

16	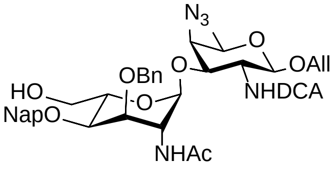<br>**64**	<1/170	4.0	-	-
17[1]	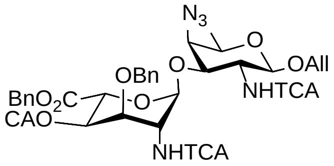	5.2/170	-	2.5	3.2
18	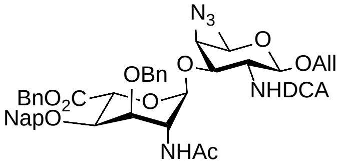<br>**65**	6.0/170	-	-	4.0
19	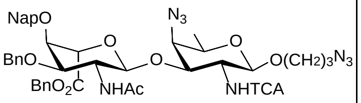<br>**41**	5.9/171	8.2	2.8	4.8
20	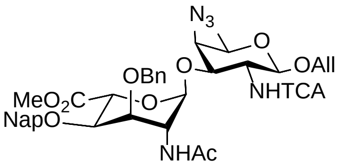<br>**S8**	4.0/170	-	-	3.6
21	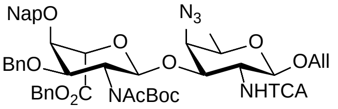<br>**57**	8.2/174	10.4	2.8	2.6
22	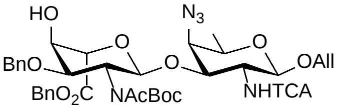<br>**58**	8.0/174	-	-	2.6
23	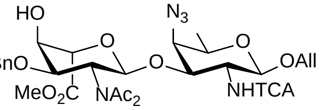<br>**S9**	7.8/175	10.8	3.2	2.0

IV. Experimental Protocols and analytical data

IVa. General procedures

Anhyd. solvents including Tol, DCM, DCE, THF, DMF, MeOH, ACN, and Py, were delivered on MS and used as received. Reactions requiring anhyd. conditions were run under an Ar atmosphere, using dried glassware. 4Å MS were activated before use by heating under high vacuum. Analytical TLC was performed with silica gel 60 F254, 0.25 mm pre-coated TLC aluminium foil plates. Compounds were visualized using UV254 and/or orcinol (1 mg·mL^{-1}) in 10% aq. H_2SO_4 with charring. Flash column chromatography was carried out using silica gel (particle size 40-63 µm). RP-HPLC purification was carried out using a Kromasil 5 µm C18 100 Å 10 × 250 mm semi-preparative column eluting with ACN in 0.08% aq. TFA. Analytical RP-HPLC of the final compounds (λ = 215 nm) was carried out using a Kromasil 3.5 µm C18 100 Å 3 × 150 mm analytical column, eluting with a 0-20% linear gradient of ACN in 0.08% aq. TFA over 20 min at a flow rate of 0.4 mL·min^{-1} (conditions A) or 1.0 mL·min^{-1} (conditions A'), a 0-20% linear gradient of ACN in 10 mM aq. ammonium acetate over 20 min at a flow rate of 0.4 mL·min^{-1} (conditions B), or using a an Aeris Peptide 3.5 µm C18 100 Å 2.1 × 150 mm analytical column, eluting with a 0-20% linear gradient of ACN in 0.08% aq. TFA over 20 min at a flow rate of 0.3 mL·min^{-1} (conditions C). Except for octasaccharide **4**, NMR spectra were recorded at 303 K on a Bruker Avance spectrometer equipped with a BBO probe at 400 MHz (^{1}H) and 100 MHz (^{13}C). Spectra were recorded in $CDCl_3$, DMSO-*d6* and D_2O. In the case of octasaccharide **4**, NMR spectra were recorded on a 800 MHz Bruker Avance NEO equipped with a high sensitivity TCI cryogenic probe. Chemical shifts are reported in ppm (*δ*) relative to the residual solvent peak in the case of $CDCl_3$ and DMSO-*d6*, and to HOD and DSS (4,4-dimethyl-4-silapentane-1-sulfonic acid) in the case of D_2O, at 7.28/77.0, 2.50/39.0 and 4.70/0.00 ppm for the ^{1}H and ^{13}C spectra, respectively. Coupling constants are reported in hertz (Hz). Elucidations of chemical structures were based on ^{1}H, COSY, DEPT-135, ^{13}C, HSQC, HMBC, HSQCND and NOESY spectra. Signals are reported as s (singlet), d (doublet), t (triplet), dd (doublet of doublet), q (quadruplet), dt (doublet of triplet), dq (doublet of quartet), ddd (doublet of doublet of doublet), m (multiplet). Signals can also be described as broad (prefix br), or partially overlapped (suffix po). Of the two magnetically non-equivalent geminal protons at C-6, the one resonating at lower field is denoted H-6a, and the one at higher field is denoted H-6b. Sugar residues are lettered according to the lettering of the repeating unit of the *S. sonnei* O-Ag and identified by a subscript (A, B) in the listing of signal assignments. For compounds made of multiple repeating units, residues are distinguished in the form of A/B, Ai/Bi, with A/B corresponding to the repeating unit at the reducing end. HRMS spectra were recorded on a WATERS QTOF Micromass instrument in the positive-ion electrospray ionisation (ESI^{+}) mode. Solutions were prepared using 1:1 ACN/H_2O containing 0.1% formic acid. In the case of sensitive compounds, solutions were prepared using 1:1 MeOH/H_2O to which was added 10 mM ammonium acetate.

General procedure for anomeric deallylation

$[Ir(COD)(PMePh_2)_2]PF_6$ (0.02 equiv.) was dissolved in anhyd. THF (20 mM) and stirred for 30-40 min under an H_2 atmosphere. The resulting yellow solution was degased several times with Ar and poured into a solution of allyl glycoside (1.0 equiv.) in anhyd. THF (50-100 mM). After stirring at rt for 1-2 h, NIS (1.1 equiv.) and H_2O, to reach a 1:5 H_2O/THF ratio, were added. After stirring at rt for 1 h, the reaction was quenched by addition of 10% aq. sodium sulphite. The reaction mixture was concentrated and the aq. phase was extracted with DCM. The combined organic layer was washed with brine, dried over anhyd. Na_2SO_4 and concentrated. Purification by flash chromatography (cHex/EtOAc) yielded the expected hemiacetal as a α/β mixture.

General procedure for the synthesis of the PTFA glycosyl donors

The hemiacetal precursor (1.0 equiv.) was dissolved in acetone (0.2 M). PTFACl (1.3 equiv.) was added followed by addition of Cs_2CO_3 (1.1 equiv.). After stirring at rt under an Ar atmosphere until completion (estimated ~2 h), the reaction mixture was filtered over a plug of Celite and washed exhaustively with anhydr. DCM. The filtrate was concentrated under reduced pressure. The crude residue was used as such in the glycosylation reaction. Purification by flash chromatography (cHex/EtOAc containing 1% Et_3N) provided analytical samples.

IVb. Compounds 1, 2, 9-11, 14-19, 21-29, 38-41, 57-65, S1-S4, and S7-S10

Allyl 4-azido-2-dichloroacetamido-2,4,6-trideoxy-β-D-galactopyranoside (9). $LiOH.H_2O$ (304 mg, 7.2 mmol, 3.0 equiv.) was added to the fully protected **8** (1.0 g, 2.4 mmol, 1.0 equiv.) in acetone/water (3:1, 24 mL). After stirring for 2 h at 50 °C, a TLC analysis (Tol/EtOAc 1:2) indicated the total consumption of the starting material (R_f 0.8) and the presence of a more polar product (R_f 0.0). The reaction mixture was concentrated under reduced pressure. The crude was passed through a short silica gel column eluting with 95:5 DCM/MeOH to give the intermediate amino alcohol after extensive drying under high vacuum. The latter had HRMS (ESI$^+$): *m/z* $[M+H]^+$ calcd for $C_9H_{16}N_4O_3$ 229.1301; found 229.1302.

Et_3N (0.5 mL, 3.6 mmol, 1.5 equiv.) was added to a solution of the crude amino alcohol in anhyd. ACN (10 mL), then cooled to 0 °C. Dichloroacetyl chloride (391 µL, 2.6 mmol, 1.1 equiv.) was added slowly and after stirring at this temperature for 30 min, a follow up by TLC (Tol/EtOAc 7:3) indicated the total consumption of the amino alcohol and the presence of a less polar product (R_f 0.2). EtOAc (30 mL) and water (20 mL) were added and the organic layer was separated, dried over anhyd. Na_2SO_4, filtered, and concentrated under reduced pressure. The crude residue was purified by flash chromatography (Tol/EtOAc 7:3→6:4) to give the desired dichloroacetamide **9** (560 mg, 1.65 mmol, 68%) as a white solid. Acceptor **9** had ^{1}H NMR (DMSO-d_6) δ 8.40 (d, 1H, $J_{NH,2}$ = 9.2 Hz, NH), 6.38 (s, 1H, $CHCl_2$), 5.81-5.76 (m, 1H, CH_{All}), 5.63 (d, 1H, J = 4.8 Hz, OH), 5.24-5.20 (m, 1H, CH_{2All}), 5.10-5.07 (m, 1H, CH_{2All}), 4.36 (d, 1H, $J_{1,2}$ = 8.4 Hz, H-1), 4.18-4.13 (m, 1H, CH_{2All}), 3.96-3.88 (m, 2H, H-3, CH_{2All}), 3.76 (dd, 1H, $J_{3,4}$ = 4.4 Hz, $J_{4,5}$ < 1.0 Hz, H-4), 3.67-3.62 (m_{po}, 1H, H-5, H-2), 1.20 (d, 3H, $J_{5,6}$ = 6.4 Hz, H-6). ^{13}C NMR (DMSO-d_6) δ 164.1

(CO_{NHDCA}), 134.9 (CH_{2All}), 116.7 (CH_{2All}), 100.5 (C-1, $^1J_{C,H}$ = 161 Hz), 70.8 (C-3), 69.2 (CH_{2All}), 68.7 (C-5), 67.6 ($CHCl_2$), 66.2 (C-4), 53.3 (C-2), 17.7 (C-6). HRMS (ESI$^+$): *m/z* [M+Na]$^+$ calcd for $C_{11}H_{16}Cl_2N_4O_4Na$ 361.0446; found 361.0442.

Allyl 3-*O*-benzyl-4,6-*O*-benzylidene-2-deoxy-2-tetrachlorophthalimido-α-L-altropyranoside (10). The known azide **12**[1] (10.0 g, 23.6 mmol, 1.0 equiv.) was dissolved in anhyd. THF (95 mL). Zn dust (12.3 g, 189 mmol, 8.0 equiv.) and AcOH (10.8 mL, 189 mmol, 8.0 equiv.) were added at rt. The reaction mixture was stirred vigorously for 1 h. A TLC follow up (Tol/EtOAc 1:1) showed the formation of a higly polar product and the full consumption of the starting **12** (R_f 0.9). The suspension was filtered through a pad of Celite and washed with DCM (100 mL) twice. The filtrate was washed with satd. aq. $NaHCO_3$. The organic layer was dried over anhyd. Na_2SO_4, filtered, and concentrated under reduced pressure. The residue, corresponding to the known amine **13**[1] was dried under high vacuum for 2 h. The crude material (9.6 g) was subjected to the next step.

Tetrachlorophthalic anhydride (4.05 g, 14.1 mmol, 0.6 equiv.) was added to the crude **13** (9.38 g, 23.6 mmol theo.) stirred in anhyd. DCM (100 mL) at rt under an Ar atmosphere. After 30 min, Et_3N (3.2 mL, 23.6 mmol, 1.0 equiv.) followed by more TCPO (4.05 g, 14.1 mmol, 0.6 equiv.) were added. The reaction mixture was stirred for another 30 min at rt, at which time a TLC follow up (EtOAc) revealed the presence of a polar product (R_f 0.0) and absence of **13** (R_f 0.15). Volatiles were eliminated under reduced pressure and the residue was dried under high vacuum for 1 h. The crude was dissolved in anhyd. Py (90 mL) and Ac_2O (11.1 mL, 118 mmol, 5.0 equiv.) was added at rt. The mixture was heated to 80 °C for 10 min, at which time a TLC follow up indicated completion. Volatiles were eliminated under reduced pressure and coevaporated with toluene (40 mL) twice. The crude was diluted with DCM (200 mL) and washed with 1N aq. HCl (300 mL), satd. aq. $NaHCO_3$ (300 mL) and brine (250 mL). The DCM layer was dried over Na_2SO_4, filtered and concentrated. The crude was purified by flash chromatography (cHex/EtOAc, 98:2 to 90:10) to give the fully protected **10** (13.0 g, 18.8 mmol, 83%) as a dense yellowish oil. Allyl glycoside **10** had R_f 0.65 (Tol/EtOAc 10:1). ^{1}H NMR ($CDCl_3$) δ 7.53-7.05 (m, 10H, H_{Ar}), 5.88-5.80 (m, 1H, CH_{All}), 5.65 (s, 1H, H_{Bzl}), 5.28-5.23 (m, 1H, CH_{2All}), 5.16-5.12 (m_{po}, 1H, CH_{2All}), 5.14 (d_{po}, 1H, $J_{1,2}$ = 4.0 Hz, H-1), 4.85 (d, 1H, J = 12.5 Hz, CH_{2Bn}), 3.92 (t, 1H, $J_{2,3}$ = 4.0 Hz, H-2), 4.71 (d, 1H, CH_{2Bn}), 4.52-4.42 (m, 2H, H-5, H-6a), 4.34 (dd_{po}, 1H, $J_{3,4}$ = 4.4 Hz, $J_{4,5}$ = 9.6 Hz, H-4), 4.27-4.22 (m, 1H, CH_{2All}), 4.10 (t, 1H, H-3), 4.03-3.97 (m, 1H, CH_{2All}), 3.88 (t, 1H, $J_{5,6b}$ = $J_{6a,6b}$ = 10.0 Hz, H-6b).^{13}C NMR ($CDCl_3$), δ 162.3 (CO_{NTCP}), 140.3, 138.1, 137.6, 137.6 ($C_{q,\ Ar}$), 133.6 (CH_{All}), 129.8, 129.0, 128.2, 128.0 (2C), 127.2, 126.9, 126.4 (C_{Ar}), 117.3 (CH_{2All}), 101.8 (C_{Bzl}), 95.4 (C-1, $^1J_{C,H}$ = 171 Hz), 76.4 (C-4), 73.1 (C-3), 72.8 (CH_{2Bn}), 69.7 (C-6), 68.4 (CH_{2All}), 59.8 (C-5), 55.5 (C-2). HRMS (ESI$^+$): *m/z* [M+Na]$^+$ calcd for $C_{31}H_{25}Cl_4NO_7Na$ 686.0283; found 686.0284.

Allyl 3-*O*-benzyl-4,6-*O*-benzylidene-2-deoxy-2-*N*-(9-fluorenylmethoxycarbonyl)-α-L-altropyranoside (11). A solution of azide **12** (2.4 g, 5.6 mmol, 1.0 equiv.) in THF (30 mL) was added triphenylphosphine (1.63 g, 6.2 mmol, 1.1 equiv.) and H_2O (3.0 mL, 169 mmol, 30 equiv.). The reaction mixture was stirred overnight at 60 °C, cooled to rt, concentrated under reduced pressure and coevaporated with toluene (10 mL) twice. The crude amine **13** in anhyd. DCM (28

mL) was stirred over freshly activated MS 4Å for 30 min at rt under an Ar atmosphere. After cooling to 0 °C, $NaHCO_3$ (953 mg, 11.3 mmol, 2.0 equiv.), DMAP (69 mg, 567 µmol, 0.1 equiv.) and FmocCl (1.7g, 6.8 mmol, 1.2 equiv.) were added. After stiring for 1 h at this temperature, a TLC analysis (cHex/EtOAc 9:1) revealed the conversion of intermediate **13** (R_f 0.45) into a less polar product (R_f 0.7). The reaction mixture was filtered and solids were washed with DCM (20 mL) twice. The filtrate was concentrated *in vacuo* and the residue was purified by flash chromatography using cHex/EtOAc (20:1→15:1) to give the Fmoc derivative **11** (3.1 g, 5.0 mmol, 88%) as a white solid. The fully protected **11** had ^{1}H NMR ($CDCl_3$) δ 7.81-7.78 (m, 2H, H_{Ar}), 7.65-7.60 (m, 2H, H_{Ar}), 7.51-7.26 (m, 15H, H_{Ar}), 5.98-5.89 (m, 1H, CH_{All}), 5.57 (s, 1H, H_{Bzl}), 5.37-5.31 (m, 1H, CH_{2All}), 5.22-5.20 (m, 1H, CH_{2All}), 4.96 (d, $J_{2,NH}$ = 8.8 Hz, NH), 4.88 (d, 1H, J = 12.6 Hz, CH_{2Bn}), 4.83 (d, 1H, CH_{2Bn}), 4.73 (s, 1H, H-1), 4.59-4.48 (m, 3H, H-5, CH_{2Fmoc}), 4.32 (dd, 1H, $J_{5,6a}$ = 5.6 Hz, $J_{6a,6b}$ = 10.4 Hz, H-6a), 4.29-4.21 (m, 3H, CH_{Fmoc}, H-2, CH_{2All}), 4.04-3.99 (m, 1H, CH_{2All}), 3.90 (brs, 1H, H-3), 3.73 (t_{po}, 1H, $J_{5,6b}$ = 10.4 Hz, H-6b), 3.70 (brd_{po}, 1H, $J_{4,5}$ = 8.8 Hz, H-4). ^{13}C NMR ($CDCl_3$) δ 155.2 (CO_{NHFmoc}), 144.3 143.6, 141.5, 141.4, 138.7, 137.6 ($C_{q,Ar}$), 133.6 (CH_{All}), 129.0, 128.2, 128.0, 127.8, 127.5, 127.3, 127.1, 127.0, 126.2, 124.7, 120.0 (C_{Ar}), 117.2 (CH_{2All}), 102.3 (C_{Bzl}), 98.9 (C-1, $^1J_{C,H}$ = 170 Hz), 77.0 (C-4), 74.2 (C-3), 72.1 (CH_{2Bn}), 69.2 (C-6), 68.4, (CH_{2All}), 66.6 (CH_{2Fmoc}), 58.7 (C-5), 52.1 (C-2), 47.3 (CH_{Fmoc}). HRMS (ESI$^+$): *m/z* $[M+Na]^+$ calcd for $C_{38}H_{37}NO_7Na$ 642.2468; found 642.2464.

3-*O*-Benzyl-4,6-*O*-benzylidene-2-deoxy-2-tetrachlorophthalimido-α/β-L-altropyranose (14). *Route 1.* $[Ir(COD)(PMePh_2)_2]PF_6$ (38 mg, 45 µmol, 0.02 equiv.) was dissolved in anhyd. THF (5.0 mL) and the red solution was stirred for 30 min under an H_2 atmosphere. The resulting light yellow solution was degassed several times with Ar and poured into a solution of allyl glycoside **10** (1.5 g, 2.26 mmol, 1.0 equiv.) in anhyd. THF (25 mL). After stirring at rt for 2 h, a TLC follow up (cHex/EtOAc; 15:1) showed the absence of the starting **10** (R_f 0.3) and the presence of a less polar spot (R_f 0.35). Iodine (1.14 g, 6.1 mmol, 1.05 equiv.) immediately followed by $NaHCO_3$ (571 mg, 6.78 mmol, 3.0 eqv.) in water (5 mL) were added. After stirring at rt for 1 h, the reaction was quenched by addition of 10% aq. Na_2SO_3. Volatiles were evaporated and the aq. layer was extracted with DCM (100 mL) twice. Purification by flash chromatography using cHex/EtOAc (10:1→8:1) gave the expected hemiacetal **14** (610 mg, 0.98 mmol, 43%) as a white floppy solid corresponding mainly to a single anomer. The later had R_f 0.15 (Tol/EtOAc; 10:1). ^{1}H NMR ($CDCl_3$) δ 7.55-7.52 (m, 2H, H_{Ar}), 7.44-7.38 (m, 3H, H_{Ar}), 7.11-7.09 (m, 2H, H_{Ar}), 6.94 (t, 2H, J = 7.6 Hz, H_{Ar}), 6.78-6.74 (m, 1H, H_{Ar}), 6.35 (dd, 1H, $J_{1,OH}$ = 8.8 Hz, $J_{1,2}$ = 4.8 Hz, H-1), 5.61 (s, 1H, H_{Bzl}), 4.84 (d, 1H, J = 12.4 Hz, CH_{2Bn}), 4.46 (dd, 1H, $J_{5,6a}$ = 5.2 Hz, $J_{6a,6b}$ = 10.4 Hz, H-6a), 4.39 (d, 1H, CH_{2Bn}), 4.30-4.24 (m, 2H, H-3, H-5), 4.02 (dd, 1H, $J_{2,3}$ = 3.2 Hz, H-2), 3.87-3.81 (m, 3H, H-6b, H-4, OH).^{13}C NMR ($CDCl_3$), δ162.9 (CO_{NTCP}), 139.6, 137.7, 137.3 ($C_{q,Ar}$), 129.4, 129.1, 128.9, 128.3, 127.9, 127.0 (2C), 126.1 (C_{Ar}), 102.0 (C_{Bzl}), 91.1 (C-1, $^1J_{C,H}$ = 176 Hz), 79.3 (C-4), 74.0 (CH_{2Bn}), 73.9 (C-3), 69.0 (C-6), 64.4 (C-5), 58.4 (C-2). HRMS (ESI$^+$): *m/z* $[M+Na]^+$ calcd for $C_{28}H_{21}Cl_4NO_7Na$ 645.9970; found 645.9964.

3-*O*-Benzyl-4,6-*O*-benzylidene-2-deoxy-2-*N*-(9-fluorenylmethoxycarbonyl)-α/β-L-altropyranose (15). Ir(COD)(PMePh$_2$)$_2$]PF$_6$ (71 mg, 84 μmol, 0.02 equiv.) in anhyd. THF (5.0 mL) was stirred under an H_2 atmosphere for 1 h at rt. The resulting yellow solution was degassed several times with Ar and transferred by use of a cannula into a solution of allyl glycoside **11** (2.6 g, 4.1 mmol, 1.0 equiv.) in anhyd. THF (40 mL). After stirring at rt for 1 h, NIS (1.03 g, 4.6 mmol, 1.1 equiv.) and H_2O (8 mL) were added and the reaction mixture was stirred for an additional hour. At completion, the reaction was quenched by addition of 10% aq. Na_2SO_3, and volatiles were eliminated. The aq. phase was extracted with DCM (40 mL) twice. The combined organic phases were dried over Na_2SO_4, filtered and concentrated under reduced pressure. Purification of the residue by flash chromatography using Tol/EtOAc (5:1→4:1) yielded the expected **15** (2.2 g, 3.7 mmol, 90%) as a white solid. Hemiacetal **15** was isolated as a 7:3 α/β mix and had R$_f$ 0.2 (Tol/EtOAc, 4:1). ^{1}H NMR (Partial assignment, CDCl$_3$) δ 7.79-7.77 (m, 2.7H, H$_{Ar}$), 7.76-7.62 (m, 2.6H, NH, H$_{Ar}$), 7.54-7.51 (m, 2.7H, H$_{Ar}$), 7.43-7.28 (m, 19.5H, H$_{Ar}$), 7.22-7.18 (m, 5.0H, H$_{Ar}$), 5.60 (s, 0.4H, H$_{Bzl,β}$), 5.57 (s, 1H, H$_{Bzl,α}$), 5.35-5.30 (m, 1H, H-1$_α$), 5.01-4.90 (m, 1.9H, H-1β, CH$_{2Bnα}$, CH$_{2Bnβ}$), 4.78-4.75 (m, 1.4H, CH$_{2Bnα}$, CH$_{2Bnβ}$), 4.58-4.55 (m, 2.6H, CH$_{2Fmoc,αβ}$), 4.34-4.30 (m, 1H, H-6a$_α$), 4.26-4.13 (m, 5H, H-2$_α$, H-2$_β$, H-3$_α$, H-3$_β$, H-5$_α$, H-5$_β$, CH$_{Fmoc,α}$, CH$_{Fmoc,β}$), 3.83-3.69 (m, 2.9H, H-4$_α$, H-4$_β$, H-6b$_β$), 3.62 (brs, 0.9H, OH$_{αβ}$). ^{13}C NMR (CDCl$_3$), δ 156.4 (CO$_{Fmoc}$), 143.6, 143.5, 141.4, 141.3, 138.3, 137.8, 137.4, 137.0 (C$_{q,Ar}$), 129.2, 129.1, 129.0, 128.5, 128.3 (2C), 128.2, 128.0, 127.8, 127.6, 127.1, 126.1 (2C), 125.3, 124.9, 124.8, 124.7, 120.0 (C$_{Ar}$), 102.6 (C$_{Bzl}$), 102.3 (C$_{Bzl}$), 94.7 (C-1$_β$, $^1J_{C,H}$ = 175.6 Hz), 92.0 (C-1$_α$, $^1J_{C,H}$ = 170.6 Hz), 77.3 (C-4$_α$, C-4$_β$), 75.3 (C-3$_α$), 75.3 (C-3$_β$), 74.5 (CH$_{2Bn, β}$), 73.5 (CH$_{2Bn, α}$), 69.3 (C-6$_α$), 68.9 (C-6$_β$), 66.8 (CH$_{2Fmoc,β}$), 66.8 (CH$_{2Fmoc,α}$), 64.5 (C-5$_α$, C-5$_β$), 54.6 (C-2$_α$), 52.7 (C-2$_β$), 47.3 (CH$_{Fmoc,α}$, CH$_{Fmoc,β}$). HRMS (ESI$^+$): *m/z* [M+Na]$^+$ calcd for $C_{35}H_{33}NO_7Na$ 602.2155; found 602.2153.

3-*O*-Benzyl-4,6-*O*-benzylidene-2-deoxy-2-tetrachlorophthalimido-α/β-L-altropyranosyl (*N*-phenyl)trifluoroacetimidate (16). Hemiacetal **14** (2.85 g, 4.2 mmol, 1.0 equiv.) was dissolved in acetone (40 mL) and PTFACl (855 μL, 5.5 mmol, 1.3 equiv.) was added followed by addition of Cs_2CO_3 (1.68 g, 5.1 mmol, 1.1 equiv.). After stirring for 2 h at rt, a TLC follow (Tol/EtOAc 10:1) showed the presence of a less polar spot (R$_f$ 0.7) and only traces of hemiacetal **14**. The reaction mixture was filtered over a pad of Celite and washed with acetone (10 mL) twice. The filtrate was concentrated under reduced pressure and the residue was purified by flash chromatography (cHex/EtOAc 95:5→93:7 containing 1% Et_3N) to give donor **16** as a white solid (2.75 g, 3.46 mmol, 80%). The latter, obtained as a 10:3 mixture of two anomers, had R$_f$ 0.3 (cHex/EtOAc 10:1). ^{1}H NMR (partial assignment, CDCl$_3$) *δ* 7.76 (brs, 0.8H, H$_{Ar}$), 7.59 (d, 0.8H, *J* = 7.6 Hz, H$_{Ar}$), 7.52-7.08 (m, 18.4H, H$_{Ar}$), 6.98 (d, 0.4H, *J* = 7.2 Hz, H$_{Ar}$), 6.82 (d, 1.6H, *J* = 7.2 Hz, H$_{Ar}$), 5.50 (s, 0.8H, CH$_{Bzl}$), 5.24 (s, 0.2H, CH$_{Bzl}$), 5.01-4.96 (m, 1.8H), 4.76-4.66 (m, 1.3H), 4.35 (dd, 0.8H, *J* = 5.2 Hz, *J* = 10.8 Hz), 4.25 (dd, 0.4H), 3.94 (t, 0.8H, *J* = 8.0 Hz, *J* = 8.4 Hz), 3.87-3.81 (m, 0.8H), 3.79-3.71 (m, 0.4H), 3.64-3.51 (m, 0.8H), 3.46-3.43 (m, 0.8H). HRMS (ESI$^+$): *m/z* [M+Na]$^+$ calcd for $C_{36}H_{25}Cl_4F_3N_2O_7Na$ 817.0266; found 817.0242.

Allyl 3-*O*-benzyl-4,6-*O*-benzylidene-2-deoxy-2-tetrachlorophthalimido-β-L-altropyranosyl-(1→3)-4-azido-2-trichloroacetamido-2,4,6-trideoxy-β-D-galactopyranoside (17), Allyl 3-*O*-benzyl-4,6-*O*-benzylidene-2-deoxy-2-tetrachlorophthalimido-α-L-altropyranosyl-(1→3)-4-azido-2-trichloroacetamido-2,4,6-trideoxy-β-D-galactopyranoside (18) and 3-*O*-Benzyl-4,6-*O*-benzylidene-2-deoxy-2-tetrachlorophthalimido-L-altral (S1). A mixture of acceptor **8** (600 mg, 1.61 mmol, 1.0 equiv.) and PTFA donor **16** (1.4 g, 1.77 mmol, 1.1 equiv.) in anhyd. DCE (20 mL) was stirred with freshly activated 4 Å MS (1.0 g) for 30 min at rt under an Ar atmosphere. The reaction mixture was cooled to -20 °C and TMSOTf (19 µL, 10 µmol, 0.06 equiv.) was added slowly. After stirring for 30 min at -20 °C, at which time a TLC follow up indicated donor consumption, Et_3N was added, the suspension was filtered and the fitrate was concentrated *in vacuo*. Flash chromatography using cHex/EtOAc (15:1→10:1) gave the unwanted β-isomer **17** (960 mg, 0.98 mmol, 61%) as a white solid, along with the desired α-isomer **18** (150 mg, 0.15 µmol, 10%), and the elimination product **S1** (220 mg, 0.36 mmol, 20% wrt **16**). The β-isomer **17** had R_f 0.35 (cHex/EtOAc 10:1). ^1H NMR ($CDCl_3$) *δ* 7.53-7.39 (m, 5H, H_{Ar}), 7.09 (dd, 2H, J = 1.0, 8.0 Hz, H_{Ar}), 6.95 (t, 2H, J = 7.6 Hz, H_{Ar}), 6.79-6.73 (m, 2H, H_{Ar}, NH), 6.20 (d, 1H, $J_{1,2}$ = 8.5 Hz, H-1_A), 5.90-5.80 (m, 1H, CH_{All}), 5.58 (s, 1H, H_{Bzl}), 5.28-5.23 (m, 1H, CH_{2All}), 5.18-5.14 (m, 1H, CH_{2All}), 4.82 (d, 1H, J = 12.5 Hz, CH_{2Bn}), 4.77 (d, 1H, $J_{1,2}$ = 8.4 Hz, H-1_B), 4.45 (dd, 1H, $J_{5,6a}$ = 5.1 Hz, $J_{6a,6b}$ = 10.2 Hz, H-6_{aA}), 4.41 (dd_{po}, 1H, $J_{3,4}$ = 3.4 Hz, $J_{2,3}$ = 10.9 Hz, H-3_B), 4.40 (d_{po}, 1H, CH_{2Bn}), 4.35-4.30 (m, 1H, CH_{2All}), 4.28 (brt, H-3_A), 4.24 (ddd, 1H, $J_{4,5}$ = 9.6 Hz, $J_{5,6}$ = 4.8 Hz, H-5_A), 4.22 (dd, 1H, $J_{2,3}$ = 3.1 Hz, H-2_A), 4.07-4.02 (m, 1H, CH_{2All}), 3.97 (brd, 1H, H-4_B), 3.86 (t, 1H, $J_{5,6b}$ = 10.4 Hz, H-6_{bA}), 3.80 (dd, 1H, $J_{4,5}$ = 9.6 Hz, $J_{3,4}$ = 2.3 Hz, H-4_A), 3.72-3.64 (m, 2H, H-2_B, H-5_B), 1.36 (d, 3H, $J_{5,6}$ = 6.4 Hz, H-6_B). ^{13}C NMR ($CDCl_3$) *δ* 163.8 (CO_{NHTCA}), 162.7, 161.6 (2C, CO_{NTCP}), 139.9, 139.5, 137.3, 137.1 ($C_{q, Ar}$), 133.5 (CH_{All}), 129.6, 129.3, 129.2, 128.9, 128.3, 128.0, 127.7, 127.1, 126.2, 126.1 (C_{Ar}), 117.7 (CH_{2All}), 102.0 (C_{Bzl}), 98.8 (C-1_B, $^1J_{C,H}$ = 161 Hz), 96.0 (C-1_A, $^1J_{C,H}$ = 173 Hz), 92.8 (CCl_3), 79.2 (C-4_A), 76.9 (C-3_B), 74.6 (CH_{2Bn}), 73.9 (C-3_A), 69.9 (CH_{2All}), 69.3 (C-5_B), 69.2 (C-6_A), 64.5 (C-5_A), 63.5 (C-4_B), 57.1 (C-2_A), 53.7 (C-2_B), 17.4 (C-6_B). HRMS (ESI^+): *m/z* $[M+Na]^+$ calcd for $C_{39}H_{34}Cl_7N_5O_{10}Na$ 1000.0023; found 1000.0015.

The α-isomer **18** had R_f 0.3 (cHex/EtOAc 10:1). ^1H NMR ($CDCl_3$) *δ* 7.54-7.51 (m, 2H, H_{Ar}), 7.48-7.37 (m, 3H, H_{Ar}), 7.28 (d, 2H, J = 6.8 Hz, H_{Ar}), 7.12 (t_{po}, 3H, J = 8.0 Hz, H_{Ar}), 6.97 (t_{po}, 1H, J = 7.2 Hz, H_{Ar}), 6.79 (d, 1H, $J_{2,NH}$ = 6.4 Hz, NH_B), 5.86-5.76 (m, 1H, CH_{All}), 5.63 (s, 1H, H_{Bzl}), 5.35 (d, 1H, $J_{1,2}$ = 5.6 Hz, H-1_A), 5.24-5.18 (m, 1H, CH_{2All}), 5.16-5.12 (m, 1H, CH_{2All}), 4.82 (d, 1H, J = 12.0 Hz, CH_{2Bn}), 4.81 (d, 1H, $J_{1,2}$ = 8.0 Hz, H-1_B), 4.74 (dd, 1H, $J_{2,3}$ = 4.8, Hz, H-2_A), 4.64 (dd, 1H, $J_{3,4}$ = 3.6 Hz, $J_{2,3}$ = 10.8 Hz, H-3_B), 4.58-4.46 (m, 2H, H-5_A, CH_{2Bn}), 4.50 (dd, 1H, $J_{5,6a}$ = 5.2 Hz, $J_{6a,6b}$ = 10.4 Hz, H-$6a_A$), 4.32-4.27 (m, 1H, CH_{2All}), 4.21 (dd, 1H, $J_{3,4}$ = 4.8 Hz, $J_{4,5}$ = 8.8 Hz, H-4_A), 4.12 (brt, 1H, H-3_A), 4.02-3.97 (m, 1H, CH_{2All}), 3.89 (brd, 1H, $J_{3,4}$ = 2.8 Hz, H-4_B), 3.85 (t, 1H, $J_{5,6b}$ = 10.1 Hz, H-$6b_A$), 3.71 (dq_{po}, 1H, H-5_B), 3.53-3.46 (m, 1H, H-2_B), 1.39 (d, 3H, $J_{5,6}$ = 6.4 Hz, H-6_B). ^{13}C NMR ($CDCl_3$) *δ* 162.5 (CO_{NHTCA}), 161.7 (CO_{NTCP}), 140.3, 137.9, 137.3 ($C_{q,Ar}$), 133.4 (CH_{All}), 129.9, 129.1, 128.4, 128.2, 127.9, 127.1, 126.9, 126.2 (C_{Ar}), 118.0 (CH_{2All}), 101.7 (C_{Bzl}), 97.7 (C-1_A, $^1J_{C,H}$ = 173 Hz), 97.3 (C-1_B, $^1J_{C,H}$ = 162 Hz), 92.1 (CCl_3,), 76.3 (C-4_A),

76.1 (C-3$_B$), 73.0 (CH_{2Bn}), 72.0 (C-3$_A$), 70.2 (CH_{2All}), 69.6 (C-6$_A$), 69.1 (C-5$_B$), 65.8 (C-4$_B$), 60.8 (C-5$_A$), 55.7 (C-2$_A$), 55.7 (C-2$_B$), 17.5 (C-6$_B$). HRMS (ESI$^+$): *m/z* $[M+NH_4]^+$ calcd for $C_{39}H_{38}Cl_7N_6O_{10}$ 995.0469; found 995.0437.

Altral **S1** had R_f 0.65 (cHex/EtOAc 10:1). ^{1}H NMR ($CDCl_3$) δ 7.58-7.56 (m, 2H, H_{Ar}), 7.45-7.40 (m, 3H, H_{Ar}), 7.15 (d$_{po}$, 2H, J = 7.2 Hz, H_{Ar}), 6.96-6.92 (m$_{po}$, 2H, H_{Ar}), 6.79-6.75 (m, 1H, H_{Ar}), 6.61 (s, 1H, H-1), 5.67 (s, 1H, H_{Bzl}), 4.83 (d, 1H, J = 12.6 Hz, CH_{2Bn}), 4.60 (dd, 1H, $J_{5,6a}$ = 5.3 Hz, $J_{6a,6b}$ = 10.4 Hz, H-6a), 4.50 (dt, 1H, $J_{5,6b}$ = 10.3 Hz, $J_{4,5}$ = 5.3 Hz, H-5), 4.39 (d, 1H, $J_{3,4}$ = 5.3 Hz, H-3), 4.38 (d, 1H, CH_{2Bn}), 4.21 (dd, 1H, H-4), 3.96 (t, 1H, H-6b).^{13}C NMR ($CDCl_3$) δ 162.5 (CO_{NTCP}), 147.3 (C-1), 139.9, 138.6, 137.2 ($C_{q,Ar}$), 129.7, 129.2, 128.7, 128.3, 127.9, 127.1, 126.9, 126.2 (C_{Ar}), 108.4 (C-2), 101.7 (C_{Bzl}), 77.9 (C-4), 74.0 (CH_{2Bn}), 68.3 (C-6), 67.6 (C-3), 65.1 (C-5). HRMS (ESI$^+$): *m/z* $[M+NH_4]^+$ calcd for $C_{28}H_{23}Cl_4N_2O_6$ 623.0310; found 623.0295.

3-*O*-Benzyl-4,6-*O*-benzylidene-2-deoxy-2-*N*-(9-fluorenylmethoxycarbonyl)-α/β-L-altropyranosyl (*N*-phenyl)trifluoroacetimidate (19). Hemiacetal **14** (170 mg, 293 µmol, 1.1 equiv.) was dissolved in acetone (6 mL). PTFACl (60 µL, 382 µmol, 1.3 equiv.) was added followed by addition of Cs_2CO_3 (105 mg, 323 µmol, 1.1 equiv.). After stirring at rt for 1 h, a TLC analysis indicated reaction completion. The reaction mixture was filtered over a pad of Celite, and solids were washed with DCM (5 mL) twice. Volatiles were evaporated under reduced pressure and the residue was purified by flash chromatography eluting with cHex/EtOAc (98:2→90:10) to give the desired **19** (200 mg, 273 µmol, 90%) as an off-white solid. The constrained PTFA donor had R_f 0.8 (Tol/EtOAc 9:1). ^{1}H NMR (α anomer, $CDCl_3$) δ 7.68-7.50 (m, 18H, H_{Ar}), 5.67 (d, 1H, J = 3.6 Hz, H-1), 5.50 (s, 1H, H_{Bzl}), 4.67 (brs, 1H, CH_{2Fmoc}), 4.54 (brs, 1H, CH_{2Bn}), 4.50 (dd$_{po}$, 1H, CH_{2Fmoc}), 437-4.33 (m, 1H, H-6a), 4.27-4.10 (m, 3H, H-5, H-3, CH_{Fmoc}), 3.70-3.58 (m, 2H, H-6b, H-2) 3.60 (dd$_{po}$, 1H, $J_{3,4}$ < 1.0 Hz, $J_{4,5}$ = 8.8 Hz, H-4). ^{13}C NMR ($CDCl_3$) δ 153.4 (CO_{NFmoc}), 141.3, 139.7, 137.8, 137.4 ($C_{q,Ar}$), 129.4, 129.1, 128.5, 128.3, 128.2 (2C), 127.8, 127.7, 127.6, 127.5, 127.3, 127.2, 127.0, 126.3, 126.1, 124.6, 124.3, 122.5, 120.4, 120.3, 120.2 (C_{Ar}), 102.6 (C_{Bzl}), 95.8 (C-1, $^1J_{C,H}$ = 182 Hz), 75.7 (C-4$_α$), 73.7 (CH_{2Bn}), 71.6 (C-3), 69.0 (C-6), 67.2 (CH_{2Fmoc}), 63.7 (C-5$_α$), 61.2 (C-2$_α$), 47.0 (CH_{Fmoc}). HRMS (ESI$^+$): *m/z* $[M+H]^+$ calcd for $C_{43}H_{38}F_3N_2O_7$ 751.2631; found 751.2665.

Allyl 3-*O*-benzyl-4,6-*O*-benzylidene-2-deoxy-2-*N*-(9-fluorenylmethoxycarbonyl)-α-L-altropyranosyl-(1→3)-4-azido-2-trichloroacetamido-2,4,6-trideoxy-β-D-galactopyranoside (21). Hemiacetal **15** (343 mg, 59 µmol, 1.1 equiv.) was dissolved in acetone (10 mL) and cooled to 0 °C. Trichloroacetonitrile (855 µL, 2.36 mmol, 4.0 equiv.) was added followed by addition of K_2CO_3 (163 mg, 1.18 mmol, 2.0 equiv.). After stirring for 4 h at 0 °C, the reaction mixture was filtered over a pad of Celite, washed with DCM (5 mL) twice. The filtrate was concentrated under reduced pressure to give the crude trichloroacetimidate **20**.

The crude **20** (1.1 equiv. theo) was mixed with acceptor **8** (200 mg, 538 µmol, 1.0 equiv.), coeveporated with toluene repeatedly, and dried under high vacuum for 2 h. The mixture was dissolved in anhyd. DCM (10 mL) and stirred with freshly activated MS 4Å (500 mg) for 45 min

under an Ar atmosphere before the temperature was set to -15 °C. TMSOTf (7 µL, 30 µmol, 0.05 equiv.) was added slowly. After stirring at this temperature for 30 min, a TLC analysis (Tol/EtOAc 3:1) showed the presence of a new spot (R_f 0.4) close to those featuring acceptor **8** (R_f 0.35) and hemiacetal **15** (R_f 0.32). Et_3N (~10 µL) was added and the suspension was filtered over a fitted funnel. The filtrate was concentrated under reduced pressure and the residue was purified by flash chromatography eluting with Tol/EtOAc (5:1→4:1) to give the desired disaccharide **21** (200 mg, 214 µmol, 62%). The coupling product had ^1H NMR ($CDCl_3$) δ 7.80-7.77 (m, 2H, H_{Ar}), 7.59-7.57 (d_{po}, 2H, H_{Ar}), 7.52-7.29 (m, 15H, H_{Ar}), 6.90 (d, $J_{2,NH}$ = 6.2 Hz, NH_B), 5.90-5.80 (m, 1H, CH_{All}), 5.56 (s, 1H, CH_{Bzl}), 5.29-5.24 (m, 1H, CH_{2All}), 5.20-5.17 (m, 1H, CH_{2All}), 4.99 (d, 1H, $J_{1,2}$ = 8.0 Hz, H-1_B), 4.87 (d_{po}, 1H, J = 11.8 Hz, CH_{2Bn}), 4.84 (d_{po}, 1H, J = 7.2 Hz, NH_A), 4.80 (d_{po}, 1H, CH_{2Bn}), 4.77 (s_{po}, 1H, H-1_A), 4.64-4.58 (m, 2H, H-3_B, H-5_A), 4.52 (brd, 2H, J = 5.8 Hz, $CH_{2, NHFmoc}$), 4.36-4.31 (m, 2H, H-$6a_A$, CH_{2All}), 4.24 (brd, 1H, $J_{2,NH}$ = 7.2 Hz, H-2_A), 4.20 (t, 1H, J = 6.0 Hz, CH_{NHFmoc}), 4.09-4.04 (m, 1H, CH_{2All}), 3.87 (brs, 1H, H-3_A), 3.78 (brq, 1H, H-5_B), 3.72 (t, 1H, $J_{5,6b}$ = $J_{6a,6b}$ = 10.5 Hz, H-$6b_A$), 3.69 (brd_{po}, 1H, $J_{3,4}$ = 3.0 Hz, H-4_B), 3.67 (brd_{po}, 1H, $J_{4,5}$ = 8.0 Hz, H-4_A), 3.49-3.43 (m, 1H, H-2_B), 1.42 (d, 3H, $J_{5,6}$ = 6.4 Hz, H-6_B). ^{13}C NMR ($CDCl_3$) δ 162.2 (CO_{NTCA}), 154.9 (CO_{NFmoc}), 143.5, 141.4, 141.3, 138.9, 137.3 ($C_{q,Ar}$), 133.5 (CH_{All}), 129.1, 128.2, 128.0, 127.8, 127.4, 127.2, 127.1, 126.2, 124.8, 124.7, 120.0 (C_{Ar}), 117.9 (CH_{2All}), 102.3 (C_{Bzl}), 101.6 (C-1_A, $^1J_{C,H}$ = 169 Hz), 97.2 (C-1_B, $^1J_{C,H}$ = 162 Hz), 92.0 (CCl_3,), 76.7 (C-4_A), 75.6 (C-3_B), 74.0 (C-3_A), 72.3 (CH_{2Bn}), 70.2 (CH_{2All}), 69.7 (C-5_B), 69.1 (C-6_A), 66.6 (CH_{2Fmoc}), 65.9 (C-4_B), 59.7 (C-5_A), 56.1 (C-2_B), 52.4 (C-2_A), 47.3 (CH_{NHFmoc}), 17.4 (C-6_B). HRMS (ESI^+): *m/z* $[M+NH_4]^+$ calcd for $C_{46}H_{50}Cl_3N_6O_7$ 951.2654; found 951.2694.

Allyl 2-azido-3-*O*-benzyl-2-deoxy-α-L-altropyranoside (S2). Azide **12** (4.0 g, 9.4 mmol, 1.0 equiv.) was dissolved in AcOH (40 mL) and water (10 mL) was added. The solution was heated at 80 °C for 2 h. A TLC follow up (Tol/EtOAc 1:1) showed the absence of the starting material (R_f 0.9) and the presence of a new spot (R_f 0.1). After reaching rt, the reaction mixture was concentrated under vacuum. The residue was coevaporated with toluene (10 mL) twice and dried over vacuum to deliver the crude diol **S2**. The latter had R_f 0.45 (Tol/EtOAc 1:1). ^1H NMR ($CDCl_3$) δ 7.38-7.34 (m, 5H, H_{Ar}), 5.99-5.90 (m, 1H, CH_{All}), 5.36-5.31 (m, 1H, CH_{2All}), 5.25-5.22 (m, 1H, CH_{2All}), 4.83 (d_{po}, 1H, J = 11.2 Hz, CH_{2Bn}), 4.80 (d_{po}, 1H, $J_{1,2}$ = 2.4 Hz, H-1), 4.61 (d, 1H, CH_{2Bn}), 4.29-4.23 (m, 1H, CH_{2All}), 4.07-4.01 (m, 1H, CH_{2All}), 3.99 (ddd, 1H, $J_{4,5}$ = 8.4 Hz, H-5), 3.92-3.88 (m, 1H, H-4) 3.89 (dd_{po}, 1H, $J_{2,3}$ = 3.5 Hz, H-2), 3.86 (dd_{po}, 1H, $J_{5,6b}$ = 3.6 Hz, H-6a), 3.79 (dd_{po}, 1H, $J_{5,6a}$ = 5.2 Hz, $J_{6a,6b}$ = 12.3 Hz, H-6b), 3.78-3.75 (m_o, 1H, H-3). ^{13}C NMR ($CDCl_3$) δ 137.0 ($C_{q, Ar}$), 133.6 (CH_{All}), 129.0, 128.6, 128.2, 128.2, 128.0 (C_{Ar}), 117.4 (CH_{2All}), 97.9 (C-1, $^1J_{C,H}$ = 170 Hz), 76.2 (C-3), 72.5 (CH_{2Bn}), 70.8 (C-5), 68.7 (CH_{2All}), 64.7 (C-4), 62.9 (C-6), 59.6 (C-2). HRMS (ESI^+): *m/z* $[M+NH_4]^+$ calcd for $C_{16}H_{25}N_4O_5$ 353.1825; found 353.1827.

Allyl 4,6-di-*O*-acetyl-2-azido-3-*O*-benzyl-2-deoxy-α-L-altropyranoside (22). The crude diol **S2** (9.4 mmol theo., 1.0 equiv.) was dissolved in pyridine (40 mL) and acetic anhydride (2.2 mL, 23.6 mmol, 2.5 equiv.) was added at rt. After stirring for 3 h, a TLC analysis indicated reaction completion. Volatiles were evaporated and coevaporated with toluene (10 mL) twice, diluted with

DCM (150 mL). The organic phase was washed with 1N aq. HCl (150 mL), 50% aq. $NaHCO_3$ (150 mL) and brine (100 mL), dried over Na_2SO_4, filtered, and concentrated under vacuum. The crude was purified by flash chromatography eluting with Tol/EtOAc (4:1→3:1) to give diacetate **22** (3.5 g, 8.3 mmol, 88%) as a white solid. Azide **22** had R_f 0.4 (Tol/EtOAc 4:1). ^{1}H NMR ($CDCl_3$) δ 7.39-7.31 (m, 5H, H_{Ar}), 5.98-5.89 (m, 1H, CH_{All}), 5.37-5.31 (m, 1H, CH_{2All}), 5.26-5.22 (m, 2H, H-4, CH_{2All}), 4.72 (d, 1H, $J_{1,2}$ = 4.4 Hz, H-1), 4.67 (dd_{po}, 1H, J = 11.6 Hz, CH_{2Bn}), 4.29 (dd_{po}, 1H, $J_{5,6a}$ = 6.0 Hz, H-6a), 4.29-4.24 (m, 2H, H-5, CH_{2All}), 4.18-4.15 (m_{po}, 1H, H-6b), 4.09-4.04 (m, 1H, CH_{2All}), 3.81 (dd, 1H, $J_{2,3}$ = 8.3 Hz, H-2), 3.81 (dd, 1H, $J_{3,4}$ = 3.8 Hz, H-3), 2.10 (s, 3H, CH_{3Ac}), 2.08 (s, 3H, CH_{3Ac}). ^{13}C NMR ($CDCl_3$) δ 170.4, 170.0 (CO_{Ac}), 137.1 ($C_{q,Ar}$), 133.4 (CH_{All}), 128.4, 128.0 (C_{Ar}), 117.3 (CH_{2All}), 98.7 (C-1, $^1J_{C,H}$ = 169 Hz), 74.6 (C-3), 72.4 (CH_{2Bn}), 69.6 (C-5), 69.2 (CH_{2All}), 66.3 (C-4), 62.7 (C-6), 61.5 (C-2), 20.8 (CH_{3Ac}), 20.6 (CH_{3Ac}). HRMS (ESI$^+$): *m/z* $[M+Na]^+$ calcd for $C_{20}H_{25}N_3O_7Na$ 442.1590; found 442.1597.

Allyl 2-azido-3-*O*-benzyl-6-*O*-*tert*-butyldiphenylsilyl-2-deoxy-α-L-altropyranoside (S3). CSA (4.1 g, 17.7 mmol, 0.5 equiv.) was added to acetal **12** (15.0 g, 35.4 mmol, 1.0 equiv.) in MeOH/DCM (4:1, 170 mL). After stirring at rt for 2 h, a TLC follow up (Tol/EtOAc 4:1) indicated reaction completion. 5% Aq. $NaHCO_3$ (300 mL) was added followed by EtOAc (500 mL). The organic phase was separated, washed with brine (500 mL), dried over Na_2SO_4 and concentrated under reduced pressure. The material was dried under high vacuum to give the crude diol **S2** as a yellow oil. The latter was used as such in the next step.

tert-Butyldiphenylchlorosilane (10.1 mL, 38.9 mmol, 1.1 equiv.) and imidazole (3.1 g, 46.0 mmol, 1.3 equiv.) were added to diol **S2** in anhyd. DMF (180 mL) at 0 °C. The reaction mixture was allowed to reach rt slowly and stirred overnight at this temperature. MeOH (10.0 mL) was added and after 30 min, volatiles were evaporated under reduced pressure. The crude material was dissolved in EtOAc (500 mL) and the organic layer was washed with 90% aq. brine (500 mL), separated, dried over Na_2SO_4, and concentrated to give the crude silyl ether **S3**. The latter had R_f 0.65 (Tol/EtOAc 9:1). ^{1}H NMR ($CDCl_3$) δ 7.71-7.68 (m, 4H, H_{Ar}), 7.40-7.34 (m, 11H, H_{Ar}), 6.00-5.90 (m, 1H, CH_{All}), 5.35-5.29 (m, 1H, CH_{2All}), 5.23-5.19 (m, 1H, CH_{2All}), 4.84 (d, 1H, $J_{1,2}$ = 4.2 Hz, H-1), 4.80 (d, 1H, J = 11.4 Hz, CH_{2Bn}), 4.62 (d, 1H, CH_{2Bn}), 4.34-4.29 (m, 1H, CH_{2All}), 4.09-4.04 (m, 1H, CH_{2All}), 4.01 (ddd, 1H, H-5), 3.95 (ddd, 1H, $J_{4,5}$ = 6.7 Hz, H-4), 3.91 (dd, 1H, $J_{5,6b}$ = 3.3 Hz, $J_{6a,6b}$ = 11.2 Hz, H-6a), 3.85-3.80 (m, 2H, $J_{5,6b}$ = 5.2 Hz, H-6b, H-2), 3.77 (dd, 1H, $J_{2,3}$ = 7.3 Hz, $J_{3,4}$ = 3.9 Hz, H-3), 3.77 (dd, 1H, $J_{4,OH}$ = 5.2 Hz, OH-4). ^{13}C NMR ($CDCl_3$) δ 137.2, 135.7, 135.6 ($C_{q,Ar}$), 133.8 (CH_{All}), 133.2, 133.1, 129.7 (2C), 128.6, 128.1, 128.0, 127.7 (C_{Ar}), 117.3 (CH_{2All}), 98.2 (C-1, $^1J_{C,H}$ = 169 Hz), 76.9 (C-3), 73.4 (C-5), 72.6 (CH_{2Bn}), 68.6 (CH_{2All}), 65.2 (C-4), 64.5 (C-6), 60.8 (C-2), 26.8 ($CH_{3,TBDPS}$), 19.2 (C_{TBDPS}). HRMS (ESI$^+$): *m/z* $[M+NH_4]^+$ calcd for $C_{32}H_{43}N_4O_5Si$ 591.3003; found 591.2971.

Allyl 4,6-di-*O*-acetyl-3-*O*-benzyl-2-deoxy-2-tetrachlorophthalimido-α-L-altropyranoside (23). Zn dust (5.6 g, 85.8 mmol, 8.0 equiv.) and AcOH (4.9 mL, 85.8 mmol, 8.0 equiv.) were added to a solution of azide **22** (4.5 g, 10.7 mmol, 1.0 equiv.) in THF (70 mL) at rt. The reaction mixture was stirred for 2 h at which point a TLC follow up (Tol/EtOAc 1:1) revealed the absence of any

remaining **22** (R_f 0.8) and the presence of a more polar product. The mixture was filtered over a pad of Celite and solids were washed with DCM (100 mL) twice. The combined organic phases were washed with satd. aq. $NaHCO_3$ (200 mL) and brine (200 mL). The organic phase was dried over Na_2SO_4, filtered, and concentrated. The residue was dried under high vacuum for 3 h and subjected as such to the next step. The crude amine intermediate had HRMS (ESI$^+$): *m/z* [M+Na]$^+$ calcd for $C_{20}H_{28}NO_7$ 394.1866; found 394.1856.

Tetrachlorophthalic anhydride (3.68 g, 12.8 mmol, 1.2 equiv.) was added to a solution of the crude intermediate in DCM (40 mL) and the solution was stirred for 30 min at rt. Et_3N (1.79 mL, 12.8 mmol, 1.2 equiv.) was added and the reaction mixture was stirred for another 30 min. Volatiles were eliminated under reduced pressure and the residue was dried under high vacuum for 1 h. The crude material was dissolved in pyridine (50 mL) and Ac_2O (5.0 mL, 53.6 mmol, 5.0 equiv.) was added at 0 °C. The mixture was heated to 80 °C for 10 min. A TLC follow up (Tol/EtOAc 9:1) showed the formation of a product (R_f 0.7) slightly more polar than azide **22** (0.75). After it reached rt, the reaction mixture was concentrated and coevaporated with Toluene (15 mL) twice. The residue was diluted with DCM (100 mL) and washed with 1N aq. HCl (200 mL), satd. aq. $NaHCO_3$ (200 mL) and brine (200 mL). The DCM layer was dried over Na_2SO_4, filtered, concentrated and the residue was purified by flash chromatography eluting with cHex/EtOAc (12:1→9:1) to give diacetate **23** as a yellowish foam (6.0 g, 9.1 mmol, 85%). Compound **23** had R_f 0.35 (Tol/EtOAc 10:1). ^{1}H NMR ($CDCl_3$) δ 7.29-7.17 (m, 2H, H_{Ar}), 7.06-7.00 (m, 3H, H_{Ar}), 5.81-5.72 (m, 1H, CH_{All}), 5.50 (dd$_{po}$, 1H, H-4), 5.37 (d, 1H, J = 7.3 Hz, H-1), 5.22-5.17 (m, 1H, CH_{2All}), 5.13-5.09 (m, 1H, CH_{2All}), 4.61 (d, 1H, J = 12.4 Hz, CH_{2Bn}), 4.53 (dd, 1H, Hz, $J_{2,3}$ = 11.2 Hz, H-2), 4.44 (dd, 1H, $J_{3,4}$ = 4.3 Hz, H-3), 4.39 (dd, 1H, $J_{5,6a}$ = 6.6 Hz, $J_{6a,6b}$ = 11.7 Hz, H-6a), 4.37 (dd, 1H, $J_{5,6b}$ = 5.7 Hz, H-6b), 4.30 (dt$_{po}$, 1H, $J_{4,5}$ = 3.1 Hz, H-5), 4.23-4.18 (m, 1H, CH_{2All}), 4.20 (d$_{po}$, 1H, CH_{2Bn}), 4.01-3.96 (m, 1H, CH_{2All}), 2.21 (s, 3H, CH_{3Ac}), 2.17 (s, 3H, CH_{3Ac}). ^{13}C NMR ($CDCl_3$) δ 170.0 (CO_{Ac}), 162.9 (CO_{NTCP}), 139.9, 137.4, 129.6, 127.0, 125.2 ($C_{q,Ar}$), 133.4 (CH_{All}), 129.0, 128.2, 128.1, 127.6 (C_{Ar}), 117.7 (CH_{2All}), 95.4 (C-1, $^1J_{C,H}$ = 169 Hz), 72.7 (C-5), 72.1 (CH_{2Bn}), 71.1 (C-3), 69.3 (CH_{2All}), 68.8 (C-4), 62.9 (C-6), 53.2 (C-2). HRMS (ESI$^+$): *m/z* [M+NH$_4$]$^+$ calcd for $C_{28}H_{29}Cl_4N_2O_9$ 677.0627; found 677.0622.

4,6-Di-*O*-acetyl-3-*O*-benzyl-2-deoxy-2-tetrachlorophthalimido-α/β-L-altropyranose (24). [Ir(COD)(PMePh$_2$)$_2$]PF$_6$ (59 mg, 0.07 mmol, 0.02 equiv.) was stirred in anhyd. THF (5.0 mL) under an H_2 atmosphere at rt for 30 min. The resulting yellow solution was degassed several times with Ar and transferred by use of a cannula into a solution of allyl glycoside **23** (2.3 g, 3.4 mmol, 1.0 equiv.) in anhyd. THF (25 mL). After stirring at rt for 1h, NIS (864 mg, 3.8 mmol, 1.05 equiv.) and H_2O (5 mL) were added. After 2 h, a TLC analysis (Tol/EtOAc 4:1) revealed the presence of a compound (R_f 0.35) more polar that allyl glycoside **23** (R_f 0.65). 10% Aq. Na_2SO_3 was added and the reaction mixture was concentrated to remove the THF and the aq. phase was extracted with DCM (50 mL) thrice. The combined DCM phases were washed with brine, dried over Na_2SO_4, filtered, and concentrated under reduced pressure. The residue was purified by flash chromatography (Tol/EtOAc 8:1→6:1) to give the expected **24** (2.0 g, 3.2 mmol, 92%) as a white floppy solid (α/β ~5:1). Hemiacetal **24** (α anomer) had ^{1}H NMR (extracted, $CDCl_3$) δ 7.28-7.01

(m, 5H, H_{Ar}), 5.61 (t, 1H, $J_{1,2} = J_{1,OH}$ = 7.6 Hz, H-1), 5.60 (dd, 1H, H-4), 4.60 (d, 1H, J = 12.4 Hz, CH_{2Bn}), 4.53 (dd, 1H, $J_{3,4}$ = 3.8 Hz, $J_{2,3}$ = 11.1 Hz, H-3), 4.43-4.35 (m, 3H, H-2, H-6a, H-6b), 4.32 (ddd, 1H, $J_{4,5}$ = 2.1 Hz, $J_{5,6}$ = 5.1 Hz, $J_{5,6}$ = 7.1 Hz, H-5), 4.22 (d, 1H, CH_{2Bn}), 3.49 (d, 1H, OH). ^{13}C NMR ($CDCl_3$) δ 170.6, 170.4 (CO_{Ac}), 163.1 (CO_{NTCP}), 140.0, 137.8, 137.4 ($C_{q,Ar}$), 129.7, 129.0, 128.1 (2C), 127.7 (C_{Ar}), 128.3, 128.2, 127.8, 125.3, 90.4 (C-1$_{\alpha}$, $^{1}J_{C,H}$ = 170 Hz), 73.8 (C-3), 71.9 (CH_{2Bn}), 70.8 (C-5), 66.9 (C-4), 62.6 (C-6), 55.0 (C-2), 21.0, 20.8 (2C, CH_{3Ac}). HRMS (ESI$^+$): *m/z* $[M+NH_4]^+$ calcd for $C_{25}H_{25}Cl_4N_2O_9$ 637.0314; found 637.0336.

Hemiacetal **24** (β anomer) had ^{1}H NMR (extracted, $CDCl_3$) δ 7.28-7.01 (m, 5H, H_{Ar}), 5.64 (dd, 1H, $J_{3,4}$ = 2.0 Hz, $J_{4,5}$ = 2.8 Hz, H-4), 5.43 (t, 1H, $J_{1,2} = J_{1,OH}$ = 4.0 Hz, H-1), 5.21 (dd, 1H, $J_{5,6a}$ = 3.2 Hz, $J_{5,6b}$ = 11.2 Hz, H-5), 4.75 (dd, 1H, $J_{1,2}$ = 3.2 Hz, $J_{2,3}$ = 10.4 Hz, H-2), 4.69 (d, 1H, J = 10.8 Hz, CH_{2Bn}), 4.60 (d, 1H, J = 12.4 Hz, CH_{2Bn}), 4.54-4.49 (m_o, 2H, H-6a, H-6b), 4.43-4.35 (m_o, 1H, $CH_{2Bn,\beta}$), 4.21-4.17 (m_{po}, 1H, H-3), 4.11 (d, 1H, OH). ^{13}C NMR ($CDCl_3$), δ 170.9, 170.3 (CO_{Ac}), 163.8 (CO_{NTCP}), 140.2, 137.8 ($C_{q,Ar}$), 128.3, 128.2, 127.8, 125.2 (C_{Ar}), 93.1 (C-1$_{\alpha}$, $^{1}J_{C,H}$ = 175 Hz), 75.2 (C-3), 71.5 (CH_{2Bn}), 67.6 (C-5), 67.5 (C-4), 64.5 (C-6), 53.0 (C-2), 21.4, 21.0 (2C, CH_{3Ac}). HRMS (ESI$^+$): *m/z* $[M+NH_4]^+$ calcd for $C_{25}H_{25}Cl_4N_2O_9$ 637.0314; found 637.0336.

4,6-Di-*O*-acetyl-3-*O*-benzyl-2-deoxy-2-tetrachlorophthalimido-α/β-L-altropyranosyl (*N*-phenyl)trifluoroacetimidate (25). Hemiacetal **24** (1.5 g, 2.4 mmol, 1.0 equiv.) was dissolved in acetone (20 mL). PTFACl (580 µL, 3.6 mmol, 1.5 equiv.) was added followed by the addition of cesium carbonate (947 mg, 2.9 mmol, 1.2 equiv.). The reaction mixture was stirred at rt. After 2 h, a TLC analysis (Tol/EtOAc 6:1) showed the presence of a new compound (R_f 0.65) and the absence of hemiacetal **24** (R_f 0.2). The reaction mixture was filtered through a pad of Celite and solids were washed with acetone (10 mL) twice. The filtrate was concentrated and the residue was purified by flash chromatography (cHex/EtOAc 9:1) to give PTFA **25** as a white solid (1.8 g, 2.2 mmol, 94%). Donor **25** (α/β 3:2) had R_f 0.65 (cHex/EtOAc 9:1). ^{1}H NMR ($CDCl_3$) δ 7.29-6.99, 6.72, 6.54 (m, 14H, H_{Ar}), 6.54, (bs_o, 0.6H, H-1$_{\alpha}$), 6.43 (brs, 0.4H, H-1$_{\beta}$), 5.71 (d, 0.4H, $J_{4,5}$ = 2.4 Hz, H-4$_{\beta}$), 5.54 (dd, 0.6H, $J_{3,4}$ = 3.2 Hz, $J_{4,5}$ = 3.6 Hz, H-4$_{\alpha}$), 5.45 (dd, 0.4H, $J_{3,4}$ = 2.8 Hz, $J_{2,3}$ = 9.6 Hz, H-3$_{\beta}$), 4.97 (dd, 0.4H, $J_{1,2}$ = 3.6 Hz, H-2$_{\beta}$), 4.71 (d, 0.4H, J = 10.4 Hz, $CH_{2Bn\beta}$), 4.7 (dd, 0.6H, $J_{1,2}$ = 4.8 Hz, $J_{2,3}$ = 10.8 Hz, H-2$_{\alpha}$), 4.65 (d, 0.6H, J = 12.4 Hz, $CH_{2Bn\alpha}$), 4.60 (dd_{po}, 0.4H, $J_{6a,6b}$ = 8.0 Hz, $J_{5,6b}$ = 4.4 Hz, H-6a$_{\beta}$), 4.65 (d, 0.6H, $CH_{2Bn\alpha}$), 4.51-4.35 (m, 2.2H, H-5$_{\alpha}$, H-5$_{\beta}$, H-6a$_{\alpha}$, H-6b$_{\beta}$, H-3$_{\alpha}$), 4.33 (dd, 0.6H, $J_{6a,6b}$ = 12.4 Hz, $J_{5,6b}$ = 4.8 Hz, H-6b$_{\alpha}$), 4.23 (d, 0.6H, $CH_{2Bn\alpha}$), 2.23, 2.16 (2s, 1.8H, $CH_{3Ac\alpha}$), 2.21, 2.17 (2s, 1.2H, $CH_{3Ac\beta}$). ^{13}C NMR ($CDCl_3$) δ 170.4 (2C, $CO_{Ac,\beta}$), 170.3, 169.9 (2C, $CO_{Ac,\alpha}$), 163.2 ($CO_{TCP,\beta}$), 162.6 ($CO_{TCP,\alpha}$), 142.9, 142.5, 140.3, 137.8, 129.8, 126.9, 124.5 ($C_{Ar,\beta,q}$), 140.1, 137.2, 129.7, 126.9, 124.5 ($C_{Ar,\alpha,q}$), 129.0, 128.6 (2C), 128.3, 128.2, 128.1, 127.9, 127.7, 125.2, 124.5 (2C), 119.4, 119.1 ($C_{Ar,\beta}$, $C_{Ar,\alpha}$), 95.2 (C-1$_{\alpha}$, $^{1}J_{C,H}$ = 182 Hz, C-1$_{\beta}$, $^{1}J_{C,H}$ = 177 Hz), 76.3 (C-5$_{\beta}$), 74.2 (C-5$_{\alpha}$), 72.3 ($CH_{2Bn\alpha}$), 71.8 ($CH_{2Bn\beta}$), 70.8 (C-3$_{\beta}$), 67.5 (C-4$_{\alpha}$, C-4$_{\beta}$), 66.7 (C-3$_{\alpha}$), 63.3 (C-6$_{\beta}$), 63.2 (C-6$_{\alpha}$), 52.3(C-2$_{\alpha}$), 51.5 (C-2$_{\beta}$), 21.4, 20.9 ($CH_{3Ac\alpha}$), 20.8, 20.6 ($CH_{3Ac\beta}$). HRMS (ESI$^+$): *m/z* $[M+Na]^+$ calcd for $C_{33}H_{25}Cl_4F_3N_2O_9Na$ 813.0164; found 813.0175.

Allyl 4,6-di-*O*-acetyl-3-*O*-benzyl-2-deoxy-2-tetrachlorophthalimido-α-L-altropyranosyl-(1→3)-4-azido-2-trichloroacetamido-2,4,6-trideoxy-β-D-galactopyranoside (26). A mix of acceptor **8** (2.03 g, 5.45 mmol, 1.0 equiv.) and PTFA **25** (5.4 g, 6.54 mmol, 1.2 equiv.) was coevaporated with anhyd. toluene (20 mL), dried under high vaccum for 1 h, and then dissolved in anhyd. DCE (130 mL). Freshly activated MS 4Å (1.0 g) was added and after stirring for 45 min at rt under an Ar atmosphere, the reaction mixture was cooled to 0 °C and TMSOTf (59 µL, 327 µmmol, 0.05 equiv.) was added. After 20 min at 0 °C, a TLC follow up (Tol/EtOAc 3:1) showed a new spot (R_f 0.4) and no remaining acceptor **8** (R_f 0.35). Et_3N was added and the suspension was passed through a fitted funnel. Solids were washed DCM (30 mL) twice and the filtrate was concentrated under reduced pressure. Flash chromatography using Tol/EtOAc (8:1→6:1) yielded the desired disaccharide **26** (4.7 g, 4.83 mmol, 88%) as a white solid. Diacetate **26** had ^{1}H NMR ($CDCl_3$) δ 7.28-7.16 (m, 2H, H_{Ar}), 7.00 (brs, 3H, H_{Ar}), 6.67 (d, 1H, $J_{2,NH}$ = 6.8 Hz, NH_B), 5.83-5.74 (m, 1H, CH_{All}), 5.56 (d, 1H, $J_{1,2}$ = 7.6 Hz, H-1$_A$), 5.46 (dd, 1H, $J_{4,5}$ = 2.8 Hz, H-4$_A$), 5.22-5.16 (m, 1H, CH_{2All}), 5.17-5.10 (m, 1H, CH_{2All}), 4.75 (d, 1H, $J_{1,2}$ = 8.3 Hz, H-1$_B$), 4.58 (dd$_{po}$, 1H, $J_{2,3}$ = 10.8 Hz, $J_{3,4}$ = 3.6 Hz, H-3$_B$), 4.57 (d$_o$, 1H, CH_{2Bn}), 4.57 (dd$_{po}$, 1H, $J_{2,3}$ = 11.2 Hz, H-2$_A$), 4.41 (dd$_{po}$, 1H, $J_{3,4}$ = 3.9 Hz, H-3$_A$), 4.40-4.35 (m, 3H, H-5$_A$, H-6a$_A$, H-6b$_A$), 4.29-4.24 (m, 1H, CH_{2All}), 4.16 (d, 1H, J = 12.4 Hz, CH_{2Bn}), 4.00-3.95 (m, 1H, H-4$_B$, CH_{2All}), 3.68 (dq, $J_{4,5}$ = 1.0 Hz, H-5$_B$), 3.55 (ddd, 1H, $J_{2,3}$ = 11.0 Hz, H-2$_B$), 2.18 (s, 3H, CH_{3Ac}), 2.17 (s, 3H, CH_{3Ac}), 1.37 (d, 3H, $J_{5,6}$ = 6.4 Hz, H-6$_B$). ^{13}C NMR ($CDCl_3$) δ 170.5, 170.3 (CO_{Ac}), 162.9 (CO_{NHTCA}), 161.5 (CO_{NTCP}), 139.9, 137.8, 137.2 ($C_{q, Ar}$), 133.4 (CH_{All}), 129.0, 128.2, 128.1, 128.0, 127.6, 125.2 (C_{Ar}), 118.0 (CH_{2All}), 97.9 (C-1$_A$, $^1J_{C,H}$ = 171 Hz), 97.5 (C-1$_B$, $^1J_{C,H}$ = 162 Hz), 92.2 (CCl_3), 76.6 (C-3$_B$), 73.2 (C-3$_A$), 72.1 (CH_{2Bn}), 70.6 (C-5$_A$), 70.1 (CH_{2All}), 69.0 (C-5$_B$), 66.5 (C-4$_A$), 65.4 (C-4$_B$), 62.8 (C-6$_A$), 55.3 (C-2$_B$), 53.1 (C-2$_A$), 20.8 (2C, CH_{3Ac}), 17.5 (C-6$_B$). HRMS (ESI$^+$): *m/z* $[M+NH_4]^+$ calcd for $C_{36}H_{38}Cl_7N_6O_{12}$ 991.0367; found 991.0355.

Allyl (2-acetamido-4,6-di-*O*-acetyl-3-*O*-benzyl-2-deoxy-α-L-altropyranosyl)-(1→3)-4-azido-2-trichloroacetamido-2,4,6-trideoxy-β-D-galactopyranoside (27) and Allyl (4,6-di-*O*-acetyl-2-*N*-((2-acetamidoethyl)carbamoyl)-3,4,5,6-tetrachlorobenzoyl-3-*O*-benzyl-2-deoxy-α-L-altropyranosyl)-(1→3)-4-azido-2-trichloroacetamido-2,4,6-trideoxy-β-D-galactopyranoside (S4). Ethylenediamine (41 µL, 617 µmol, 4.0 equiv.) was added to disaccharide **24** (150 mg, 154 µmol, 1.0 equiv.) in n-butanol (8 mL) and the solution was heated at 70 °C for 72 h. A TLC follow up (Tol/EtOAc 4:1) indicated the absence of phtalimide **24** and after the reaction mixture reached rt, volatiles were eliminated and the residue was coevaporated with toluene (5 mL) twice. Acetic anhydride (0.15 mL, 1.5 mmol, 10 equiv.) was added to the residue in pyridine (4 mL) and the system was stirred at rt. In the absence of further evolution after for 4 h (TLC: Tol/EtOAc 4:1), volatiles were evaporated and the residue was purified by column chromatography eluting with Tol/EtOAc (80:20→70:30) to give **27** (76 mg, 101 µmol, 65%) as a white solid and side-product **S4** (33 mg, 31 µmol, 20%). The desired **27** had R_f 0.45 (Tol/EtOAc 4:1). ^{1}H NMR ($CDCl_3$) δ 7.41-7.30 (m, 5H, H_{Ar}), 6.67 (d, 1H, $J_{2,NH}$ = 8.3 Hz, NH_B), 5.83-5.74 (m, 1H, CH_{All}), 5.56 (d, 1H, $J_{1,2}$ = 7.6 Hz, NH_A), 5.29-5.24 (m, 1H, CH_{2All}), 5.20-5.17 (m, 1H, CH_{2All}), 5.02 (dd, 1H, $J_{3,4}$ = 3.4 Hz, $J_{4,5}$ = 8.2 Hz, H-4$_A$), 4.90 (d, 1H, $J_{1,2}$ = 2.2 Hz, H-1$_A$), 4.87 (d, 1H, $J_{1,2}$ = 8.4 Hz, H-1$_B$), 4.67 (d,

1H, J = 12.3 Hz, CH_{2Bn}), 4.64 (d, 1H, CH_{2Bn}), 4.59 (ddd$_{po}$, 1H, $J_{5,6a}$ = 5.6 Hz, H-5$_A$), 4.55 (dd$_{po}$, 1H, $J_{3,4}$ = 3.5 Hz, H-3$_B$), 4.37-4.31 (m$_{po}$, 1H, CH_{2All}), 4.44-4.34 (m, 2H, H-2$_A$, H-6a$_A$), 4.18 (dd, 1H, $J_{5,6b}$ = 2.9 Hz, $J_{6a,6b}$ = 12.0 Hz, H-6b$_A$), 4.09-4.04 (m, 1H, CH_{2All}), 3.91 (dd, 1H, $J_{2,3}$ = 5.3 Hz, H-3$_A$), 3.84 (brd, 1H, $J_{3,4}$ = 3.4 Hz, H-4$_B$), 3.77 (brq, $J_{4,5}$ = 1.0 Hz, H-5$_B$), 3.58 (ddd, 1H, $J_{2,3}$ = 10.8 Hz, H-2$_B$), 2.18, (s, 3H, CH_{3Ac}), 2.17 (s, 3H, CH_{3Ac}), 1.37 (d, 3H, $J_{5,6}$ = 6.4 Hz, H-6$_B$). ^{13}C NMR ($CDCl_3$) δ 170.5, 170.3 (2C, CO_{Ac}), 162.9 (CO_{NHTCA}), 161.5 (CO_{NTCP}), 139.9, 137.8, 137.2, ($C_{q, Ar}$), 133.4 (CH_{All}), 129.0, 128.2, 128.1, 128.0, 127.6, 125.2 (C_{Ar}), 118.0 (CH_{2All}), 97.9 (C-1$_A$, $^1J_{C,H}$ = 171 Hz), 97.5 (C-1$_B$, $^1J_{C,H}$ = 162 Hz), 92.2 (CCl_3), 76.6 (C-3$_B$), 73.2 (C-3$_A$), 72.1 (CH_{2Bn}), 70.6 (C-5$_A$), 70.1 (CH_{2All}), 69.0 (C-5$_B$), 66.5 (C-4$_A$), 65.4 (C-4$_B$), 62.8 (C-6$_A$), 55.3 (C-2$_B$), 53.1 (C-2$_A$), 20.8 (2C, CH_{3Ac}), 17.5 (C-6$_B$). HRMS (ESI$^+$): *m/z* [M+H]$^+$ calcd for $C_{30}H_{39}Cl_3N_5O_{11}$ 750.1711; found 750.1746.

The side-product **S4** had R$_f$ 0.25 (Tol/EtOAc 7:3). ^{1}H NMR ($CDCl_3$) δ 8.25 (d, 1H, $J_{2,NH}$ = 8.0 Hz, NH$_B$), 7.39-7.17 (m, 5H, H$_{Ar}$), 6.56 (d, 1H, J = 5.2 Hz, NH), 6.31 (d, 1H, $J_{2,NH}$ = 7.6 Hz, NH$_A$), 5.89-5.79 (m, 1H, CH_{All}), 5.28-5.22 (m, 1H, CH_{2All}), 5.22 (dd$_{po}$, 1H, $J_{3,4}$ = 2.5 Hz, $J_{4,5}$ = 5.2 Hz, H-4$_A$), 5.16-5.13 (m, 1H, CH_{2All}), 5.06 (d, 1H, $J_{1,2}$ = 5.6 Hz, H-1$_A$), 4.79 (d, 1H, $J_{1,2}$ = 8.0 Hz, H-1$_B$), 4.68 (d, 1H, J = 11.2 Hz, CH_{2Bn}), 4.62 (d, 1H, CH_{2Bn}), 4.48 (d, 1H, $J_{3,4}$ = 3.6 Hz, $J_{2,3}$ = 10.8 Hz, H-3$_B$), 4.44-4.31 (m, 3H, H-5$_A$, H-6a$_A$, CH_{2All}), 4.21 (brs, 2H, H-2$_A$, H-3$_A$), 4.15 (dd, 1H, $J_{5,6b}$= 3.2 Hz, $J_{6a,6b}$ = 11.2 Hz, H-6b$_A$), 4.09-4.04 (m, 1H, CH_{2All}), 3.95-3.90 (m, 1H, H-2$_B$), 3.90 (brd, 1H, $J_{3,4}$ = 3.6 Hz, H-4$_B$), 3.77 (brq$_{po}$, 1H, H-5$_B$), 3.58-3.47 (m, 4H, CH_2CH_2), 2.10, 2.07, 1.99 (3s, 9H, CH_{3NAc}), 1.40 (d, 3H, $J_{5,6}$ = 6.4 Hz, H-6$_B$). ^{13}C NMR ($CDCl_3$) δ 171.3 (CO_{NHAc}), 170.5 (CO_{NHAc}), 170.1 (CO_{Ac}), 164.5, 164.3 (CO_{NTCP}), 162.0 (CO_{NHTCA}), 137.3, 135.3, 135.2, 135.0, 134.2, 129.8, 129.7 ($C_{q,Ar}$), 133.7 (CH_{All}), 129.0, 128.3, 128.2, 127.9, 127.7 (C_{Ar}), 117.3 (CH_{2All}), 100.3 (C-1$_A$, $^1J_{C,H}$ = 169 Hz), 98.9 (C-1$_B$, $^1J_{C,H}$ = 163 Hz), 92.9 (CCl_3), 78.2 (C-3$_B$), 72.6 (C-3$_A$), 71.7 (CH_{2Bn}), 70.6 (C-5$_A$), 69.7 (CH_{2All}), 69.3 (C-5$_B$), 66.3 (C-4$_A$), 65.2 (C-4$_B$), 62.5 (C-6$_A$), 54.1 (C-2$_B$), 53.1 (C-2$_A$), 40.7 (OCH_2), 38.2 (OCH_2), 23.1, 20.8 (CH_{3Ac}, CH_{3NHAc}), 17.5 (C-6$_B$). HRMS (ESI$^+$): *m/z* [M+NH$_4$]$^+$ calcd for $C_{40}H_{48}Cl_7N_8O_{13}$ 1093.1161; found 1093.1177.

Allyl 2-acetamido-3-*O*-benzyl-2-deoxy-α-L-altropyranosyl-(1→3)-4-azido-2,4,6-trideoxy-2-trichloroacetamido-β-D-galactopyranoside (28). NaOMe (26 µL, 25% NaOMe in MeOH, 0.2 equiv.) was added to disaccharide **26** (500 mg, 514 µmol, 1.0 equiv.) in anhyd. methanol (15 mL). After stirring at rt for 1 h, a TLC follow up (Tol/EtOAc 1:2) indicated reaction completion and Dowex resin (H$^+$) was added pinch by pinch to reach pH 7. The suspension was filtered by passing through a fitted funnel and the filtrate was concentrated and dried under high vacuum. The obtained diol had (HRMS (ESI$^+$): *m/z* [M+NH$_4$]$^+$ calcd for $C_{32}H_{34}Cl_7N_6O_{10}$ 907.0156; found 907.0156)

Following extensive drying, the intermediate diol was dissolved in THF/MeOH (1:4, 15 mL). Ethylenediamine (137 µL, 2.0 mmol, 4.0 equiv.) was added and the mixture was heated at 70 °C for 48 h. At reaction completion (TLC: EtOAc), the reaction mixture was cooled down, concentrated under reduced pressure, and coevaporated with toluene (5 mL) twice. Et_3N (500 µL) and acetic anhydride (485 µL, 5.1 mmol, 10 equiv.) were added to the residue in methanol (10 mL). After stirring at rt for 2 h, a TLC follow up (EtOAc/MeOH 9:1) showed reaction completion. Volatiles were evaporated and the residue was purified by column chromatography (DCM/MeOH

95:5) to give the desired **28** (235 mg, 353 μmol, 69%) as a white solid. Diol **28** had R_f 0.2 (EtOAc). ^{1}H NMR (DMSO-d_6) δ 8.85 (d, 1H, J = 9.0 Hz, NH_B), 7.93 (d_{po}, 1H, J = 8.7 Hz, NH_A), 7.44-7.22 (m, 5H, H_{Ar}), 5.86-5.76 (m, 1H, CH_{All}), 5.26-5.21 (m, 1H, CH_{2All}), 5.12-5.09 (m, 1H, CH_{2All}), 4.79 (d, 1H, $J_{1,2}$ = 1.7 Hz, H-1_A), 4.65 (d, 1H, J = 12.0 Hz, CH_{2Bn}), 4.61-4.56 (m, 2H, OH), 4.51 (d_{po}, 1H, $J_{1,2}$ = 8.3 Hz, H-1_B), 4.50 (d_{po}, 1H, CH_{2Bn}), 4.27 (ddd, 1H, $J_{2,3}$ = 3.9 Hz, H-2_A), 4.22-4.17 (m, 1H, CH_{2All}), 4.11-4.07 (m_{po}, 1H, $J_{3,4}$ = 3.6 Hz, H-3_B), 4.09 (brs_o, 1H, H-4_B), 4.04 (ddd, 1H, $J_{4,5}$ = 9.2 Hz, $J_{5,6a}$ = 2.1 Hz, $J_{5,6b}$ = 7.1 Hz, H-5_A), 3.99-3.96 (m, 1H, CH_{2All}), 3.90-3.84 (m, 1H, H-2_B), 3.76-3.70 (m, 2H, H-5_B, H-$6a_A$), 3.64 (m, 1H, H-4_A), 3.54-3.48 (m_{po}, 1H, H-$6b_A$), 3.47 (pt, 1H, $J_{3,4}$ = 4.1 Hz, H-3_A), 1.79 (s, 3H, CH_{3Ac}), 1.27 (d, 3H, $J_{5,6}$ = 6.4 Hz, H-6_B). ^{13}CNMR (DMSO-d_6) δ 169.0 (CO_{NHTCA}), 162.2 (CO_{NHAc}), 139.4 ($C_{q,Ar}$), 134.8 (CH_{All}), 128.2, 127.9, 127.4 (C_{Ar}), 116.8 (CH_{2All}), 102.0 (C-1_A, $^1J_{C,H}$ = 170 Hz), 100.3 (C-1_B, $^1J_{C,H}$ = 163 Hz), 93.5 (CCl_3), 77.4 (C-3_B), 76.9 (C-3_A), 71.6 (C-5_A), 71.0 (CH_{2Bn}), 69.7 (C-5_B), 69.3 (CH_{2All}), 65.3 (C-4_B), 64.9 (C-4_A), 62.0 (C-6_A), 53.4 (C-2_B), 49.4 (C-2_A), 22.9 (CH_{3Ac}), 17.7 (C-6_B). HRMS (ESI$^+$): m/z $[M+H]^+$ calcd for $C_{26}H_{35}Cl_3N_5O_9$ 666.1486; found 666.1500.

Allyl (benzyl 2-acetamido-3-*O*-benzyl-2-deoxy-α-L-altropyranosyluronate)-(1→3)-4-azido-2-trichloroacetamido-2,4,6-trideoxy-β-D-galactopyranoside (29). *Route 1.* TEMPO (89 mg, 0.057 mmol, 0.2 equiv.) and BAIB (242 mg, 0.75 mmol, 2.5 equiv.) were added to diol **28** (200 mg, 301 μmol, 1.0 equiv.) in DCM/H_2O (2:1, 15 mL). The reaction was stirred at rt for 4 h. At completion as indicated by TLC (EtOAc/MeOH 9:1), 10% aq. Na_2SO_3 was added and the biphasic mixture was diluted with DCM (20 mL). The aq. phase was separated and extracted with DCM (10 mL) twice. The aq. phase was acidified with dilute aq. HCl to reach pH 1 and washed with DCM (10 mL) thrice. The combined organic phases were washed with brine, dried by passing through a phase separator filter and concentrated under reduced pressure. Benzyl bromide (142 μL, 1.2 mmol, 4.0 equiv.) and K_2CO_3 (83 mg, 0.60 mmol, 2.0 equiv.) were added to the residue in anhyd. DMF (2 mL). After stirring at rt for 1 h, the reaction mixture was diluted with H_2O (50 mL) and the aq. layer was extracted with DCM (20 mL) thrice. The organic phases were combined, washed with brine (20 mL), dried over anhyd. Na_2SO_4, filtered and concentrated *in vacuo*. Flash chromatography eluting with Tol/EtOAc (3:1→2:1) gave the benzyl ester **29** (85 mg, 110 μmol, 36%) as a white solid.

Route 2. The benzyl ester **37** (see below, 340 mg, 374 μmol, 1.0 equiv.) was dissolved in DCM (6.0 mL) and phosphate buffer pH 7 (1.0 mL) was added. The biphasic mixture was cooled to 0 °C and DDQ (144 mg, 636 μmol, 1.7 equiv.) was added. Stirring was pursued for 2 h while the bath was allowed to reach rt. The mixture was diluted with DCM (10 mL) and the DCM layer was washed with satd aq. $NaHCO_3$, water and brine, then dried over Na_2SO_4, and concentrated under reduced pressure. The residue was purified by flash chromatography (Tol/EtOAc 65:35→55:45) to give alcohol **29** (290 mg, 325 μmol, 87%) as a white solid. Disaccharide **29** had R_f 0.3 (Tol/EtOAc 1:1). ^{1}H NMR ($CDCl_3$) δ 7.43-7.31 (m, 10H, H_{Ar}), 6.81 (d, 1H, $J_{2,NH}$ = 7.2 Hz, NH_B), 5.89-5.79 (m, 1H, CH_{All}), 7.93 (d, 1H, $J_{2,NH}$ = 8.4 Hz, NH_A), 5.28 (d, 1H, $J_{2,NH}$ = 12.1 Hz, CH_{2Bn-6}, CH_{2All}), 5.29-5.23 (m_{po}, 1H, CH_{2All}), 5.22 (d_{po}, 1H, CH_{2Bn-6}), 5.20-5.16 (m, 1H, CH_{2All}), 4.93 (d, 1H, $J_{1,2}$ = 3.2 Hz, H-1_A), 4.78 (d_{po}, 1H, $J_{1,2}$ = 8.4 Hz, H-1_B), 4.73 (d, 1H, J = 11.8 Hz, CH_{2Bn}), 4.69

(d$_{po}$, 1H, $J_{4,5}$ = 7.8 Hz, H-5$_A$), 4.66 (d, 1H, CH_{2Bn}), 4.51 (dd, 1H, $J_{3,4}$ = 3.7 Hz, $J_{2,3}$ = 10.7 Hz, H-3$_B$), 4.34-4.39 (m, 2H, H-2$_A$, CH_{2All}), 4.12 (ddd, 1H, $J_{4,5}$ = 7.8 Hz, H-4$_A$), 4.07-4.01 (m, 1H, CH_{2All}), 3.85 (dd, 1H, $J_{2,3}$ = 5.0 Hz, $J_{3,4}$ = 3.5 Hz, H-3$_A$), 3.80 (d, 1H, $J_{3,4}$ = 3.2 Hz, H-4$_B$), 3.61 (dq, 1H, $J_{4,5}$ = 0.9 Hz, H-5$_B$), 3.55 (ddd, $J_{2,3}$ = 10.8 Hz, 1H, H-2$_B$), 2.76 (d, 1H, $J_{4,OH}$ = 8.2 Hz, OH), 1.95 (s, 3H, CH_{3Ac}), 1.27 (d, 3H, $J_{5,6}$ = 6.4 Hz, H-6$_B$). ^{13}C NMR ($CDCl_3$) δ 169.5 (CO_{NHTCA}), 169.4 (C-6), 162.1 (CO_{NHAc}), 137.6, 135.0 ($C_{q,Ar}$), 133.5 (CH_{All}), 128.7, 128.6 (2C), 128.4 (2C), 128.2, 128.0 (C_{Ar}), 117.9 (CH_{2All}), 100.6 (C-1$_A$, $^1J_{C,H}$ = 170 Hz), 97.6 (C-1$_B$, $^1J_{C,H}$ = 162 Hz), 92.2 (CCl_3), 76.7 (C-3$_B$), 75.2 (C-3$_A$), 72.0 (CH_{2Bn}), 71.0 (C-5$_A$), 70.0 (CH_{2All}), 69.6 (C-5$_B$), 67.4 (CH_{2Bn-6}), 65.6 (C-4$_A$), 65.4 (C-4$_B$), 55.3 (C-2$_B$), 49.5 (C-2$_A$), 23.2 (CH_{3Ac}), 17.2 (C-6$_B$). HRMS (ESI$^+$): *m/z* $[M+Na]^+$ calcd for $C_{33}H_{38}Cl_3N_5O_{10}Na$ 792.1582; found 792.1584.

(Benzyl 2-acetamido-3-*O*-benzyl-2-deoxy-4-*O*-(2-naphthylmethyl)-α-L-altropyranosyluronate)-(1→3)-4-azido-2-trichloroacetamido-2,4,6-trideoxy-α/β-D-galactopyranose (38). $[Ir(COD)(PMePh_2)_2]PF_6$ (13 mg, 15 µmol, 0.02 equiv.) in anhyd. THF (4.0 mL) was degassed repeatedly and stirred for 30 min under a hydrogen atmosphere. The resulting yellow solution was degassed several times with Ar and transferred by means of a cannula into a solution of allyl glycoside **37** (700 mg, 770 µmol, 1.0 equiv.) in anhyd. THF (10 mL). After stirring for 2 h at rt, NIS (191 mg, 847 µmol, 1.1 equiv.) and H_2O (12 mL) were added. After stirring for an additional hour, a TLC analysis (Tol/EtOAc 7:3) showed the complete consumption of disaccharide **37** (R_f 0.45) and the presence of a polar spot (R_f 0.2). 10% Aq. Na_2SO_3 was added. Votatiles were removed under reduced pressure and the aq. phase was extracted with DCM (20 mL) twice. The combined organic layers were washed with brine, dried over anhyd. Na_2SO_4, and concentrated. Purification of the residue by flash chromatography (cHex/EtOAc 5:1→4:1) gave the expected hemiacetal **38** (620 mg, 713 µmol, 92%) as a white solid. Hemiacetal **38**, isolated as a 10:7 α/β mixture had R_f 0.15 (Tol/EtOAc 1:1). The major anomer had ^{1}H NMR ($CDCl_3$) δ 7.84-7.26 (m, 17H, H_{Ar}), 6.94 (d, $J_{2,NH}$ = 7.2 Hz, NH$_B$), 5.90-5.80 (m, 1H, CH_{All}), 5.73 (d, $J_{2,NH}$ = 6.8 Hz, NH$_A$), 5.33 (d, 1H, $J_{1,2}$ = 5.6 Hz, H-1$_A$), 5.27-5.22 (m, 1H, CH_{2All}), 5.20-5.14 (m$_{po}$, 3H, CH_{2All}, CH_{2Bn-6}), 4.80 (d, 1H, $J_{4,5}$ = 4.4 Hz, H-5$_A$), 4.77 (d, 1H, $J_{1,2}$ = 8.4 Hz, H-1$_B$), 4.70 (brs, 2H, CH_{2Nap}), 4.53-4.46 (m, 3H, H-3$_B$, CH_{2Bn}), 4.33-4.28 (m, 1H, CH_{2All}), 4.10 (dd$_{po}$, $J_{3,4}$ = 2.8 Hz, H-4$_A$), 4.07-4.00 (m, 3H, H-3$_A$, H-2$_A$, CH_{2All}), 3.93 (brd, 1H, $J_{3,4}$ = 3.2 Hz, H-4$_B$), 3.57 (ddd$_{po}$, 1H, $J_{2,3}$ = 9.2 Hz, H-2$_B$), 3.47 (brq, 1H, H-5$_B$), 1.86 (s, 3H, CH_{3NAc}), 1.23 (d, 3H, $J_{5,6}$ = 6.4 Hz, H-6$_B$). ^{13}C NMR ($CDCl_3$) δ 170.4 (CO_{NTCA}), 169.3 (C-6$_A$), 161.9 (CO_{NAc}), 137.9, 134.9, 134.8, 133.1, 133.0 ($C_{q,Ar}$), 133.5 (CH_{All}), 128.7 (2C), 128.6, 128.3, 128.1, 127.9, 127.8, 127.6, 126.9, 126.1, 126.0, 125.9 (C_{Ar}), 117.9 (CH_{2All}), 99.5 (C-1$_A$, $^1J_{C,H}$ = 169 Hz), 97.6 (C-1$_B$, $^1J_{C,H}$ = 162 Hz), 92.4 (CCl_3), 76.6 (C-3$_B$), 73.4 (C-3$_A$), 72.9 (C-4$_A$), 71.9 (C-5$_A$), 71.8 (2C, CH_{2Bn}, CH_{2Nap}), 70.1 (CH_{2All}), 69.3 (C-5$_B$), 67.5 (CH_{2Bn-6}), 65.1 (C-4$_B$), 55.0 (C-2$_B$), 52.1 (C-2$_A$), 23.4 (CH_{3Ac}), 17.3 (C-6$_B$). HRMS (ESI$^+$): *m/z* $[M+H]^+$ calcd for $C_{41}H_{43}Cl_3N_5O_{10}$ 870.2076; found 870.2070.

(Benzyl 2-acetamido-3-*O*-benzyl-2-deoxy-4-*O*-(2-naphthylmethyl)-α-L-altropyranosyluronate)-(1→3)-4-azido-2-trichloroacetamido-2,4,6-trideoxy-α/β-D-galactopyranosyl (*N*-phenyl)trifluoroacetimidate (39) and 2-Trichloromethyl-[(benzyl 2-

acetamido-3-*O*-benzyl-2-deoxy-4-*O*-(2-naphthylmethyl)-α-L-altropyranosyluronate)-(1→3)-4-azido-1,2,4,6-tetradeoxy-α-D-galactopyrano]-[2,1,d]-oxazoline (40). Hemiacetal **38** (630 mg, 725 µmol, 1.0 equiv.) was dissolved in acetone (10 mL). PTFACl (149 µl, 942 µmol, 1.3 equiv.) was added followed by Cs_2CO_3 (260 mg, 797 µmol, 1.1 equiv.). The reaction mixture was stirred for 2 h at rt under an Ar atmosphere. A TLC follow up (Tol/EtOAc 5:1) showed that the starting **38** had evolved into two less polar spots (R_f 0.3 and 0.35). The reaction mixture was filtered over a pad of Celite, washed with acetone (10 mL) twice and the filtrate was concentrated under reduced pressure. The residue was purified by rapid flash chromatography (cHex/EtOAc 4:1→2:1, 1% Et_3N) to give the desired donor as a 3:2 mix of **39** and **40** (670 mg, 644 µmol, 89%) isolated as a floppy white solid. The isolated mix of donors **39** and **40** had ^{1}H NMR ($CDCl_3$) δ 7.87-6.80 (m, $22H_{Ar,PTFA}$, $NH_{B,PTFA}$, 17H-$_{Ar,oxa}$), 6.56 (bs, 0.6H, H-$1_{B,PTFA}$), 6.18 (d, 0.4H, $J_{1,2}$ = 7.0 Hz, H-$1_{B,oxa}$), 5.86 (d, 0.4H, $J_{2,NH}$ = 7.2 Hz, $NH_{A,oxa}$), 5.48 (d, 0.4H, $J_{1,2}$ = 5.6 Hz, H-$1_{A,oxa}$), 5.41 (d_{po}, 0.6H, $J_{1,2}$ = 7.6 Hz, H-$1_{A,PTFA}$), 5.39 (dpo, $J_{1,2}$ = 8.0 Hz, $NH_{A,PTFA}$), 5.23 (d, 0.4H, J = 12 Hz, CH_{2Bn-6}), 5.16 (s_{po}, 1.2H, CH_{2Bn-6}), 5.14 (d_{po}, 0.4H, CH_{2Bn-6}), 4.87 (d, 0.6H, J = 12.5 Hz, CH_{2Nap}), 4.81 (d, 0.6H, CH_{2Nap}), 4.78 (d_{po}, 0.6H, $J_{3,4}$ = 3.0 Hz, H-5_A), 4.76 (d_{po}, 0.6H, $J_{3,4}$ = 4.9 Hz, H-5_A), 4.69 (d, 0.4H, J = 12.4 Hz, CH_{2Nap}), 4.64 (d, 0.4H, CH_{2Nap}), 4.59 (d, 0.4H, J = 12.1 Hz, CH_{2Bn}), 4.54 (d, 0.4H, CH_{2Bn}), 4.48 (dd, 0.6H, $J_{3,4}$ = 3.1 Hz, $J_{3,4}$ = 11.0 Hz, H-3_B), 4.42 (d_o, 0.6H, J = 12.0 Hz, CH_{2Bn}), 4.42-4.37 (m_o, 0.6H, H-2_B), 4.30 (ddd, 0.6H, H-2_A), 4.26 (bs, 0.6H, H-4_B), 4.20-4.23 (m, 2.2H, CH_{2Bn}, H-3_A, H-4_A, H-2_B), 4.10 (dd_{po}, 0.6H, H-4_A), 4.08-4.03 (m_{po}, 0.4H, H-2_A), 4.05-4.99 (m, 0.6H, H-5_B), 3.86 (m, 0.4H, H-4_B), 3.75 (dd, 0.4H, H-5_B), 3.57 (dd, 0.4H, $J_{3,4}$ = 3.7 Hz, $J_{2,3}$ = 8.1 Hz, H-3_B), 3.54 (dd, 0.6H, $J_{2,3}$ = 10.1 Hz, $J_{3,4}$ = 2.8 Hz, H-3_A), 1.94 (s, 1.8H, CH_{3Ac}), 1.93 (s, 1.2H, CH_{3Ac}), 1.28 (d_{po}, 0.6H, $J_{5,6}$ = 6.3 Hz, H-6_B), 1.27 (d_{po}, 0.4H, $J_{5,6}$ = 6.3 Hz, H-6_B). ^{13}C NMR (Partial, $CDCl_3$) δ 173.2, 170.7, 170.5 (CO_{NTCA}), 169.3, 168.9 (C-6_A), 162.9, 162.1 (CO_{NAc}), 143.0, 137.9, 137.3, 135.0, 134.9, 134.7, 134.6, 133.1, 133.0 ($C_{q,Ar}$), 129.1, 128.8 (2C), 128.6, 128.5, 128.4, 128.3 (2C), 128.2, 128.0 (2C), 127.9 (2C), 127.8, 127.6, 127.2, 126.9, 126.8, 126.2, 126.1 (2C), 126.0 (2C), 125.9, 124.4, 119.4, 118.3 (C_{Ar}), 107.6 (C-$1_{B,oxa}$, $^1J_{C,H}$ = 183 Hz), 99.0 (C-1_A, $^1J_{C,H}$ = 169 Hz), 98.8 (C-1_A, $^1J_{C,H}$ = 171 Hz), 93.7 (bs, C-1_{B-PTFA}), 92.4 (CCl_3), 86.9 (CCl_3), 81.1 (C-3_A), 74.5 (C-3_B), 73.6 (C-5_A), 73.1 (C-4_A), 73.0 (C-4_A), 72.5 (C-3_B), 72.1 (CH_{2Nap}), 71.8 (2C, CH_{2Nap}, CH_{2Bn}), 71.5 (C-5_A), 71.4 (2C, C-3_A, CH_{2Bn}), 69.0 (C-5_B), 68.0 (C-5_B), 67.4 (2C, CH_{2Bn-6}), 64.2 (C-4_B), 63.5 (C-2_B), 61.3 (C-4_B), 52.3 (C-2_A), 51.0 (C-2_A), 50.3 (C-2_B), 23.7, 23.4 (CH_{3Ac}), 17.5, 17.3 (C-6_B). HRMS (ESI$^+$): *m/z* $[M+H]^+$ calcd for $C_{49}H_{47}Cl_3F_3N_6O_{10}$ 1041.2372; found 1041.2378.

3-Azidopropyl (benzyl 2-acetamido-3-*O*-benzyl-2-deoxy-4-*O*-(2-naphthylmethyl)-α-L-altropyranosyluronate)-(1→3)-4-azido-2-trichloroacetamido-2,4,6-trideoxy-β-D-galactopyranoside (41). Freshly activated MS 4Å (50 mg) was added to donors **39**/**40** (2:1, 50 mg, 48 µmol, 1.0 equiv.) in anhyd. DCM (3.0 mL) containing 3-azidopropanol (22 µL, 240 µmol, 5.0 equiv.). The reaction mixture was stirred for 45 min at rt in an Ar atmosphere and cooled to 0 °C. $Yb(OTf)_3$ (3.0 mg, 5.0 µmol, 0.1 equiv.) was added and after stirring for 30 min at 0 °C, a TLC analysis (Tol/EtOAc 7:3) confirmed the absence of donors **39**/**40** (R_f 0.45, 0.55) and the presence of a major compound (R_f 0.2). Et_3N (2.0 µL) was added and the suspension was filtered over a

fitted funnel, and washed thoroughly with DCM. The filtrate was concentrated under reduced pressure and the residue was purified by flash chromatography (Tol/EtOAc 60:40→50:50) to give the condensation product **41** (36 mg, 37 μmol, 78%) as a white solid. The azidopropyl glycoside **41** had R_f 0.2 (Tol/EtOAc 4:1). ^{1}H NMR ($CDCl_3$) δ 7.85-7.71 (m, 4H, H_{Ar}), 7.51-7.47 (m, 2H, H_{Ar}), 7.42-7.25 (m, 11H, H_{Ar}), 6.83 (d, $J_{2,NH}$ = 7.2 Hz, NH_B), 5.55 (d, $J_{2,NH}$ = 7.2 Hz, NH_A), 5.28 (d, 1H, $J_{1,2}$ = 5.9 Hz, H-1_A), 5.22 (d, H, J = 12.0 Hz, CH_{2Bn-6}), 5.19 (d, H, CH_{2Bn-6}), 4.82 (d, 1H, $J_{4,5}$ = 4.8 Hz, H-5_A), 4.75 (d, H, J = 12.5 Hz, CH_{2Nap}), 5.22 (d, H, CH_{2Nap}), 4.65 (d, 1H, $J_{1,2}$ = 8.4 Hz, H-1_B), 4.53 (d, 1H, J = 11.9 Hz, CH_{2Bn}), 4.45 (d, 1H, CH_{2Bn}), 4.39 (dd, 1H, $J_{3,4}$ = 3.5 Hz, $J_{2,3}$ = 10.8 Hz, H-3_B), 4.11 (dd, $J_{3,4}$ = 2.8 Hz, H-4_A), 4.07 (pdt, 1H, H-2_A), 3.97 (dd_{po}, 1H, $J_{2,3}$ = 8.2 Hz, H-3_A), 3.93-3.88 (m, 2H, H-4_B, OCH_2), 3.62 (ddd, 1H, H-2_B), 3.56-3.51 (m, 1H, OCH_2), 3.49 (brq, 1H, H-5_B), 3.38 (t, 2H, J = 6.8 Hz, NCH_2), 1.89-1.78 (m, 2H, CH_2), 1.87 (s, 3H, CH_{3NHAc}), 1.23 (d, 3H, $J_{5,6}$ = 6.2 Hz, H-6_B). ^{13}C NMR ($CDCl_3$) δ 170.3 (CO_{NHTCA}), 169.3 (C-6_A), 161.8 (CO_{NHAc}), 137.8, 134.9, 134.8, 133.1, 133.0 ($C_{q,Ar}$), 128.7, 128.6, 128.3, 128.2, 128.1, 127.8 (2C), 127.7, 126.8, 126.1, 126.0, 125.9 (C_{Ar}), 99.5 (C-1_A, $^1J_{C,H}$ = 171 Hz), 97.2 (C-1_B, $^1J_{C,H}$ = 162 Hz), 92.5 (CCl_3), 76.5 (C-3_B), 73.6 (C-3_A), 72.7 (C-4_A), 71.9 (C-5_A), 71.8 (2C, $CH_{2Bn,}$ CH_{2Nap}), 69.4 (C-5_B), 67.4 (CH_{2Bn-6}), 66.3 (OCH_2), 64.9 (C-4_B), 54.7 (C-2_B), 52.0 (C-2_A), 48.1 (CH_2N_3), 29.0 (CH_2), 23.5 (CH_{3Ac}), 17.3 (C-6_B). HRMS (ESI$^+$): *m/z* $[M+H]^+$ calcd for $C_{44}H_{48}Cl_3N_8O_{10}$ 953.2559; found 953.2542.

Allyl (benzyl 2-acetamido-3,4-di-*O*-benzyl-2-deoxy-α-L-altropyranosyluronate)-(1→3)-(4-azido-2-trichloroacetamido-2,4,6-trideoxy-β-D-galactopyranosyl)-(1→3)-(benzyl 3-*O*-benzyl-2-(*N*,*N*-diacetyl)amino-2-deoxy-α-L-altropyranosyluronate)-(1→3)-4-azido-2-trichloroacetamido-2,4,6-trideoxy-β-D-galactopyranoside (S7). Donors **S5**/**S6** (not described, 140 mg, 141 μmol, 1.25 equiv.) and acceptor **48** (92 mg, 113 μmol, 1.0 equiv.) were coevaporated with anhyd. toluene (2 mL) twice and subjected to extensive drying under high vacuum. The mixture was dissolved in anhyd. DCE (5.0 mL) and freshly activated MS 4Å (100 mg) was added. The mixture stirred under an Ar atmosphere for 1 h and cooled to 0 °C. TfOH (1.2 μL in 50 μL anhyd. ACN) was added slowly. The mixture was allowed to reach rt slowly and was stirred for 1 h. A TLC analysis (Tol/EtOAc 6:1) showed donor consumption and the presence of two more polar spots (R_f 0.25 and R_f 0.0). Et_3N (2.0 μL) was added and the suspension was filtered over a fitted funnel. The filtrate was concentrated and the residue was purified by flash chromatography (Tol/EtOAc 10:1→3:1) to give tetrasaccharide **S7** (60 mg, 38 μmol, 33%, corr. yield 91%) as a white solid, along with the recovered acceptor **48** (58 mg, 72 μmol, 63%) and the hemiacetal issued from **S5**/**S6** hydrolysis (80 mg, 98 μmol, 69% wrt **S5**/**S6**). ^{1}H NMR ($CDCl_3$) δ 7.46-7.12 (m, 25H, H_{Ar}), 6.90 (d, 1H, $J_{2,NH}$ = 6.8 Hz, NH_{B1}), 6.72 (d, 1H, $J_{2,NH}$ = 7.6 Hz, NH_B), 5.90-5.80 (m, 1H, CH_{All}), 5.64 (d, 1H, $J_{1,2}$ = 8.0 Hz, H-1_A), 5.54 (d, $J_{2,NH}$ = 8.0 Hz, NH_{A1}), 5.27-5.21 (m, 5H, CH_{2All}, CH_{2Bn-6}), 5.19-5.15 (m, 1H, CH_{2All}), 5.10 (d, 1H, $J_{1,2}$ = 4.8 Hz, H-1_{A1}), 4.88 (d, 1H, $J_{1,2}$ = 8.4 Hz, H-1_{B1}), 4.82-4.81 (m, 2H, H-5_A, H-5_{A1}), 4.76 (d, 1H, $J_{1,2}$ = 8.4 Hz, H-1_B), 4.58-4.48 (m, 5H, H-3_{B1}, CH_{2Bn}), 4.44 (dd, 1H, $J_{2,3}$ = 10.7 Hz, $J_{3,4}$ = 3.8 Hz, H-3_B), 4.44-4.37 (d_{po}, 1H, J = 11.6 Hz, CH_{2Bn}), 4.38-4.37 (m_{po}, 1H, H-4_A), 4.33-4.26 (m_{po}, 2H, CH_{2All}, CH_{2Bn}), 4.26 (dd, 1H, $J_{2,3}$ = 10.6 Hz, $J_{3,4}$ = 3.4 Hz, H-3_A), 4.17-4.12 (m, 1H, H-2_{A1}), 4.08 (d, 1H, $J_{3,4}$ = 3.7 Hz, H-4_{B1}), 4.05-3.98

(m, 2H, CH_{2All}, H-2_A), 3.95 (dd_{po}, 1H, H-4_{A1}), 3.90 (dd, 1H, $J_{3,4}$ = 2.8 Hz, $J_{3,4}$ = 6.8 Hz, H-3_{A1}), 3.85 (d, 1H, $J_{3,4}$ = 3.2 Hz, H-4_B), 3.55-3.44 (m, 3H, H-2_B, H-2_{B1}, H-5_B), 3.46 (dq_{po}, 1H, H-5_{B1}), 2.35 (brs, 3H, CH_{3Ac}), 2.31 (brs, 3H, CH_{3Ac}), 1.93 (s, 3H, CH_{3Ac}), 1.29 (d, 3H, $J_{5,6}$ = 6.4 Hz, H-6_{B1}), 1.19 (d, 3H, $J_{5,6}$ = 6.4 Hz, H-6_{B1}). ^{13}C NMR ($CDCl_3$) δ 176.9 (CO_{NAc})169.9, 169.6 (C-6_A), 168.3 161.9, 161.6 (CO_{NTCA}), 138.0, 137.4 (2C), 135.0 (2C) ($C_{q,Ar}$), 133.5 (CH_{All}), 129.0, 128.9, 128.8, 128.7, 128.6, 128.5, 128.4 (2C), 128.3, 128.2, 128.0 (2C), 127.9, 127.8, 127.7, 125.2 (C_{Ar}), 117.9 (CH_{2All}), 100.0 (C-1_{A1}, $^1J_{C,H}$ = 169 Hz), 99.7 (C-1_{B1}, $^1J_{C,H}$ = 165 Hz), 98.4 (C-1_A, $^1J_{C,H}$ = 175 Hz), 97.6 (C-1_B, $^1J_{C,H}$ = 162 Hz), 92.4 (CCl_3), 92.1 (CCl_3), 76.7 (C-3_{B1}), 76.1 (C-5_A), 75.8 (C-3_B), 73.4 (C-3_A), 73.2 (C-4_A), 72.6 (C-3_{A1}), 72.0, 71.6, 71.5 (CH_{2Bn}), 71.3 (C-4_{A1}), 71.0 (C-5_{A1}), 70.1 (CH_{2All}), 69.7, 68.5 (2C, C-5_B, C-5_{B1}), 67.5, 67.4 (CH_{2Bn-6}), 65.2 (C-4_{B1}), 64.8 (C-4_{B1}), 59.4 (C-2_A), 55.3, 55.2 (2C, C-2_B, C-2_{B1}), 51.9 (C-2_{A1}), 27.7, 25.3, 23.4 (3C, CH_{3Ac}), 17.4, 17.2 (2C, C-6_B, C-6_{B1}). HRMS (ESI$^+$): *m/z* $[M+NH_4]^+$ calcd for $C_{72}H_{79}Cl_6N_{10}O_{20}$ 1615.3589; found 1615.3596.

Allyl (benzyl 2-(*N-tert*-butyloxycarbonyl)acetamido-3-*O*-benzyl-2-deoxy-4-*O*-(2-naphthylmethyl)-α-L-altropyranosyluronate)-(1→3)-4-azido-2-trichloroacetamido-2,4,6-trideoxy-β-D-galactopyranoside (57). Di-*tert*-butylcarbonate (1.22 g, 5.6 mmol, 8.0 equiv.) followed by DMAP (34 mg, 282 μmol, 0.4 equiv.) were added to disaccharide **37** (640 mg, 704 μmol, 1.0 equiv.) in anhyd. THF (20 mL). After heating at 50 °C for 2 h, a TLC follow up (Tol/EtOAc 4:1) showed the presence of a less polar spot (R_f 0.75) and the absence of the starting **37** (R_f 0.1). The reaction mixture was allowed to reach rt and concentrated under reduced pressure. The residue was purified by flash chromatography (Tol/EtOAc 10:1→7:1) to give the desired **57** as a white solid (520 mg, 515 μmol, 73%). Disaccharide **57** had ^{1}H NMR ($CDCl_3$) δ 7.84-7.74 (m, 4H, H_{Ar}), 7.49-7.46 (m, 3H, H_{Ar}), 7.39-7.36 (m, 5H, H_{Ar}), 7.29-7.18 (m, 5H, H_{Ar}), 6.75 (d, $J_{2,NH}$ = 7.2 Hz, NH_B), 5.92-5.82 (m, 1H, CH_{All}), 5.72 (d, 1H, $J_{1,2}$ = 8.2 Hz, H-1_A), 5.29-5.23 (m, 1H, CH_{2All}), 5.22 (d_{po}, 1H, CH_{2Bn-6}), 5.19 (d_{po}, 1H, J = 12.1 Hz, CH_{2Bn-6}), 5.20-5.16 (m_{po}, 1H, CH_{2All}), 5.00-4.89 (br, 1H, H-2_A), 4.85 (d_{po}, 1H, J = 12.3 Hz, CH_{2Bn}), 4.81 (d_{po}, 1H, CH_{2Bn}), 4.75 (d_{po}, 1H, $J_{1,2}$ = 8.3 Hz, H-1_B), 4.72 (d, $J_{4,5}$ = 2.6 Hz, H-5_A), 4.42 (d_{po}, 1H, J = 11.8 Hz, CH_{2Nap}), 4.38 (dd_{po}, 2H, $J_{2,3}$ = 10.5 Hz, $J_{3,4}$ = 2.3 Hz, H-3_B), 4.37 (pt_o, 1H, $J_{3,4}$ = 2.8 Hz, H-4_A), 4.34-4.29 (m, 2H, CH_{2Nap}, CH_{2All}), 4.24 (brd, 1H, $J_{2,3}$ = 10.4 Hz, H-3_A), 4.05 (bd, 1H, H-4_B), 4.05-4.00 (m, 1H, CH_{2All}), 3.61-3.54 (pdt, 1H, H-2_B), 3.44 (dq, 1H, $J_{4,5}$ = 1.0 Hz, H-5_B), 2.34 (s, 3H, CH_{3NAc}), 1.48 (s, 9H, CH_{3NBoc}), 1.26 (d, 3H, $J_{5,6}$ = 6.2 Hz, H-6_B). ^{13}C NMR ($CDCl_3$) δ 174.4 (br, CO_{NAc}), 169.1 (C-6_A), 161.7 (CO_{NTCA}), 153.9 (br, CO_{NBoc}), 137.8, 135.5, 135.0, 133.2, 132.9 ($C_{q,Ar}$), 133.6 (CH_{All}), 129.0, 128.8, 128.7 (2C), 128.2 (2C), 127.8, 127.6, 126.2, 126.0, 125.8, 125.7, 125.2 (10C, C_{Ar}), 117.9 (CH_{2All}), 98.9 (C-1_A, $^1J_{C,H}$ = 174 Hz), 97.9 (C-1_B, $^1J_{C,H}$ = 163 Hz), 92.4 (CCl_3), 83.7 (C_{Boc}), 76.9 (br, C-3_B), 74.1 (2C, C-4_A, C-5_A), 73.7 (br, C-3_A), 72.5 (CH_{2Nap}), 71.7 (CH_{2Bn}), 70.1 (CH_{2All}), 68.8 (C-5_B), 67.4 (CH_{2Bn-6}), 65.3 (C-4_B), 55.0 (C-2_B), 27.8 (CH_{3Boc}), 27.1 (CH_{3Ac}), 17.4 (C-6_B). HRMS (ESI$^+$): *m/z* $[M+NH_4]^+$ calcd for $C_{49}H_{58}Cl_3N_6O_{12}$ 1027.3179; found 1027.3176.

Allyl (benzyl 2-(*N-tert*-butyloxycarbonyl)acetamido-3-*O*-benzyl-2-deoxy-α-L-altropyranosyluronate)-(1→3)-4-azido-2-trichloroacetamido-2,4,6-trideoxy-β-D-

galactopyranoside (58). DDQ (256 mg, 1.1 mmol, 3.0 equiv.) was added to the fully protected disaccharide **57** (380 mg, 377 µmol, 1.0 equiv.) in DCM/phosphate buffer pH 7 (10:1, 10 mL). After stirring vigorously at rt for 2 h, TLC analysis (Tol/EtOAc 4:1) revealed that the starting **57** (R_f 0.75) had evolved into a more polar product (R_f 0.35). 5% Aq. $NaHCO_3$ (10 mL) and DCM (10 mL) were added. The DCM layer was separated, washed with brine (25 mL), dried over Na_2SO_4, and concentrated. The residue was purified by flash chromatography (Tol/EtOAc 5:1→4:1) to give alcohol **58** as a white solid (260 mg, 257 µmol, 79%). Disaccharide **58** had ^{1}H NMR ($CDCl_3$) δ 7.44-7.40 (m, 5H, H_{Ar}), 7.32-7.22 (m, 5H, H_{Ar}), 6.65 (d, $J_{2,NH}$ = 7.6 Hz, NH_B), 5.91-5.81 (m, 1H, CH_{All}), 5.61 (d, 1H, $J_{1,2}$ = 8.0 Hz, H-1_A), 5.28-5.22 (m_{po}, 1H, CH_{2All}), 5.25 (s, 2H, $CH_{2Bn\text{-}6}$), 5.19-5.15 (m, 1H, CH_{2All}), 4.71 (d_{po}, 1H, $J_{4,5}$ = 2.6 Hz, H-5_A), 4.70-4.62 (br, 1H, H-2_A), 4.65 (d, 1H, $J_{1,2}$ = 8.4 Hz, H-1_B), 4.55-4.53 (m_{po}, 1H, H-4_A), 4.54 (d_{po}, 1H, CH_{2Bn}), 4.42 (d, 1H, J = 11.6 Hz, CH_{2Bn}), 4.35 (dd, 1H, $J_{2,3}$ = 10.8 Hz, $J_{3,4}$ = 3.7 Hz, H-3_B), 4.33-4.27 (m, 2H, H-3_A, CH_{2All}), 4.04-3.99 (m, 2H, H-4_B, CH_{2All}), 3.61 (pt, 1H, H-2_B), 3.39 (dq, 1H, $J_{4,5}$ = 1.0 Hz, H-5_B), 2.59 (d, 1H, $J_{4,OH}$ = 2.2 Hz, OH), 2.31 (s, 3H, CH_{3Ac}), 1.50 (s, 9H, CH_{3Boc}), 1.25 (d, 3H, $J_{5,6}$ = 6.3 Hz, H-6_B). ^{13}C NMR ($CDCl_3$) δ 174.1 (br, CO_{NAc}), 168.8 (C-6_A), 161.7 (CO_{NTCA}), 153.6 (br, CO_{NBoc}), 137.2, 135.0 ($C_{q,Ar}$), 133.6 (CH_{All}), 128.8 (2C), 128.5, 128.1, 127.8, (10C, C_{Ar}), 117.9 (CH_{2All}), 98.5 (C-1_A, $^1J_{C,H}$ = 174 Hz), 98.1 (C-1_B, $^1J_{C,H}$ = 161 Hz), 92.4 (CCl_3), 83.9 (OC_{Boc}), 76.8 (C-3_B), 75.2 (C-5_A), 73.3 (br, C-3_A), 72.0 (CH_{2Bn}), 70.1 (CH_{2All}), 68.7 (C-5_B), 67.6 ($CH_{2Bn\text{-}6}$), 66.8 (C-4_A), 65.3 (C-4_B), 54.9 (C-2_B), 27.8 (CH_{3Boc}), 27.1 (CH_{3Ac}), 17.3 (C-6_B). HRMS (ESI$^+$): *m/z* $[M+NH_4]^+$ calcd for $C_{38}H_{50}Cl_3N_6O_{12}$ 887.2552; found 887.2558.

(Benzyl 2-(*N*-*tert*-butyloxycarbonyl)acetamido-3-*O*-benzyl-2-deoxy-4-*O*-(2-naphthylmethyl)-α-L-altropyranosyluronate)-(1→3)-4-azido-2-trichloroacetamido-2,4,6-trideoxy-α/β-D-galactopyranose (59). A solution of $[Ir(COD)(PMePh_2)_2]PF_6$ (12 mg, 13 µmol, 0.03 equiv.) in anhyd. THF (3.0 mL), was degassed and stirred for 20 min under an H_2 atmosphere. The resulting yellow solution was degassed repeatedly with Ar and poured into a solution of allyl glycoside **57** (430 mg, 473 µmol, 1.0 equiv.) in anhyd. THF (16 mL). After stirring for 1 h at rt, NIS (117 mg, 520 µmol, 1.1 equiv.) and H_2O (4.0 mL) were added. After stiring for another hour at rt, a TLC follow up (CHex/EtOAc 6:1) showed the absence of allyl glycoside **57** (R_f 0.6) and products in close vicinity. 10% Aq. Na_2SO_3 was added, and volatiles were evaporated. The aq. phase was extracted with DCM (10 mL) twice. The combined organic layers were washed with brine (30 mL), dried over anhyd. Na_2SO_4, filtered, and concentrated under reduced pressure. Purification of the residue by flash chromatography (Chex/EtOAc 70:30→65:35) gave the expected hemiacetal **59** (360 mg, 356 µmol, 87%) as a white solid. Hemiacetal **59** had R_f 0.15, 0.25 (Tol/EtOAc, 3:1). ^{1}H NMR (major isomer, $CDCl_3$) δ 7.84-7.17 (m, 17H, H_{Ar}), 6.65 (d, 1H, $J_{2,NH}$ = 7.6 Hz, NH_B), 5.85 (d, 1H, $J_{1,2}$ = 8.4 Hz, H-1_A), 5.40 (brs, 1H, H-1_B), 5.24 (d, 1H, $CH_{2Bn\text{-}6}$), 5.16 (d, 1H, J = 12.0 Hz, $CH_{2Bn\text{-}6}$), 5.05 (brs, 1H, H-2_A), 4.90 (d, 1H, CH_{2Nap}), 4.82 (d, 1H, J = 12.3 Hz, CH_{2Nap}), 4.72 (d, 1H, $J_{4,5}$ = 2.4 Hz, H-5_A), 4.41 (d, 1H, J = 11.6 Hz, CH_{2Bn}), 4.36 (d_{po}, 1H, H-4_A), 4.35-4.26 (m, 2H, H-3_B, H-2_B), 4.20 (dd, 1H, $J_{2,3}$ = 10.5 Hz, $J_{3,4}$ = 2.1 Hz, H-3_A), 4.13 (brs_{po}, 1H, H-4_B), 4.11 (bq_{po}, 1H, H-5_B), 3.15 (brs, 1H, OH), 2.36 (s, 3H, CH_{3NAc}), 1.47 (s_{po}, 9H, CH_{3Boc}), 1.22 (d, 3H, $J_{5,6}$ = 6.2 Hz, H-6_B). ^{13}C NMR (major isomer, $CDCl_3$) δ 174.7 (br, CO_{NAcBoc}),

168.9 (C-6$_A$), 162.0 (CO$_{NTCA}$), 153.4 (br, CO$_{Boc}$), 137.7, 137.5, 135.4, 135.1, 134.8, 133.2, 133.0 (C$_{q,Ar}$), 128.9, 128.8, 128.7 (2C), 128.5, 128.3 (2C), 128.1, 127.8, 127.7, 126.3, 126.2, 126.0, 125.9, 125.7 (C$_{Ar}$), 98.0 (br, C-1$_A$, $^1J_{C,H}$ = 174 Hz), 92.4 (CCl$_3$), 90.8 (C-1$_B$, $^1J_{C,H}$ = 174 Hz), 84.1 (C$_{Boc}$), 74.2 (C-5$_A$), 74.0, 73.9 (2br, 3C, C-3$_B$, C-4$_A$, C-3$_A$), 72.7 (CH$_{2Nap}$), 71.6 (CH$_{2Bn}$), 67.4 (CH$_{2Bn-6}$), 65.6 (C-4$_B$), 64.6 (C-5$_B$), 55.4 (br, C-2$_A$), 51.1 (C-2$_B$), 27.8 (CH$_{3NBoc}$), 27.4 (CH$_{3Ac}$), 17.3 (C-6$_B$). HRMS (ESI$^+$): *m/z* [M+NH$_4$]$^+$ calcd for $C_{46}H_{58}Cl_3N_7O_{12}$ 987.2860; found 987.2862.

(Benzyl 2-(*N-tert*-butyloxycarbonyl)acetamido-3-*O*-benzyl-2-deoxy-4-*O*-(2-naphthylmethyl)-α-L-altropyranosyluronate)-(1→3)-4-azido-2-trichloroacetamido-2,4,6-trideoxy-α/β-D-galactopyranosyl) *N*-(phenyl)trifluoroacetamidate (60). PTFACl (23 μL, 148 μmol, 1.3 equiv.) and Cs_2CO_3 (197 mg, 604 μmol, 1.1 equiv.) were added to hemiacetal **59** (110 mg, 113 μmol, 1.0 equiv.) in acetone (4.0 mL). After stirring at rt for 2 h under an Ar atmosphere, a TLC follow up (Tol/EtOAc 6:1) indicated that hemiacetal **59** (R_f 0.1) had been converted to a less polar compound (R_f 0.85). The suspension was filtered over a pad of Celite, solids were washed with DCM (4 mL) twice, and volatiles were evaporated. The crude residue was purified by flash chromatography (Chex/EtOAc 90:10→98:12, 1% Et_3N) to give donor **60** (110 mg, 96 μmol, 86%) as a white solid. The donor had ^{1}H NMR (main isomer, CDCl$_3$) δ 7.85-7.10 (m, 21H, H$_{Ar}$), 6.79 (d, 2.2H, J = 7.6 Hz), 6.53 (brs, 0.9H), 5.94 (d, 1H, J = 8.4 Hz), 5.24 (d, 1H, CH$_{2Bn-6}$), 5.16 (d, 1H, J = 12.0 Hz, CH$_{2Bn-6}$), 4.90 (brs, 2H, CH$_{2Nap}$), 4.74 (d, 1H, J = 2.0 Hz, H-5$_A$), 4.37 (d$_{po}$, 2H, H-2$_A$, CH$_{2Bn}$), 4.36-4.33 (m, 3H, H-3$_A$, H-4$_A$, H-4$_B$), 4.23 (d$_{po}$, 1H, J = 11.9 Hz, CH$_{2Bn}$), 4.22-4.17 (m, 2H, H-2$_B$, H-3$_B$), 3.97-3.91 (dq, 1H, H-5$_B$), 2.37 (s, 3H, CH$_{3Ac}$), 1.47 (s, 9H, CH$_{3Boc}$), 1.25 (d, 3H, H-6$_B$). ^{13}C NMR (CDCl$_3$) δ 168.6, 162.1, 143.0, 138.0, 137.5, 135.0, 134.7, 133.2, 133.0 (C$_{q,Ar}$), 129.4, 129.0, 128.8, 128.7, 128.6 (2C), 128.5, 128.3, 128.2 (2C), 128.0, 127.9, 127.8 (2C), 127.6, 126.8, 126.3, 126.1, 126.0, 125.9, 125.7, 125.2, 124.4, 120.4, 119.3 (C$_{Ar}$), 107.3, 97.0, 93.4, 92.1, 84.4 (C$_{Boc}$), 77.2, 74.5 (C-5$_A$), 74.2 (2C), 72.5 (CH$_{2Nap}$), 71.6 (CH$_{2Bn}$), 67.7 (C-5$_B$), 67.5 (CH$_{2Bn-6}$), 64.5 (C-2$_A$), 50.3 (C-2$_B$), 27.9, 27.8, 27.3, 26.9, 17.6, 17.3. HRMS (ESI$^+$): *m/z* [M+NH$_4$]$^+$ calcd for $C_{54}H_{58}Cl_3F_3N_7O_{12}$ 1158.3156; found 1158.3137.

Allyl (benzyl 2-(*N-tert*-butyloxycarbonyl)acetamido-3-*O*-benzyl-2-deoxy-4-*O*-(2-naphthylmethyl)-α-L-altropyranosyluronate)-(1→3)-4-azido-2-trichloroacetamido-2,4,6-trideoxy-β-D-galactopyranyl)-(1→4)-(benzyl 2-(*N-tert*-butyloxycarbonyl)acetamido-3-*O*-benzyl-2-deoxy-α-L-altropyranosyluronate)-(1→3)-4-azido-2-trichloroacetamido-2,4,6-trideoxy-β-D-galactopyranoside (61). A mix of PTFA donor **60** (58 mg, 51 μmol, 1.1 equiv.) and acceptor **58** (40 mg, 46 μmol, 1.0 equiv.) was coevaporated with anhyd. toluene, dried under vacuum thoroughly, and taken into anhyd. DCE (2.0 mL) containing activated MS 4Å (100 mg). The reaction mixture was stirred at rt for 30 min under an Ar atmosphere and cooled to 0 °C. TfOH (0.2 μL, 0.05 equiv.) in 10 μL ACN was added. After stirring at this temperature for another 30 min, a TLC analysis (Tol/EtOAc 4:1) showed the absence of donor **60** and the presence of a new spot. Et_3N was added and solids were filtered off. Volatiles were evaporated and the crude was purified by flash chromatography (Tol/EtOAc 85:15→80:20) to give the desired **61** (35 mg, 19 μmol, 41%) as a white solid. Tetrasaccharide **61** had R_f 0.35 (Tol/EtOAc 4:1). ^{1}H NMR (CDCl$_3$)

δ 7.83-7.75 (m, 5H, H_{Ar}), 7.48-7.17 (m, 24H, H_{Ar}), 6.85 (brs, 1H, NH_{B*}), 6.85 (brs, 1H, NH_{B1*}), 5.89-5.83 (m, 1H, CH_{All}), 5.73 (d, 1H, $J_{1,2}$ = 8.0 Hz, H-1_{A*}), 5.53 (brd, 1H, $J_{1,2}$ = 7.6 Hz, H-1_{A1*}), 5.28-5.15 (m, 6H, $2CH_{2Bn-6}$, CH_{2All}), 5.03-4.88 (brs, 2H, H-2_{A*}, H-1_{B1}), 4.82 (brs, 3H, H-5_{A*}, CH_{2Nap}), 4.71-4.68 (m, 2H, H-5_{A1*}, H-1_{B}), 4.56-4.53 (m, 2H, H-2_{A1*}, H-3_{B*}), 4.44-4.21 (m, 9H, H-3_{B1*}, H-3_{A*}, H-3_{A1*}, H-4_{A*}, H-4_{A1*}, $2CH_{2Bn}$), 4.07-4.00 (m, 3H, H-4_{B}, CH_{2All}), 3.64-3.38 (m, 1H, H-2_{B}, H-5_{B}), 2.38-2.22 (s, 6H, CH_{3Ac}), 1.52-1.43 (m, 18H, CH_{3Boc}), 1.29 (brd, 3H, $J_{5,6}$ = 6.4 Hz, H-6_{B*}), 1.19 (brd, 3H, $J_{5,6}$ = 6.0 Hz, H-6_{B1*}). ^{13}C NMR ($CDCl_3$) δ 174.4 (CO_{NAcBoc}), 169.2, 168.7 (2C, C-6_{A} , C-6_{A1}), 161.9, 161.6 (2C, CO_{NTCA}), 153.8 (2C, CO_{NAcBoc}), 137.9, 137.8, 135.5, 135.1, 15.0, 133.2, 132.9 ($C_{q, Ar}$), 133.7 (CH_{All}), 129.0, 128.9, 128.8, 128.7, 128.6 (2C), 128.3, 128.2 (2C), 127.9, 127.8, 127.7, 127.6 (2C), 126.2, 126.0, 125.8, 125.7, 125.2 (10C, C_{Ar}), 117.6 (CH_{2All}), 99.4 (C-1_{B*}, $^{1}J_{C,H}$ = 169 Hz), 98.9 (C-1_{A*}, $^{1}J_{C,H}$ = 175 Hz), 98.5 (2C, C-1_{B1*}, $^{1}J_{C,H}$ = 164 Hz, C-1_{A1*}, $^{1}J_{C,H}$ = 177 Hz), 92.4 (CCl_3), 92.1 (CCl_3), 83.8 (C_{Boc}), 83.6 (C_{Boc}), 77.6, 77.2 (2C, C-3_{B}, C-3_{B1}), 76.0, 74.1 (2C, C-5_{A}, C-5_{A1}), 73.6 (2C, C-3_{A}, C-3_{A1}), 72.5 (CH_{2Nap}), 71.7, 71.6 (2C, CH_{2Bn}), 70.0 (CH_{2All}), 68.8 (2C, C-5_{B}, C-5_{B1}), 67.4, 67.3 (2C, CH_{2Bn-6}), 66.8 (2C, C-4_{A}, C-4_{A1}), 65.2 (2C, C-4_{B}, C-4_{B1}), 55.4 (2C, C-2_{A}, C-2_{A1}), 54.7 (2C, C-2_{B}, C-2_{B1}), 27.9, 27.8 (2C, CH_{3NBoc}), 27.4, 27.2 (2C, CH_{3Ac}), 17.4, 17.2 (2C, C-6_{B}, C-6_{B1}). HRMS (ESI$^+$): *m/z* $[M+NH_4]^+$ calcd for $C_{84}H_{98}Cl_6N_{11}O_{23}$ 1838.4963; found 1838.4982.

Allyl 3-*O*-benzyl-2-deoxy-4-*O*-(2-napthylmethyl)-6-*O*-*tert*-butyldiphenylsilyl-2-tetrachlorophthalimido-α-L-altropyranosyl-(1→3)-4-azido-2-dichloroacetamido-2,4,6-trideoxy-β-D-galactopyranoside (62). The crude PTFA donor **33** (737 mg, 680 µmol, 1.15 equiv.) and acceptor **5** (200 mg, 592 µmol, 1.0 equiv.) were mixed and co-evaporated with toluene (5 mL) twice. The mixture was dried thoroughly under high vacuum for 1 h, dissolved in anhyd. ACN (15 mL) and stirred for 30 min with freshly activated MS 4 Å (1.0 g) under an Ar atmosphere. After cooling to -15 °C, TMSOTf (8 µL, 34 µL, 0.05 equiv.) was added slowly. After stirring for 1 h at this temperature, a TLC analysis (Tol/EtOAc 9:1) revealed donor consumption and the presence of a new major spot (R_f 0.7). Et_3N (15 µL) was added and solids were filtered off. The filtrate was concentrated and the residue was purified by flash chromatography (cHex/EtOAc 90:10→85:15). Disaccharide **62** (490 mg, 397 µmol, 67%) was obtained as white solid. The coupling product had **62** had ^{1}H NMR ($CDCl_3$) δ 7.84-7.77 (m, 4H, H_{Ar}), 7.66-7.62 (m, 4H, H_{Ar}), 7.53-7.37 (m, 9H, H_{Ar}), 7.04-6.97 (m, 5H, H_{Ar}), 6.45 (d, 1H, $J_{2,NH}$ = 7.2 Hz, NH_{B}), 5.85-5.76 (m, 1H, CH_{All}), 5.67 (s, 1H, $CHCl_2$), 5.40 (d, 1H, $J_{1,2}$ = 7.2 Hz, H-1_{A}), 5.23-5.17 (m, 1H, CH_{2All}), 5.15-5.11 (m, 1H, CH_{2All}), 4.96 (d, 1H, J = 12.6 Hz, CH_{2Nap}), 4.82 (d_{po}, 1H, CH_{2Nap}), 4.82 (dd_{po}, 1H, $J_{2,3}$ = 11.1 Hz, H-2_{A}), 4.62 (d_{po}, 1H, $J_{1,2}$ = 8.4 Hz, H-1_{B}), 4.59 (d, 1H, J = 12.4 Hz, CH_{2Bn}), 4.48 (dd, 1H, $J_{3,4}$ = 3.6 Hz, $J_{2,3}$ = 10.8 Hz, H-3_{B}), 4.34 (pdt, 1H, $J_{4,5}$ = 3.2 Hz, H-5_{A}), 4.29 (dd_{po}, 1H, $J_{3,4}$ = 3.5 Hz, H-3_{A}), 4.27-4.22 (m, 1H, CH_{2All}), 4.12 (pt, H-4_{A}), 4.08 (d, 1H, CH_{2Bn}), 3.99-3.94 (m, 1H, CH_{2All}), 3.84 (dd, 1H, $J_{6a,6b}$ = 10.9 Hz, $J_{5,6a}$ = 6.3 Hz, H-$6a_{A}$), 3.80 (brd_{po}, 1H, H-4_{B}), 3.78 (dd_{po}, 1H, $J_{5,6b}$ = 6.2 Hz, H-$6b_{A}$), 3.50 (ddd_{po}, 1H, H-2_{B}), 4.47 (dq_{po}, 1H, $J_{4,5}$ = 1.1 Hz, H-5_{B}), 1.20 (d, 3H, $J_{5,6}$ = 6.3 Hz, H-6_{B}), 1.03 (s, 9H, CH_{3TBDPS}). ^{13}C NMR ($CDCl_3$) δ 163.9 (CO_{NTCA}), 163.3 (CO_{NTCP}), 139.8, 137.7, 135.7, 133.2, 133.0, 132.9, 129.5, 127.2 ($C_{q,Ar}$), 133.6 (CH_{All}), 135.6, 135.5, 129.9, 128.1, 128.0, 127.9 (2C), 127.8, 127.6, 127.5, 127.4, 126.5, 126.0, 125.9, 125.8 (C_{Ar}), 117.7 (CH_{2All}), 98.0 (C-

1B, $^1J_{C,H}$ = 163 Hz), 97.8 (C-1A, $^1J_{C,H}$ = 170 Hz), 76.1 (C-5A), 75.6 (C-3B), 73.9 (C-3A), 72.7 (CH_{2Nap}), 72.1 (CH_{2Bn}), 71.5 (C-4A), 69.9 (CH_{2All}), 69.1 (C-5B), 66.3 (CCl_3), 65.1 (C-4B), 62.9 (C-6A), 54.6 (C-2B), 54.3 (C-2A), 26.8 (CH_{3TBDPS}), 19.2 (C_{TBDPS}), 17.2 (C-6B). HRMS (ESI$^+$): *m/z* $[M+NH_4]^+$ calcd for $C_{59}H_{61}Cl_6N_6O_{10}Si$ 1251.2350; found *m/z* 1251.2330.

Allyl 2-acetamido-3-*O*-benzyl-2-deoxy-4-*O*-(2-naphthylmethyl)-6-*O*-*tert*-butyldiphenylsilyl--α-L-altropyranosyl-(1→3)-4-azido-2-dichloroacetamido-2,4,6-trideoxy-β-D-galactopyranoside (63). Ethylenediamine (75 µL, 1.13 mmol, 4.0 equiv.) was added to disaccharide **62** (350 mg, 284 µmol, 1.0 equiv.) in THF/MeOH (1:1, 14 mL). The reaction mixture was heated at 50 °C for 60 h at which point, a TLC analysis (Tol/EtOAc 7:3) revealed the consumption of the starting **62** (R_f 0.95) and the presence of a new spot (R_f 0.1). After cooling to rt, Et_3N (0.5 mL) was added followed by Ac_2O (268 µL, 2.8 mmol, 10.0 equiv.). A TLC analysis (Tol/EtOAc 6:4) showed the presence of a new spot (R_f 0.4). Solids were filtered off and washed with DCM (5 mL) twice. The filtrate was concentrated and the crude product was purified by column chromatography (cHex/EtOAc 3:1→2:1). Disaccharide **63**, obtained as a white solid (250 mg, 247 µmol, 87%), had ^{1}H NMR ($CDCl_3$) δ 7.85-7.63 (m, 8H, H_{Ar}), 7.51-7.18 (m, 15H, H_{Ar}), 6.68 (m, 1H, $J_{2,NH}$ = 7.2 Hz, NHB), 5.94 (s, 1H, $CHCl_2$), 5.91-5.82 (m, 1H, CH_{All}), 5.29 (d_o, 1H, $J_{2,NH}$ = 7.7 Hz, NHA), 5.28-5.24 (m_{po}, 1H, CH_{2All}), 5.20-5.16 (m, 1H, CH_{2All}), 4.88 (d, 1H, $J_{1,2}$ = 8.4 Hz, H-1B), 4.77 (d, 1H, $J_{2,NH}$ = 12.2 Hz, CH_{2Nap}), 7.73 (d_o, 1H, $J_{1,2}$ = 3.8 Hz, H-1A,), 4.72 (d_{po}, 1H, CH_{2Nap}), 4.71 (d_o, 1H, CH_{2Bn}), 4.55 (d, 1H, J = 12.2 Hz, CH_{2Bn}), 4.47-4.39 (m, 3H, H-2A, H-3B, H-5A), 4.34-4.29 (m, 1H, CH_{2All}), 4.07-4.02 (m, 1H, CH_{2All}), 3.99 (dd_{po}, 1H, $J_{5,6a}$ = 2.7 Hz, $J_{6a,6b}$ = 11.1 Hz, H-6aA,), 3.95 (dd_{po}, 1H, $J_{5,6b}$ = 4.8 Hz, H-6bA), 3.91 (dd, 1H, $J_{2,3}$ = 3.6 Hz, H-3A), 3.65 (dd, 1H, $J_{3,4}$ = 3.6 Hz, $J_{4,5}$ = 8.8 Hz, H-4A), 3.57 (brd, 1H, $J_{3,4}$ = 3.4 Hz, H-4B), 3.53 (dq, 1H, $J_{4,5}$ = 1.1 Hz, H-5B), 3.43 (ddd, 1H, $J_{2,3}$ = 10.8 Hz, H-2B), 1.78 (s, 3H, CH_{3Ac}), 1.18 (d, 3H, $J_{5,6}$ = 6.3 Hz, H-6B), 1.08 (s, 9H, CH_{3TBDPS}).^{13}C NMR ($CDCl_3$) δ 169.3 (CO_{NDCA}), 164.7 (CO_{NAc}), 138.8, 135.2, 133.5 (2C), 133.1, 133.0 ($C_{q,Ar}$), 133.7 (CH_{All}), 135.7, 135.6, 12.7, 129.0,128.2 (2C), 127.8, 127.7, 127.6 (2C), 126.7, 126.1, 125.9, 125.9 (C_{Ar}), 117.67 (CH_{2All}), 101.5 (C-1A, $^1J_{C,H}$ = 170 Hz), 97.8 (C-1B, $^1J_{C,H}$ = 163 Hz), 76.5 (C-3B), 72.5 (C-3A), 70.6 (C-4A), 71.4 (CH_{2Nap}), 70.6 (CH_{2Bn}), 70.0 (CH_{2All}), 69.8 (C-5A), 69.6 (C-5B), 66.5 ($CHCl_2$), 65.6 (C-4B), 63.7 (C-6A), 55.3 (C-2B), 49.8 (C-2A), 27.0 (CH_{3TBDPS}), 23.1 (CH_{3Ac}), 19.4 (C_{TBDPS}), 17.1 (C-6B). HRMS (ESI$^+$): *m/z* $[M+H]^+$ calcd for $C_{53}H_{62}Cl_2N_5O_9Si$ 1010.3694; found 1010.3669.

Allyl 2-acetamido-3-*O*-benzyl-2-deoxy-4-*O*-(2-naphthylmethyl)-α-L-altropyranosyl-(1→3)-4-azido-2-dichloroacetamido-2,4,6-trideoxy-β-D-galactopyranoside (64). TBAF (83 mg, 266 µmol, 1.2 equiv.) was added to disaccharide **63** (220 mg, 221 µmol, 1.0 equiv.) in anhyd. THF (10 mL) at rt. After 2 h, TLC monitoring (EtOAc), showed reaction completion and the presence of a more spot (R_f 0.1). Acetic acid (27 µL, 266 µmol, 1.2 equiv.) was added. Volatiles were removed under reduced pressure. The residue was purified by flash chromatography (EtOAc/MeOH 100:0→85:15) to give alcohol **64** as a white solid (135 mg, 175 µmol, 80%). Disaccharide **64** had ^{1}H NMR (DMSO-d_6) δ 8.57 (d, 1H, $J_{2,NH}$ = 9.2 Hz, NHB), 7.94-7.82 (m, 4H, H_{Ar}, NHA), 7.76 (brs, 1H, H_{Ar}), 7.51-7.49 (m, 2H, H_{Ar}), 7.42-7.39 (m, 3H, H_{Ar}), 7.32-7.25 (m, 3H, H_{Ar}), 6.44 (s, 1H,

$CHCl_2$), 5.84-5.76 (m, 1H, CH_{All}), 5.26-5.21 (m, 2H, CH_{2All}), 5.12-5.09 (m, 1H, CH_{2All}), 4.76 (brs, 1H, H-1_A), 4.71 (d, 1H, J = 11.6 Hz, CH_{2Nap}), 4.66-4.61 (m, 2H, CH_{2Bn}, OH), 4.55 (d, 1H, J = 11.6 Hz, CH_{2Bn}), 4.50 (d_{po}, 1H, CH_{2Nap}), 4.48 (d, 1H, $J_{1,2}$ = 8.0 Hz, H-1_B), 4.28-4.15 (m, 3H, H-2_A, H-5_A, CH_{2All}), 4.03-3.94 (m, 3H, H-3_B, H-4_B, CH_{2All}), 3.82-3.69 (m, 5H, H-2_B, H-5_B, H-3_A, H-4_A, H-$6a_A$), 3.56-3.50 (m, 1H, H-$6b_A$), 1.78 (s, 3H, CH_{3Ac}), 1.23 (d, 3H, $J_{5,6}$ = 6.4 Hz, H-6_B). ^{13}C NMR (DMSO-d_6) δ 169.3 (CO_{NDCA}), 164.2 (CO_{NAc}), 139.3, 136.5, 133.2, 132.9 ($C_{q,Ar}$), 134.9 (CH_{All}), 128.3, 128.2, 128.1, 128.0, 127.5, 126.5, 126.4, 126.3 (2C) (C_{Ar}), 116.8 (CH_{2All}), 101.8 (C-1_A, $^1J_{C,H}$ = 169 Hz), 100.1 (C-1_B, $^1J_{C,H}$ = 161 Hz), 77.7 (C-3_B), 73.6 (C-3_A), 72.6 (C-4_A), 70.9 (CH_{2Nap}), 70.3 (CH_{2Bn}), 69.8 (C-5_A), 69.5 (C-5_B), 69.2 (CH_{2All}), 67.6 ($CHCl_2$), 65.4 (C-4_B), 61.5 (C-6_A), 52.4 (C-2_B), 49.5 (C-2_A), 22.9 (CH_{3Ac}), 17.6 (C-6_B). HRMS (ESI$^+$): *m/z* 772.2502 (calcd for $C_{37}H_{43}Cl_2N_5O_9H$ $[M+H]^+$ *m/z* 772.2516).

Allyl (benzyl 2-acetamido-3-*O*-benzyl-6-*O*-benzyl-4-*O*-(2-naphthylmethyl)-2-deoxy-α-L-altropyranosyluronate)-(1→3)-4-azido-2-dichloroacetamido-2,4,6-trideoxy-β-D-galactopyranoside (65). TEMPO (4 mg, 26 μmol, 0.2 equiv.), followed by BAIB (104 mg, 324 μmol, 2.5 equiv.), were added to a suspension of alcohol **64** (100 mg, 130 μmol, 1.0 equiv.) in DCM/water (2:1, 6.0 mL) at rt. After stirring vigorously for 2 h at rt, TLC monitoring (EtOAc/MeOH 20:1) showed the presence of a major polar spot and absence of the starting **64** (R_f 0.2). 50% Aq. Na_2SO_3 (5 mL) was added, the DCM layer was separated, and the water phase was extracted with chroroform/isopropanol (3:1, 10 mL) twice. The water phase was acidified with dilute aq. HCl to reach pH ~1 and again extracted with chroroform/isopropanol (3:1, 10mL) twice. The combined organic phases were washed with brine (50 mL), dried by passing through a phase separator filter and concentrated under reduced pressure. The crude thus obtained was dissolved in DMF (2.0 mL) rt and benzyl bromide (44 μL, 259 μmol, 2.0 equiv.) followed by K_2CO_3 (27 mg, 194 μmol, 1.5 equiv.) were added. After stirring for 4 h at rt, water (20 mL) was added. The aq. layer was washed with DCM (5.0 mL) three times. The combined DCM parts were washed with brine (20 mL), dried over Na_2SO_4, and concentrated under reduce pressure. The crude was purified by flash chromatography with (Tol/EtOAc 70:30→65:35). The benzyl ester **65**, obtained as an off-white solid (70 mg, 79 μmol, 61%), had R_f 0.2 (Tol/EtOAc 7:3). ^{1}H NMR ($CDCl_3$) δ 7.84-7.68 (m, 5H, H_{Ar}), 7.51-7.25 (m, 12H, H_{Ar}), 6.78 (d, $J_{2,NH}$ = 7.2 Hz, NH_B), 6.04 (s, 1H, $CHCl_2$), 5.91-5.81 (m, 2H, NH_A, CH_{All}), 5.37 (d, 1H, $J_{1,2}$ = 6.0 Hz, H-1_A), 5.28-5.13 (m_{po}, 4H, CH_{2All}, CH_{2Bn-6}), 4.75 (d, 1H, $J_{4,5}$ = 4.0 Hz, H-5_A), 4.73 (d_{po}, 1H, $J_{1,2}$ = 8.8 Hz, H-1_B,), 4.73 (d_{po}, 1H, CH_{2Nap}), 4.68 (d, 1H, J = 12.4 Hz, CH_{2Nap}), 4.51 (d_{po}, 1H, J = 12. 0 Hz, CH_{2Bn}), 4.48 (d_{po}, 1H, CH_{2Bn}), 4.47 (dd_{po}, $J_{3,4}$ = 3.8 Hz, $J_{2,3}$ = 10.9 Hz, H-3_B), 4.33-4.28 (m, 1H, CH_{2All}), 4.11-4.01 (m, 2H, H-3_A, H-4_A, CH_{2All}), 3.95-3.90 (m, 2H, H-2_A, H-4_B), 3.52 (pdt, 1H, H-2_B), 3.43 (q, 1H, H-5_B), 1.88 (s, 3H, CH_{3Ac}), 1.24 (d, 3H, $J_{5,6}$ = 6.4 Hz, H-6_B). ^{13}C NMR ($CDCl_3$), δ170.6 (C-6_A), 169.3 (CO_{NDCA}), 164.3 (CO_{NAc}), 137.9, 134.9, 134.8, 133.1, 133.0 ($C_{q,Ar}$), 133.6 (CH_{All}), 133.1, 133.0 ($C_{q,Ar}$), 128.7 (2C), 128.6, 128.3, 128.2, 128.1, 127.9, 127.8, 127.6, 126.8, 126.1, 126.0, 125.9 (17C, C_{Ar}), 117.9 (CH_{2All}), 99.3 (C-1_A, $^1J_{C,H}$ = 170. Hz), 97.9 (C-1_B, $^1J_{C,H}$ = 162 Hz), 77.0 (C-3_B), 73.4 (C-3_A), 73.1 (C-4_A), 72.1 (C-5_A), 72.0 (2C, $CH_{2Bn,Nap}$), 70.1 (CH_{2All}), 69.2 (C-5_B), 67.5

(CH_{2Bn-6}), 66.7 ($CHCl_2$), 65.2 (C-4$_B$), 54.3 (C-2$_B$), 52.5 (C-2$_A$), 23.4 (CH_{3Ac}), 17.3 (C-6$_B$). HRMS (ESI$^+$): *m/z* [M+H]$^+$ calcd for $C_{44}H_{48}Cl_2N_5O_{10}$ 876.2778; found 876.2773.

Allyl (methyl 2-acetamido-3-*O*-benzyl-2-deoxy-4-*O*-(2-naphthylmethyl)-α-L-altropyranosyluronate)-(1→3)-4-azido-2-trichloroacetamido-2,4,6-trideoxy-β-D-galactopyranoside (S8). 25% Methanolic NaOMe (2.0 µL, 11 µmol, 0.5 equiv.) was added to the benzyl ester **47** (20 mg, 21 µmol, 1.0 equiv.) dissolved in anhyd. MeOH (2.6 mL). After stirring at rt overnight, a TLC (Tol/EtOAc 7:3) follow up indicated reaction completion. AcOH (~2 µL) was added and volatiles were evaporated under reduced pressure. The residue was purified by flash chromatography (Tol/EtOAc 60:40→55:45) to give the methyl ester **S8** (13 mg, 17.7 µmol, 74%) as a white solid. The later had R_f 0.35 (Tol/EtOAc 6:4). ^{1}H NMR ($CDCl_3$) δ 7.85-7.70 (m, 4H, H_{Ar}), 7.54-7.29 (m, 9H, H_{Ar}), 6.86 (d, $J_{2,NH}$ = 7.2 Hz, NH$_B$), 5.90-5.80 (m, 1H, CH_{All}), 5.56 (d, 1H, $J_{2,NH}$ = 7.6 Hz, NH$_A$), 5.27-5.23 (m, 1H, CH_{All}), 5.20-5.16 (m$_{po}$, 2H, CH_{2All}), 5.20 (d$_{po}$, $J_{1,2}$ = 4.0 Hz, H-1$_A$), 4.83 (d$_{po}$, 1H, $J_{1,2}$ = 8.4 Hz, H-1$_B$), 4.82 (d$_{po}$, 1H, $J_{4,5}$ = 3.6 Hz, H-5$_A$), 4.73-4.69 (m$_{po}$, 2H, CH_{2Nap}), 4.63-4.51 (brs, 2H, CH_{2Bn}, H-3$_B$), 4.35-4.31 (m, 1H, CH_{2All}), 4.12-4.01 (m, 5H, H-2$_A$, H-3$_A$, H-4$_A$, H-4$_B$, CH_{2All}), 3.77 (s, 3H, OCH_3), 3.75 (q$_{po}$, 1H, H-5$_B$), 3.63-3.57 (m, 1H, H-2$_B$), 1.87 (s, 3H, CH_{3Ac}), 1.39 (d, 3H, $J_{5,6}$ = 6.0 Hz, H-6$_B$). ^{13}C NMR ($CDCl_3$) δ 170.2, 170.1 (C-6$_A$, CO_{NAc}), 161.8 (CO_{NTCA}), 137.9, 134.9, 133.1, 133.0 ($C_{q,Ar}$), 133.5 (CH_{All}), 128.7, 128.3, 128.2, 128.1 (2C), 127.8, 127.7, 126.7, 126.1, 126.0, 125.8 (C_{Ar}), 117.9 (CH_{2All}), 99.7 (C-1$_A$, $^1J_{C,H}$ = 170 Hz), 97.8 (C-1$_B$, $^1J_{C,H}$ = 162 Hz), 92.4 (CCl_3), 76.3 (C-3$_B$), 73.2 (C-3$_A$), 72.8 (C-4$_A$), 71.7 (CH_{2Nap}), 71.6 (CH_{2Bn}), 71.3 (C-5$_A$), 70.1 (CH_{2All}), 69.5 (C-5$_B$), 65.1 (C-4$_B$), 55.2 (C-2$_B$), 52.5 (OCH_3), 51.8 (C-2$_A$), 23.4 (CH_{3Ac}), 17.4 (C-6$_B$). HRMS (ESI$^+$): *m/z* [M+H]$^+$ calcd for $C_{38}H_{42}Cl_3N_5O_{10}$ 834.2070; found 834.2059.

Allyl (methyl 3-*O*-benzyl-2-(*N*,*N*-diacetyl)amino-2-deoxy-α--altropyranosyluronate)-(1→3)-4-azido-2-trichloroacetamido-2,4,6-trideoxy-β-D-galactopyranoside (S9). K_2CO_3 (6.8 mg, 4.9 µmol, 0.2 equiv.) was added to alcohol **48** (20 mg, 24.7 µmol, 1.7 equiv.) in MeOH (1.5 mL) and the suspension was stirred at rt for 1 h. At completion as indicated by TLC analysis (Tol/EtOAc 1:1), AcOH (~3 µL, 4.9 µmol, 0.2 equiv.) was added and the mixture was concentrated under reduced pressure. The residue was purified by flash chromatography (Tol/EtOAc 4:1→3:1) to give the methyl ester **S9** (15 mg, 20.4 µmol, 82%). Disaccharide **S9** had R_f 0.25 (Tol/EtOAc 4:1). ^{1}H NMR ($CDCl_3$) δ 7.42-7.17 (m, 5H, H_{Ar}), 6.86 (d, 1H, $J_{2,NH}$ = 7.2 Hz, NH$_B$), 5.89-5.80 (m, 1H, CH_{All}), 5.64 (d, 1H, $J_{1,2}$ = 7.8 Hz, H-1$_A$), 5.27-5.22 (m, 1H, CH_{2All}), 5.18-5.15 (m, 1H, CH_{2All}), 4.86 (d, 1H, $J_{1,2}$ = 8.4 Hz, H-1$_B$), 4.75 (dd, 1H, $J_{3,4}$ = 3.6 Hz, $J_{2,3}$ = 10.8 Hz, H-3$_B$), 4.71 (d, 1H, $J_{4,5}$ = 2.0 Hz, H-5$_A$), 4.57 (d, 1H, J = 11.6 Hz, CH_{2Bn}), 4.51-4.50 (m, 1H, H-4$_A$), 4.47 (d, 1H, CH_{2Bn}), 4.37 (dd, 1H, $J_{2,3}$ = 10.8 Hz, $J_{3,4}$ = 3.2 Hz, H-3$_A$), 4.33-4.29 (m, 1H, CH_{All}), 4.11 (d, 1H, H-4$_B$), 4.06-4.00 (m, 2H, H-2$_A$, CH_{2All}), 3.85 (s, 3H, OCH_3), 3.75 (dq, 1H, $J_{4,5}$ = 1.2 Hz, H-5$_B$), 3.56-3.49 (m, 1H, H-2$_B$), 2.55 (d, 1H, $J_{4,OH}$ = 1.8 Hz, OH), 2.34 (brs, 6H, CH_{3Ac}), 1.36 (d, 3H, $J_{5,6}$ = 6.0 Hz, H-6$_B$). ^{13}C NMR ($CDCl_3$) δ 175.0 (br, 2C, CON_{Ac}), 168.9 (C-6$_A$), 162.1 (CO_{NTCA}), 136.8 ($C_{q,Ar}$), 133.5 (CH_{All}), 128.6, 128.4, 128.0 (C_{Ar}), 118.1 (CH_{2All}), 98.1 (C-1$_A$, $^1J_{C,H}$ = 175 Hz), 97.4 (C-1$_B$, $^1J_{C,H}$ = 163 Hz), 92.2 (CCl_3), 76.3 (C-3$_B$), 75.1 (C-5$_A$), 72.5 (C-3$_A$), 72.3 (CH_{2Bn}), 70.2

(CH_{2All}), 68.7 (C-5_B), 66.7 (C-4_A), 65.5 (C-4_B), 59.0 (C-2_A), 55.6 (C-2_B), 52.7 (OCH_3), 21.4 (CH_{3Ac}), 17.4 (C-6_B). HRMS (ESI^+): *m/z* $[M+NH_4]^+$ calcd for $C_{29}H_{40}Cl_3N_6O_{11}$ 753.1821; found 753.1811.

Allyl (methyl 2-acetamido-3-*O*-benzyl-2-deoxy-α-L-threohexopyranosiduronate)-(1→3)-4-azido-2-trichloroacetamido-2,4,6-trideoxy-β-D-galactopyranoside (S10). K_2CO_3 (4 mg, 32 µmol, 1.0 equiv.) was added to tetrasaccharide **53** (50 mg, 32 µmol, 1.0 equiv.) in MeOH (1.0 mL). After stirring at rt for 1 h, a TLC (Tol/EtOAc 1:1) follow up indicated completion and the reaction mixture was neutralized by gentle stirring with Dowex resin (H^+ form). Solids were filtered, washed with methanol (~2 mL) twice. Volatiles were eliminated and the residue was purified by flash chromatography (Tol/EtOAc 6:1→5:1) to give the elimation product **S10** (14 mg, 18.6 µmol, 63%). Disaccharide **S10** had R_f 0.35 (Tol/EtOAc 7:1). ^{1}H NMR ($CDCl_3$) δ 7.42-7.28 (m, 5H, H_{Ar}), 6.87 (d, 1H, $J_{2,NH}$ = 7.2 Hz, NH_B), 6.33 (dd, 1H, $J_{3,4}$ = 4.8 Hz, H-4_A), 5.88-5.80 (m, 1H, CH_{All}), 5.49 (d, 1H, $J_{2,NH}$ = 8.8 Hz, NH_A), 5.29 (d_{po}, 1H, $J_{1,2}$ = 2.8 Hz, H-1_A), 5.28-5.23 (m, 1H, CH_{2All}), 5.20-5.16 (m, 1H, CH_{2All}), 4.92 (d, 1H, $J_{1,2}$ = 8.4 Hz, H-1_B), 4.80 (dd_{po}, 1H, $J_{3,4}$ = 3.6 Hz, $J_{2,3}$ = 10.8 Hz, H-3_B), 4.78 (d_{po}, 1H, CH_{2Bn}), 4.73 (d, 1H, J = 12.4 Hz, CH_{2Bn}), 4.46-4.43 (m, 1H, H-2_A), 4.35-4.30 (m, 1H, CH_{All}), 4.08-4.03 (m, 1H, CH_{2All}), 3.94-3.92 (m, 2H, H-4_B, H-3_A), 3.85 (s, 3H, OCH_3), 3.79-3.74 (dq_{po}, 1H, $J_{4,5}$ = 1.2 Hz, H-5_B), 3.50-3.43 (m, 1H, H-2_B), 1.94 (brs, 3H, CH_{3Ac}), 1.38 (d, 3H, $J_{5,6}$ = 6.4 Hz, H-6_B). ^{13}C NMR ($CDCl_3$) δ 168.9 (C-6_A), 162.6 (CO_{NAc}), 162.2 (CO_{NTCA}), 140.4 (C-5_A), 138.4 ($C_{q,Ar}$), 133.5 (CH_{All}), 128.3, 127.6, 127.5 (C_{Ar}), 117.8 (CH_{2All}), 111.4 (C-4_A), 100.1 (C-1_A, $^1J_{C,H}$ = 176 Hz), 97.4 (C-1_B, $^1J_{C,H}$ = 164 Hz), 92.2 (CCl_3), 77.5 (C-3_B), 70.5 (CH_{2Bn}), 70.3 (C-3_A), 70.1 (CH_{2All}), 69.3 (C-5_B), 65.9 (C-4_B), 55.5 (C-2_B), 52.6 (OCH_3), 49.2 (C-2_A), 23.1 (CH_{3Ac}), 17.4 (C-6_B). HRMS (ESI^+): *m/z* $[M+H]^+$ calcd for $C_{27}H_{32}Cl_3N_5O_9S$ 676.1344; found 676.1344.

Propyl (2-acetamido-2-deoxy-α-L-altropyranosyluronic acid)-(1→3)-2-acetamido-4-amino-2,4,6-trideoxy-β-D-galactopyranoside (1).[1] *Route 1.* TFA (15 µL, 197 µmol, 4 equiv.) was added to a solution of disaccharide **57** (46 mg, 49 µmol, 1.0 equiv.) in DCM (2 mL) at rt. After 3-4 h, a TLC follow up with Tol/EtOAc (4:1) showed the presence of a more polar single spot. Dilute aq. $NaHCO_3$ (5 mL) was added followed by DCM (5 mL). The DCM layer was separated, dried over Na_2SO_4, filtered, and concentrated under reduced pressure. 20% $Pd(OH)_2$/C (68 mg) was added to a solution of the crude intermediate in *t*BuOH/DCM/H_2O (16 mL, 7:3:1) and after extensive degassing, the atmosphere was saturated with hydrogen. After stirring under hydrogen for 48 h, the suspension was passed through a 0.2 µm filter and washed thoroughly with methanol. Volatiles were evaporated and the crude intermediate was dissolved in water (5 mL) and lyophilized. Purification by semi-preparative RP-HPLC gave the known propyl glycoside **1** as a white solid (16 mg, 35 µmol, 71%).

Route 2. 20% $Pd(OH)_2$/C (100 mg) was added to a solution of **47** (50 mg, 53 µmol, 1.0 equiv.) in *t*BuOH/DCM/H_2O (17 mL, 20:5:2, v/v/v). After stirring for 48 h in a hydrogen atmosphere. The suspension was passed through a 0.2 µm filter and washed thoroughly with methanol. The filtrate was concentrated and the crude product was dried under vaccum. The obtained white powder was

dissolved in methanol (5 mL) and hydroxylamine (3.7 mg, µmol, 1.0 equiv.) was added. Monitoring by LCMS revealed the full consumption of the mono-acetate product and the presence of the desired product (LCMS: $[M+H]^+$ *m/z* 867.2) after 4 h. Phosphate buffer pH 7 was added with frequent pH monitoring to achieve pH 7. The mixture was diluted with water (10 mL) and lyophilized. After freeze-drying, purification of the crude material by semi-preparative RP-HPLC gave the known propyl glycoside[1] **1** as a white solid (14 mg, 30 µmol, 57%). Disaccharide **1** had RP-HPLC (215 nm) Rt = 13.9 min (conditions B), Rt = 12.2 min (conditions C). HRMS (ESI^+): *m/z* calcd for $C_{19}H_{35}N_3O_9Na$ $[M+Na]^+$ *m/z* 486.2064; found 486.2067.

Propyl (2-acetamido-2-deoxy-α-L-altropyranosyluronic acid)-(1→3)-(2-acetamido-4-amino-2,4,6-trideoxy-β-D-galactopyranosyl)-(1→4)-(2-acetamido-2-deoxy-α-L-altropyranosyluronic acid)-(1→3)-(2-acetamido-4-amino-2,4,6-trideoxy-β-D-galactopyranoside (2). Tetrasaccharide **61** (15 mg, 8.2 µmol, 1.0 equiv.), contaminated to a 15-20% extent by the disaccharide partners, was dissolved in DCM (1.0 mL) and TFA (15 µL, 197 µmol, 24 equiv.) was added. After stirring for 3 h at rt, a TLC follow up (Tol/EtOAc 2:1) indicated reaction completion. 10 % Aq. $NaHCO_3$ (5 mL) and DCM (5 mL) were added. The organic layer was separated, dried over Na_2SO_4, filtered, and concentrated under reduced pressure. The crude was dissolved in *t*BuOH/DCM/H_2O (11 mL, 7:3:1) and 20% $Pd(OH)_2$/C (50 mg) was added. After stirring under an atmosphere of hydrogen for 48 h, the suspension was passed through a syringe filter (0.2 µm) and washed thoroughly with methanol. The filtrate was evaporated and the crude was dissolved in water (5 mL) and lyophilized. The residue was purified by semi-preparative RP-HPLC to give the propyl glycoside **2** as a white foam (2.6 mg, 3.0 µmol, 37% (underestimated)). Analytical data for tetrasaccharide **2** were as described in the main text.

V. References

[1] H. B. Pfister, L. A. Mulard, *Org. Lett.* **2014**, *16*, 4892-4895.

VI. ^{1}H and ^{13}C spectra for compounds 2-4, 9-11, 14-19, 17, 21-32, 34-41, 47-65, S1-S4, S7-10

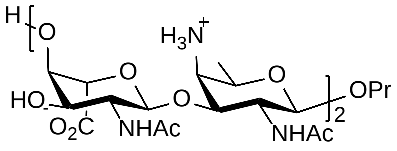

2

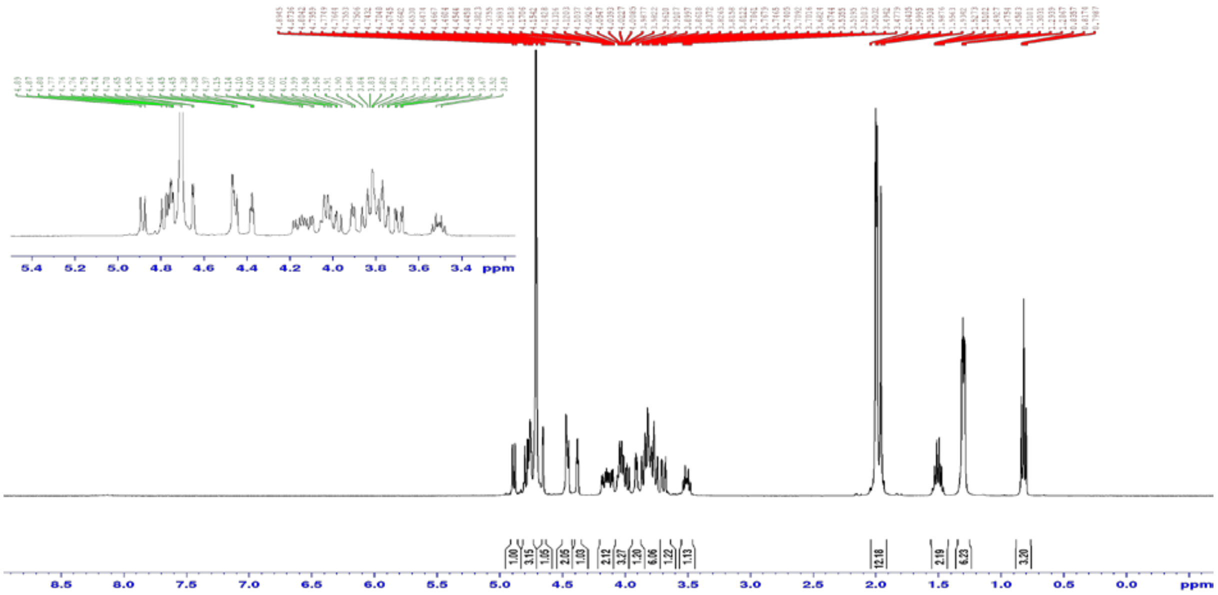

Figure. ^{1}H NMR (D_2O, 400 MHz) spectrum of **2**.

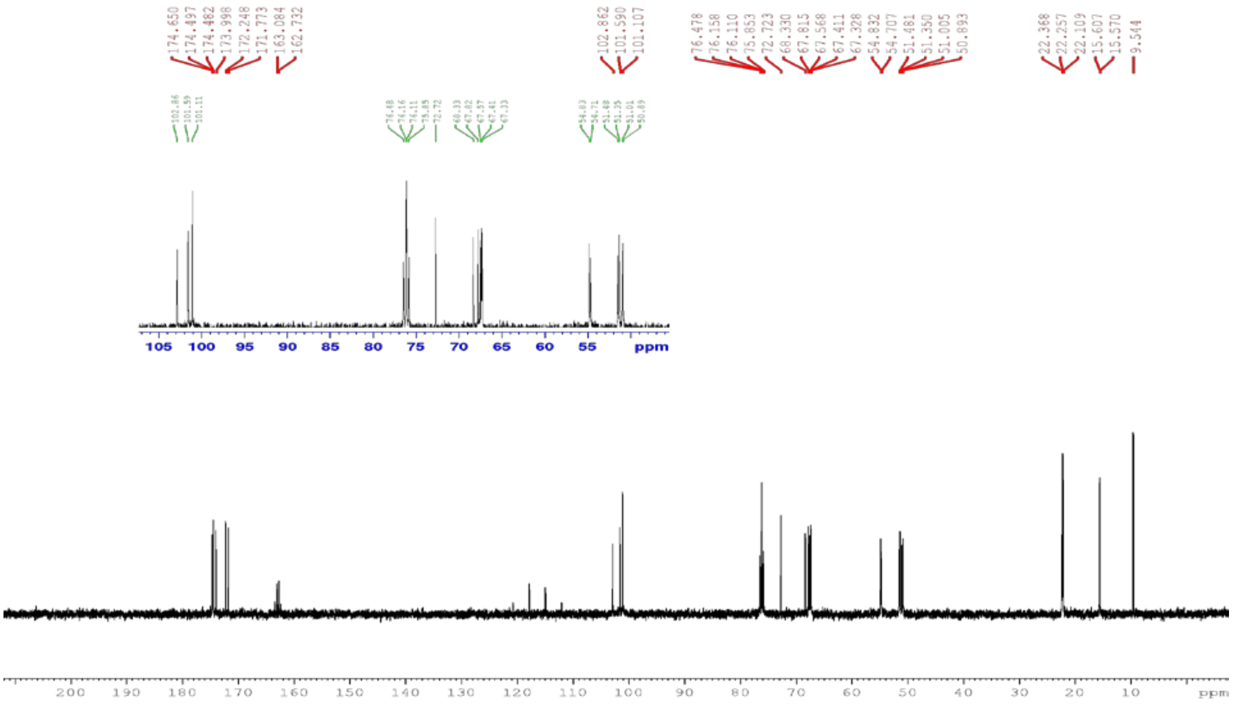

Figure. ^{13}C NMR (D_2O, 400 MHz) spectrum of **2**.

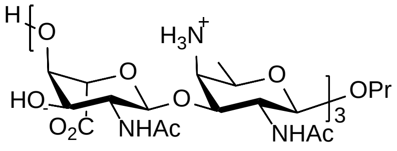

3

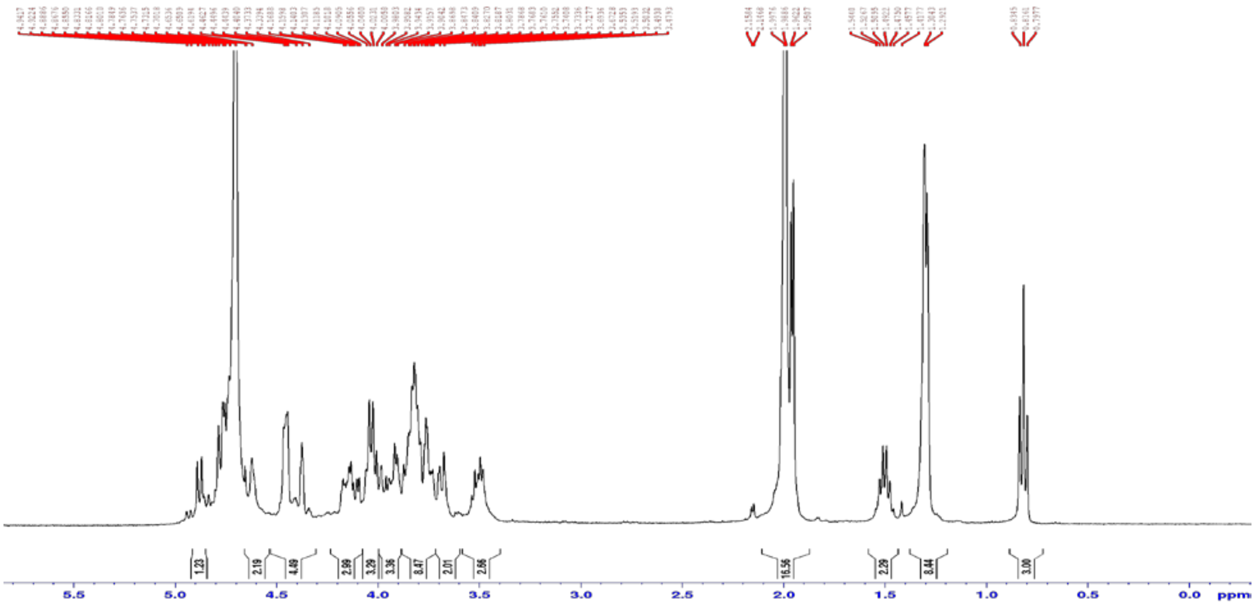

Figure. ^{1}H NMR (D_2O, 400 MHz) spectrum of **3**.

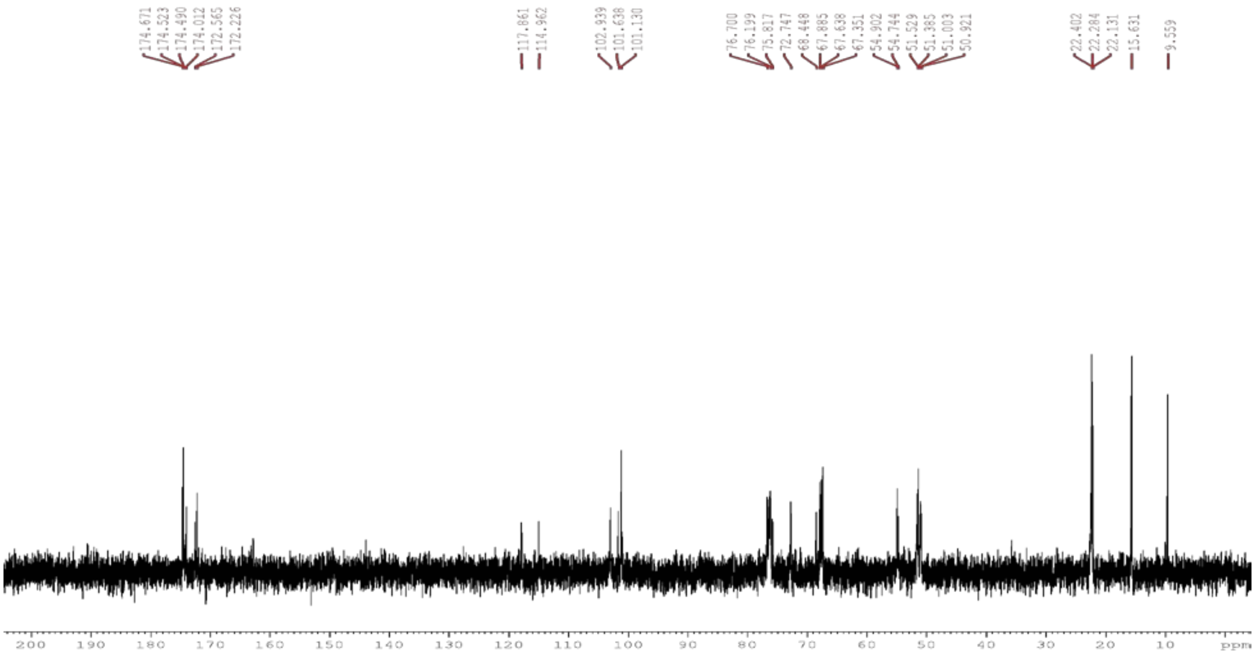

Figure. ^{13}C NMR (D_2O, 400 MHz) spectrum of **3**.

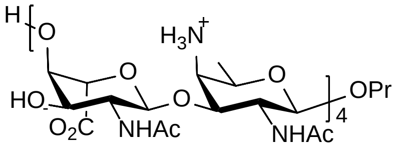

4

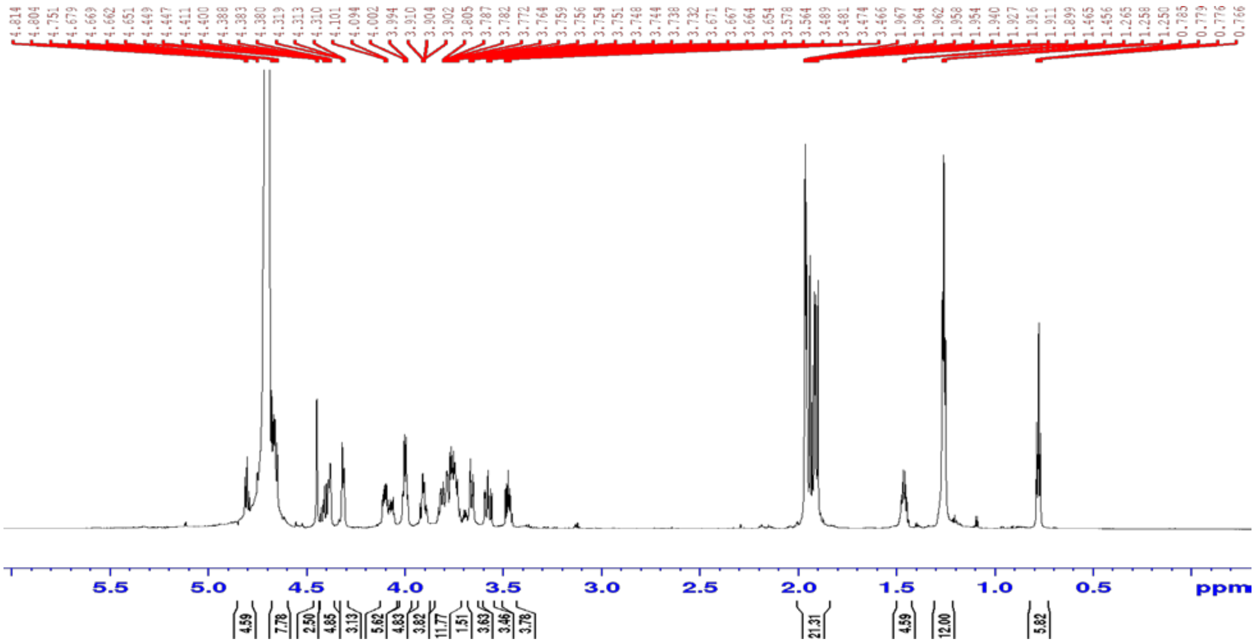

Figure. ^{1}H NMR (D_2O, 800 MHz) spectrum of **4** (with trifluoroacetate as counter ion).

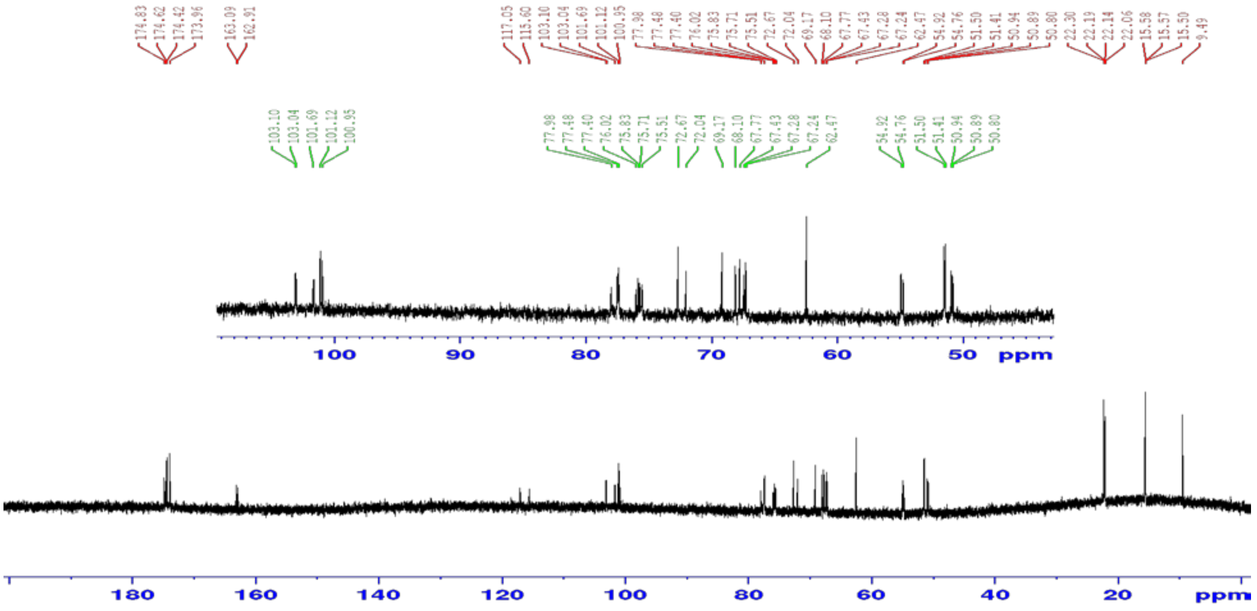

Figure. ^{13}C NMR (D_2O, 800 MHz) spectrum of **4** (with trifluoroacetate as counter ion)

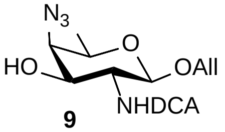

9

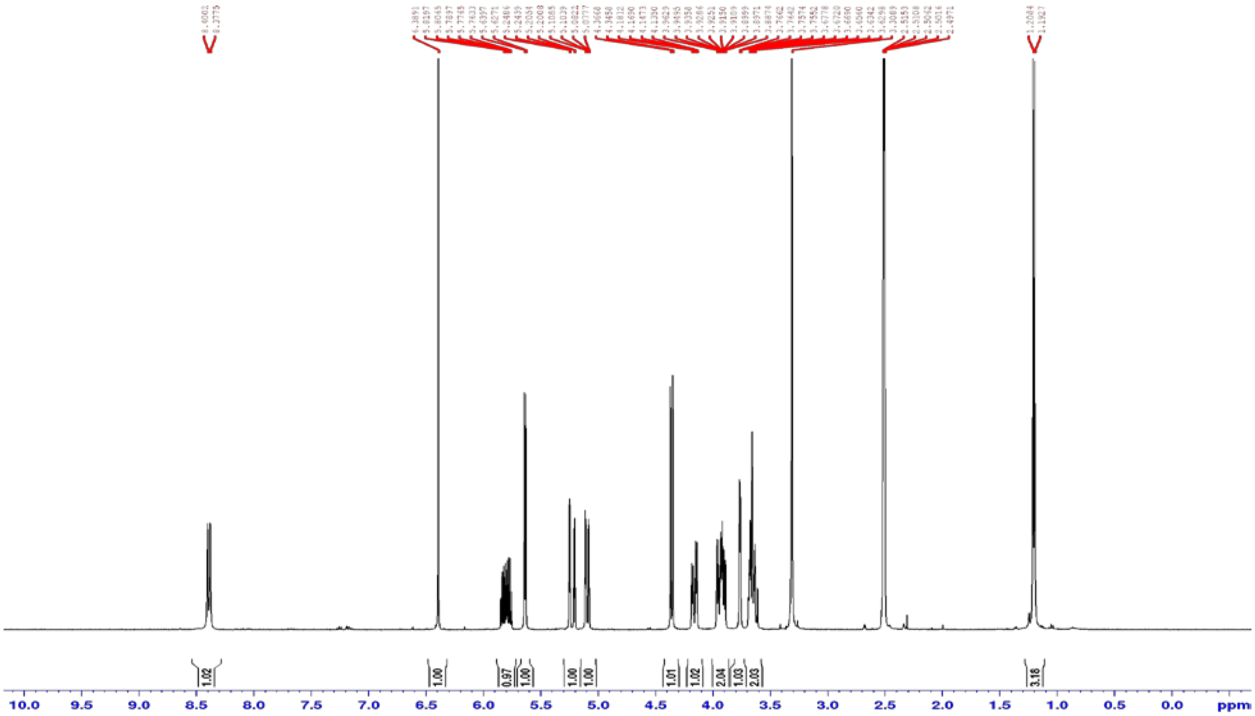

Figure. ^{1}H NMR (DMSO-d_6, 400 MHz) spectrum of **9**.

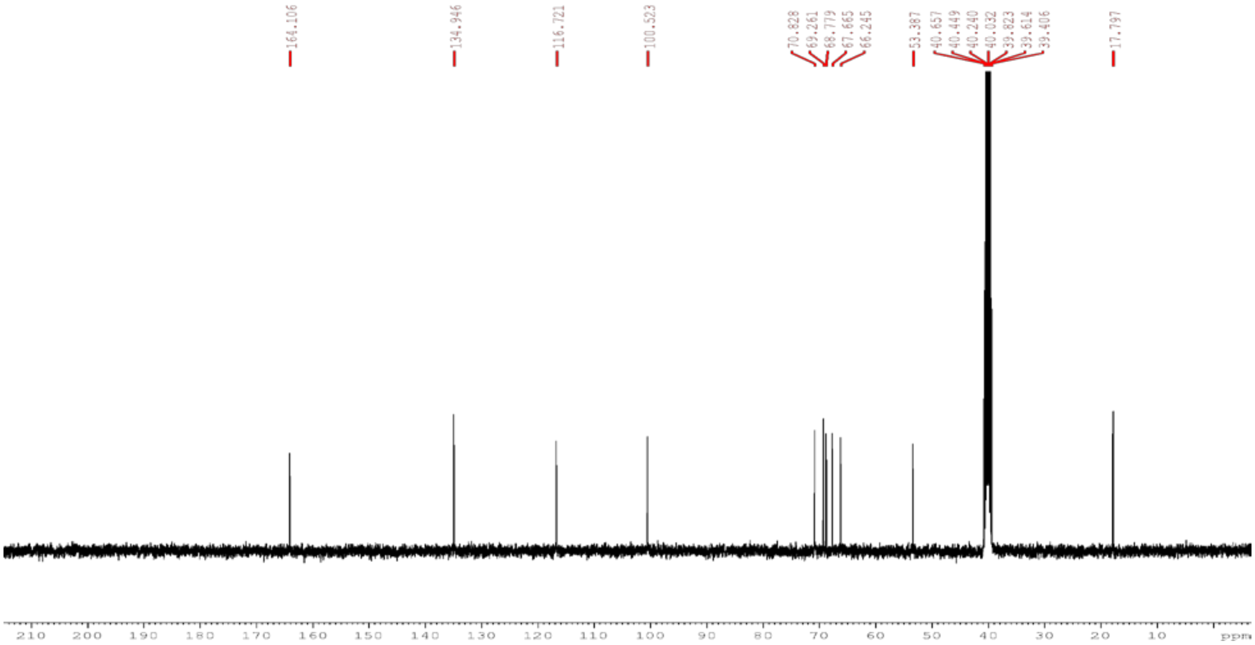

Figure. ^{13}C NMR (DMSO-d_6, 400 MHz) spectrum of **9**.

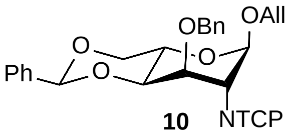

10

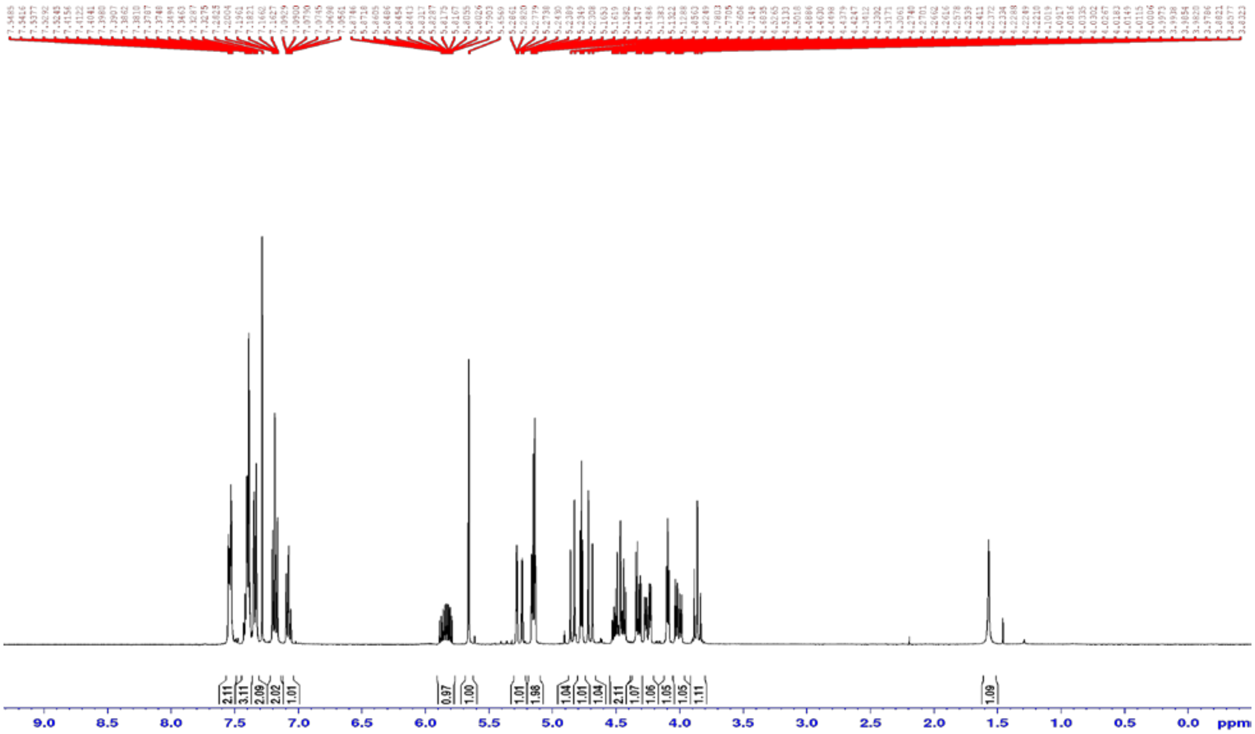

Figure. ^{1}H NMR ($CDCl_3$, 400 MHz) spectrum of **10**.

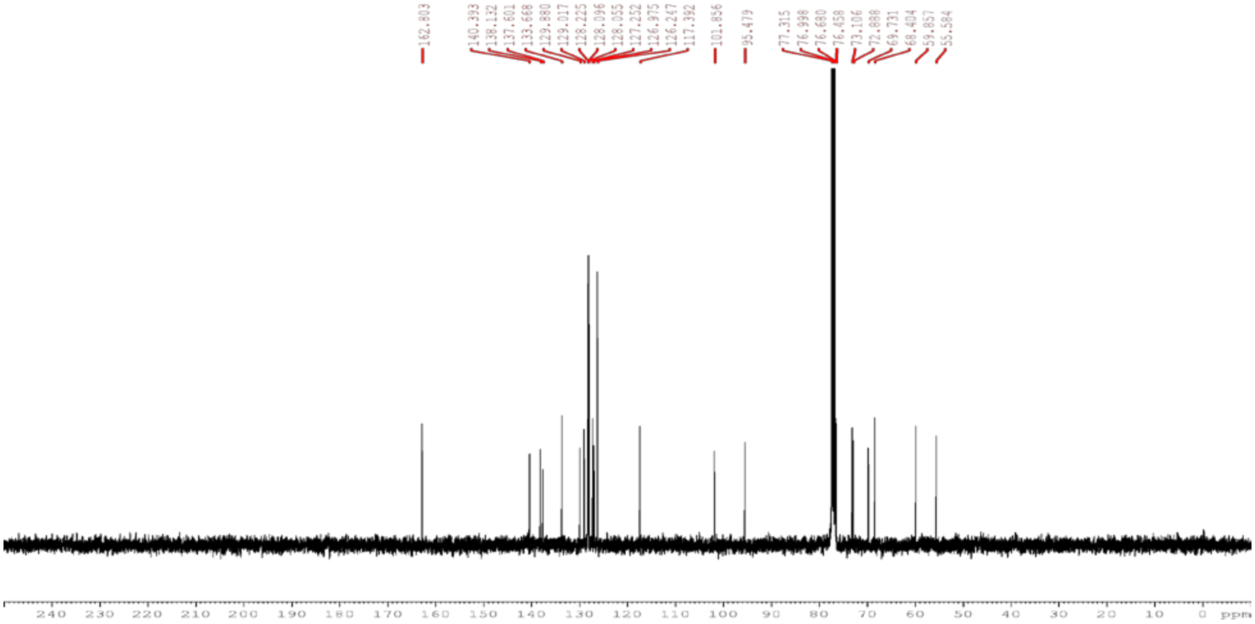

Figure. ^{13}C NMR ($CDCl_3$, 400 MHz) spectrum of **10**.

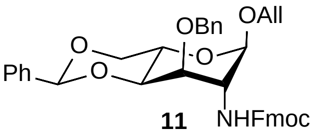

11

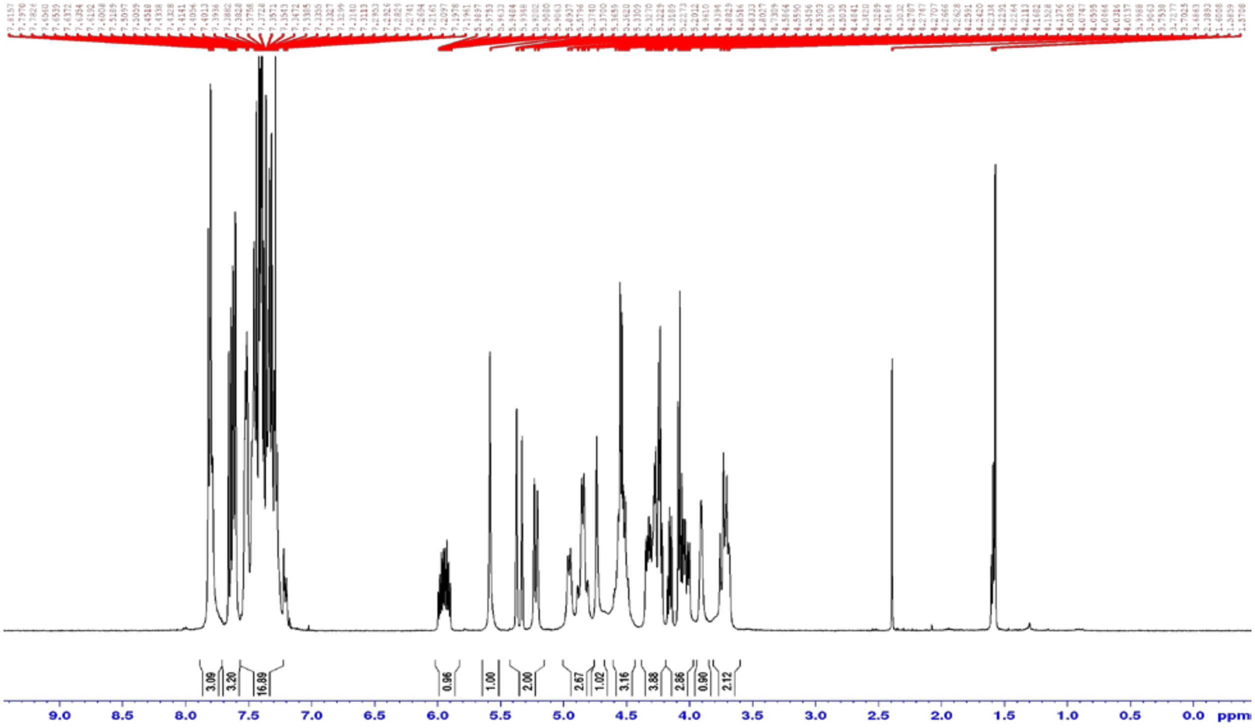

Figure. ^{1}H NMR ($CDCl_3$, 400 MHz) spectrum of **11**.

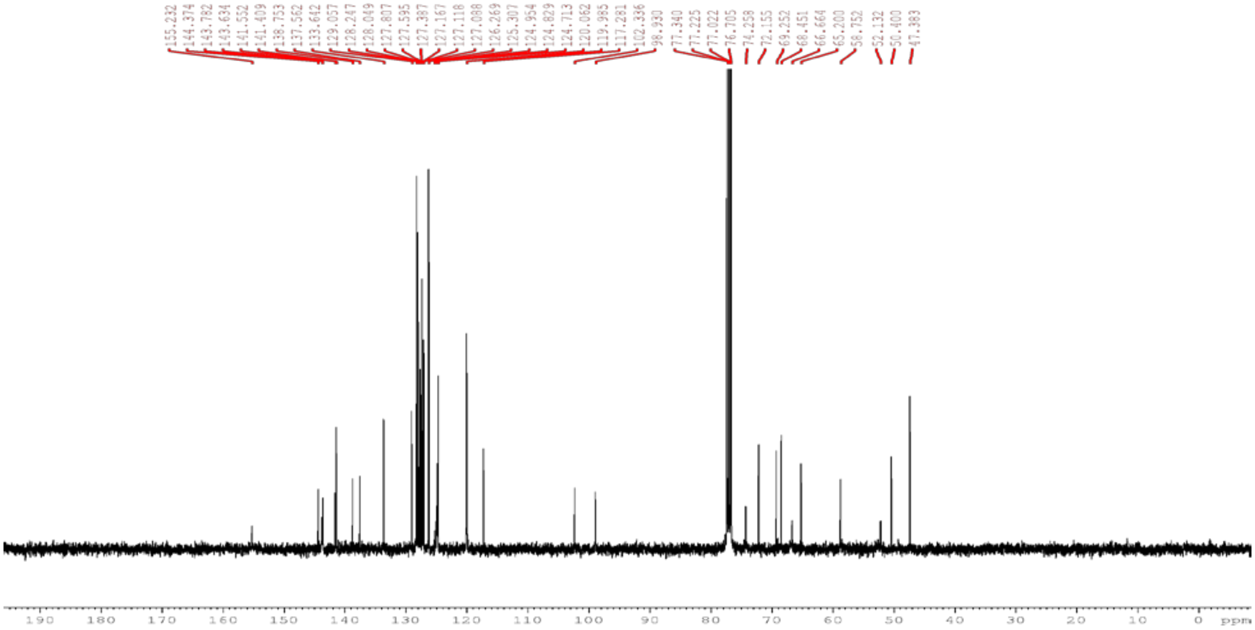

Figure. ^{13}C NMR ($CDCl_3$, 400 MHz) spectrum of **11**.

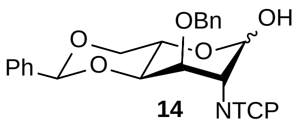

14

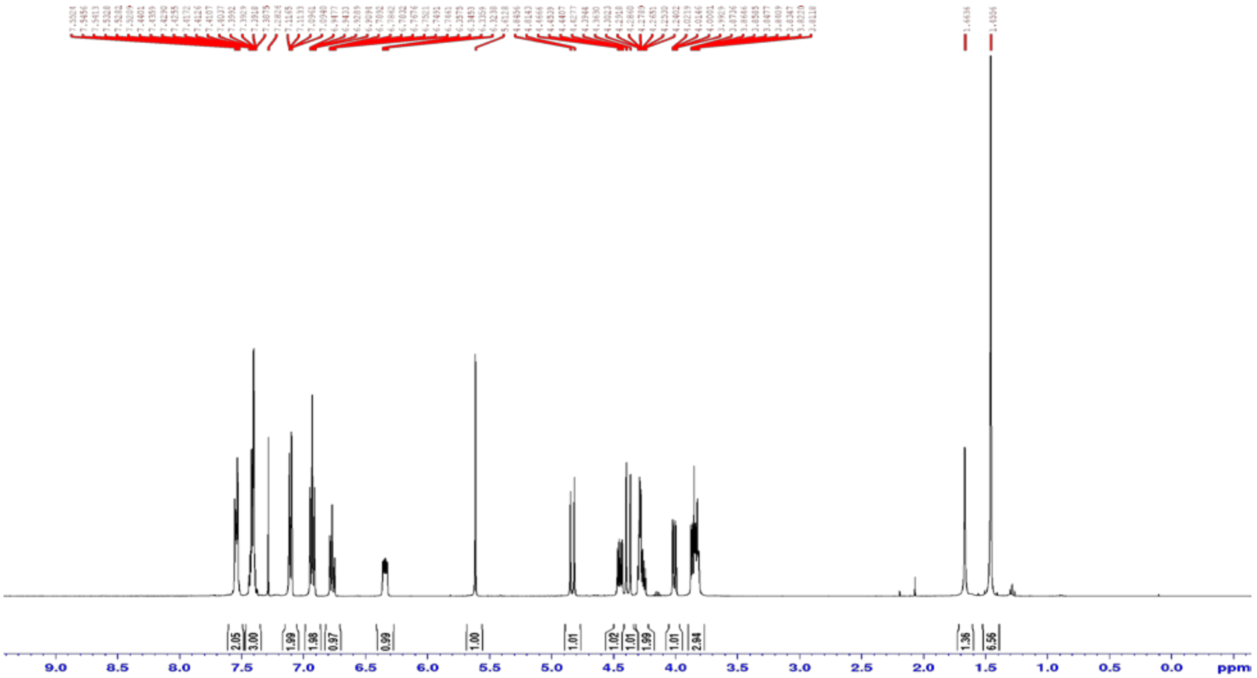

Figure. ^{1}H NMR ($CDCl_3$, 400 MHz) spectrum of **14**.

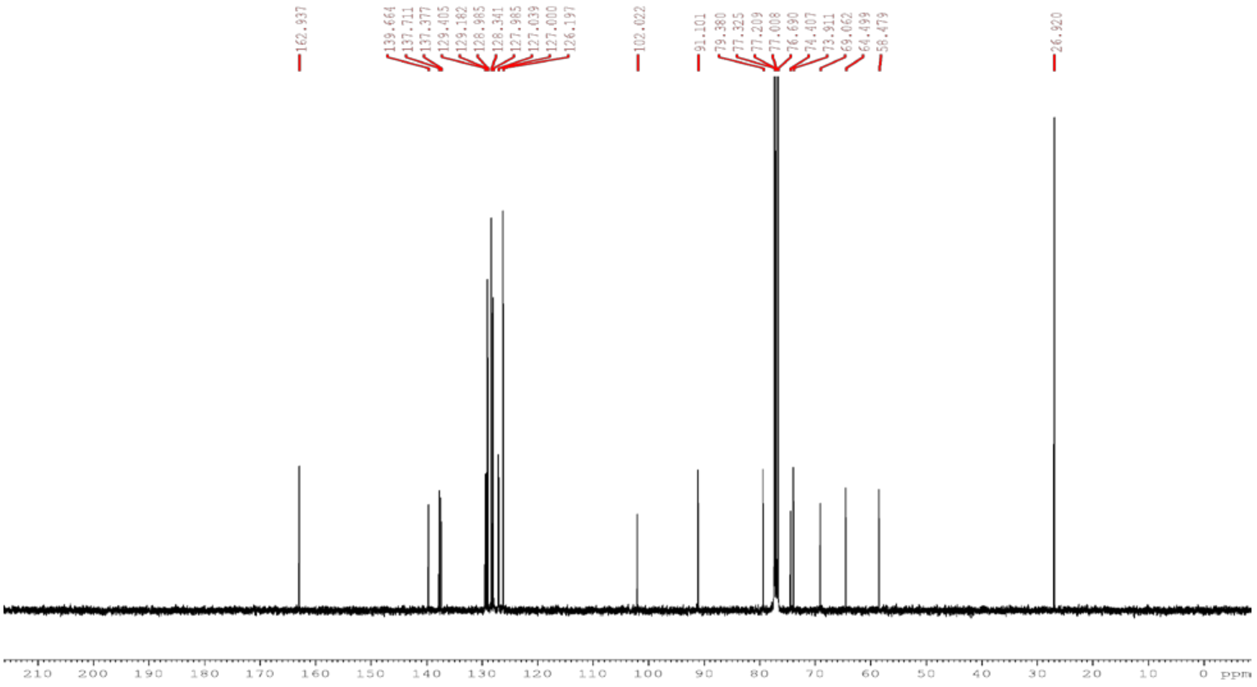

Figure. ^{13}C NMR ($CDCl_3$, 400 MHz) spectrum of **14**.

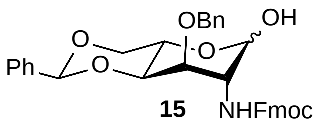

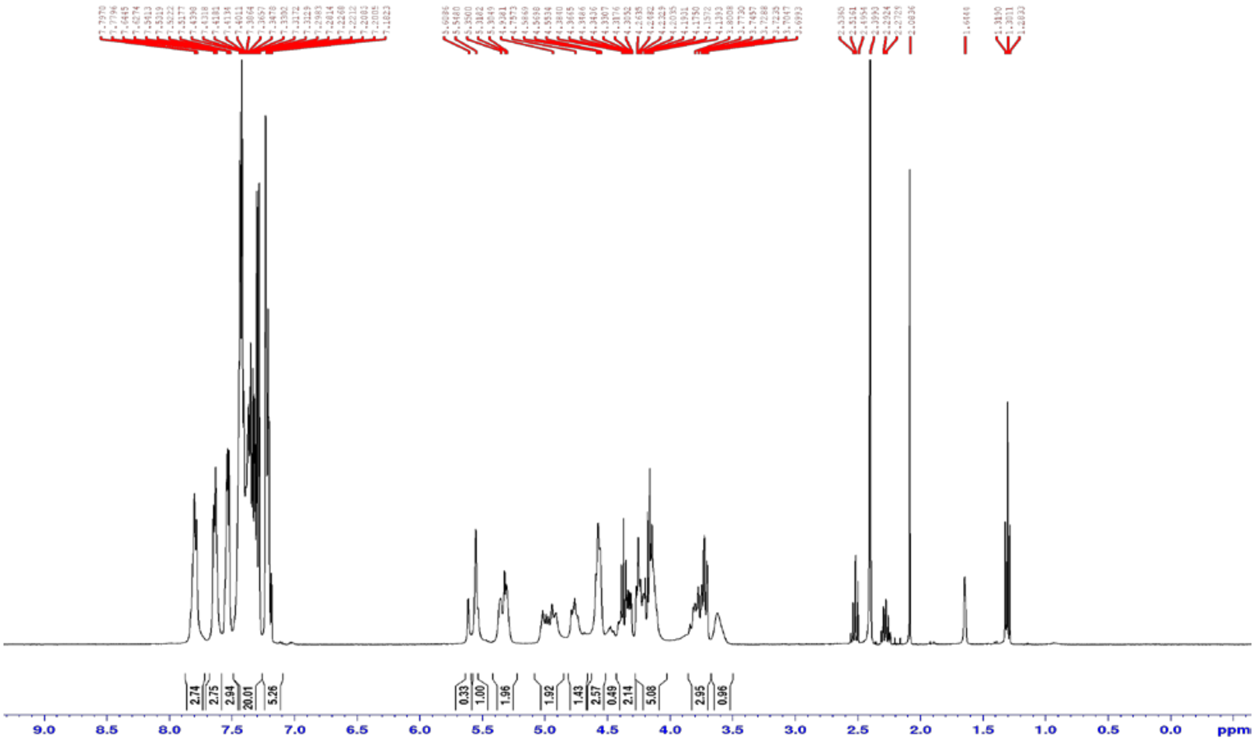

Figure. ^{1}H NMR ($CDCl_3$, 400 MHz) spectrum of **15**.

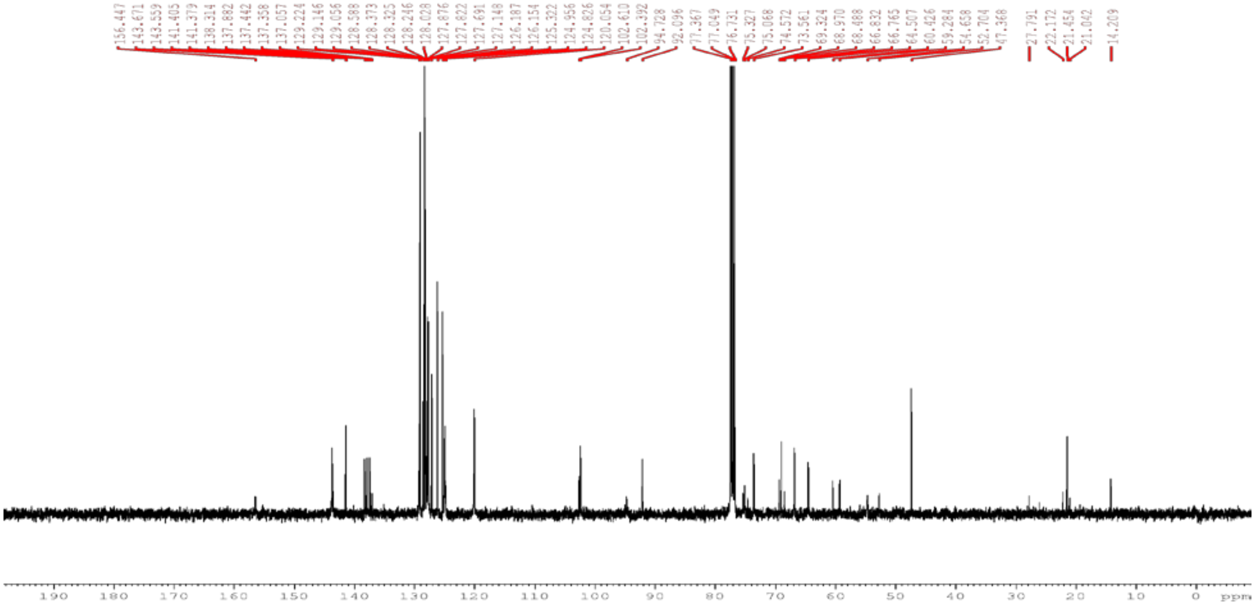

Figure. ^{13}C NMR ($CDCl_3$, 400 MHz) spectrum of **15**.

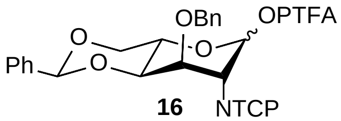

16

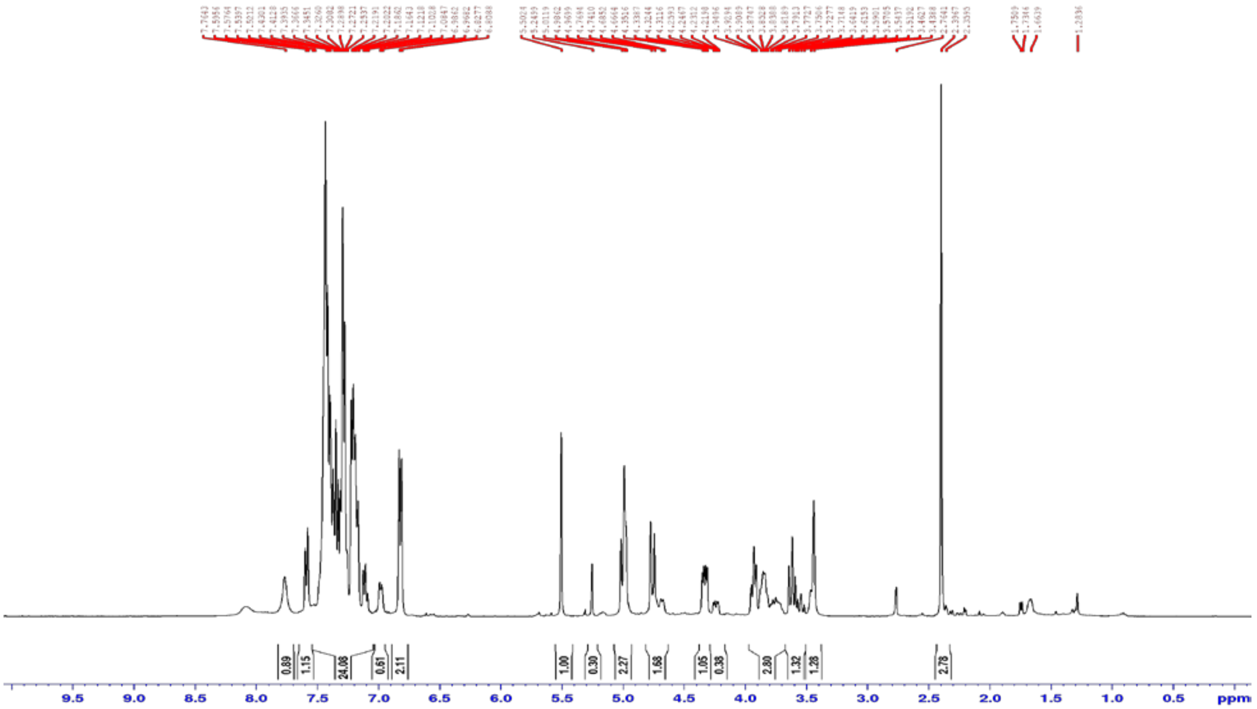

Figure. ^{1}H NMR ($CDCl_3$, 400 MHz) spectrum of **16**.

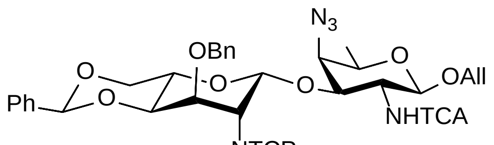

17

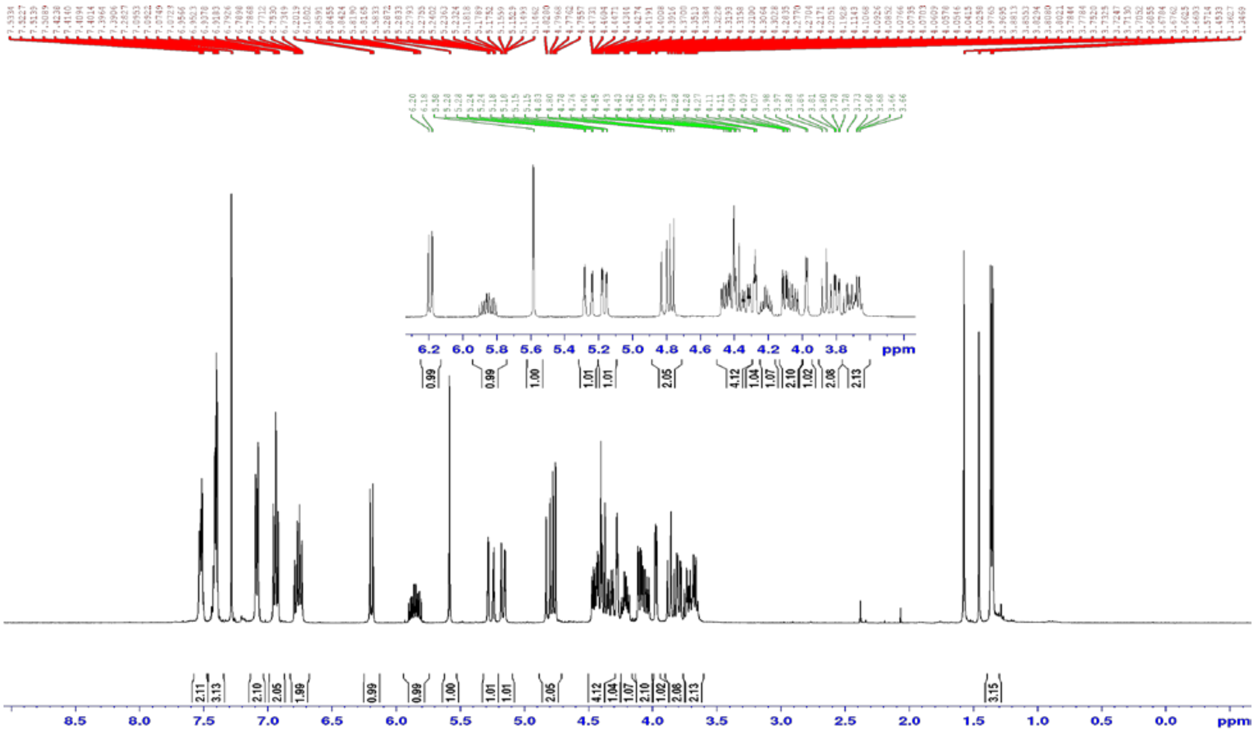

Figure. ^{1}H NMR ($CDCl_3$, 400 MHz) spectrum of **17**.

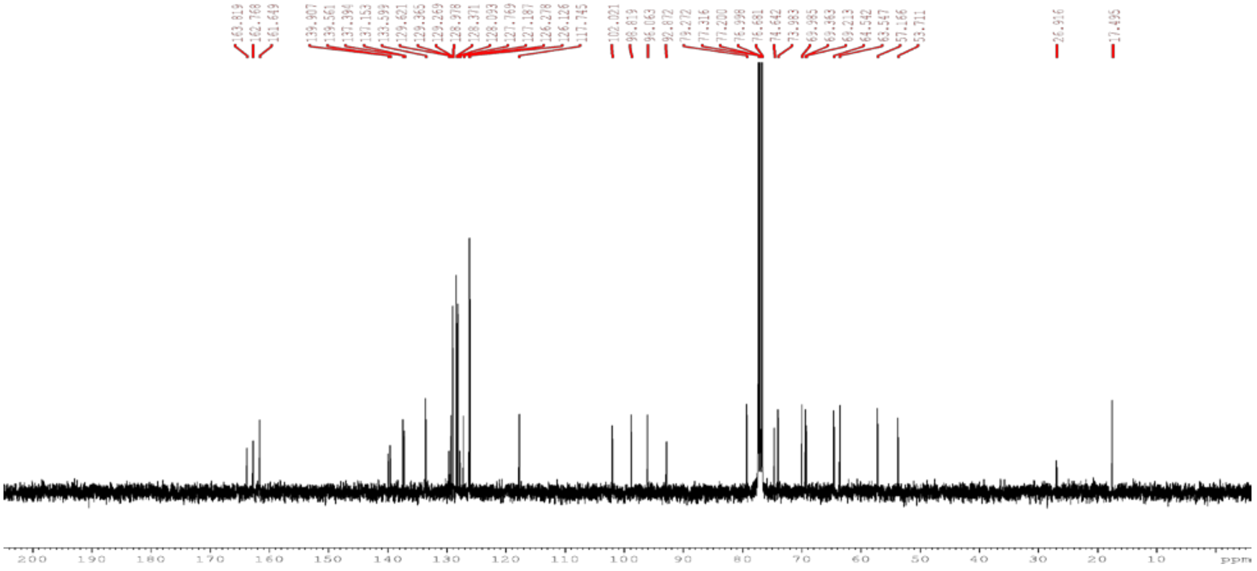

Figure. ^{13}C NMR ($CDCl_3$, 400 MHz) spectrum of **17**.

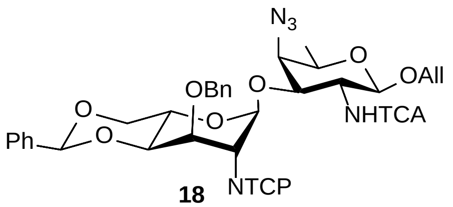

18

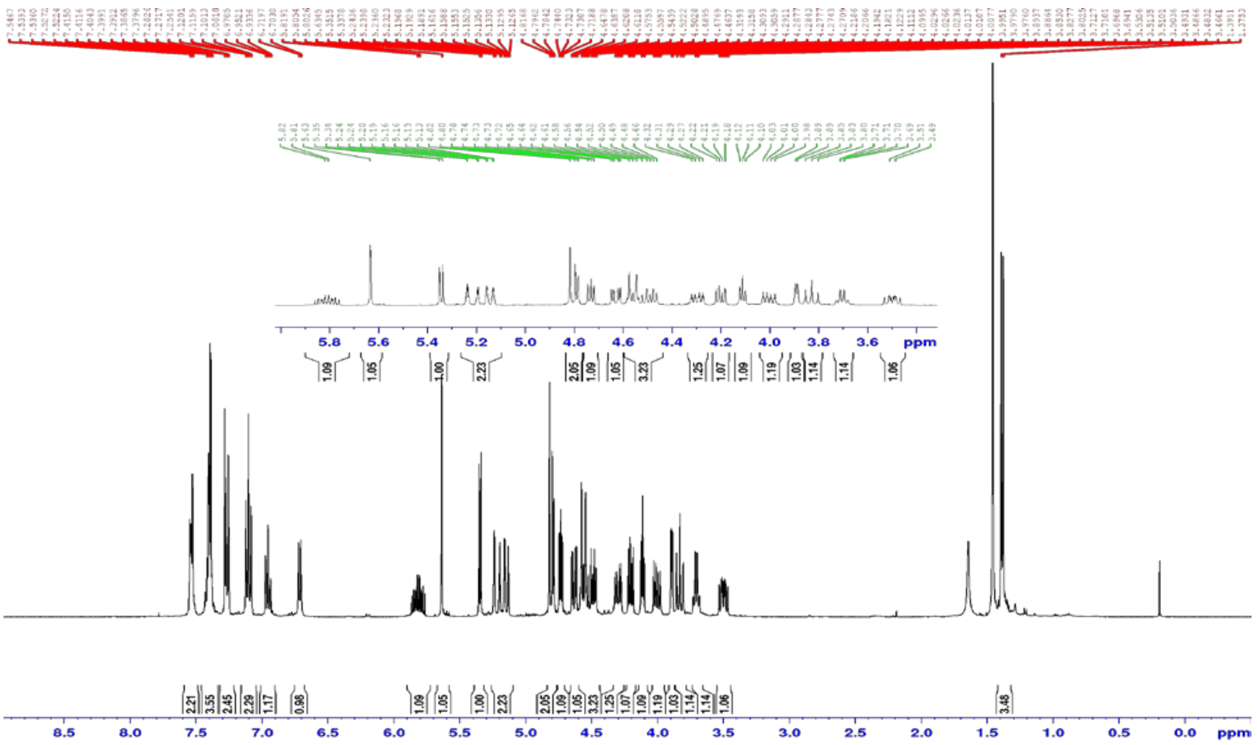

Figure. ^{1}H NMR ($CDCl_3$, 400 MHz) spectrum of **18**.

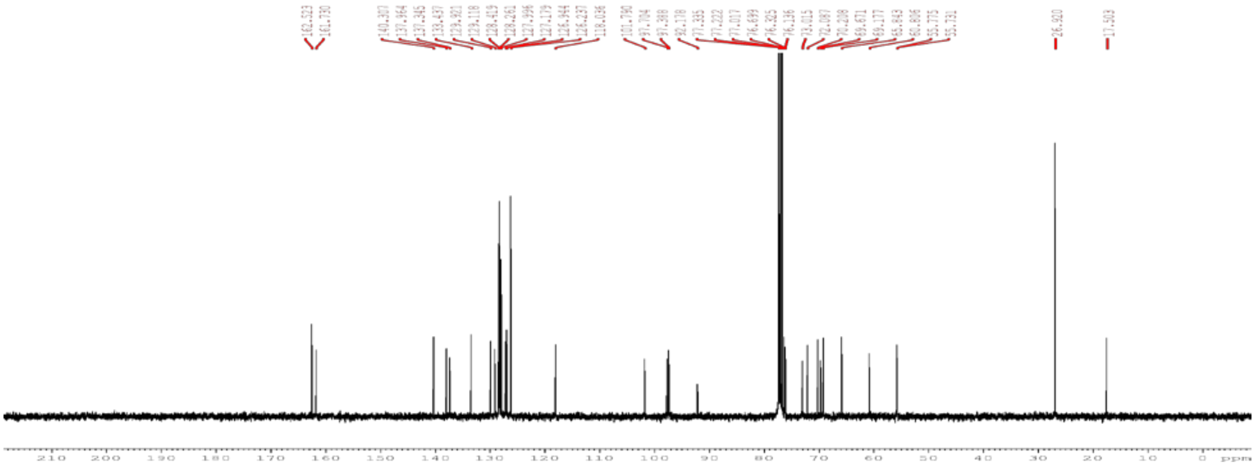

Figure. ^{13}C NMR ($CDCl_3$, 400 MHz) spectrum of **18**.

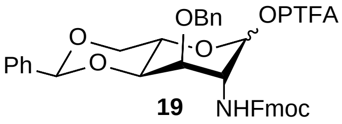

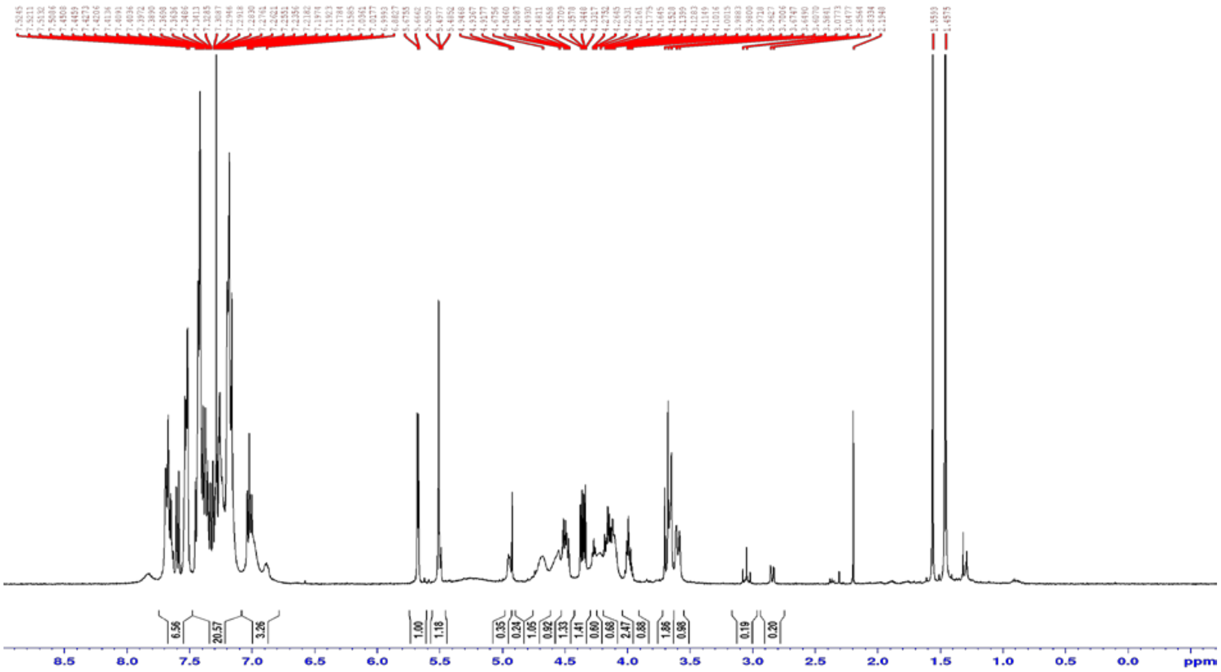

Figure. ^{1}H NMR ($CDCl_3$, 400 MHz) spectrum of **19**.

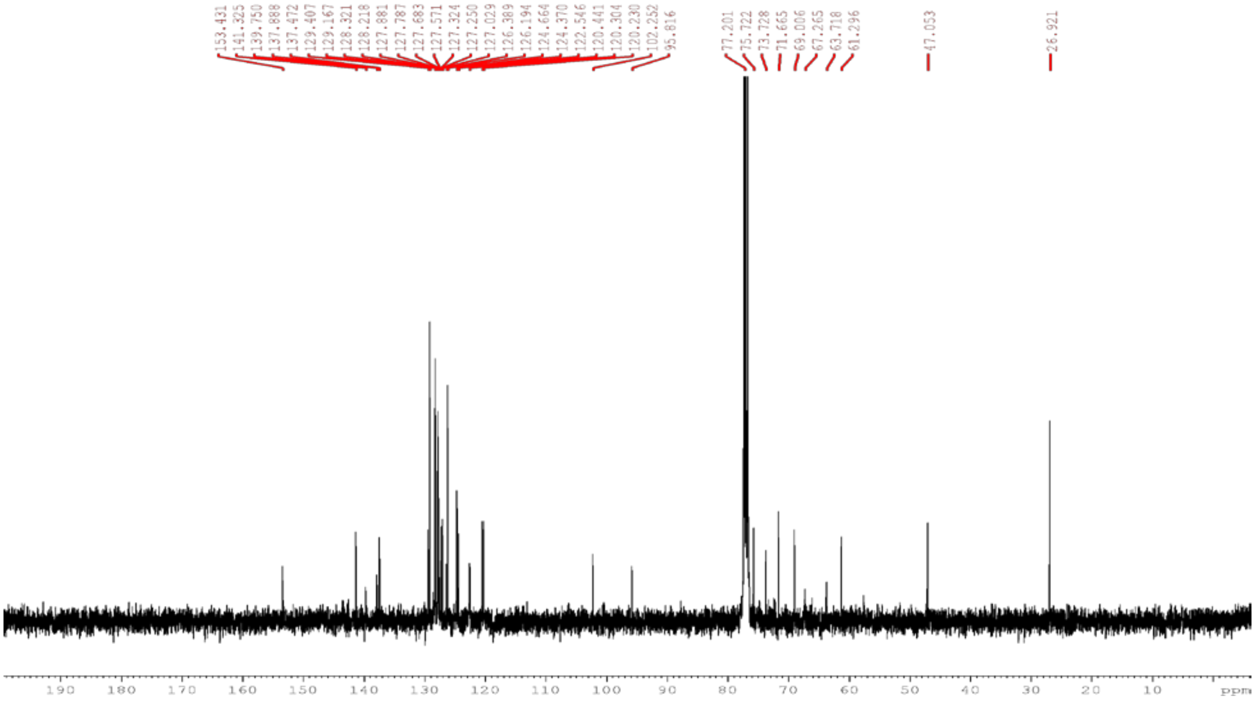

Figure. ^{13}C NMR ($CDCl_3$, 400 MHz) spectrum of **19**.

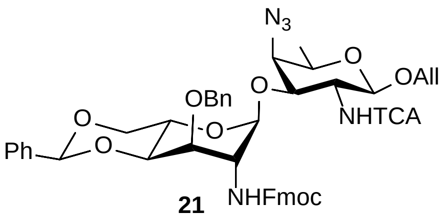

21

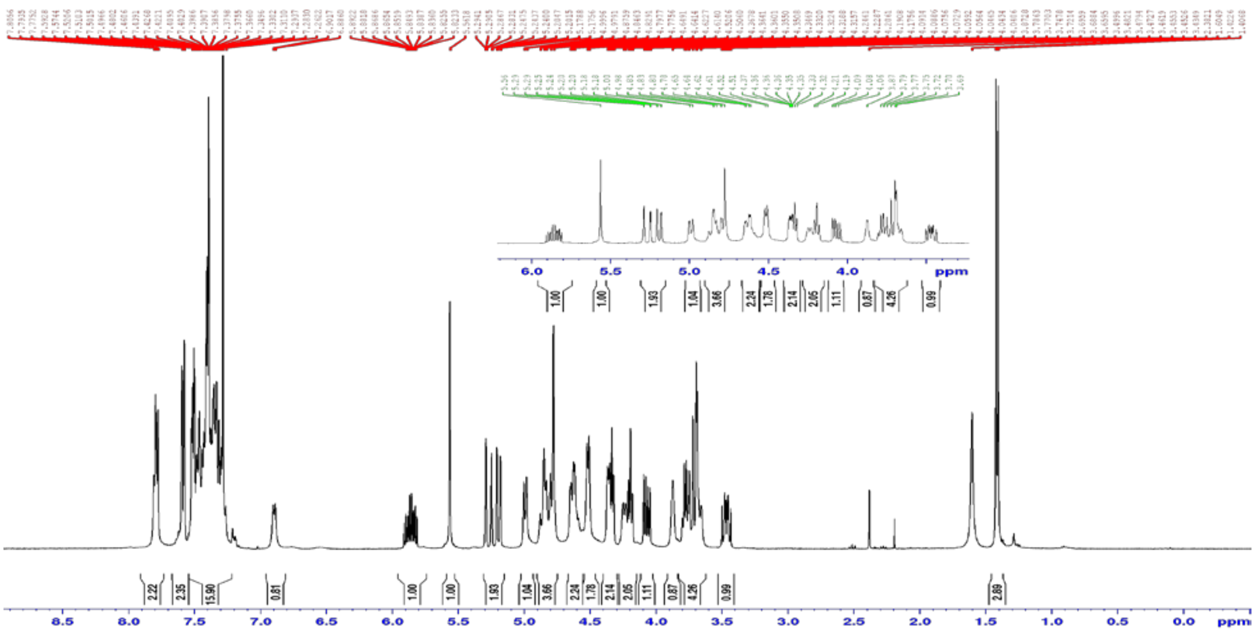

Figure. ^{1}H NMR ($CDCl_3$, 400 MHz) spectrum of **21**.

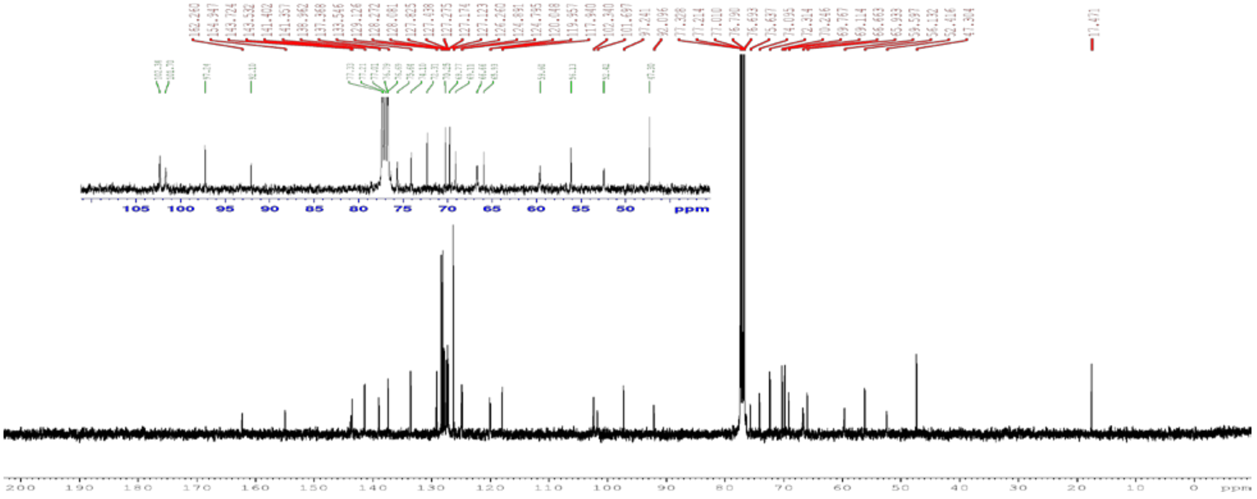

Figure. ^{13}C NMR ($CDCl_3$, 400 MHz) spectrum of **21**.

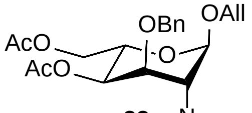

22

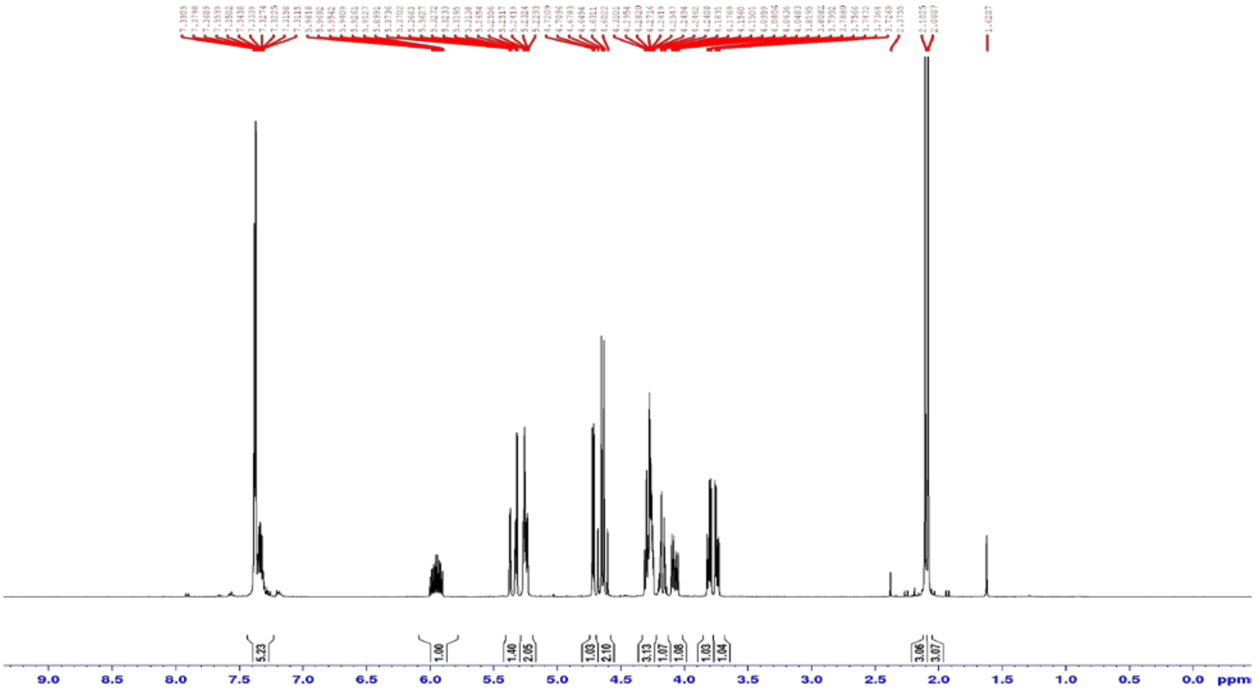

Figure. ^{1}H NMR ($CDCl_3$, 400 MHz) spectrum of **22**.

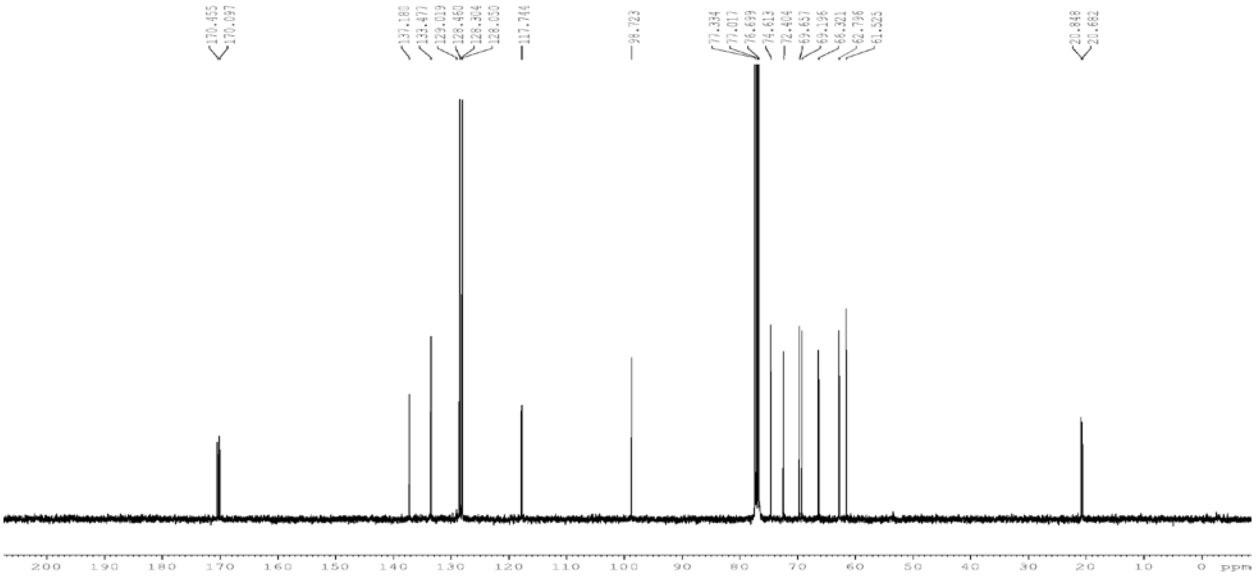

Figure. ^{13}C NMR ($CDCl_3$, 400 MHz) spectrum of **22**.

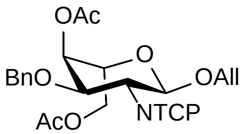

23

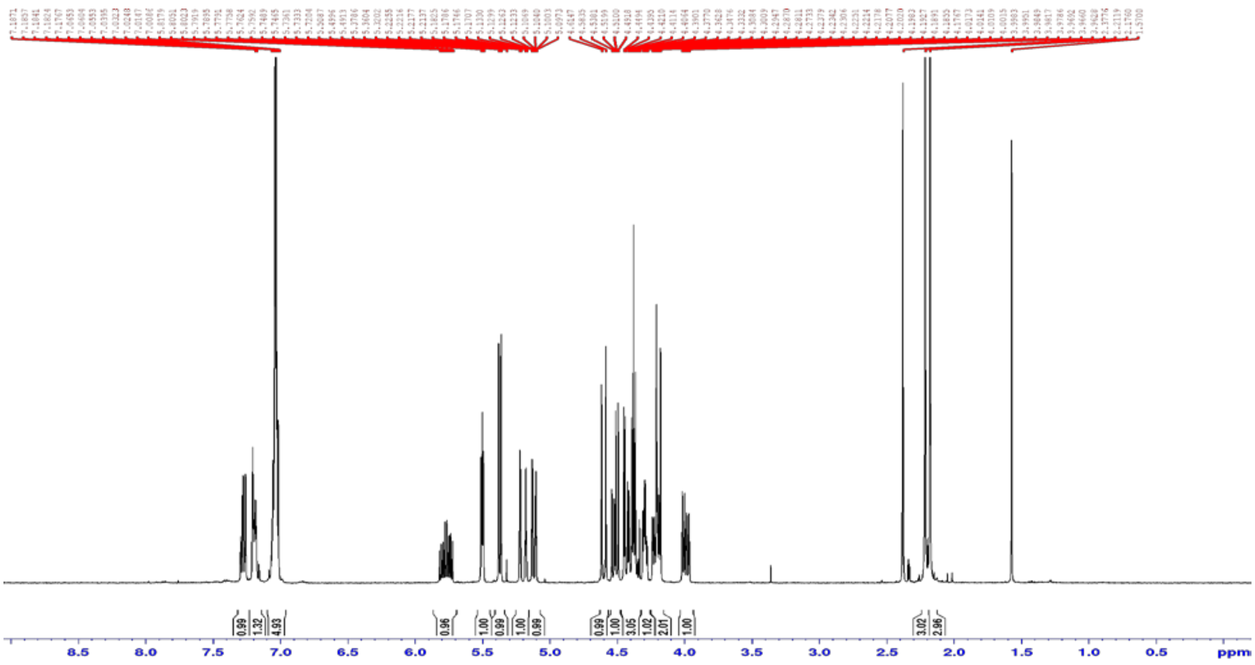

Figure. ^{1}H NMR ($CDCl_3$, 400 MHz) spectrum of **23**.

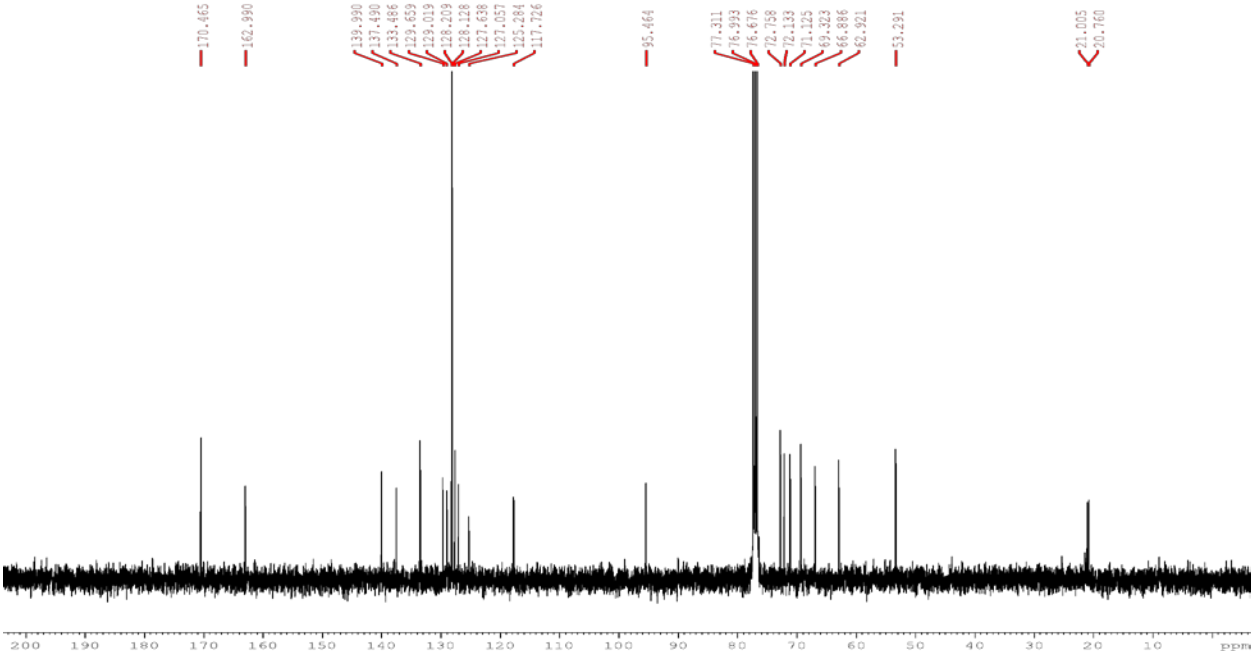

Figure. ^{13}C NMR ($CDCl_3$, 400 MHz) spectrum of **23**.

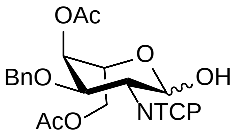

24

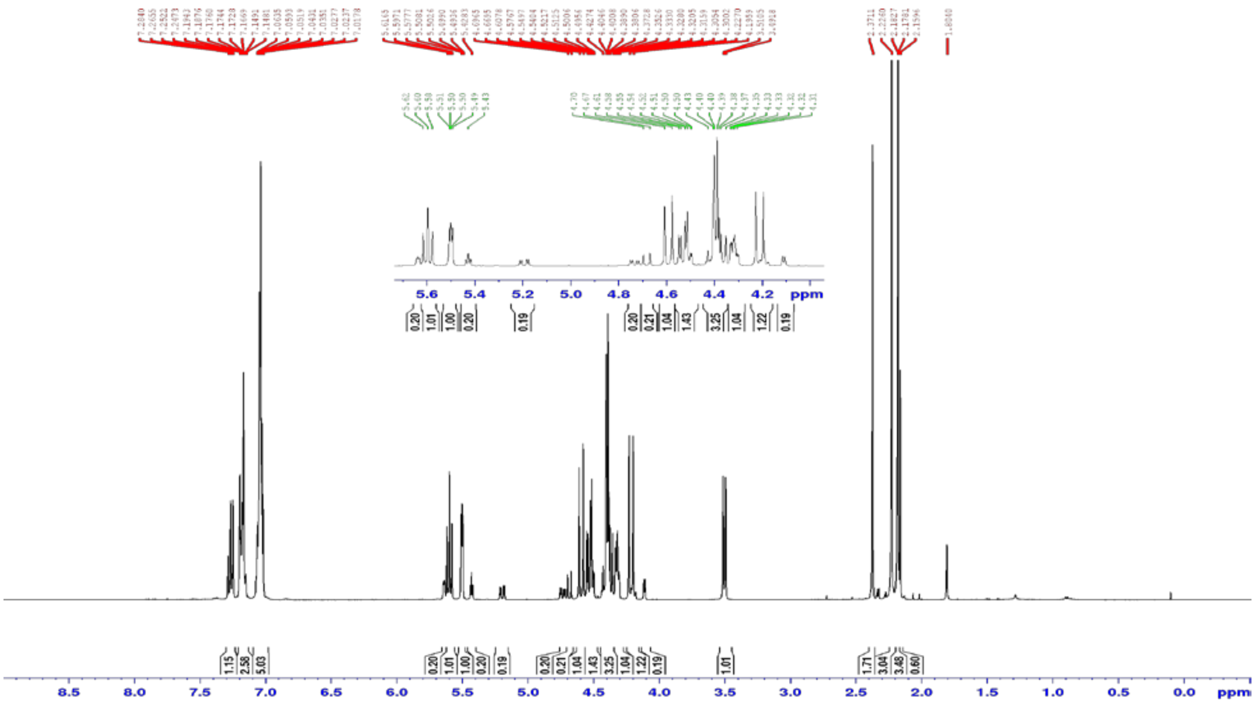

Figure. ^{1}H NMR ($CDCl_3$, 400 MHz) spectrum of **24**.

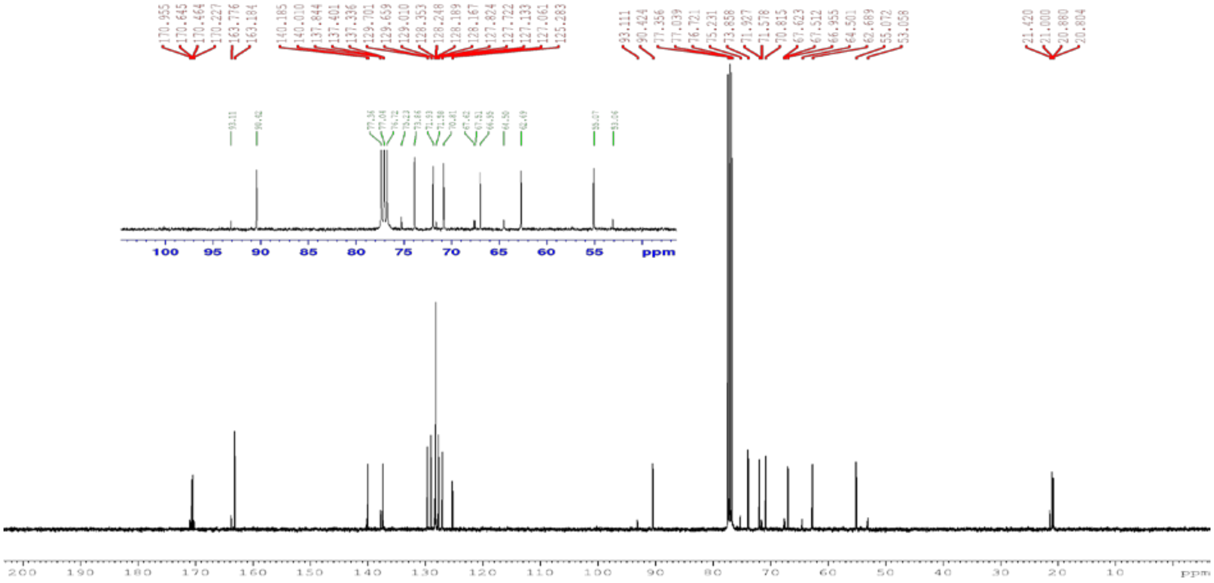

Figure. ^{13}C NMR ($CDCl_3$, 400 MHz) spectrum of **24**.

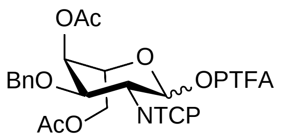

25

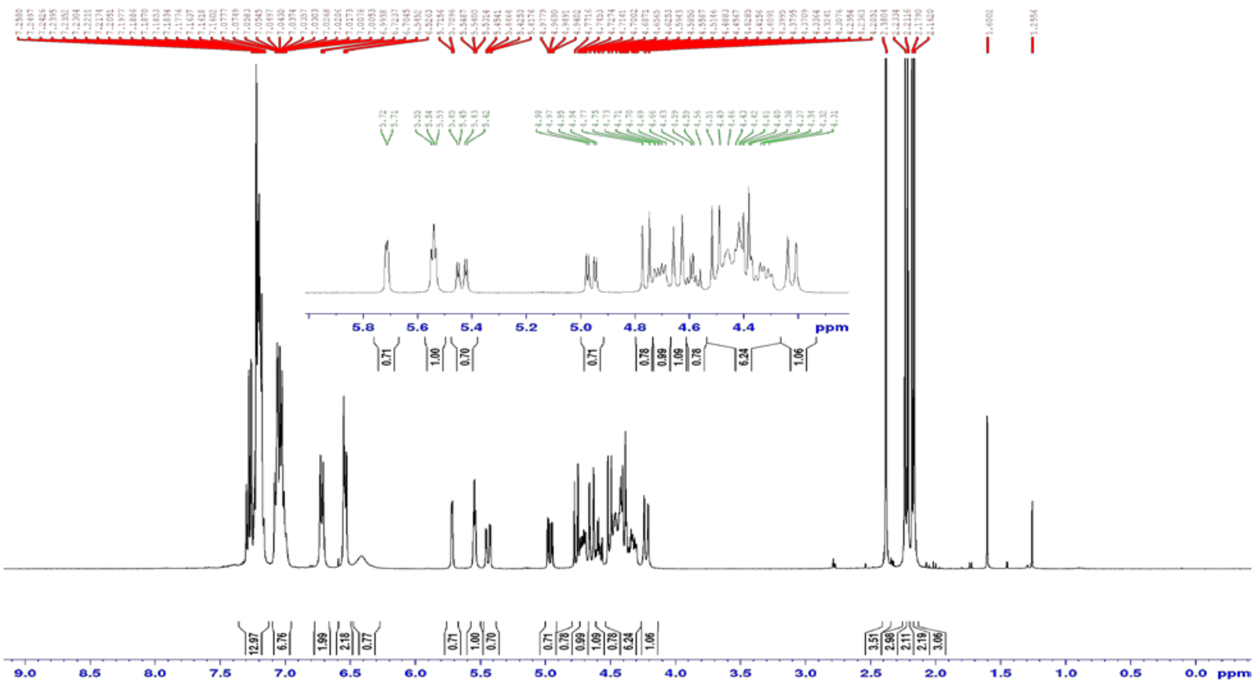

Figure. ^{1}H NMR ($CDCl_3$, 400 MHz) spectrum of **25**.

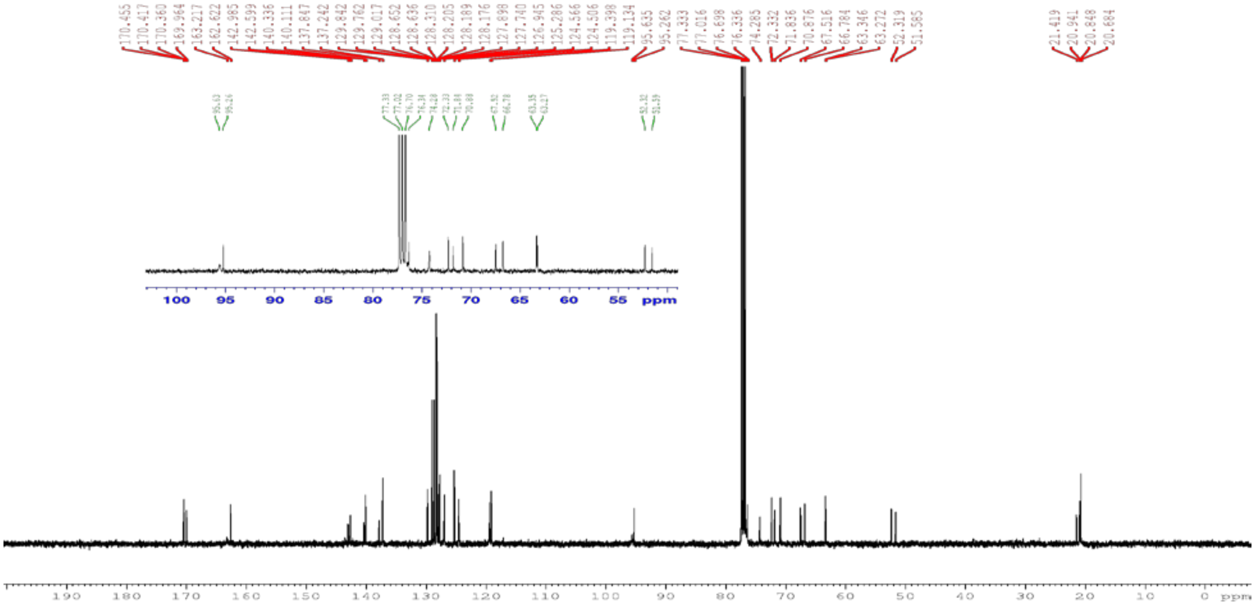

Figure. ^{13}C NMR ($CDCl_3$, 400 MHz) spectrum of **25**.

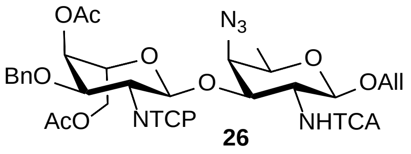

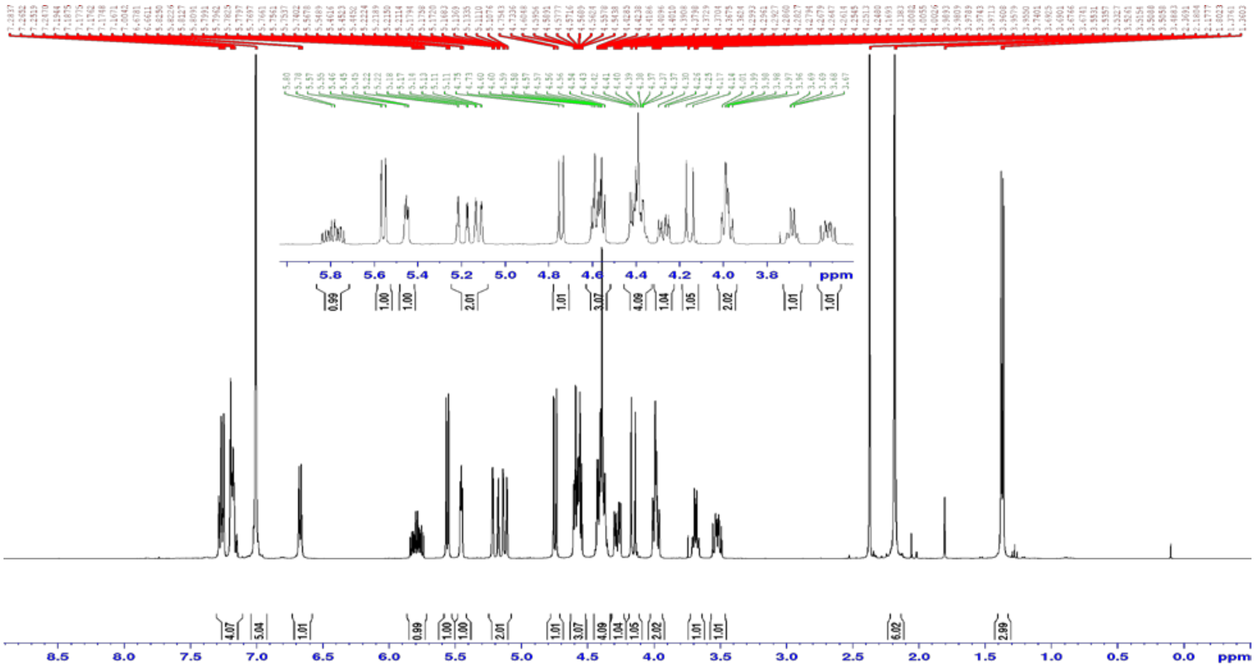

Figure. ^{1}H NMR ($CDCl_3$, 400 MHz) spectrum of **26**.

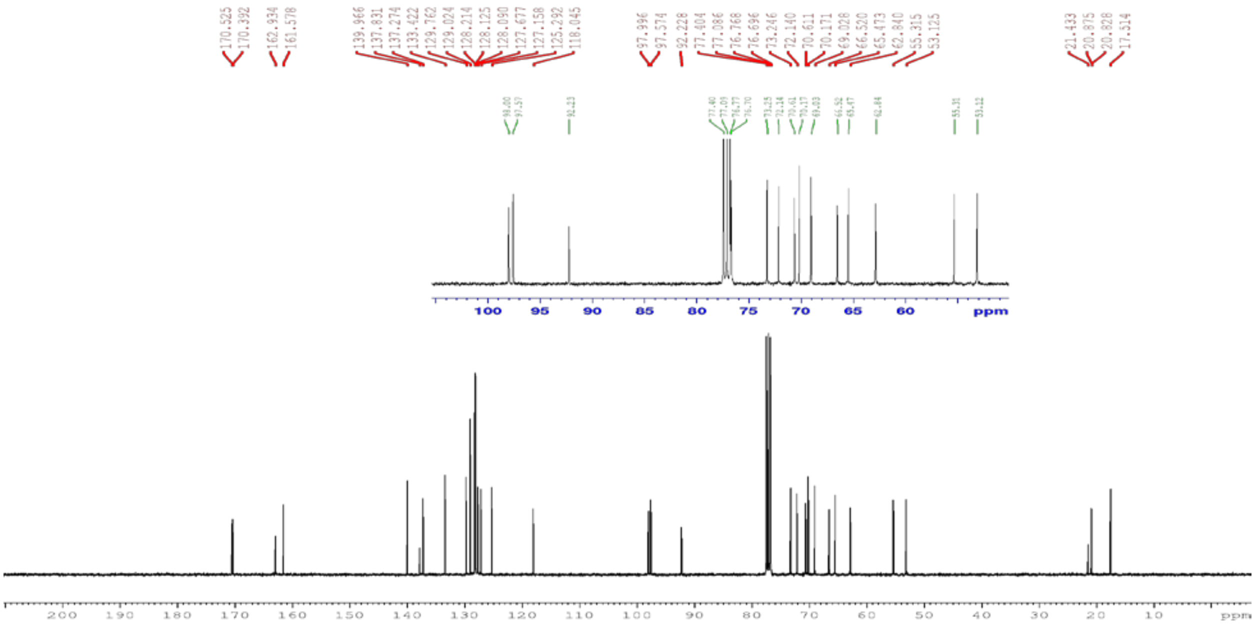

Figure. ^{13}C NMR ($CDCl_3$, 400 MHz) spectrum of **26**

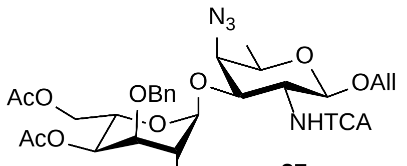

27

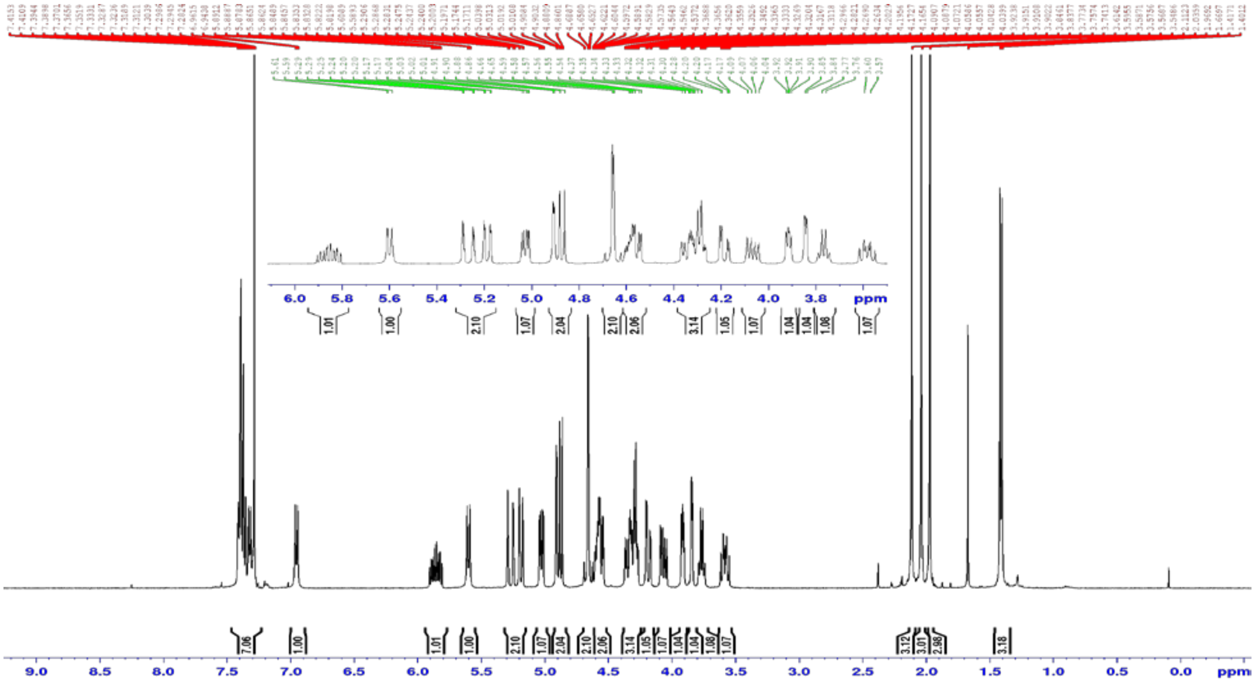

Figure. ^{1}H NMR ($CDCl_3$, 400 MHz) spectrum of **27**.

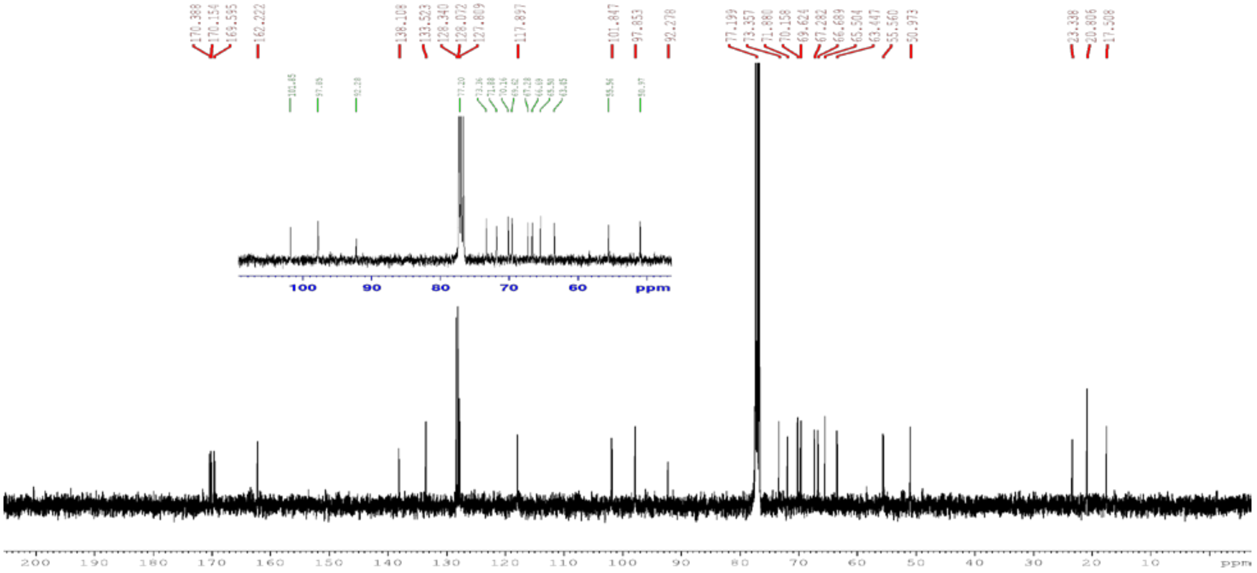

Figure. ^{13}C NMR ($CDCl_3$, 400 MHz) spectrum of **27**.

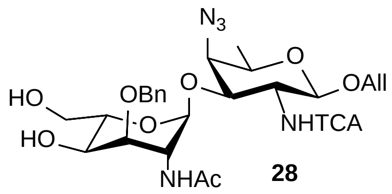

28

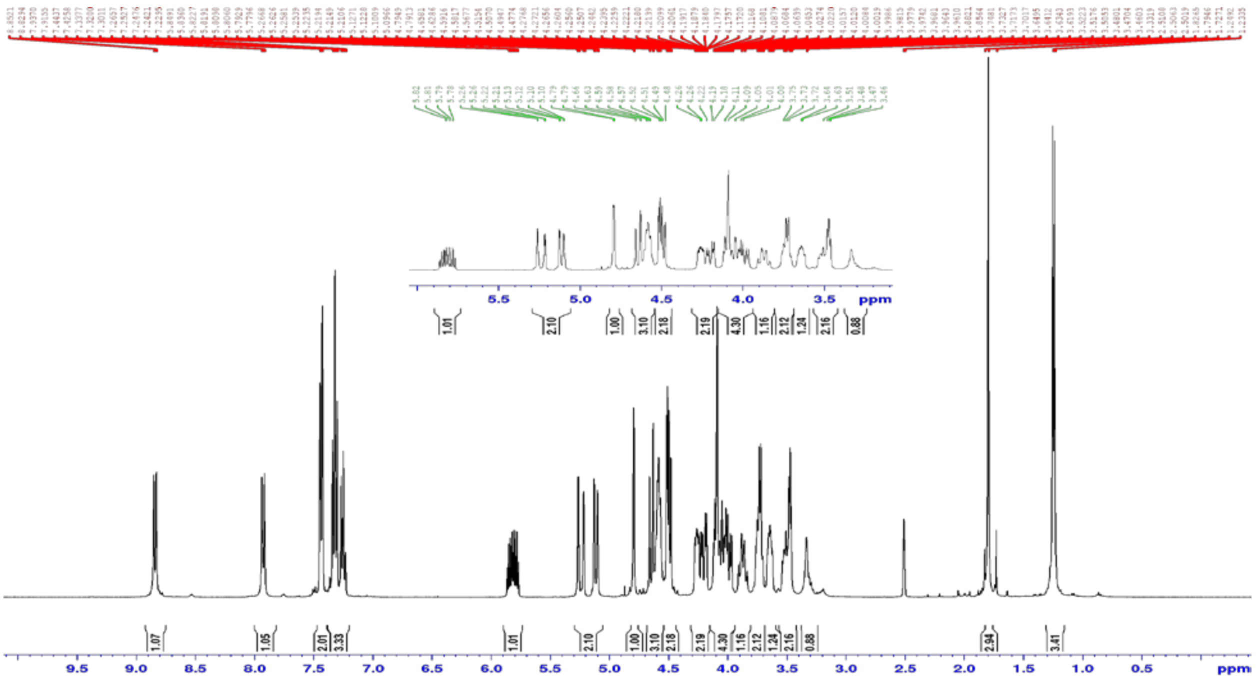

Figure. ^{1}H NMR (DMSO-d_6, 400 MHz) spectrum of **28**.

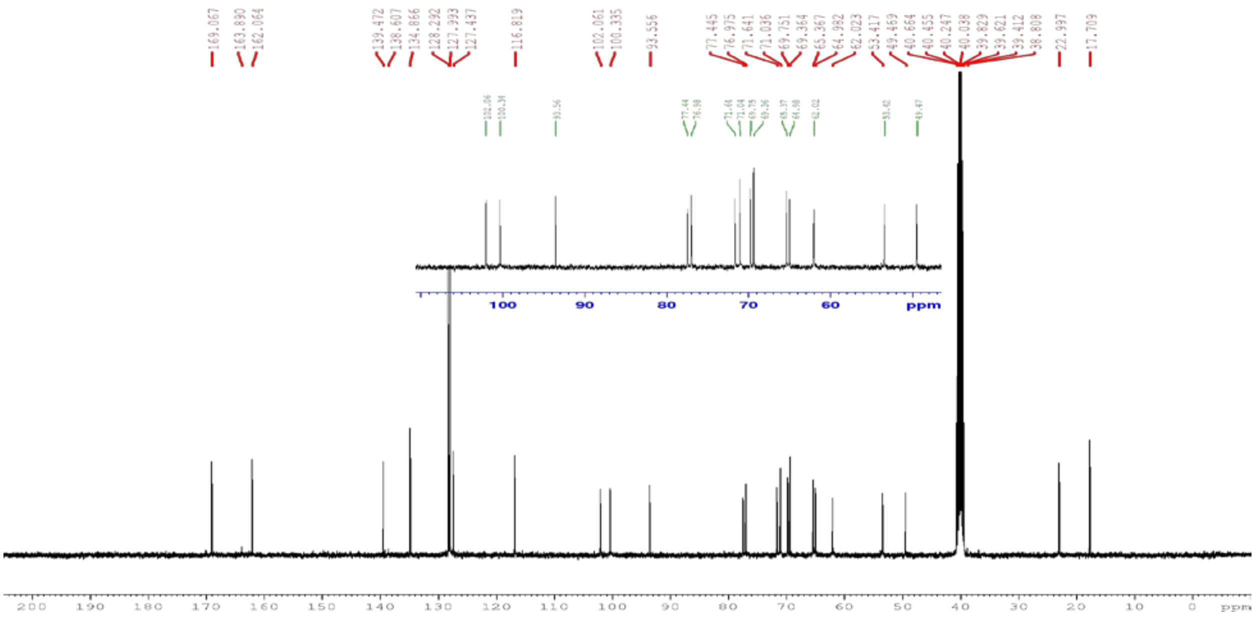

Figure. ^{13}C NMR (DMSO-d_6, 400 MHz) spectrum of **28**.

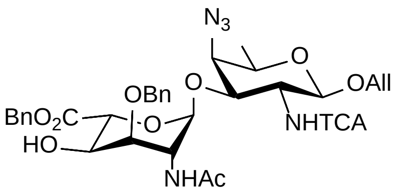

29

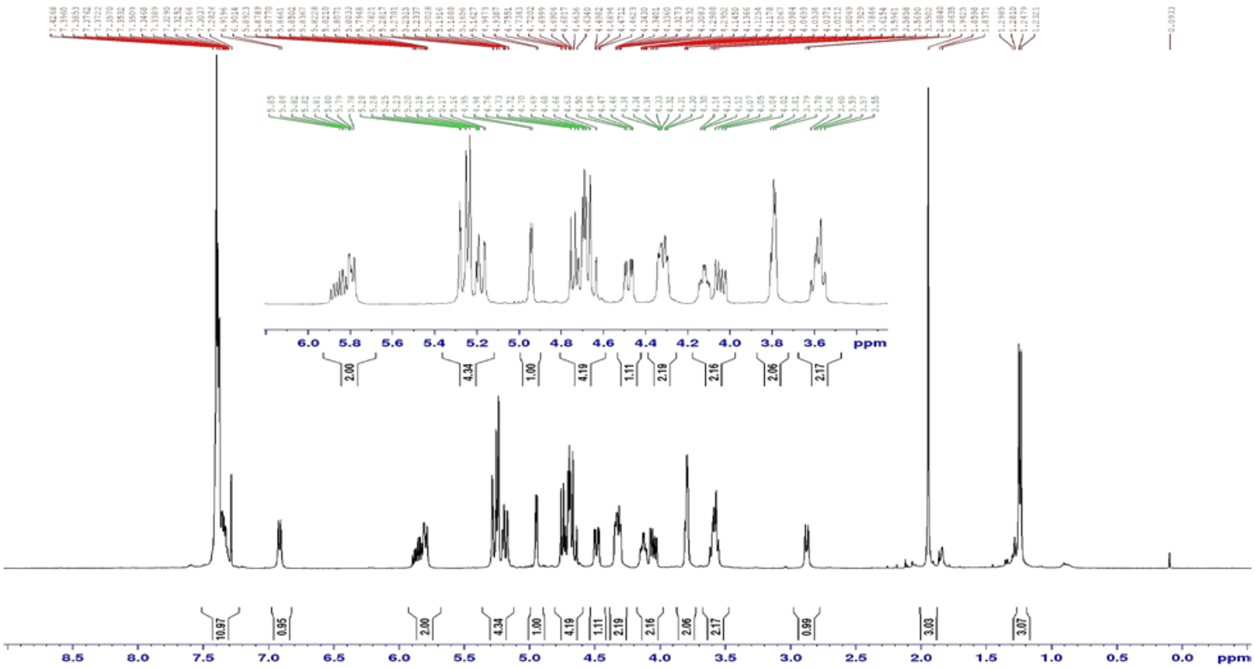

Figure. ^{1}H NMR ($CDCl_3$, 400 MHz) spectrum of **29**.

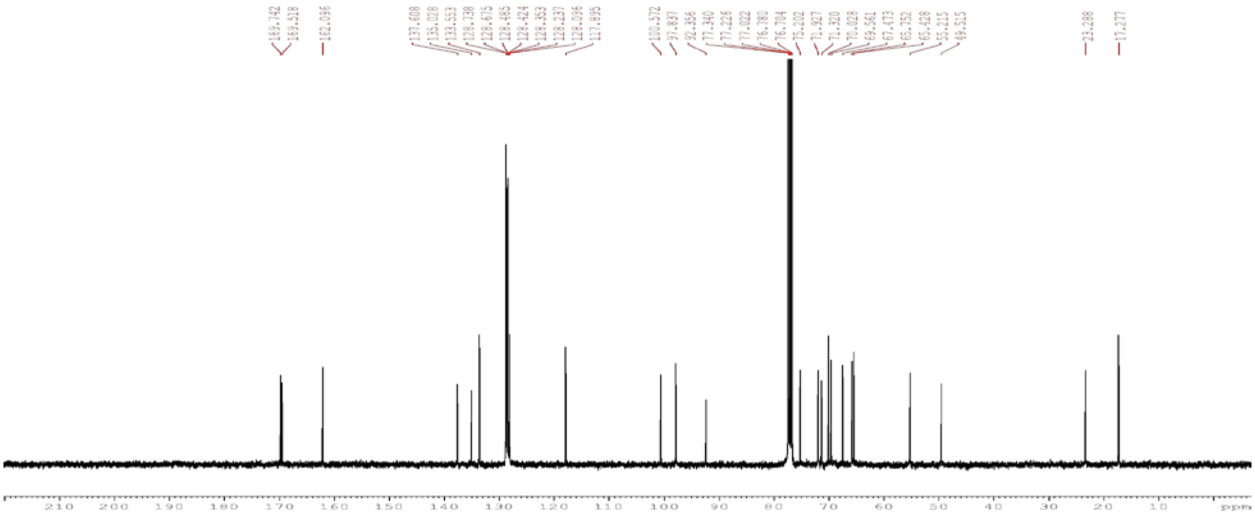

Figure. ^{13}C NMR ($CDCl_3$, 400 MHz) spectrum of **29**.

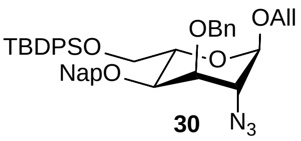

30

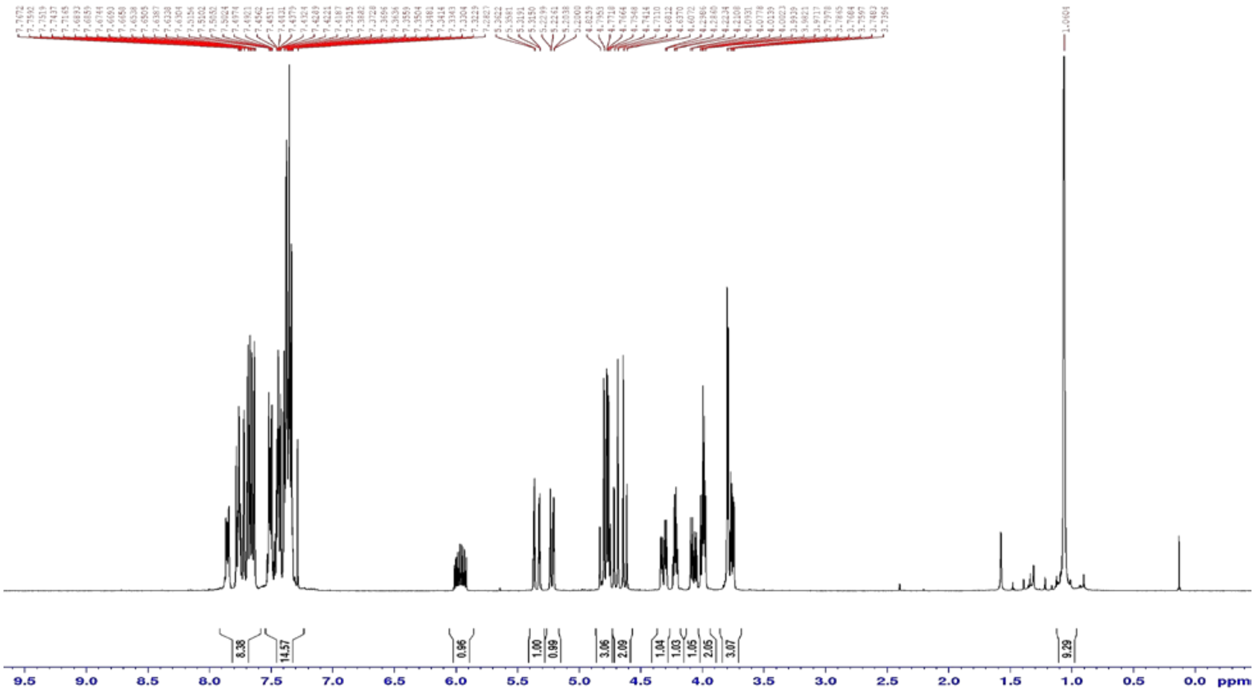

Figure. ^{1}H NMR ($CDCl_3$, 400 MHz) spectrum of **30**.

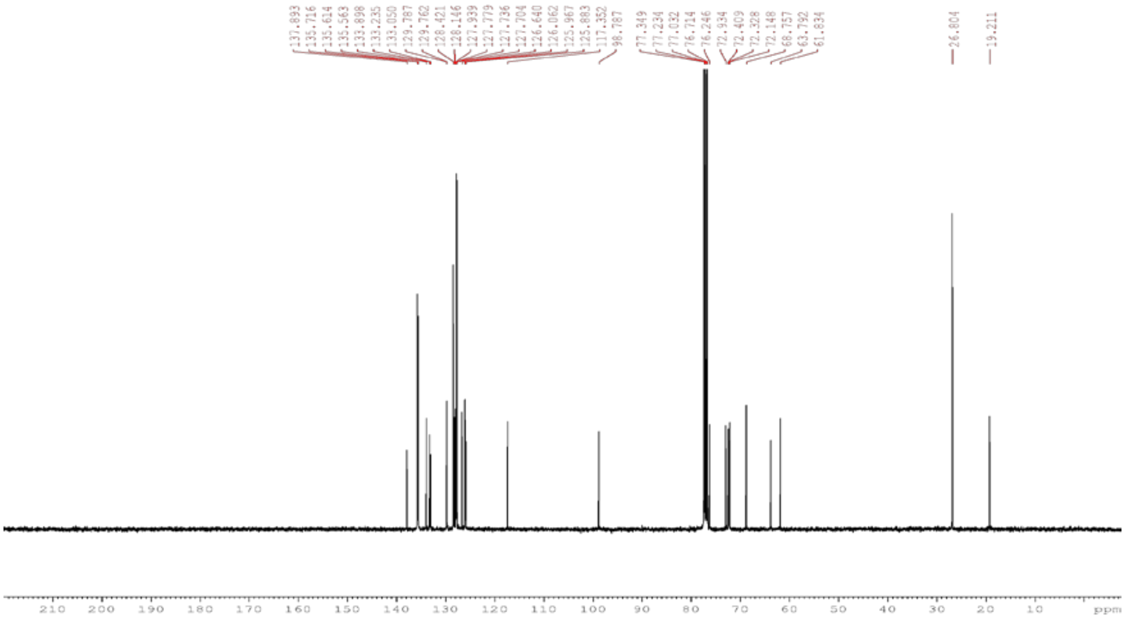

Figure. ^{13}C NMR ($CDCl_3$, 400 MHz) spectrum of **30**.

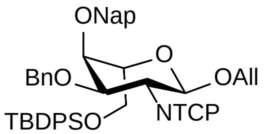

31

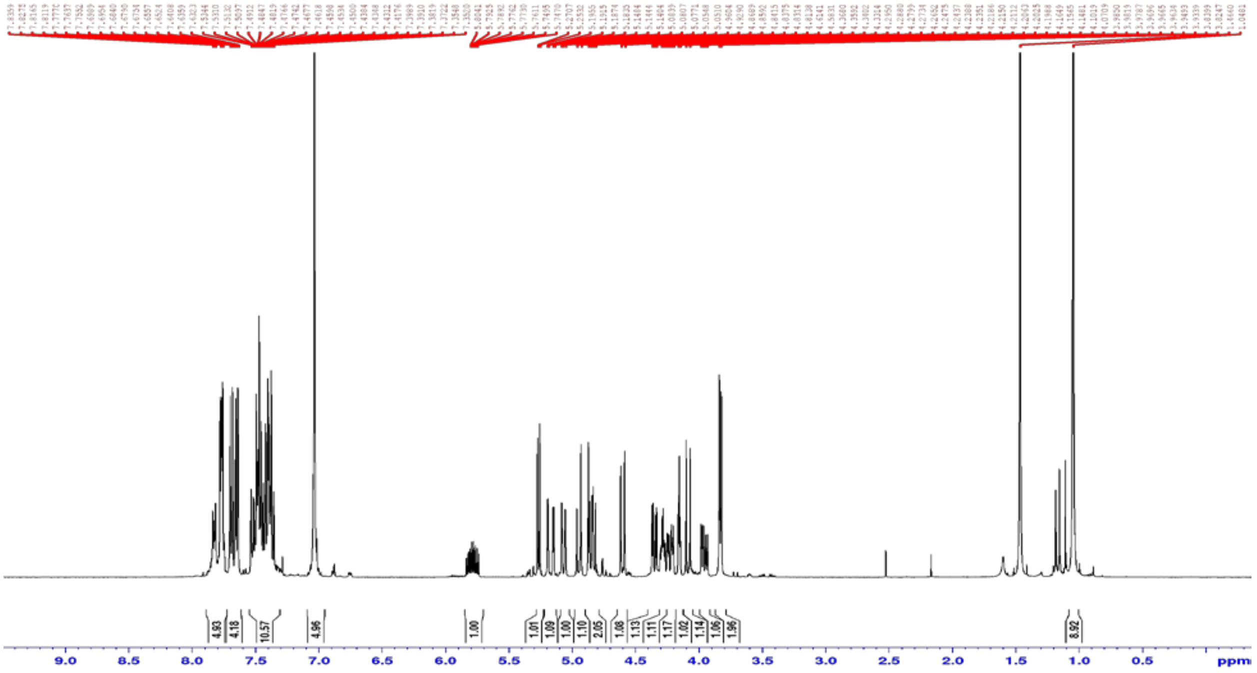

Figure. ^{1}H NMR ($CDCl_3$, 400 MHz) spectrum of **31**.

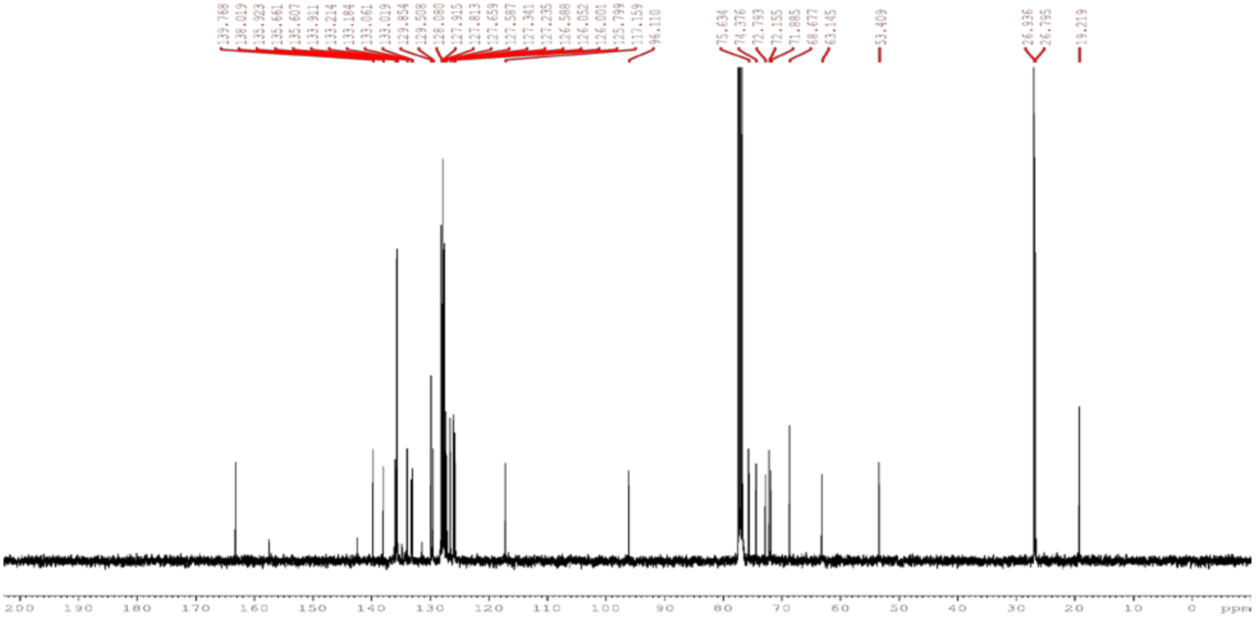

Figure. ^{13}C NMR ($CDCl_3$, 400 MHz) spectrum of **31**.

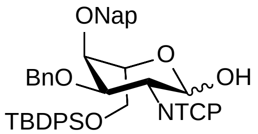

32

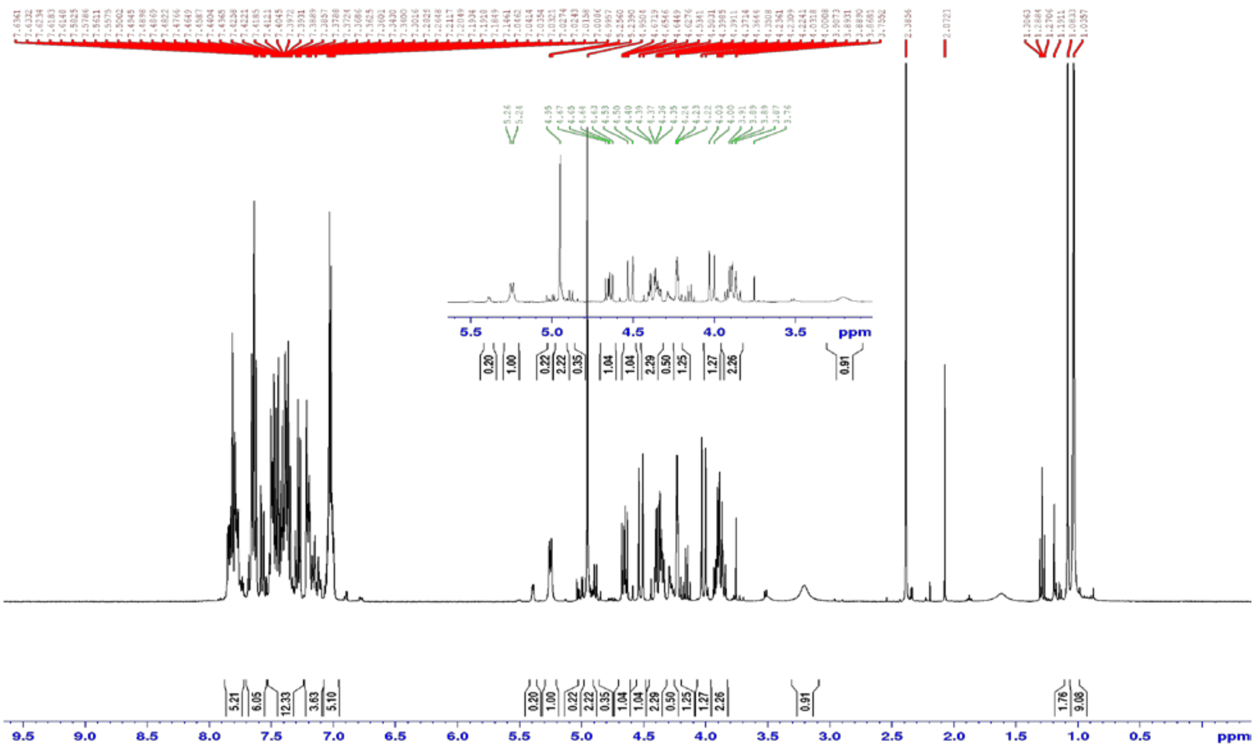

Figure. ^{1}H NMR ($CDCl_3$, 400 MHz) spectrum of **32**.

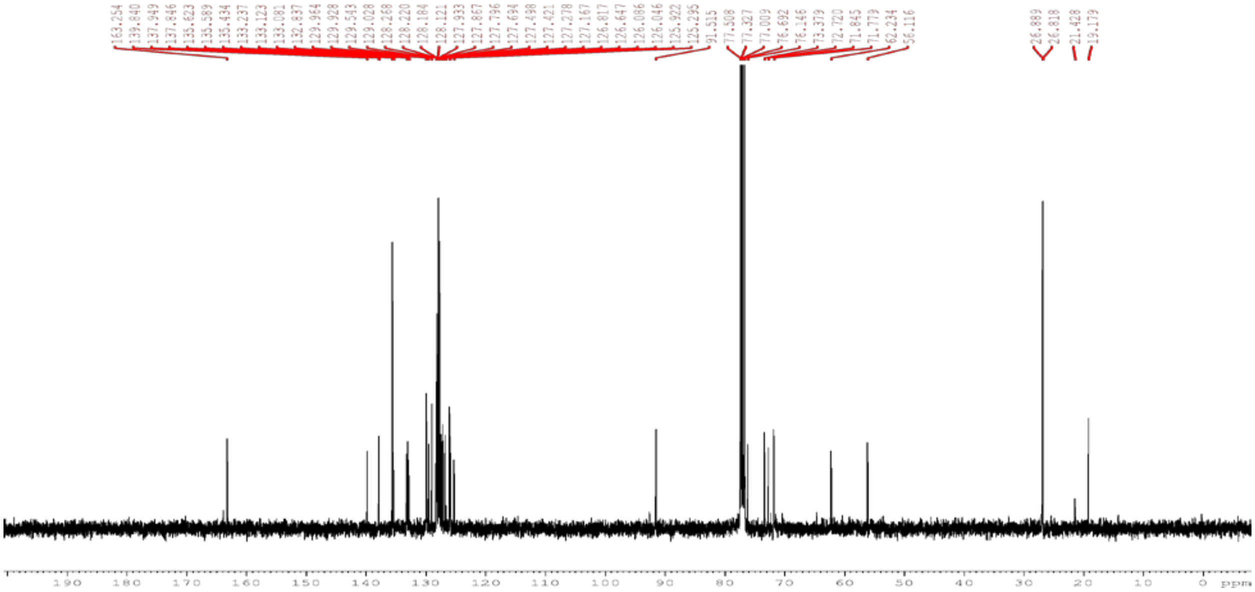

Figure. ^{13}C NMR ($CDCl_3$, 400 MHz) spectrum of **32**.

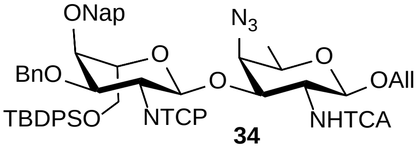

34

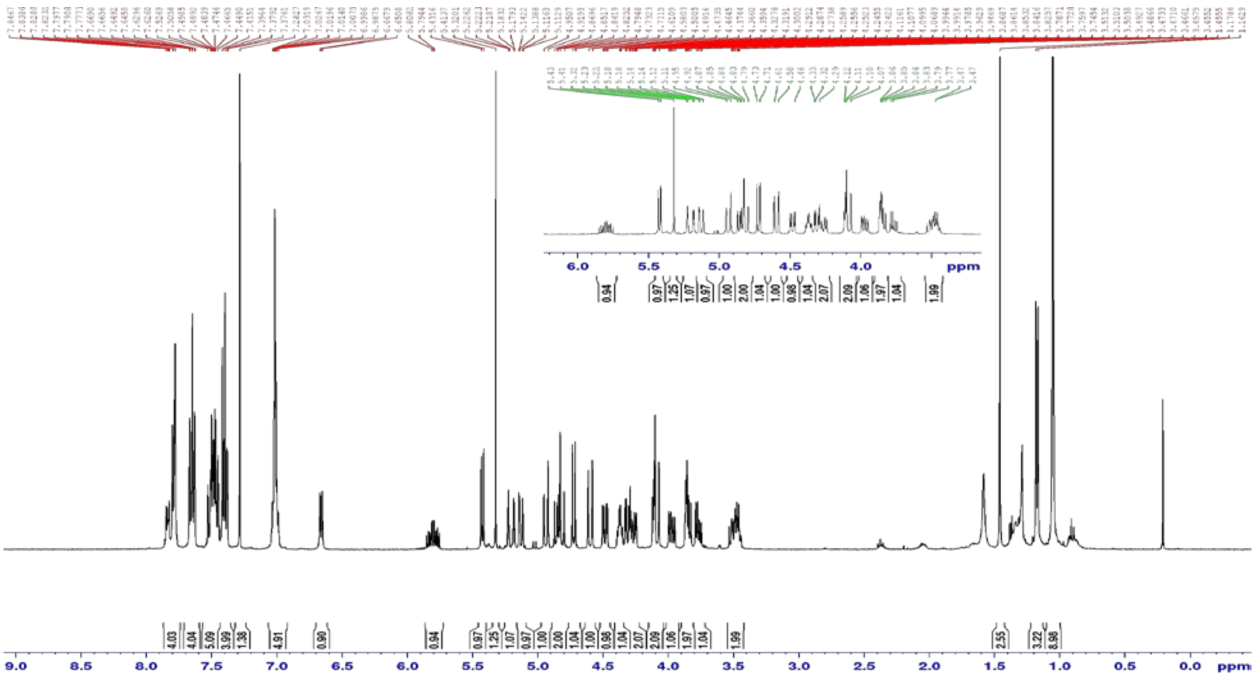

Figure. ^{1}H NMR ($CDCl_3$, 400 MHz) spectrum of **34**.

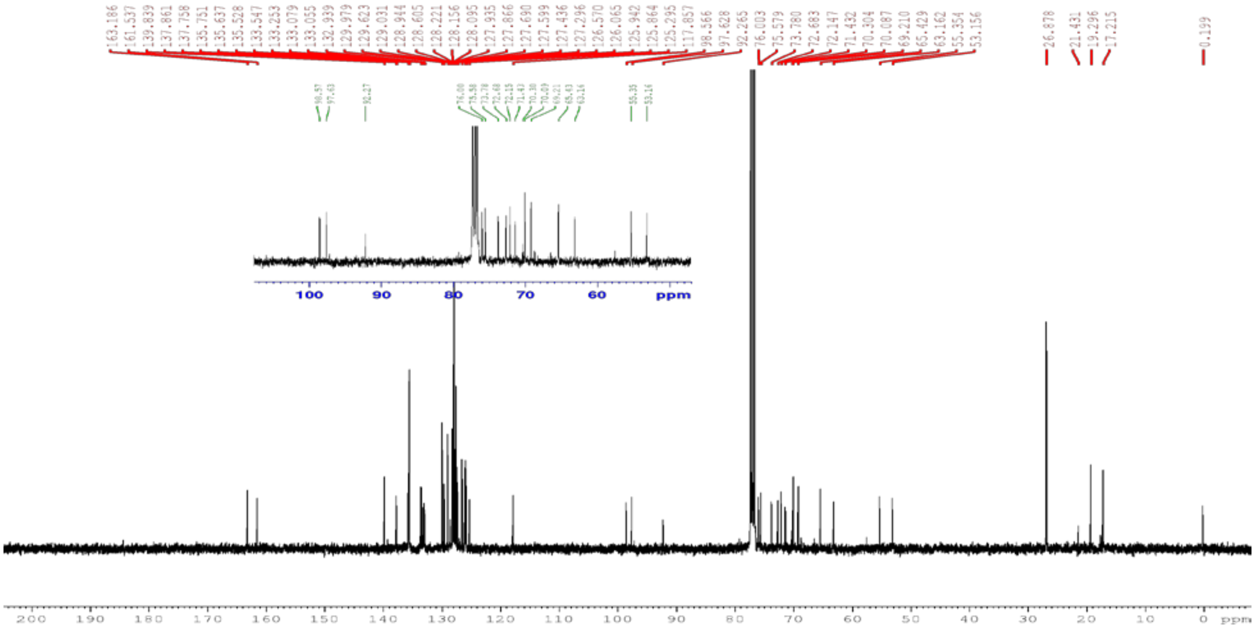

Figure. ^{13}C NMR ($CDCl_3$, 400 MHz) spectrum of **34**.

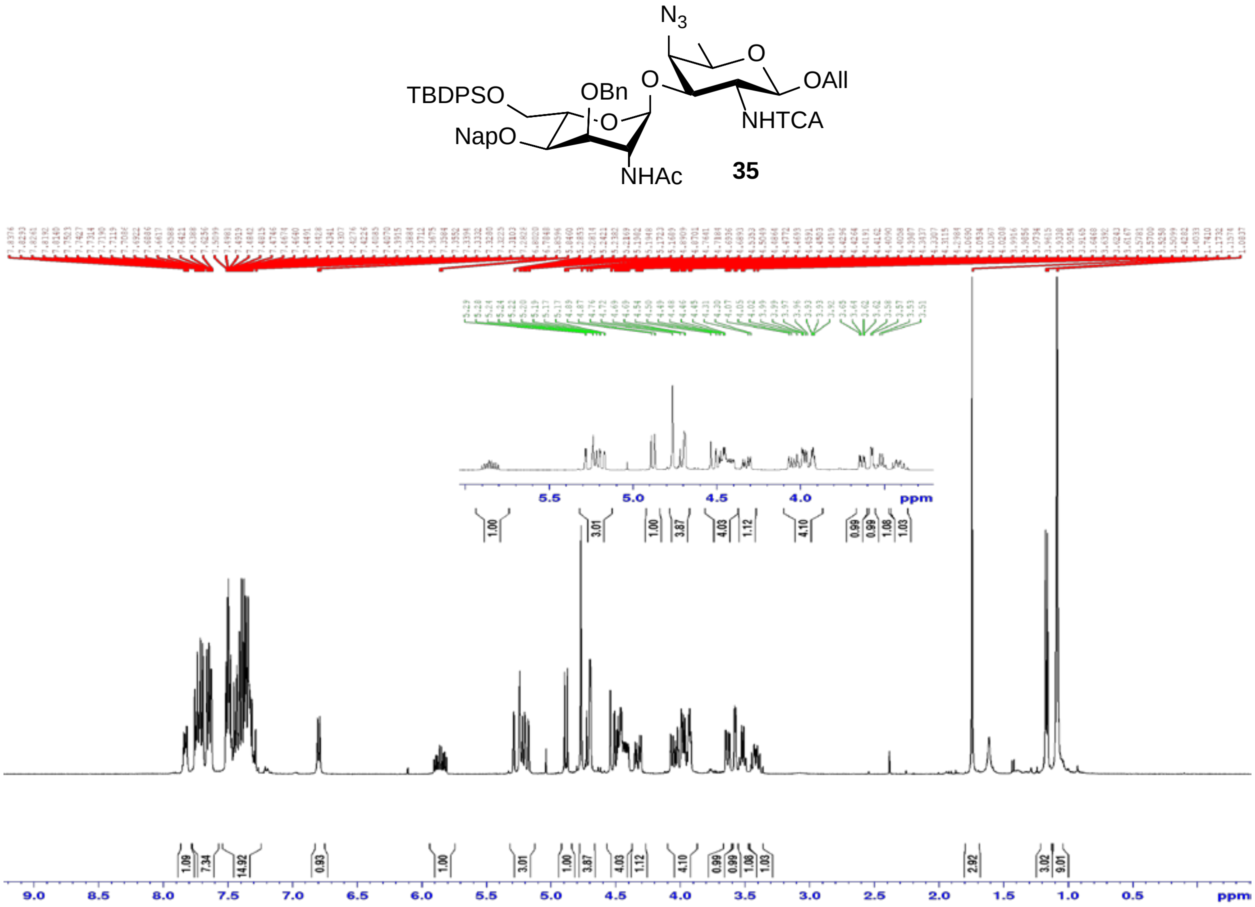

Figure. ^{1}H NMR ($CDCl_3$, 400 MHz) spectrum of **35**.

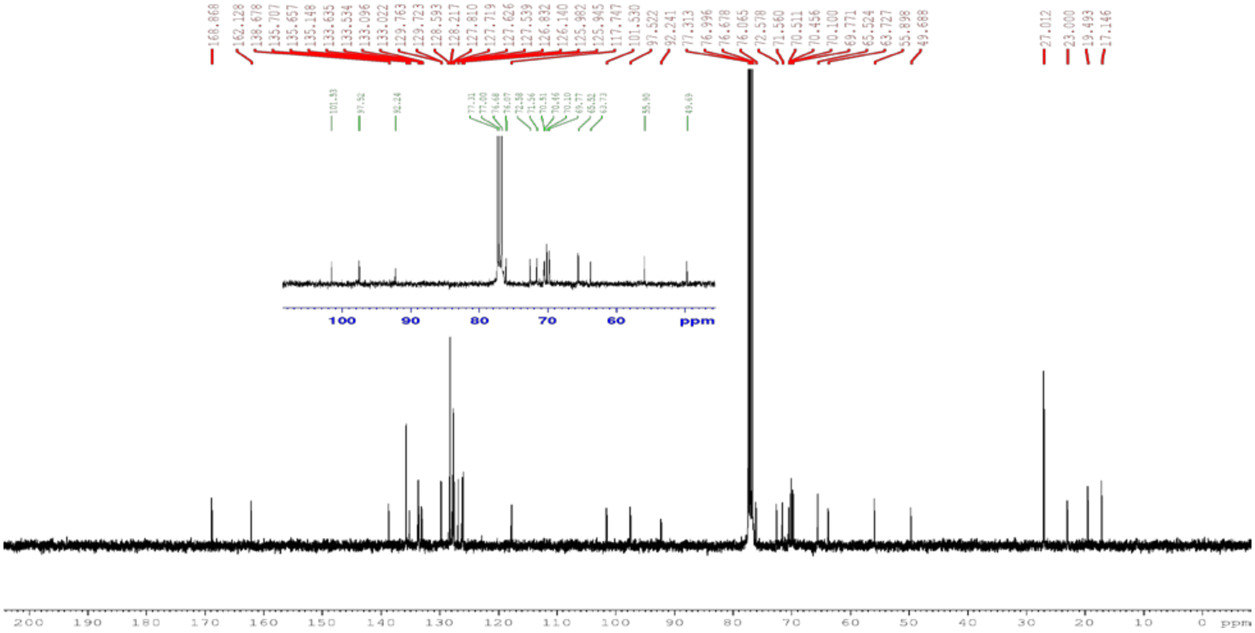

Figure. ^{13}C NMR ($CDCl_3$, 400 MHz) spectrum of **35**.

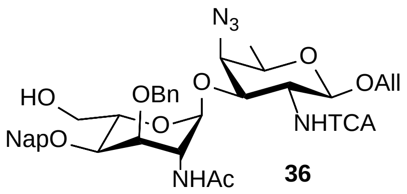

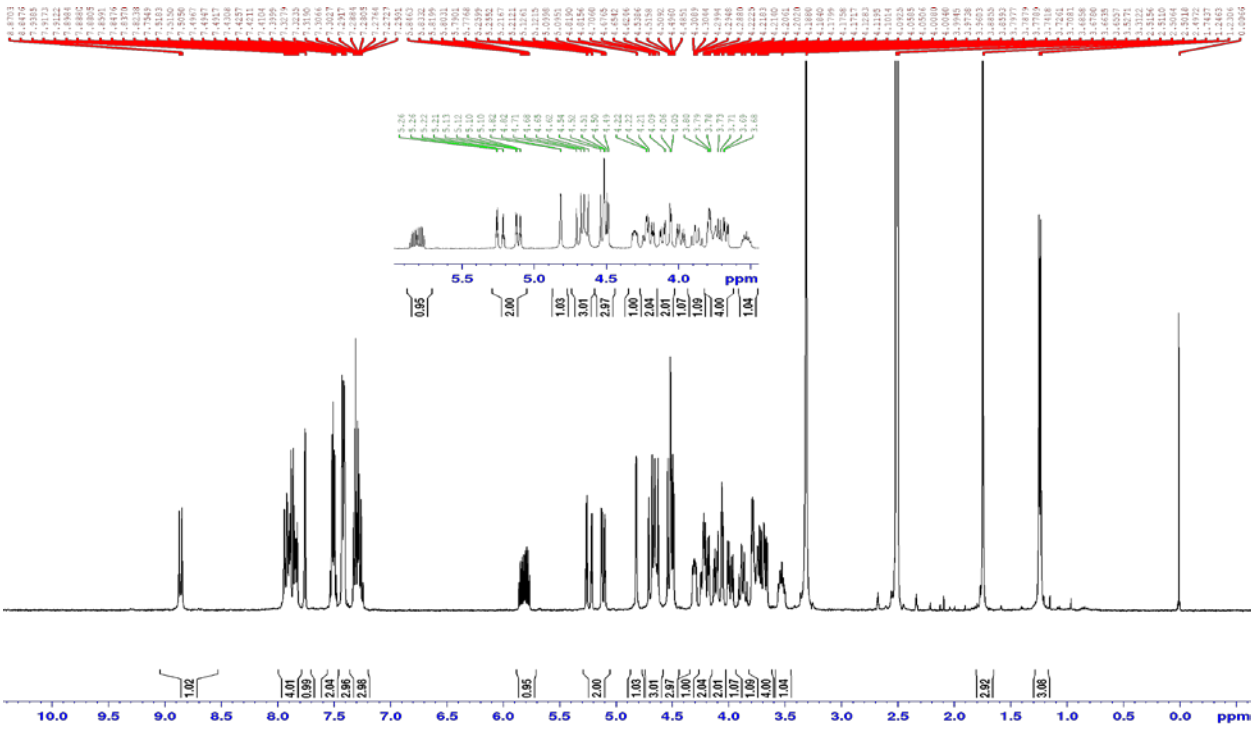

Figure. ^{1}H NMR (DMSO-d_6, 400 MHz) spectrum of **36**.

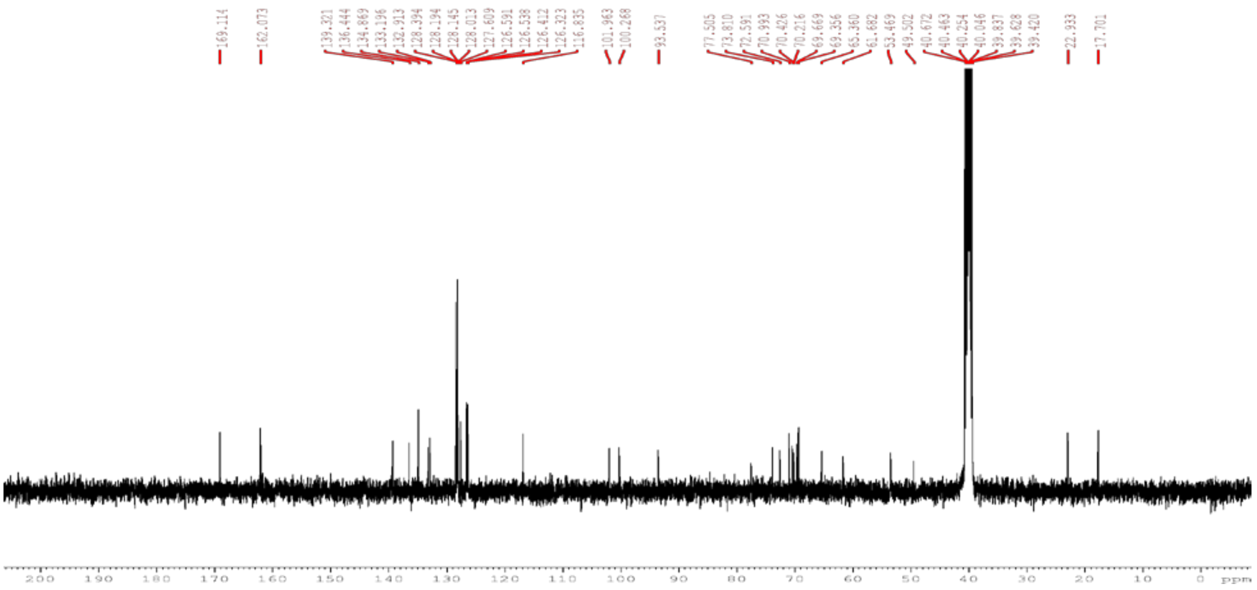

Figure. ^{13}C NMR (DMSO-d_6, 400 MHz) spectrum of **36**.

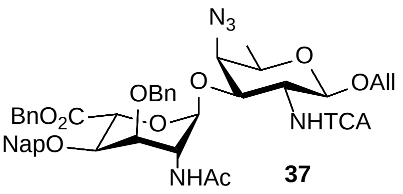

37

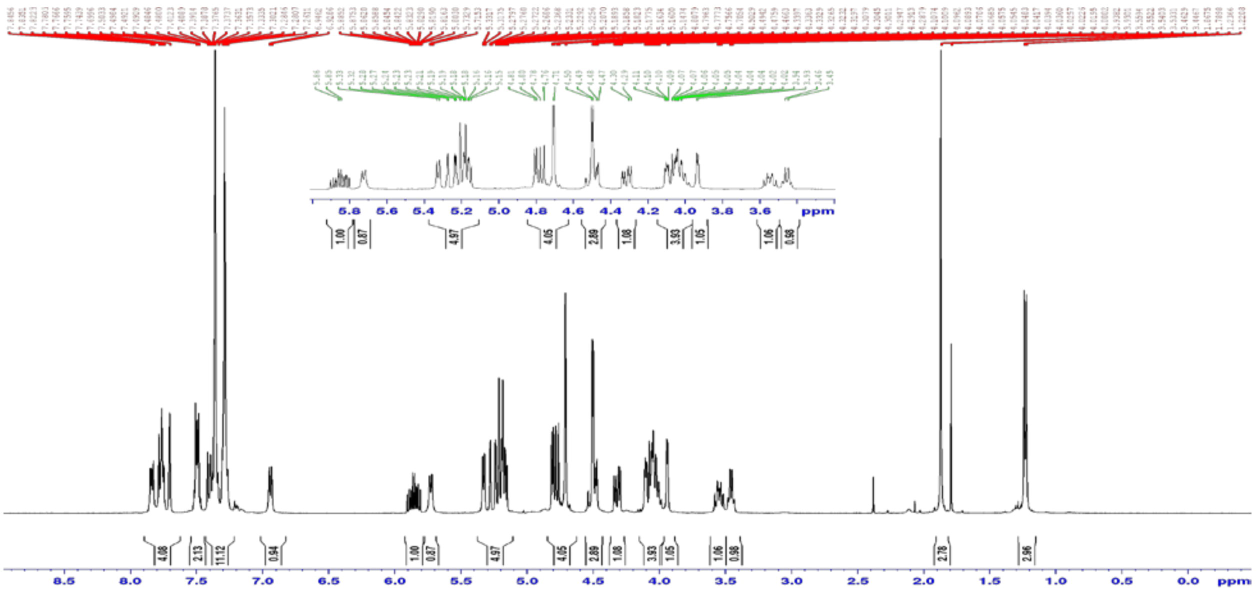

Figure. ^{1}H NMR ($CDCl_3$, 400 MHz) spectrum of **37**.

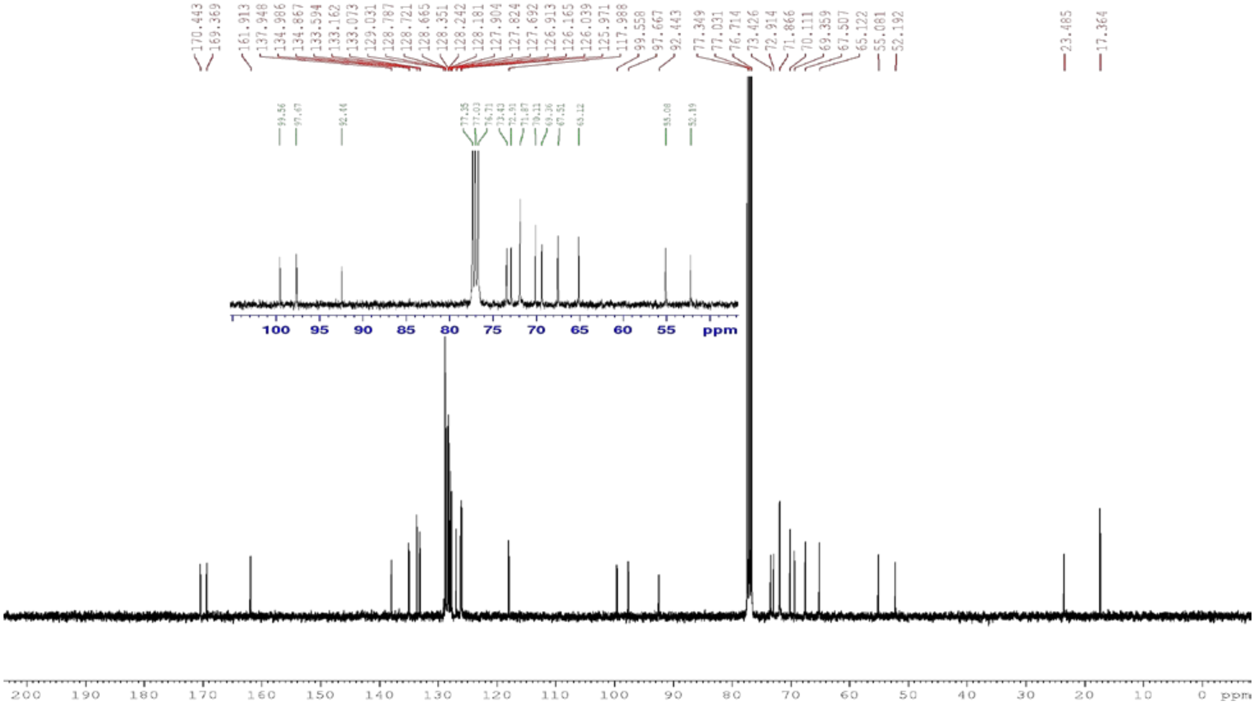

Figure. ^{13}C NMR ($CDCl_3$, 400 MHz) spectrum of **37**.

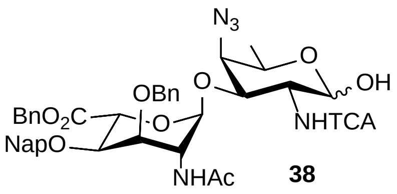

38

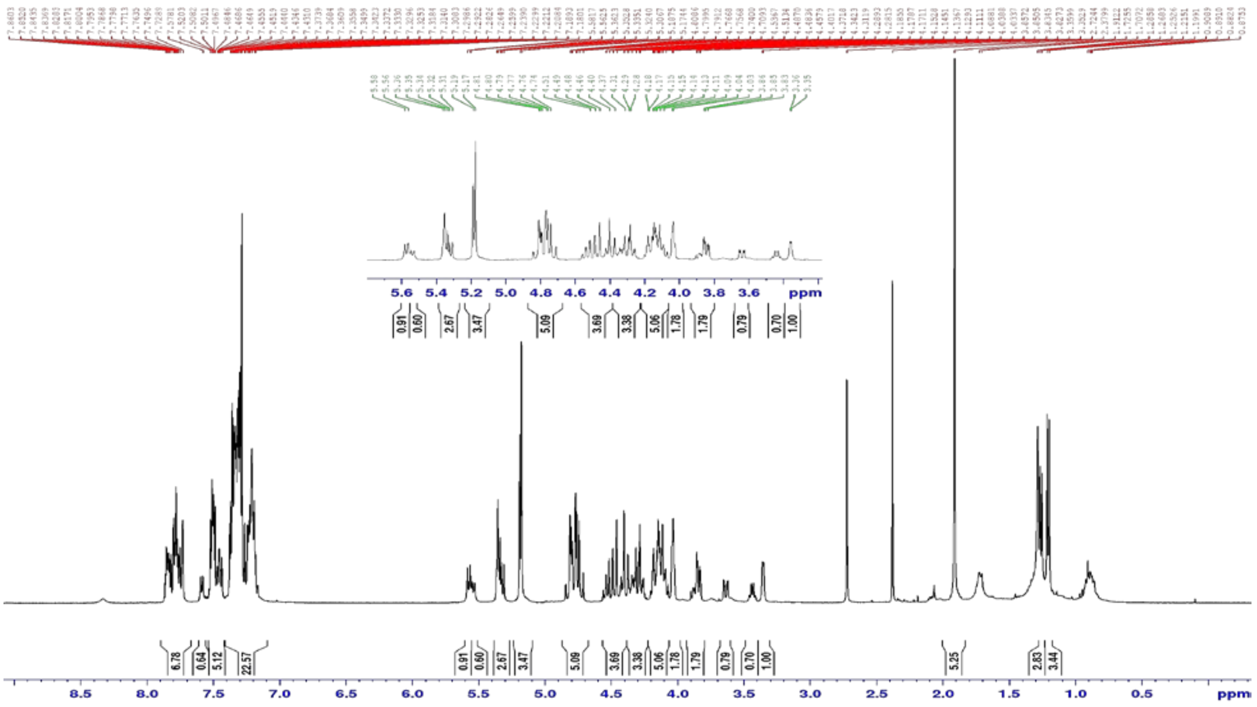

Figure. ^{1}H NMR ($CDCl_3$, 400 MHz) spectrum of **38**.

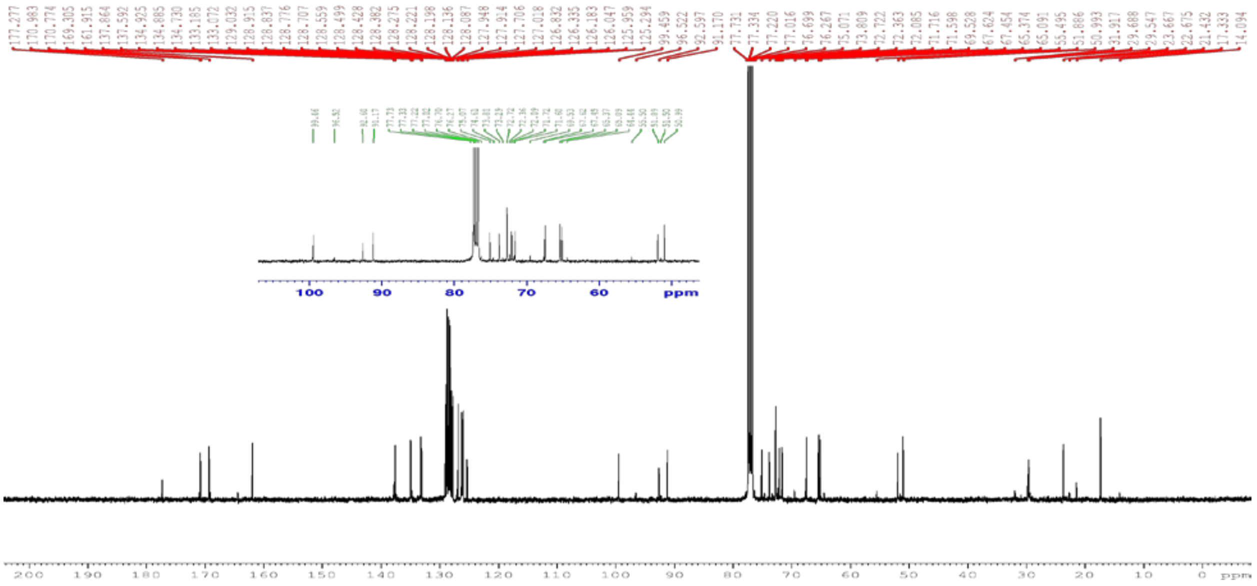

Figure. ^{13}C NMR ($CDCl_3$, 400 MHz) spectrum of **38**.

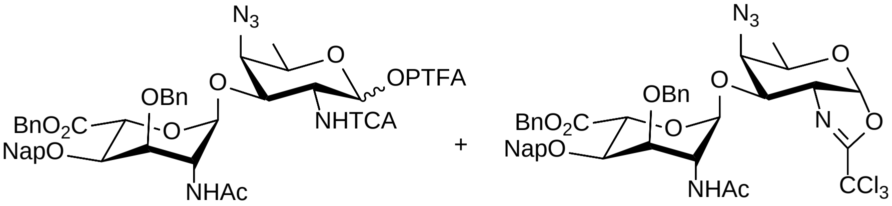

39 ~ 2:1 **40**

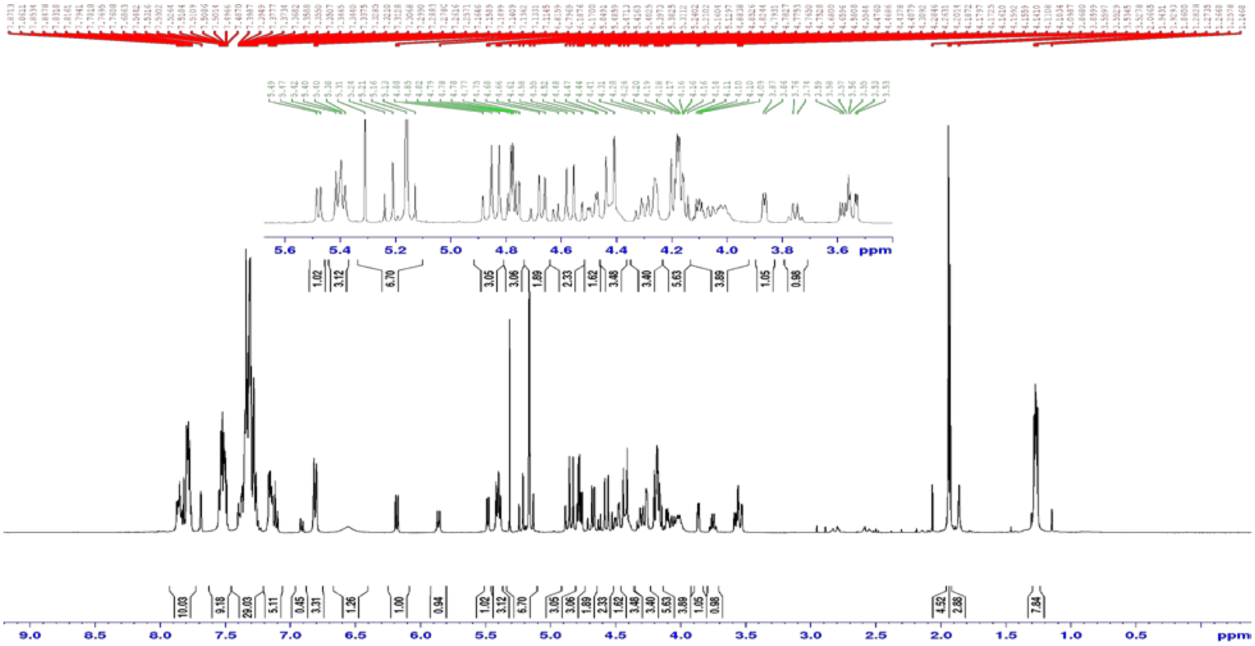

Figure. ^{1}H NMR ($CDCl_3$, 400 MHz) spectrum of **39** and **40**.

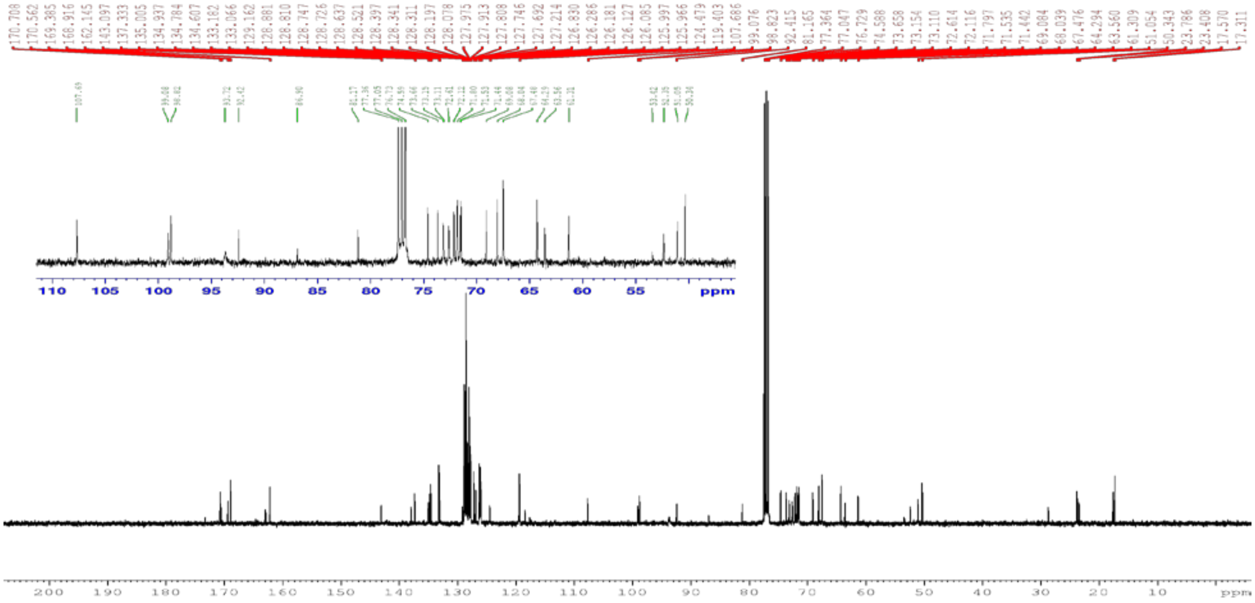

Figure. ^{13}C NMR ($CDCl_3$, 400 MHz) spectrum of **39** and **40**.

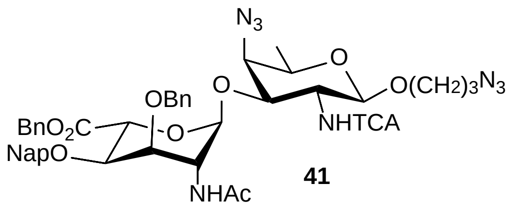

41

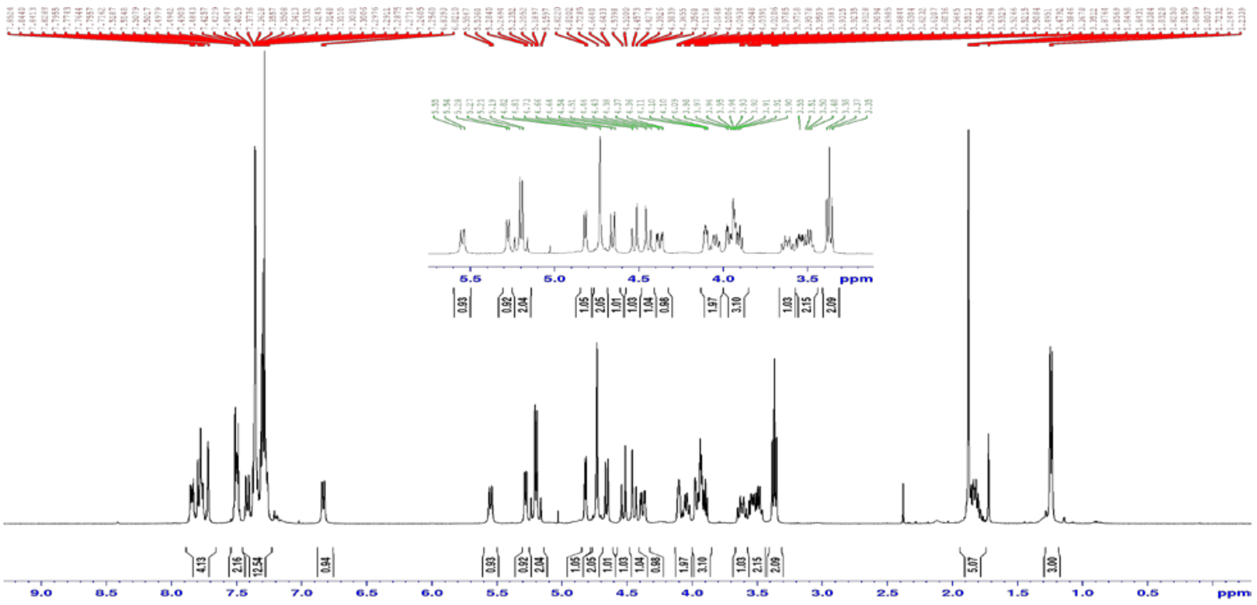

Figure. ^{1}H NMR ($CDCl_3$, 400 MHz) spectrum of **41**.

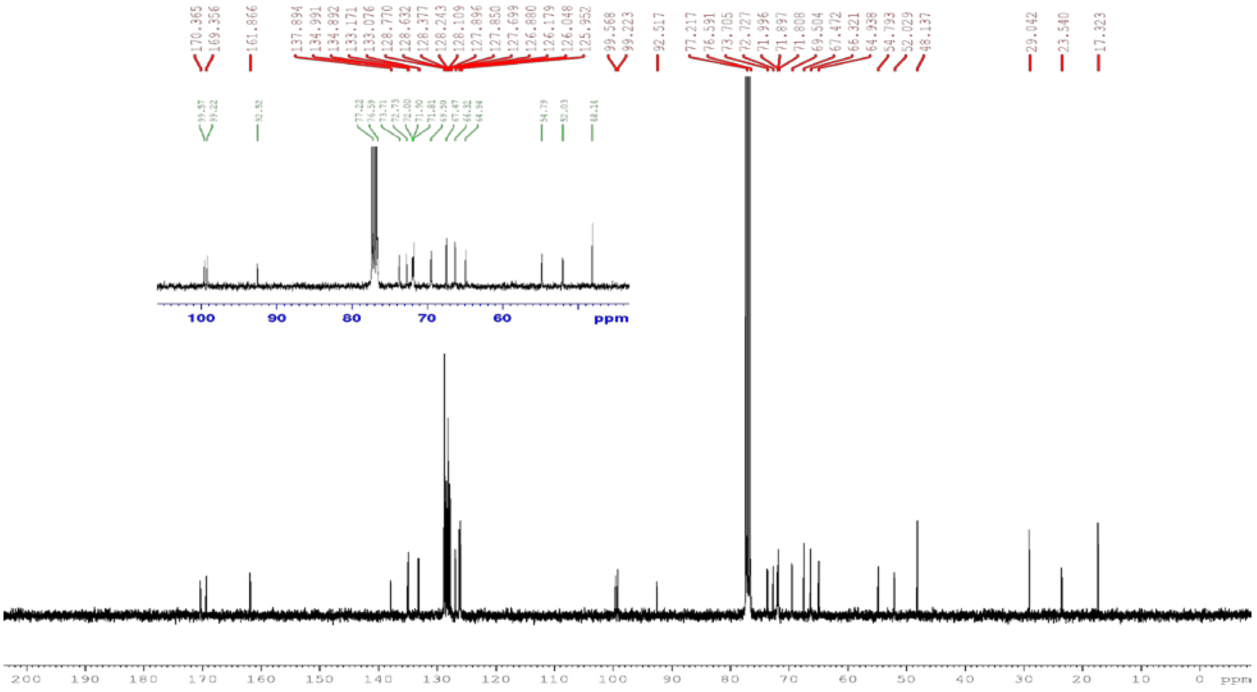

Figure. ^{13}C NMR ($CDCl_3$, 400 MHz) spectrum of **41**.

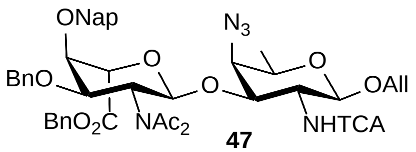

47

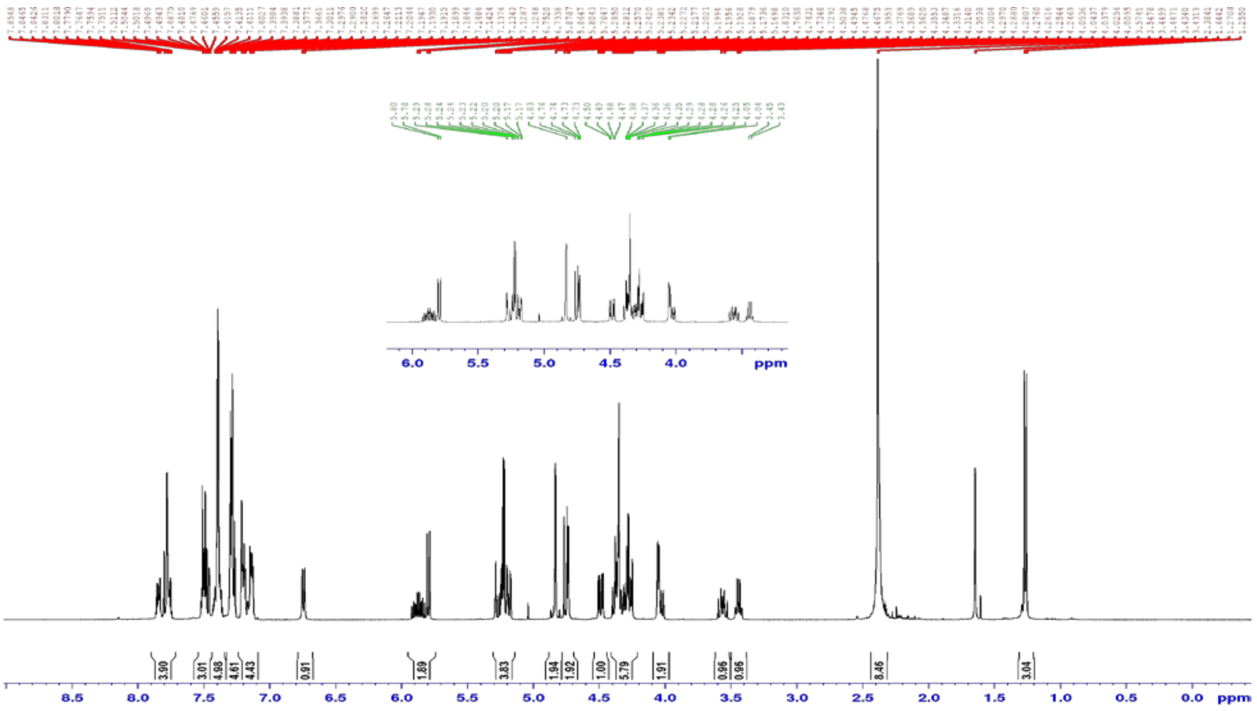

Figure. ^{1}H NMR ($CDCl_3$, 400 MHz) spectrum of **47**.

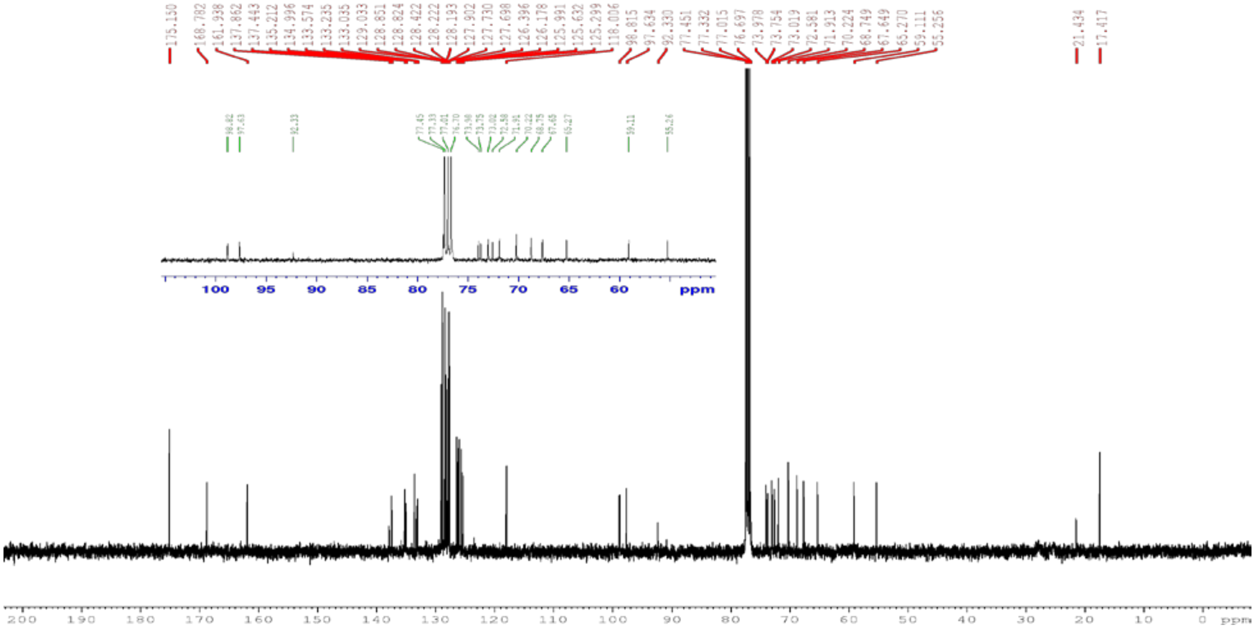

Figure. ^{13}C NMR ($CDCl_3$, 400 MHz) spectrum of **47**.

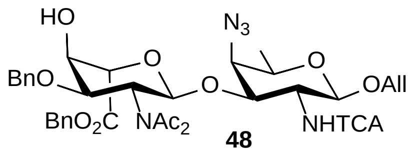

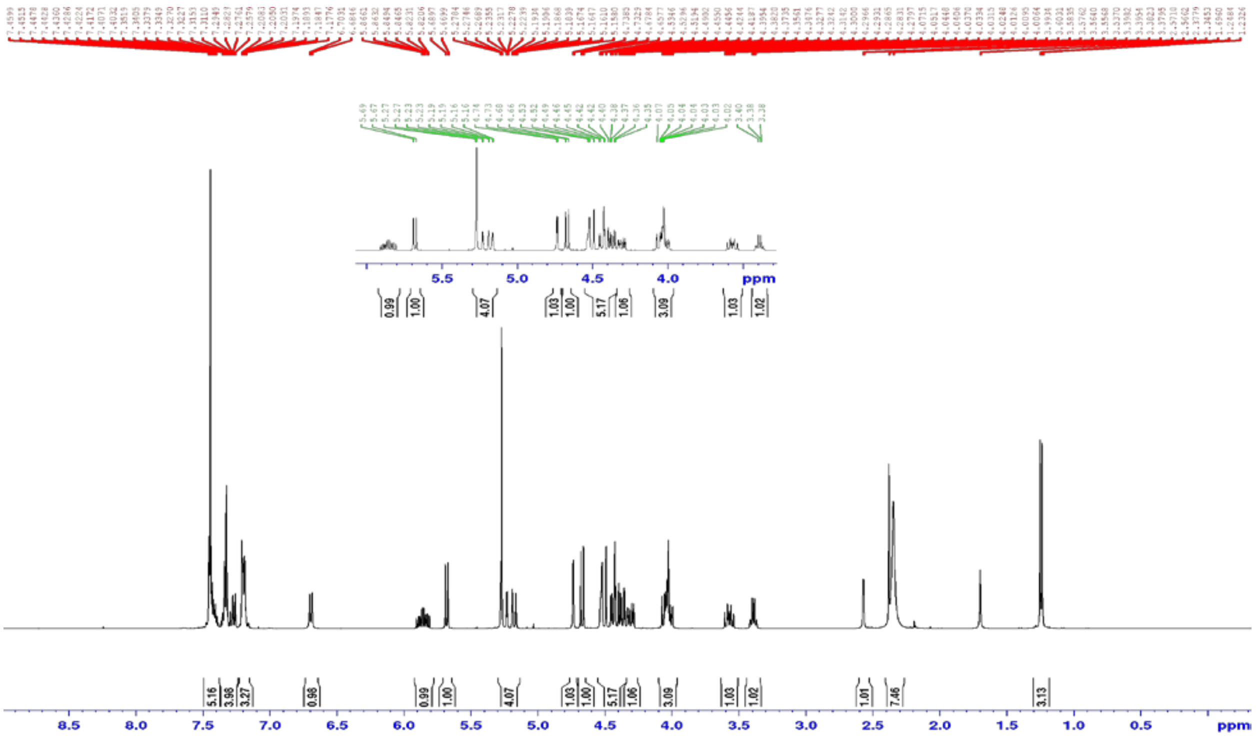

Figure. ^{1}H NMR (CDCl$_3$, 400 MHz) spectrum of **48**.

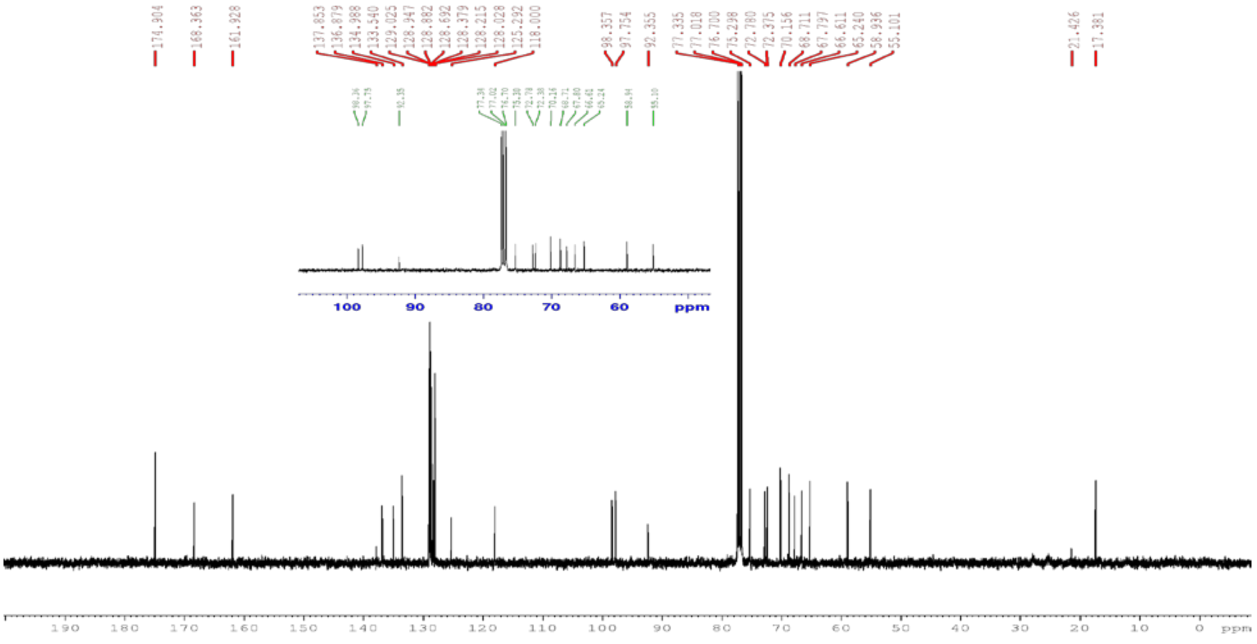

Figure. ^{13}C NMR (CDCl$_3$, 400 MHz) spectrum of **48**.

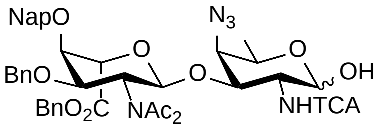

49

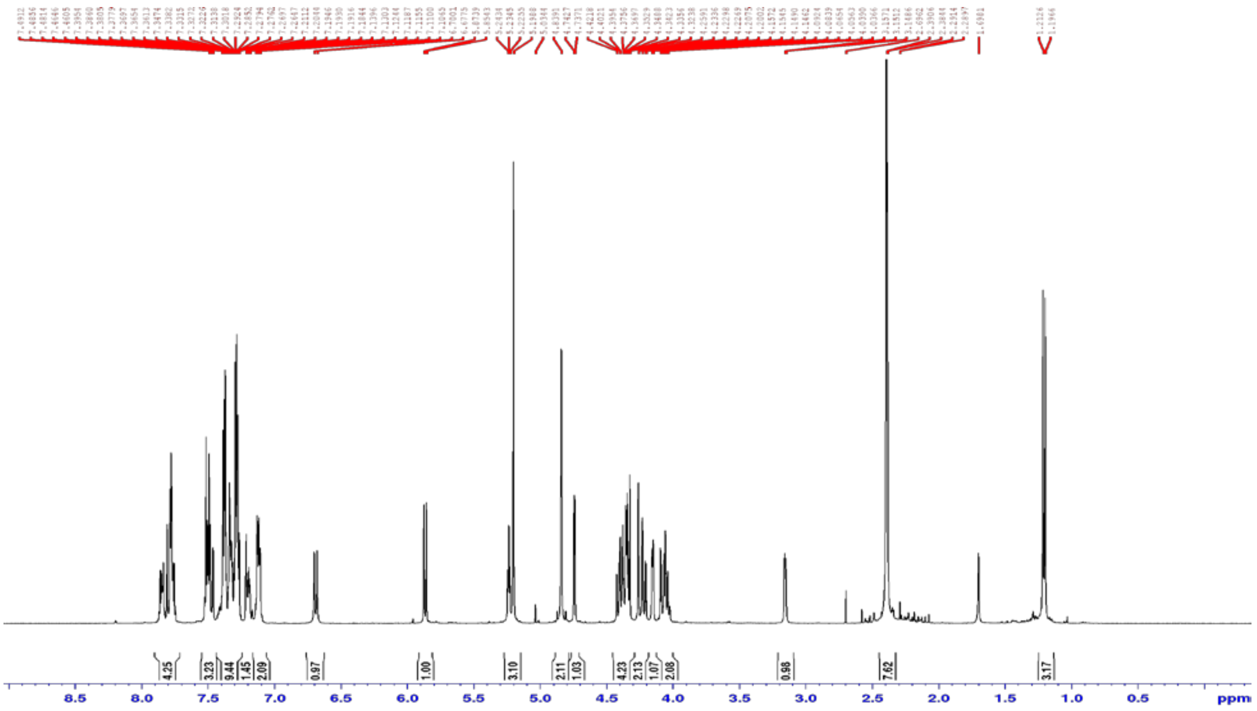

Figure. ^{1}H NMR ($CDCl_3$, 400 MHz) spectrum of **49**.

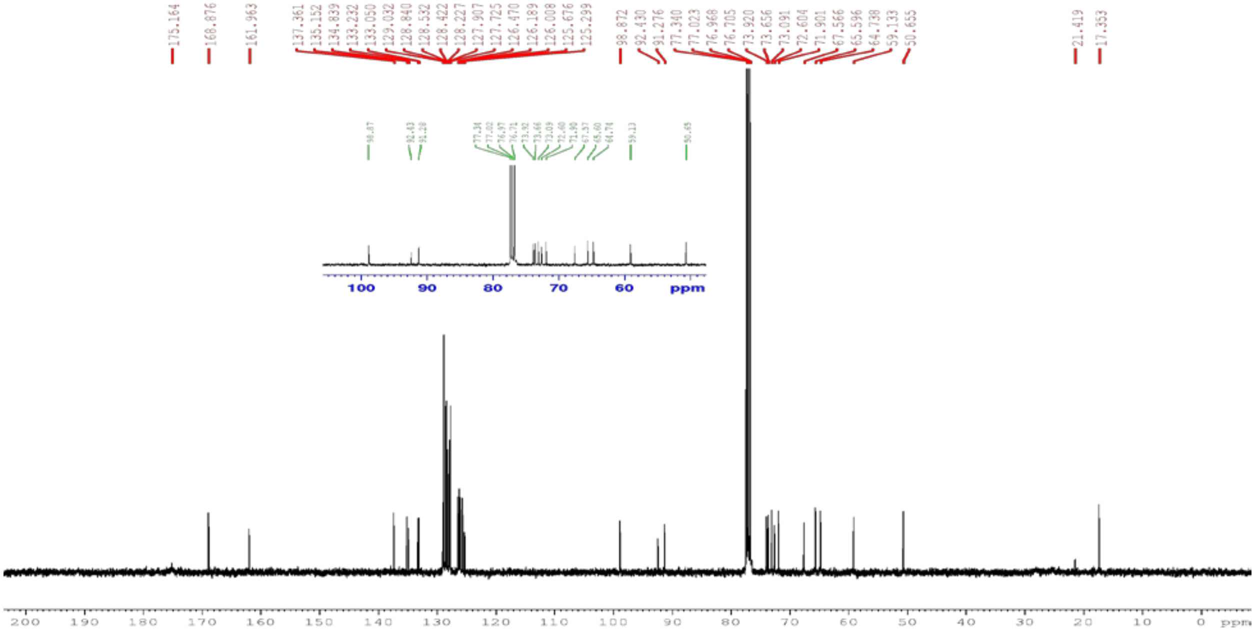

Figure. ^{13}C NMR ($CDCl_3$, 400 MHz) spectrum of **49** (α-isomer).

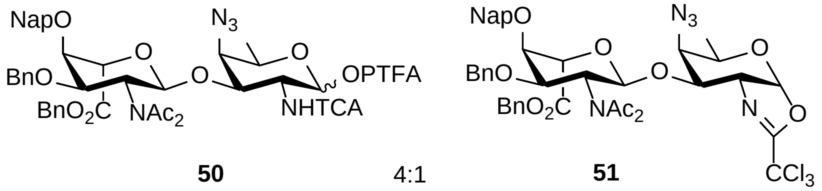

50 4:1 **51**

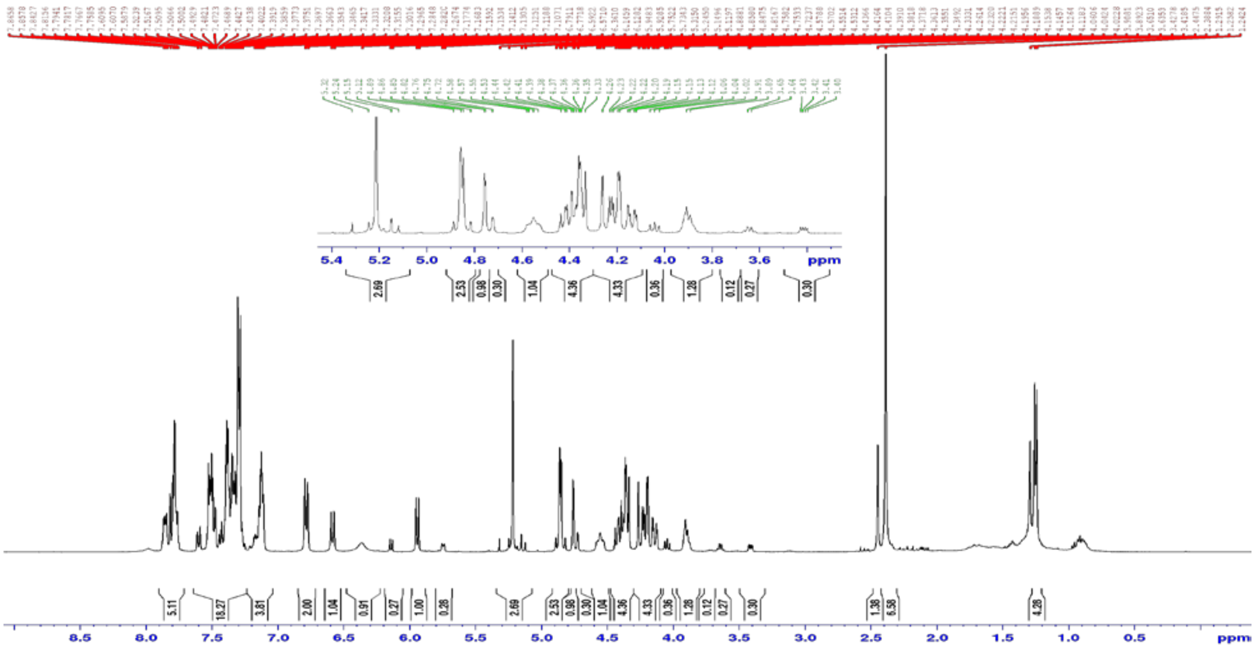

Figure. ^{1}H NMR ($CDCl_3$, 400 MHz) spectrum of **50** and **51**.

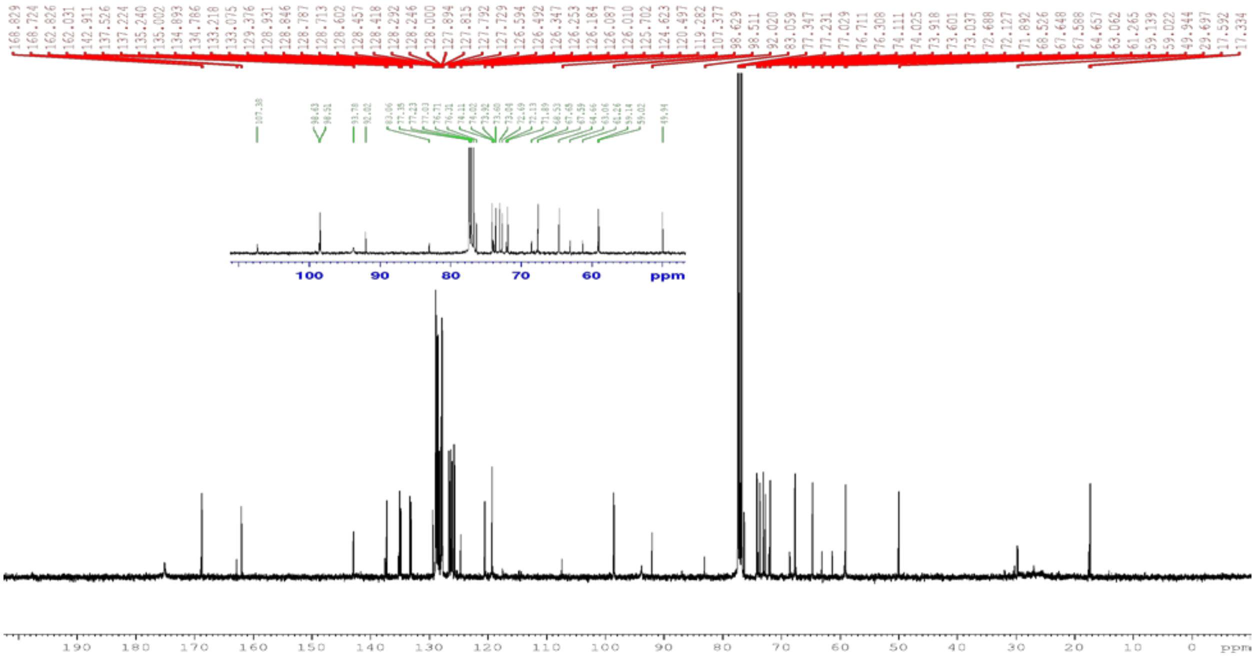

Figure. ^{13}C NMR ($CDCl_3$, 400 MHz) spectrum of **50** and **51**.

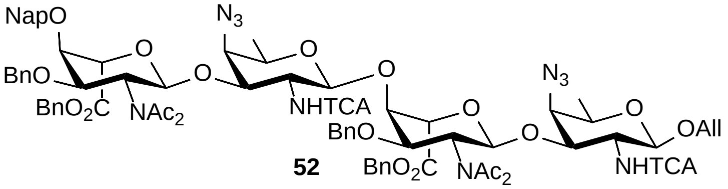

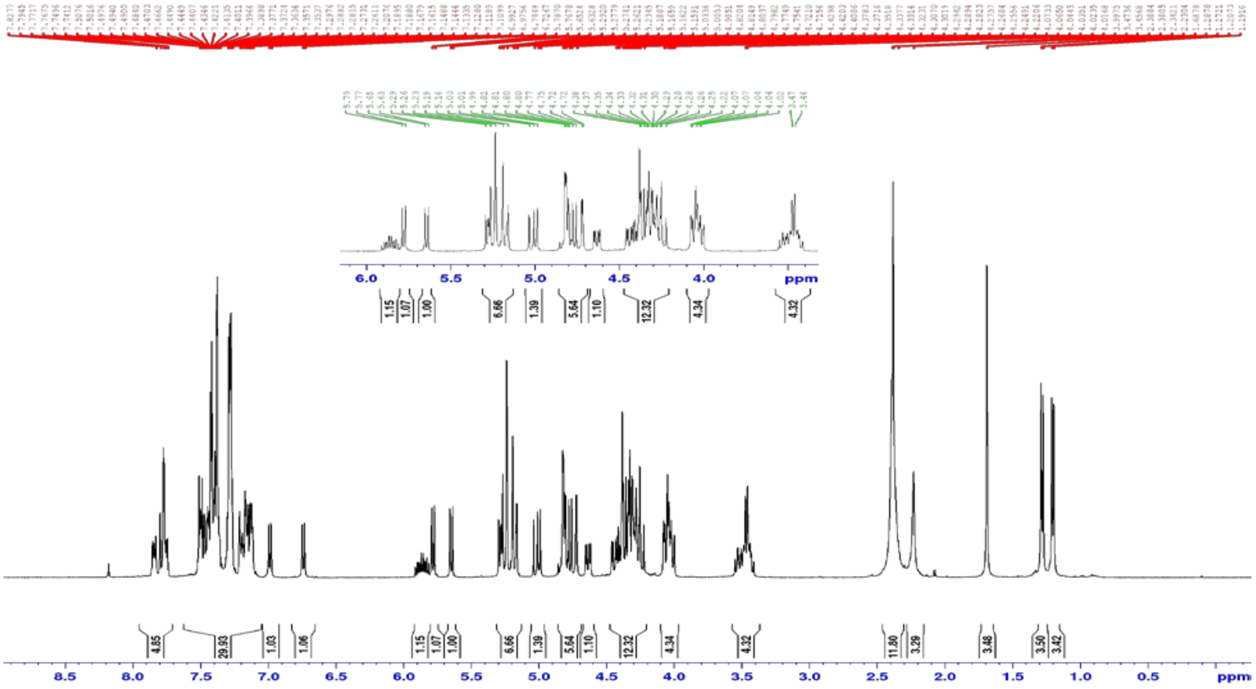

Figure. ^{1}H NMR (CDCl$_3$, 400 MHz) spectrum of **52**.

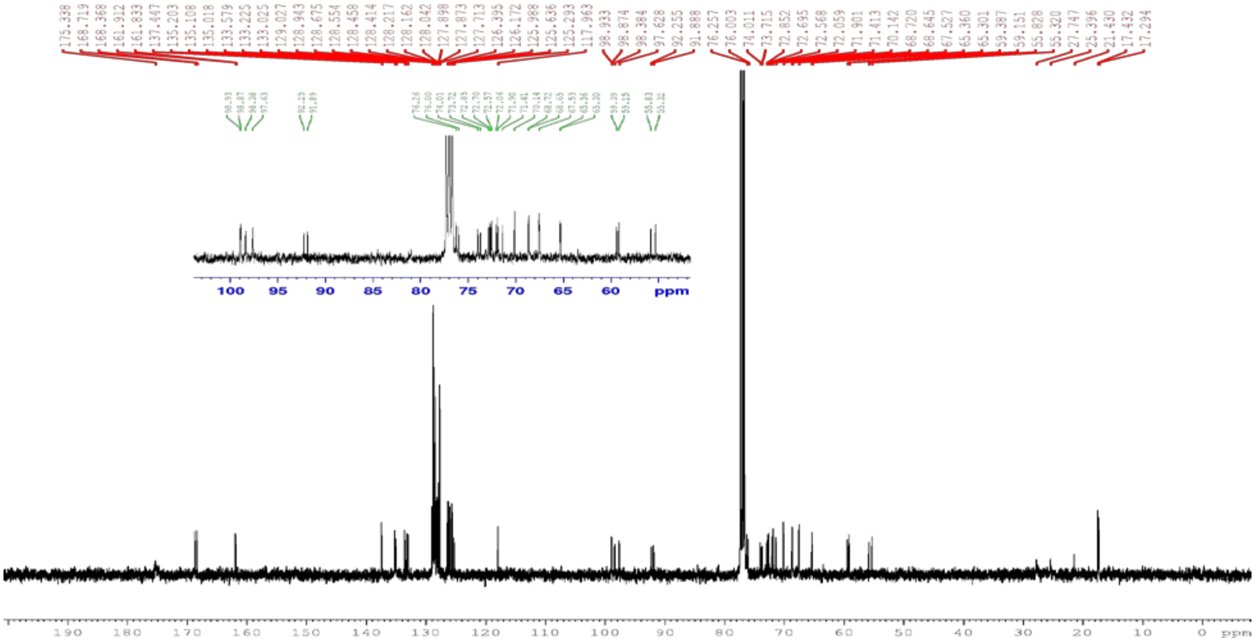

Figure. ^{13}C NMR (CDCl$_3$, 400 MHz) spectrum of **52**.

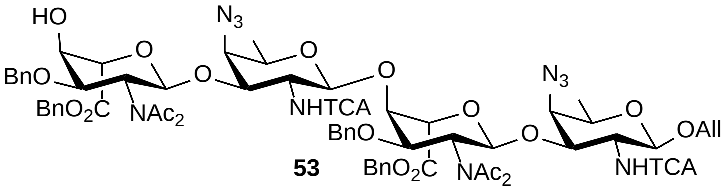

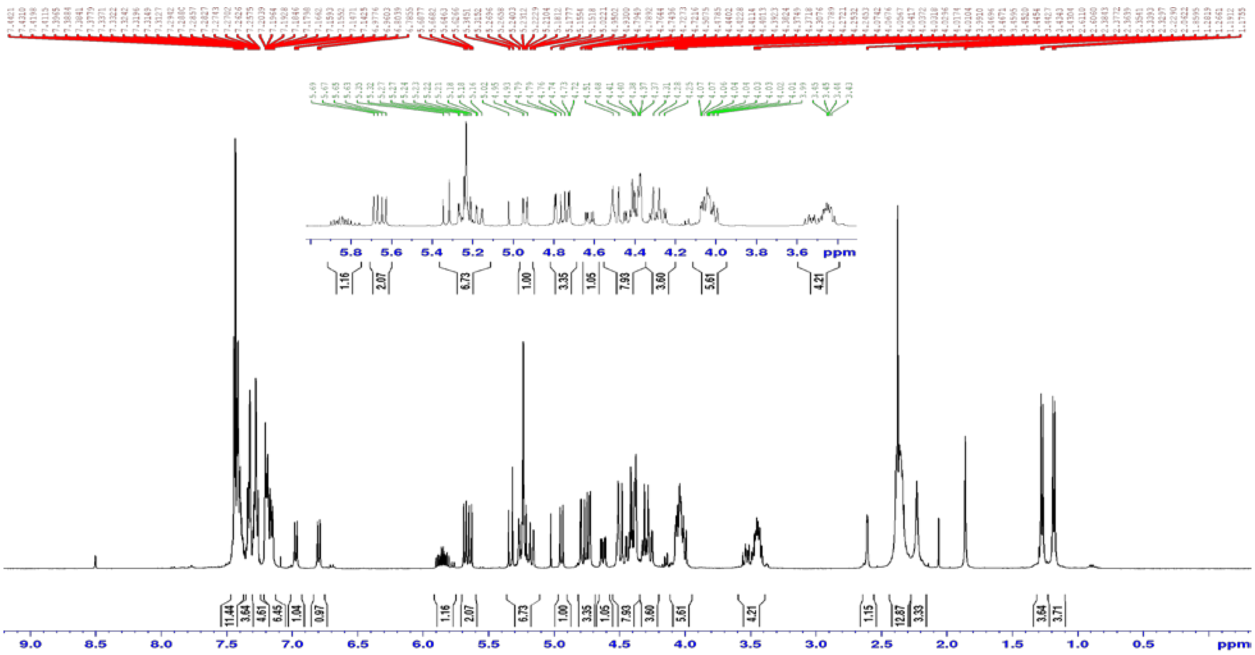

Figure. ^{1}H NMR ($CDCl_3$, 400 MHz) spectrum of **53**.

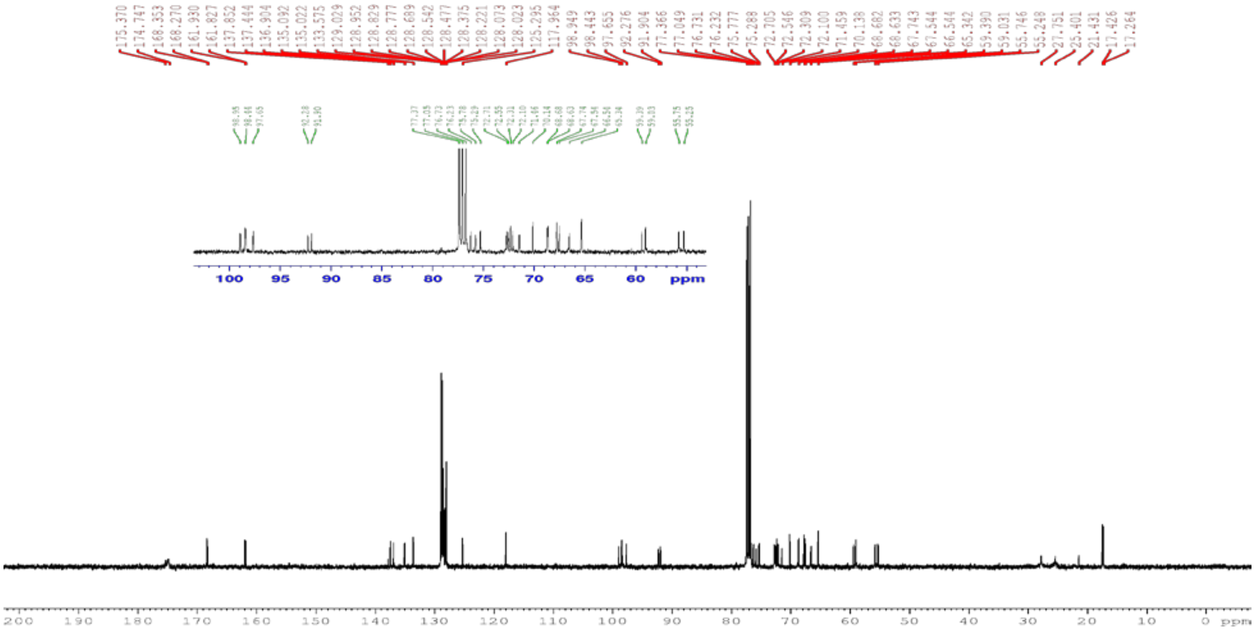

Figure. ^{13}C NMR ($CDCl_3$, 400 MHz) spectrum of **53**.

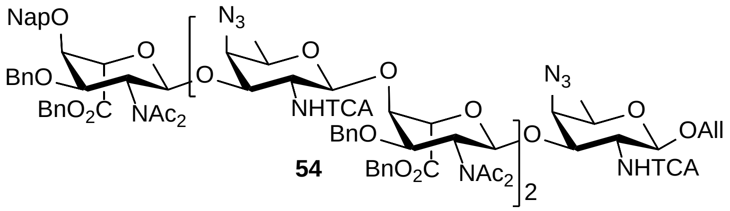

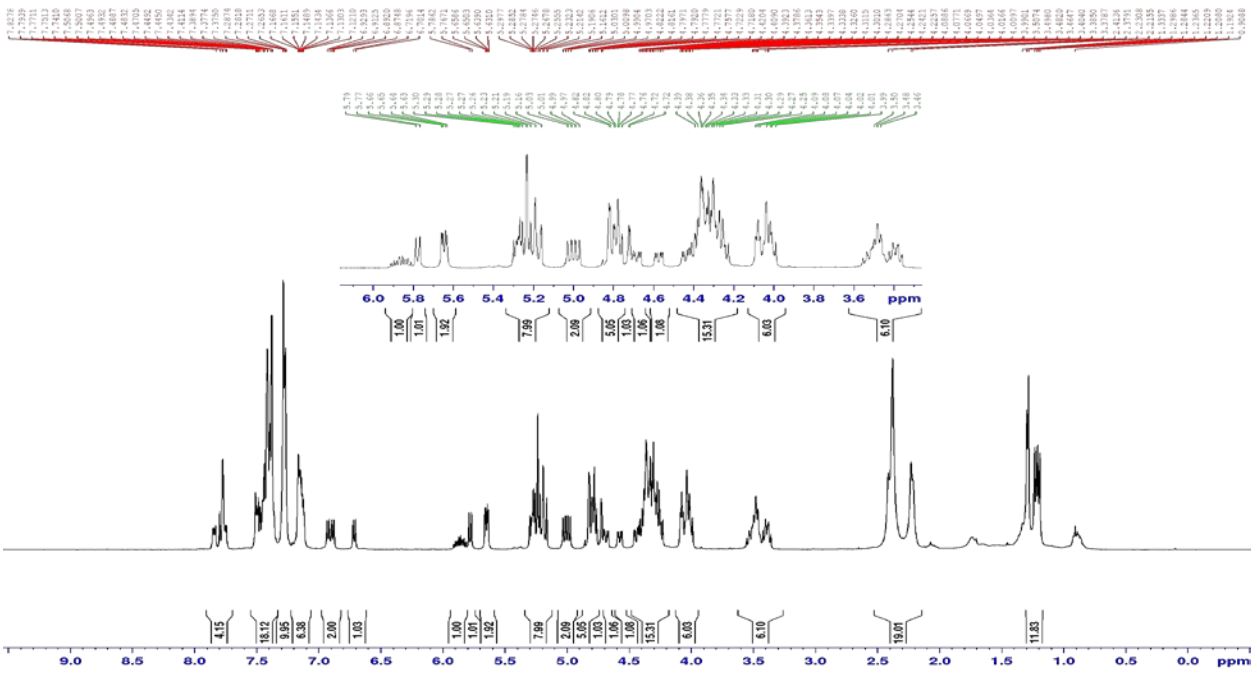

Figure. ^{1}H NMR ($CDCl_3$, 400 MHz) spectrum of **54**.

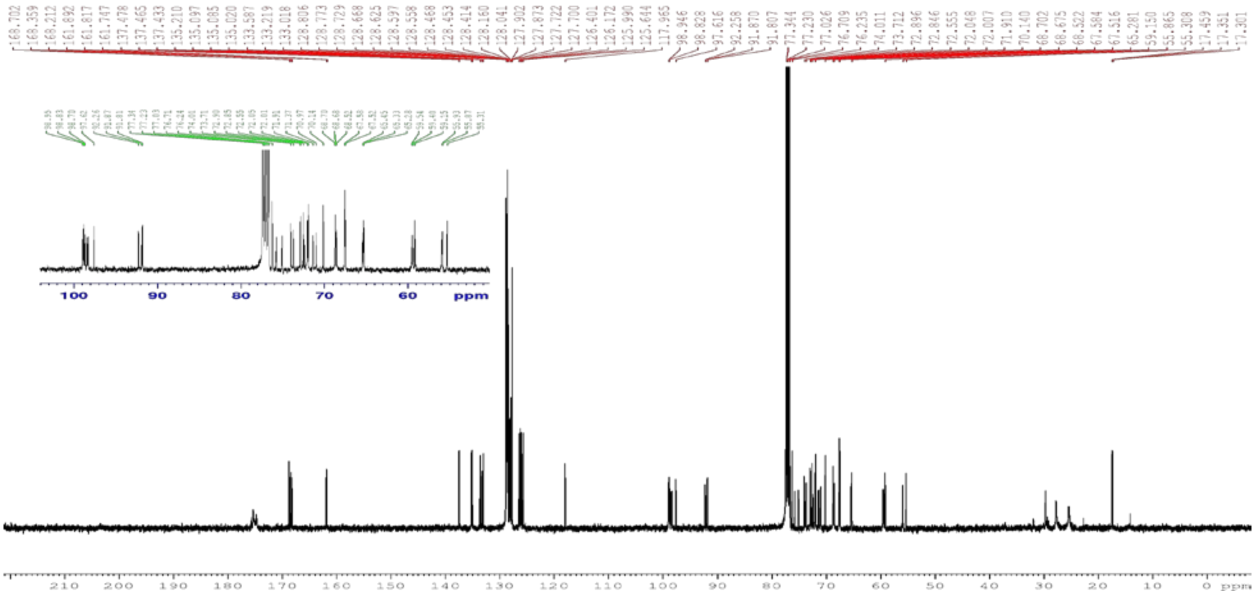

Figure. ^{13}C NMR ($CDCl_3$, 400 MHz) spectrum of **54**.

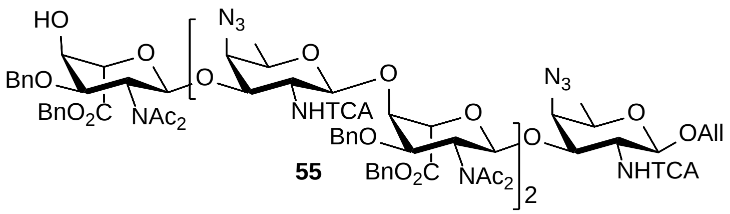

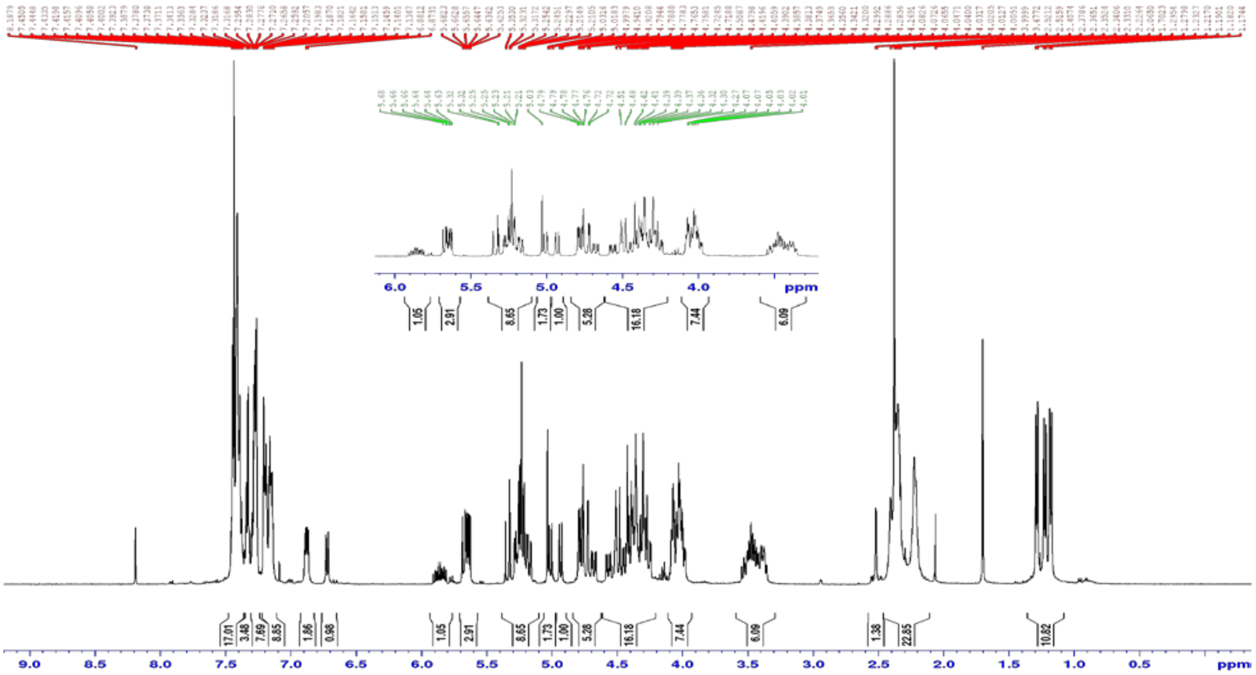

Figure. ^{1}H NMR ($CDCl_3$, 400 MHz) spectrum of **55**.

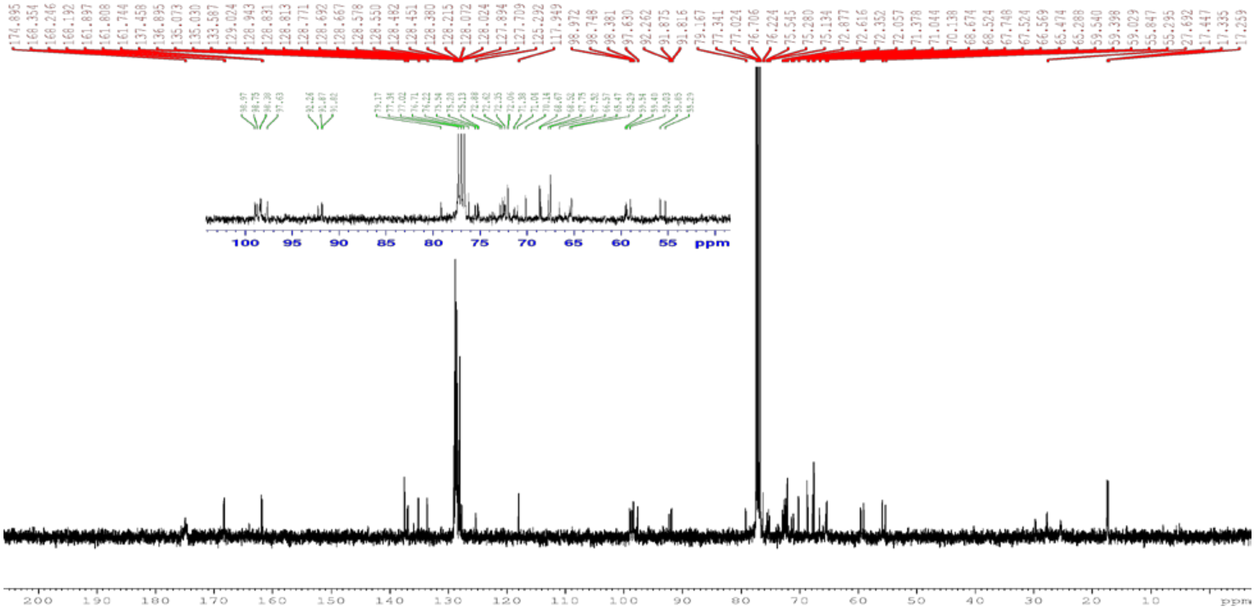

Figure. ^{13}C NMR ($CDCl_3$, 400 MHz) spectrum of **55**.

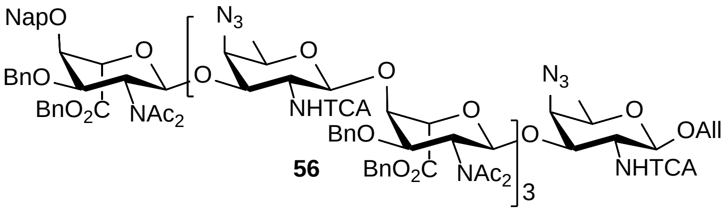

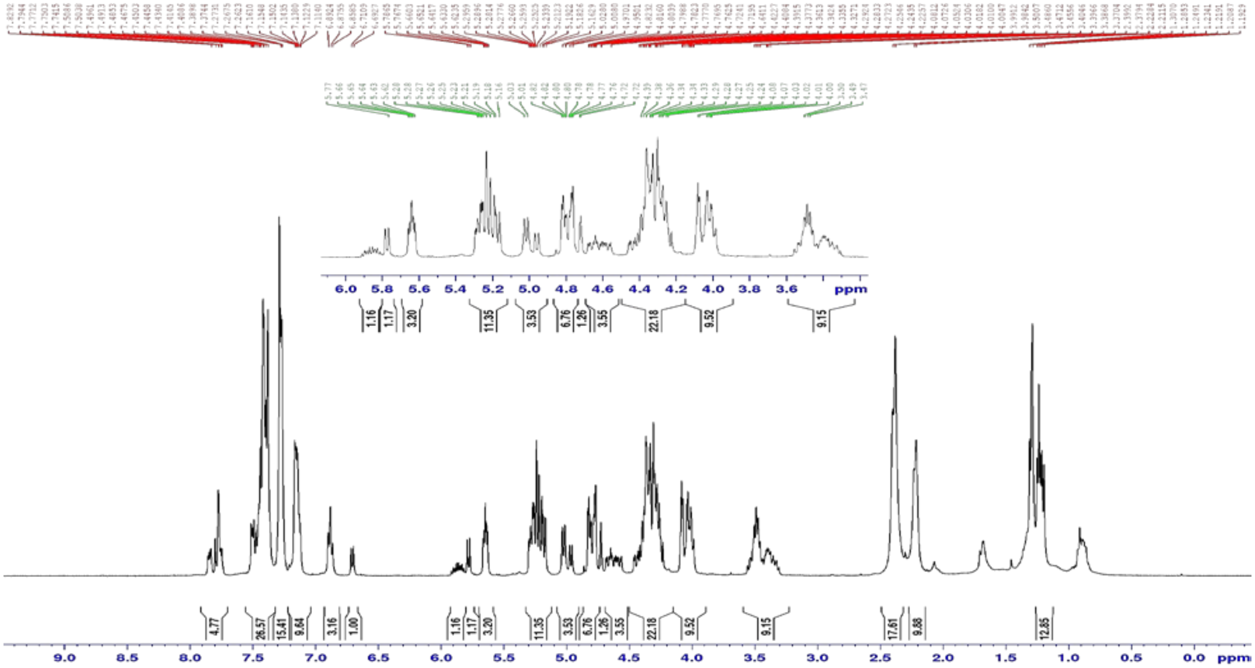

Figure. ^{1}H NMR ($CDCl_3$, 400 MHz) spectrum of **56**.

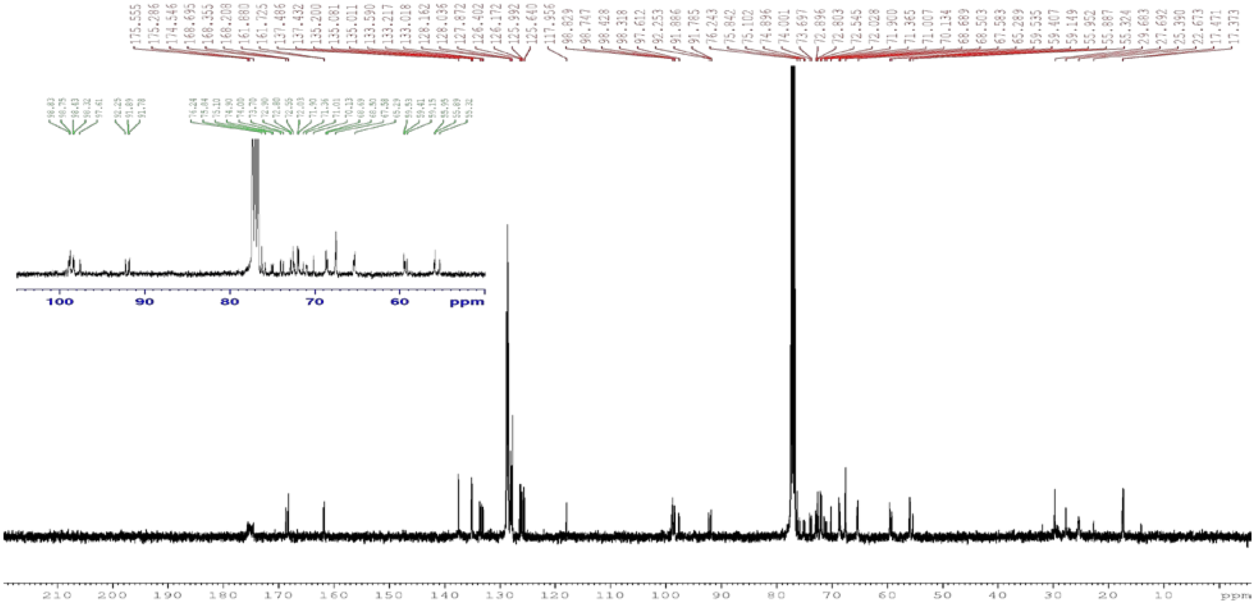

Figure. ^{13}C NMR ($CDCl_3$, 400 MHz) spectrum of **56**.

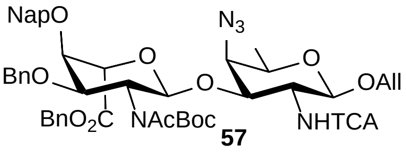

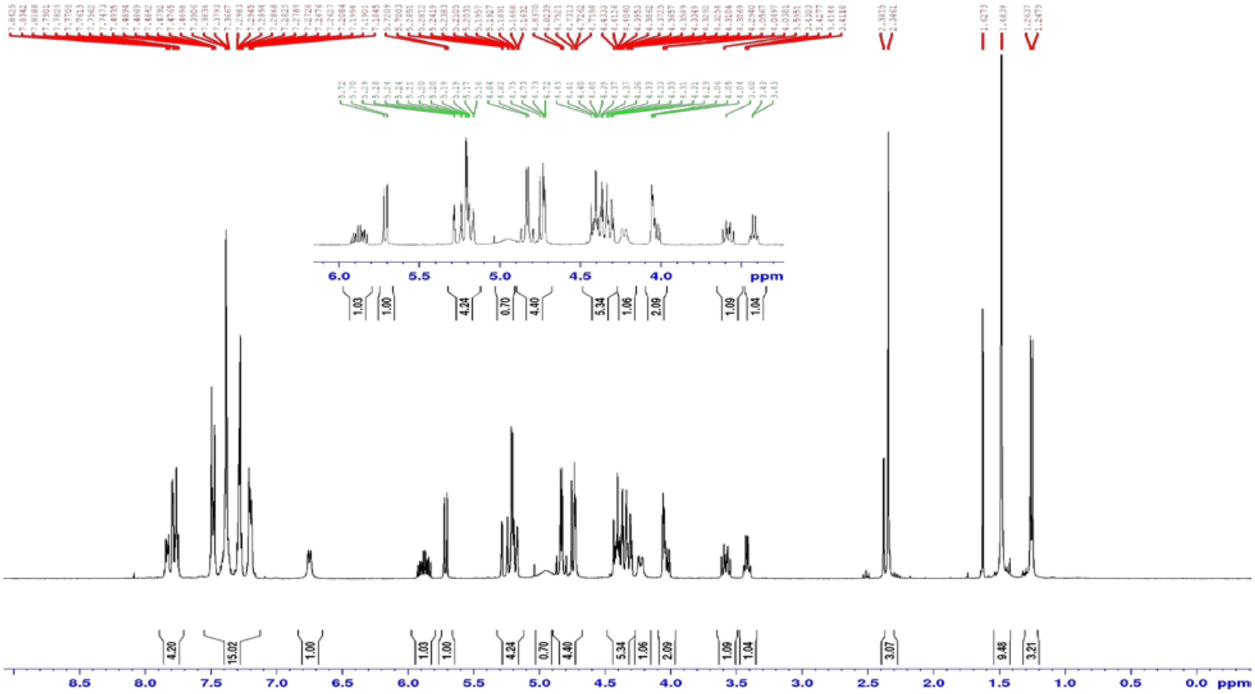

Figure. ^{1}H NMR ($CDCl_3$, 400 MHz) spectrum of **57**.

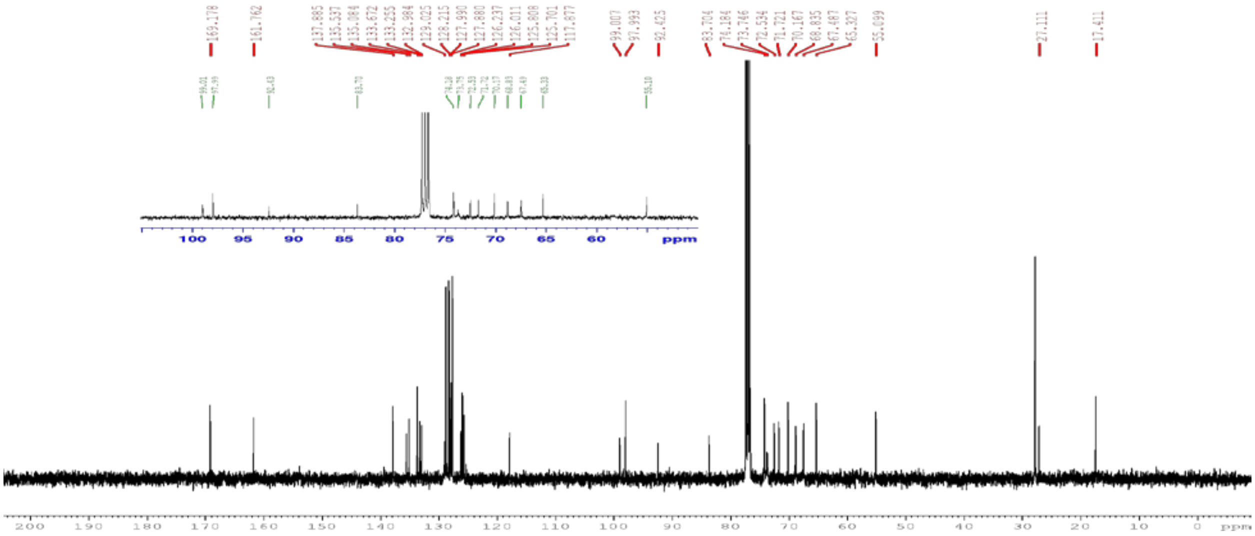

Figure. ^{13}C NMR ($CDCl_3$, 400 MHz) spectrum of **57**.

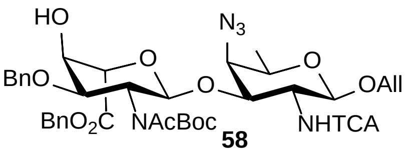

58

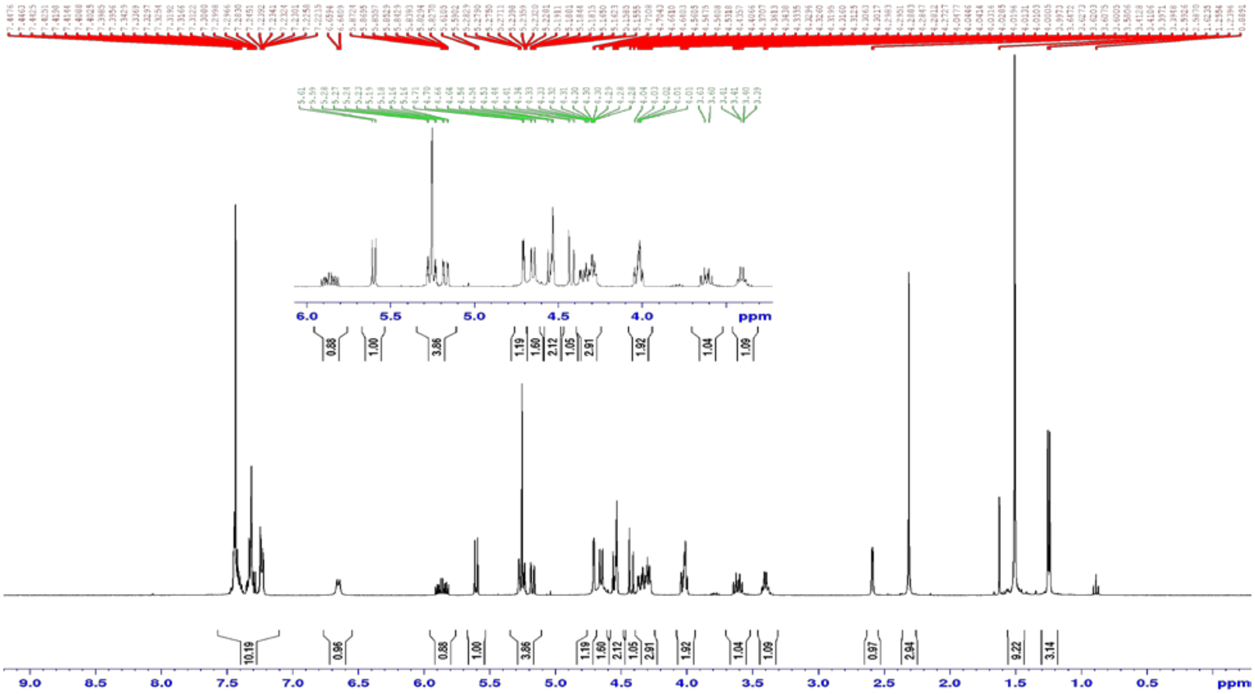

Figure. ^{1}H NMR ($CDCl_3$, 400 MHz) spectrum of **58**.

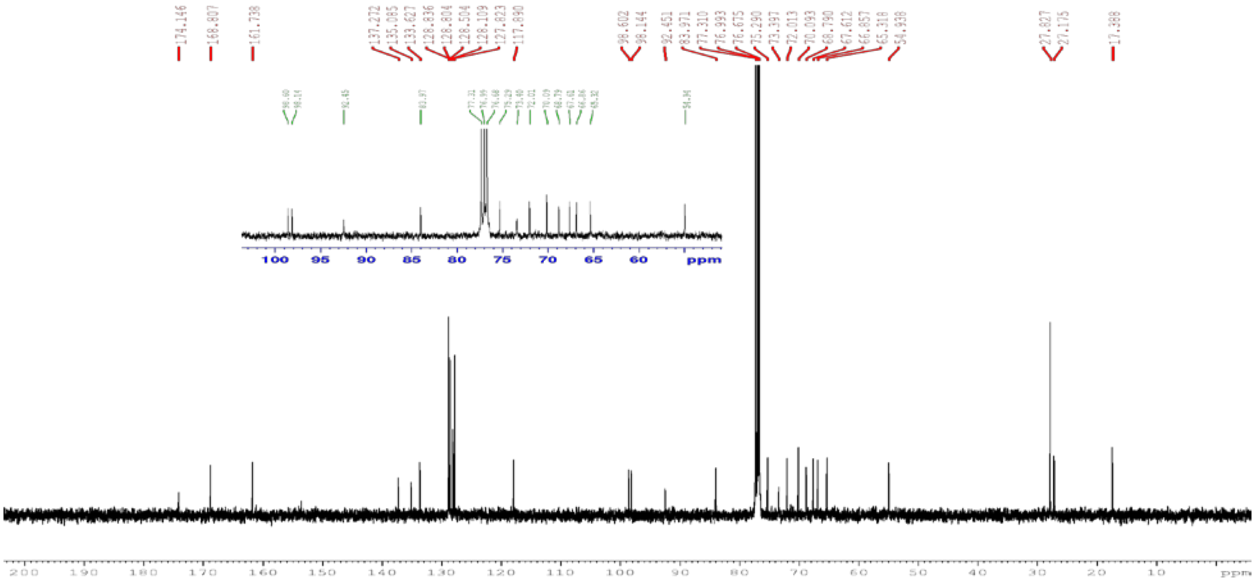

Figure. ^{13}C NMR ($CDCl_3$, 400 MHz) spectrum of **58**.

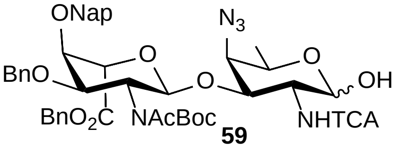

59

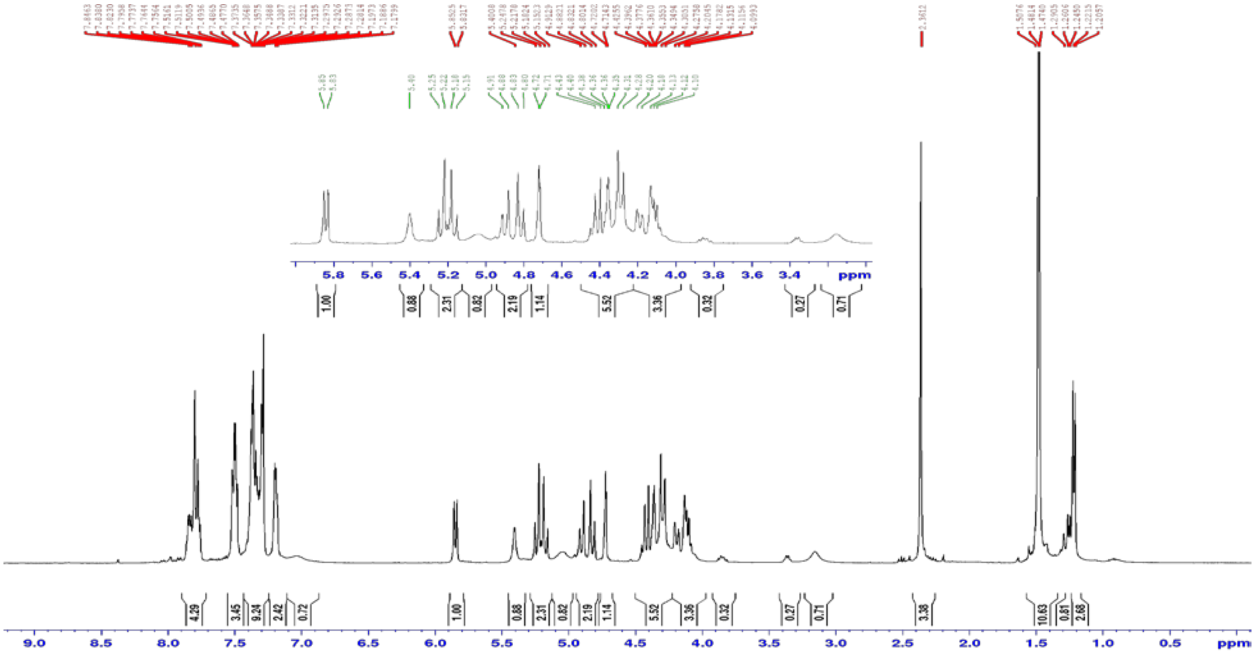

Figure. ^{1}H NMR ($CDCl_3$, 400 MHz) spectrum of **59**.

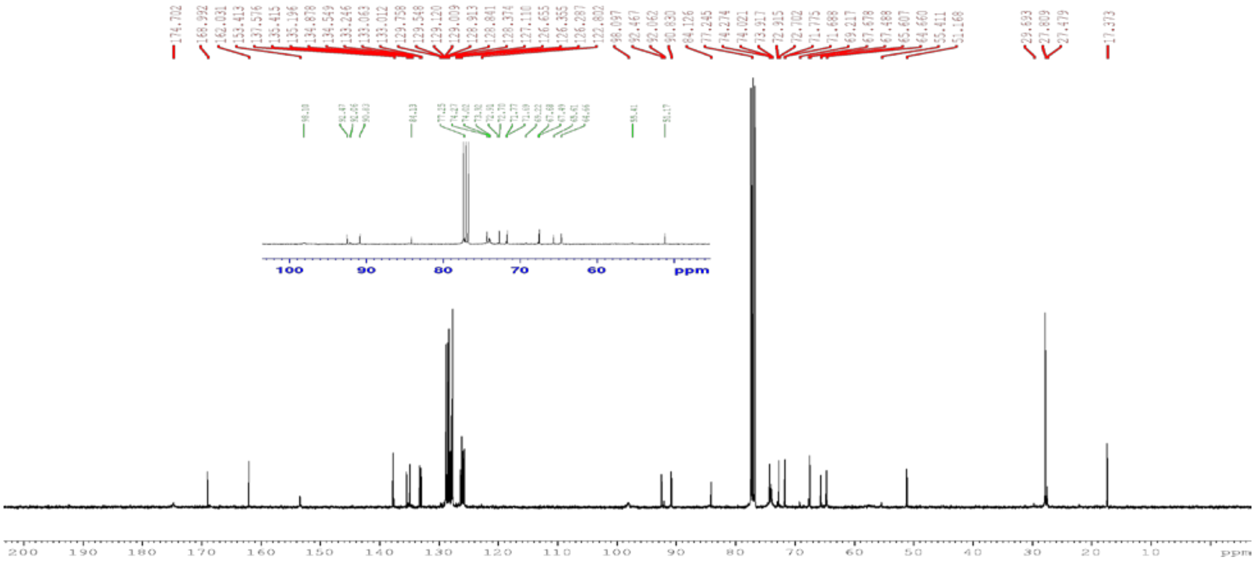

Figure. ^{13}C NMR ($CDCl_3$, 400 MHz) spectrum of **59**.

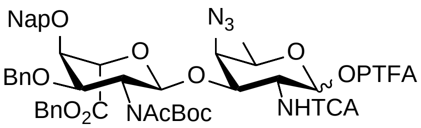

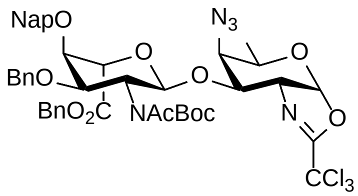

60

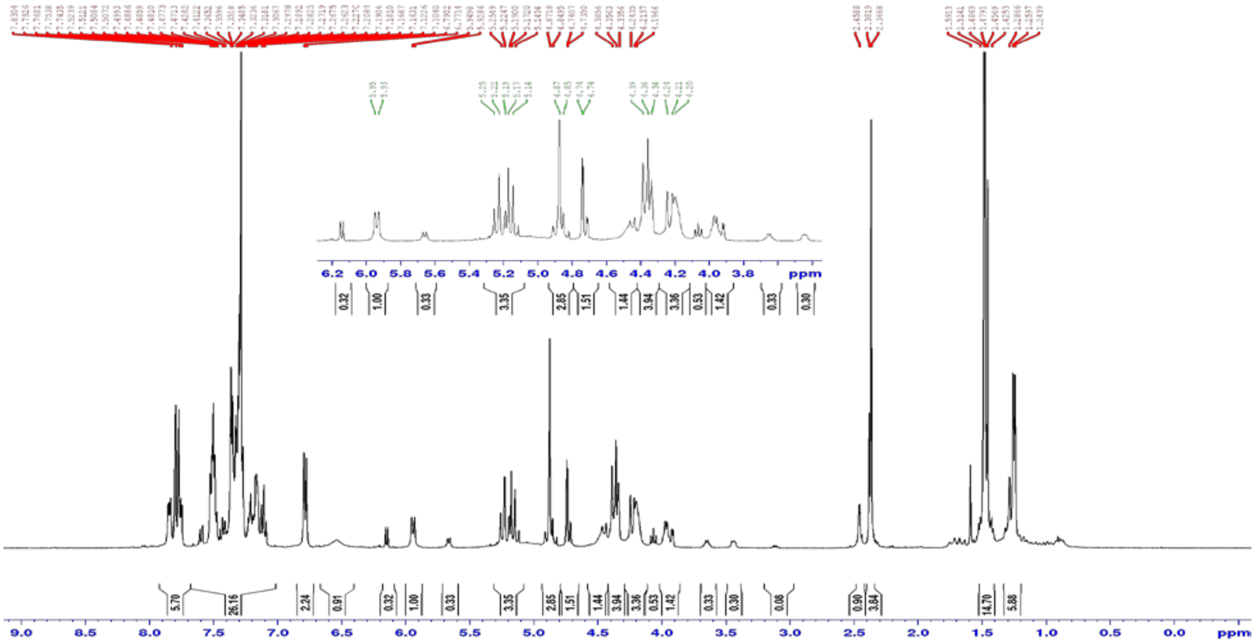

Figure. ^{1}H NMR ($CDCl_3$, 400 MHz) spectrum of **60**.

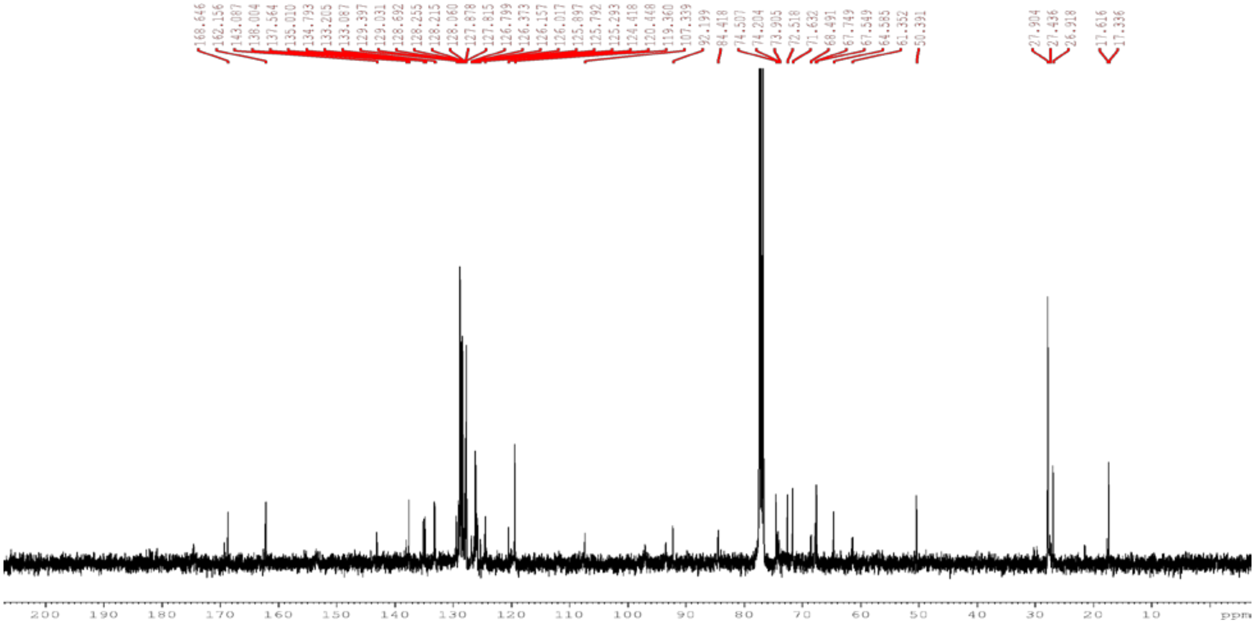

Figure. ^{13}C NMR ($CDCl_3$, 400 MHz) spectrum of **60**.

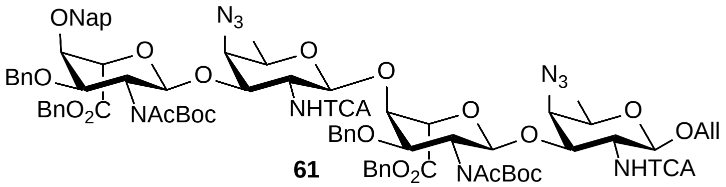

61

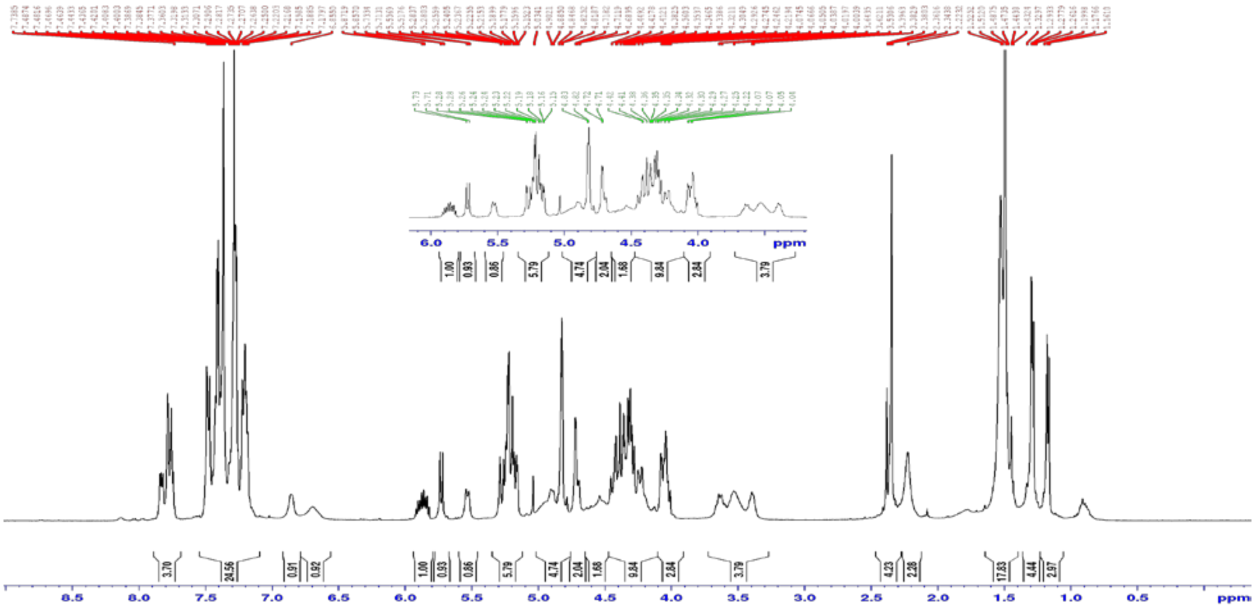

Figure. ^{1}H NMR ($CDCl_3$, 400 MHz) spectrum of **61**.

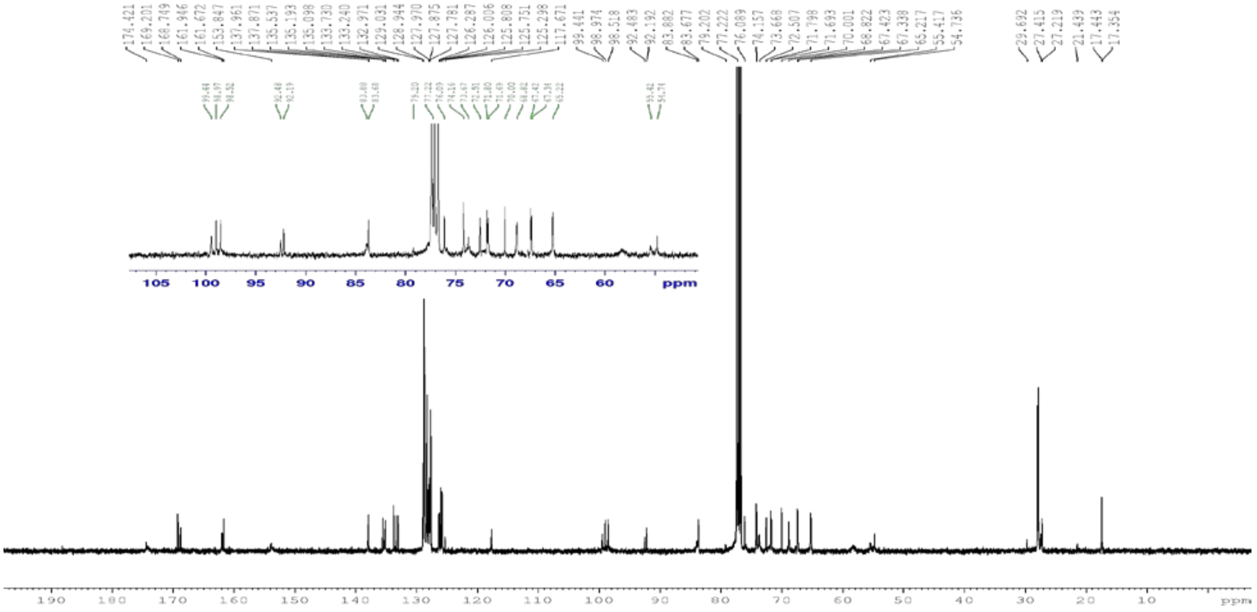

Figure. ^{13}C NMR ($CDCl_3$, 400 MHz) spectrum of **61**.

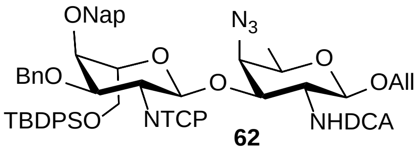

62

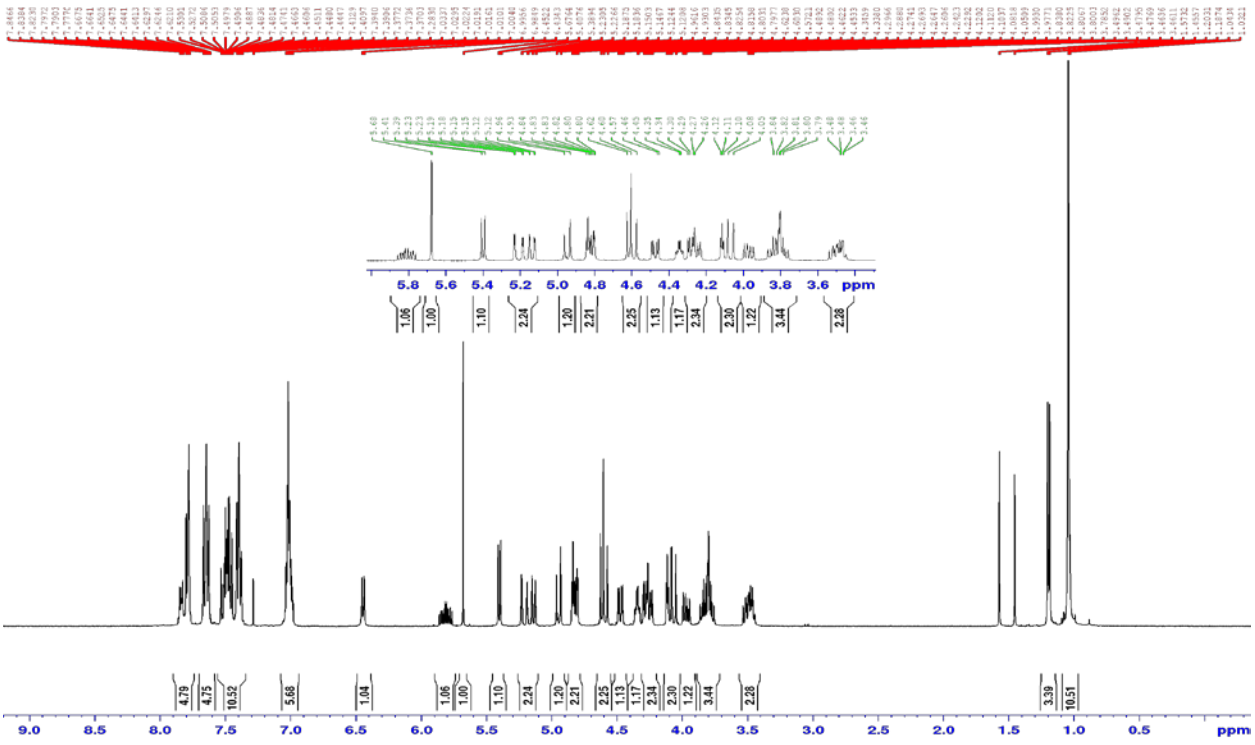

Figure. ^{1}H NMR ($CDCl_3$, 400 MHz) spectrum of **62**.

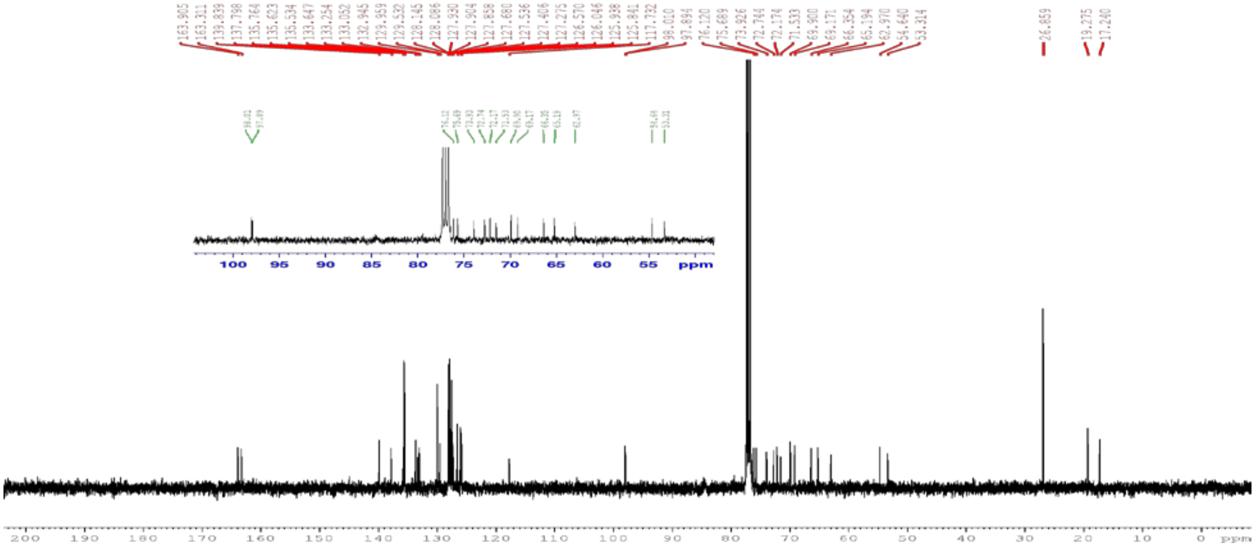

Figure. ^{13}C NMR ($CDCl_3$, 400 MHz) spectrum of **62**.

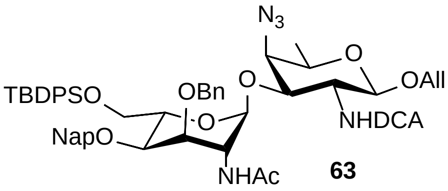

63

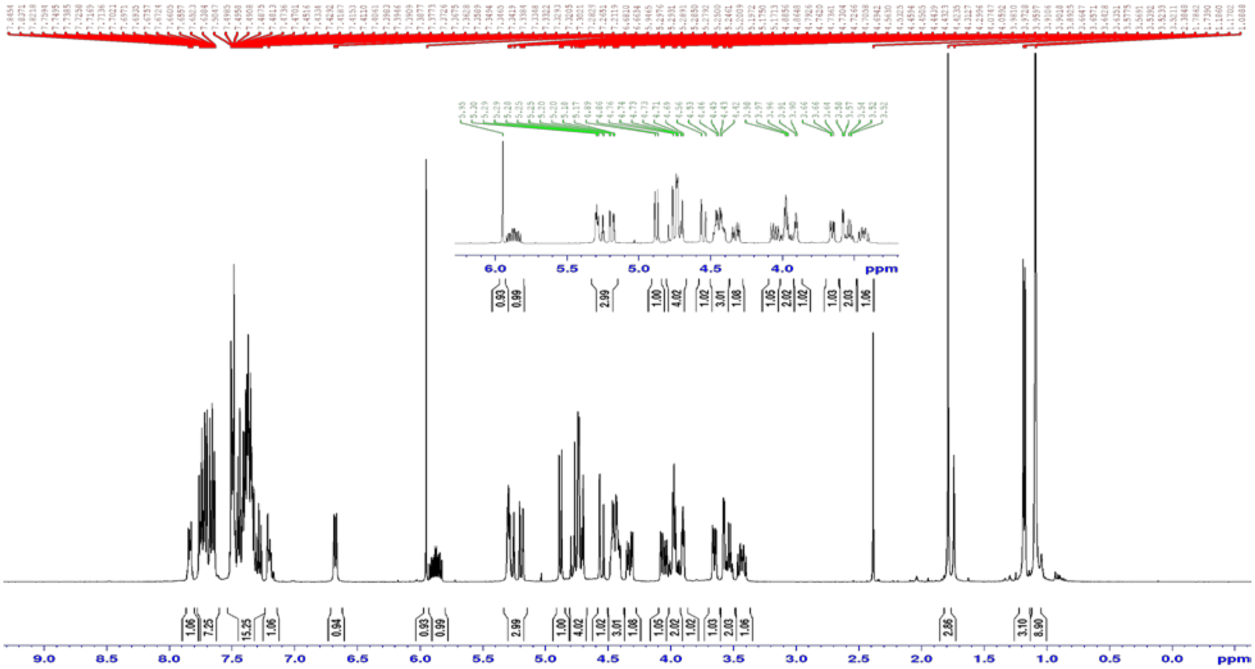

Figure. ^{1}H NMR ($CDCl_3$, 400 MHz) spectrum of **63**.

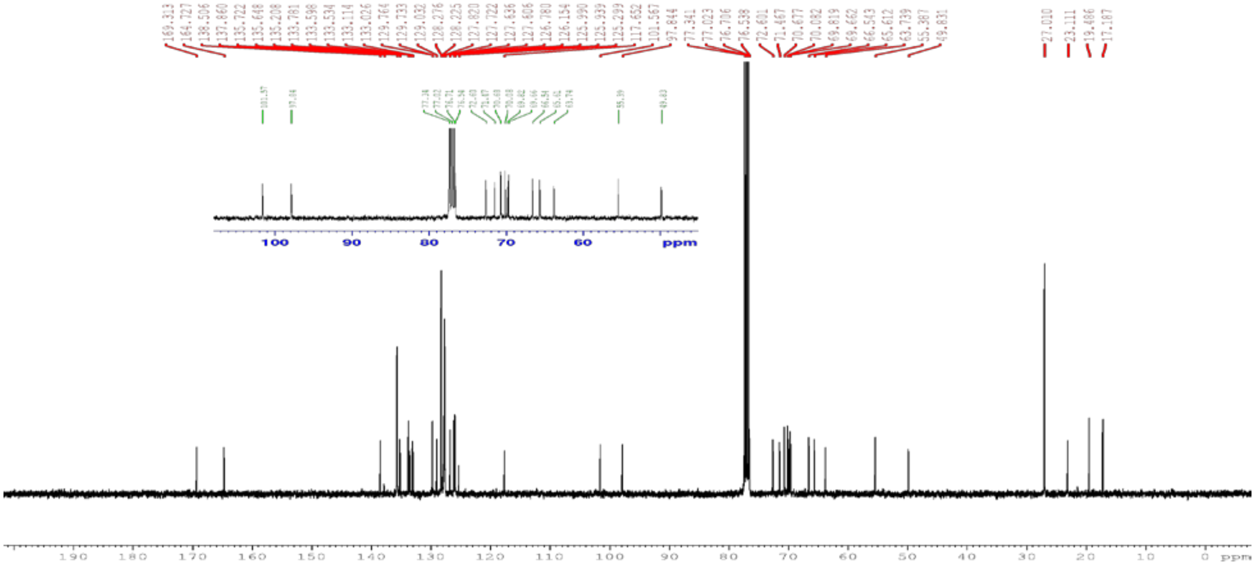

Figure. ^{13}C NMR ($CDCl_3$, 400 MHz) spectrum of **63**.

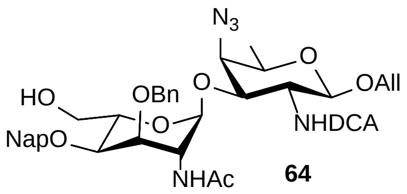

64

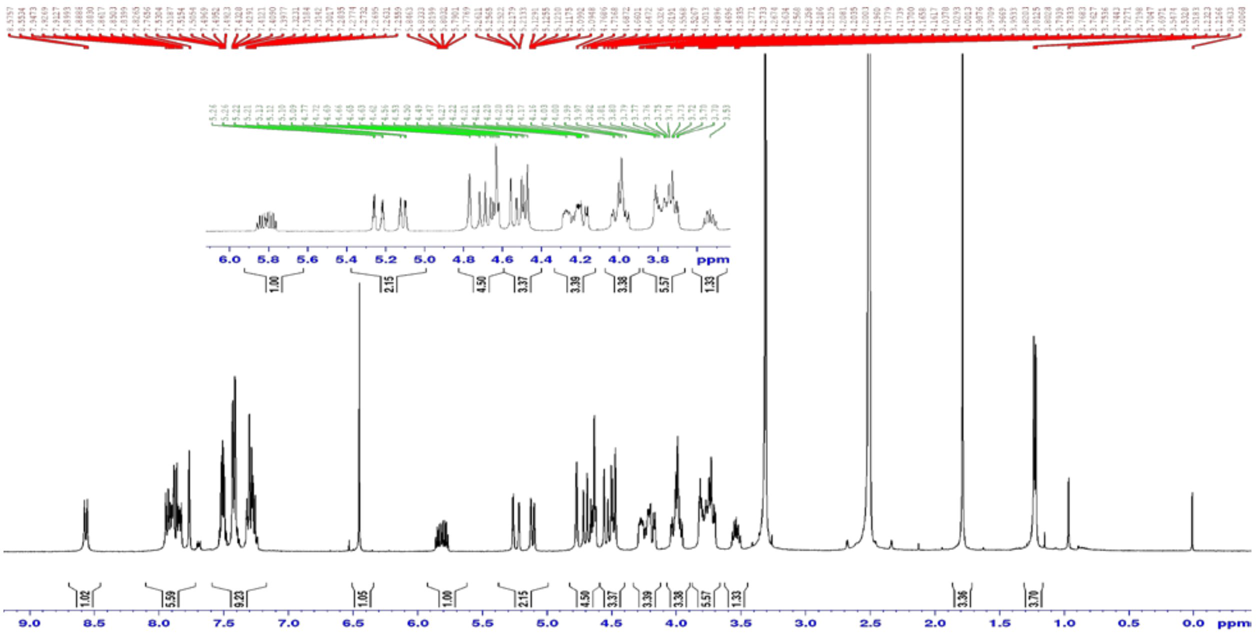

Figure. ^{1}H NMR (DMSO-d_6, 400 MHz) spectrum of **64**.

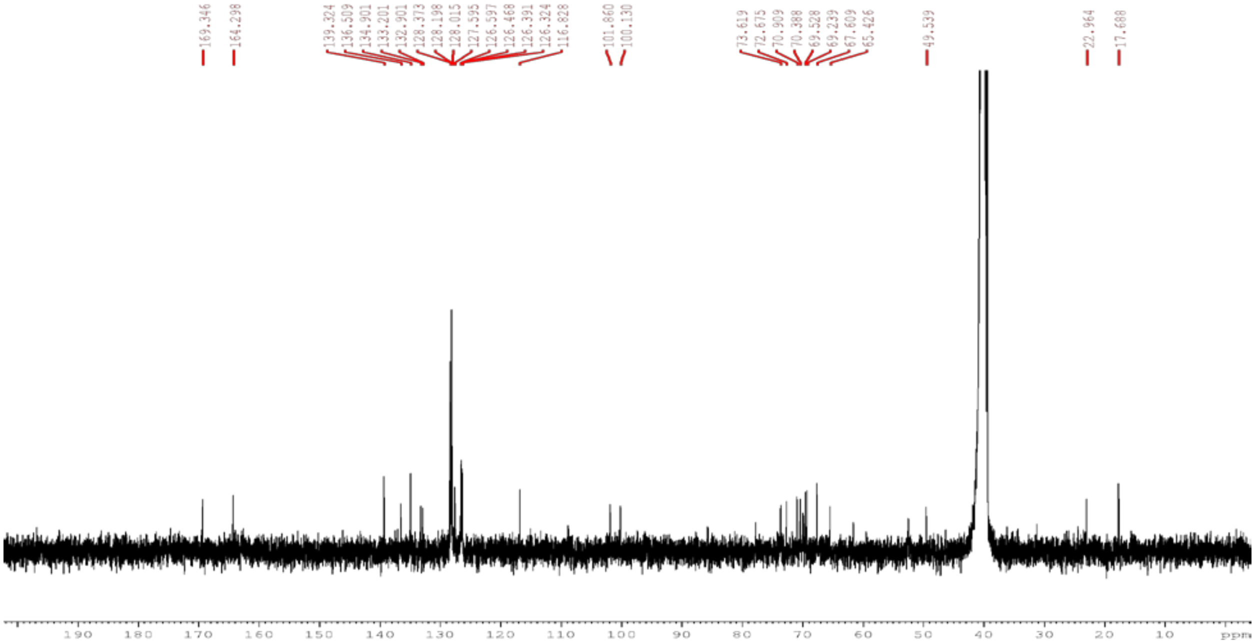

Figure. ^{13}C NMR (DMSO-d_6, 400 MHz) spectrum of **64**.

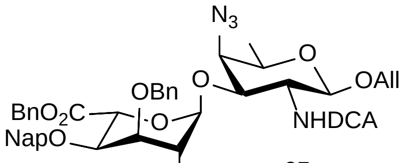

65

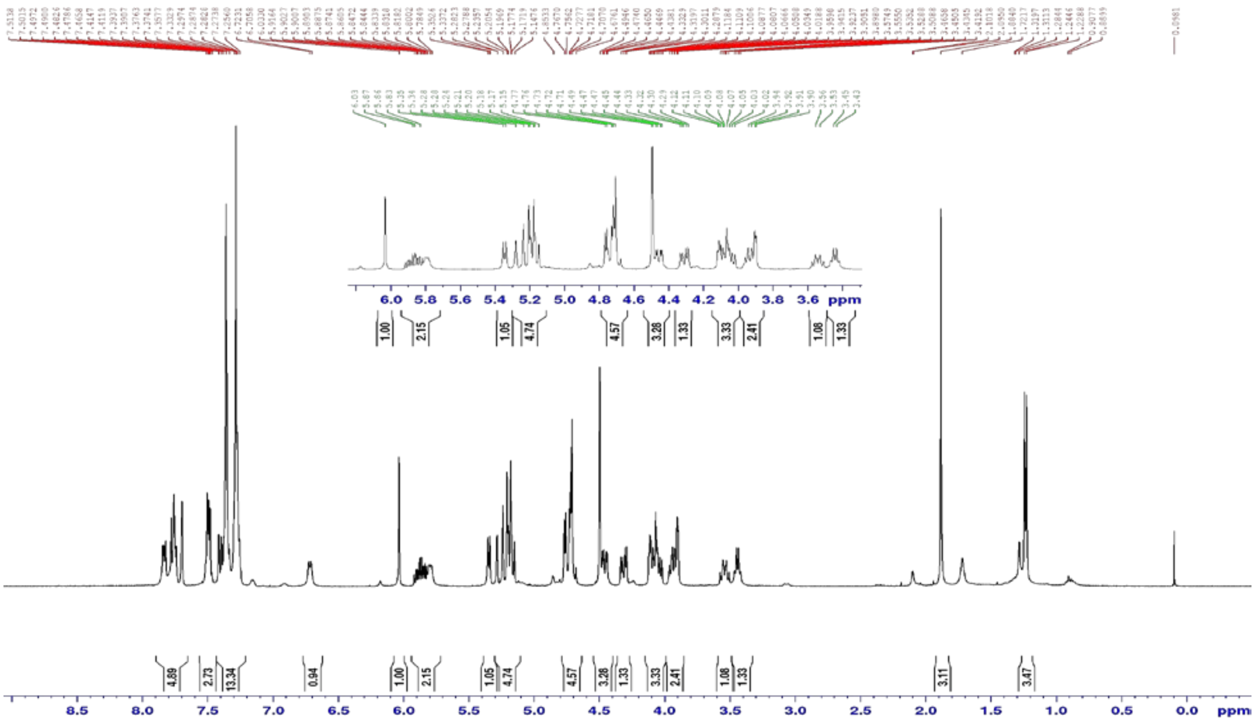

Figure. ^{1}H NMR ($CDCl_3$, 400 MHz) spectrum of **65**.

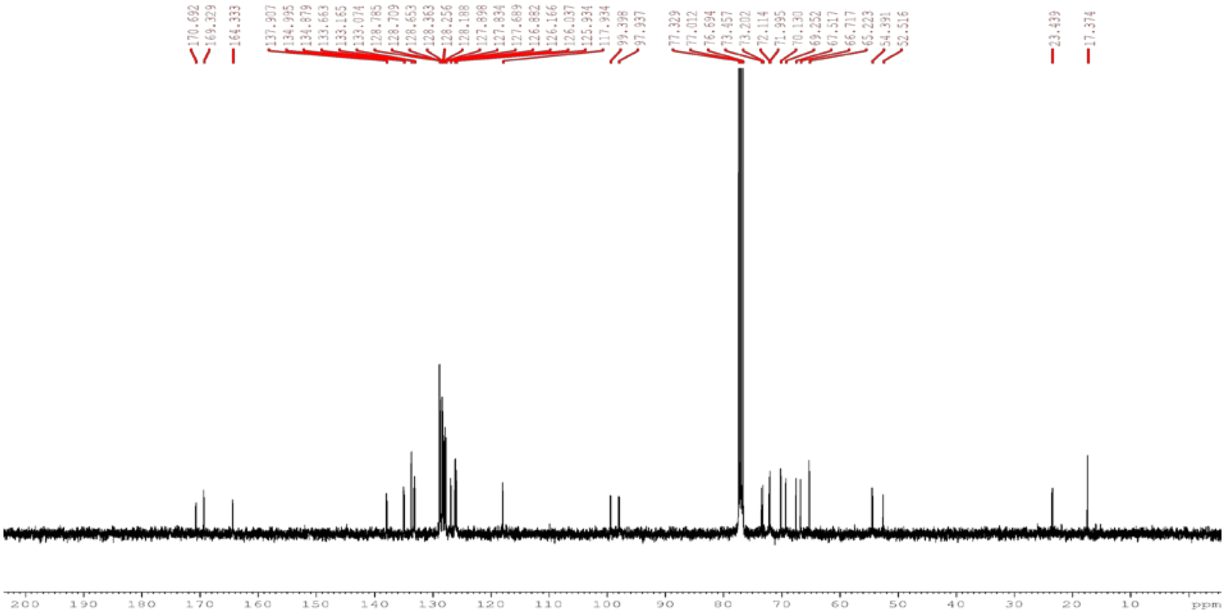

Figure. ^{13}C NMR ($CDCl_3$, 400 MHz) spectrum of **65**.

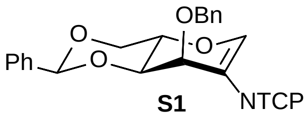

S1

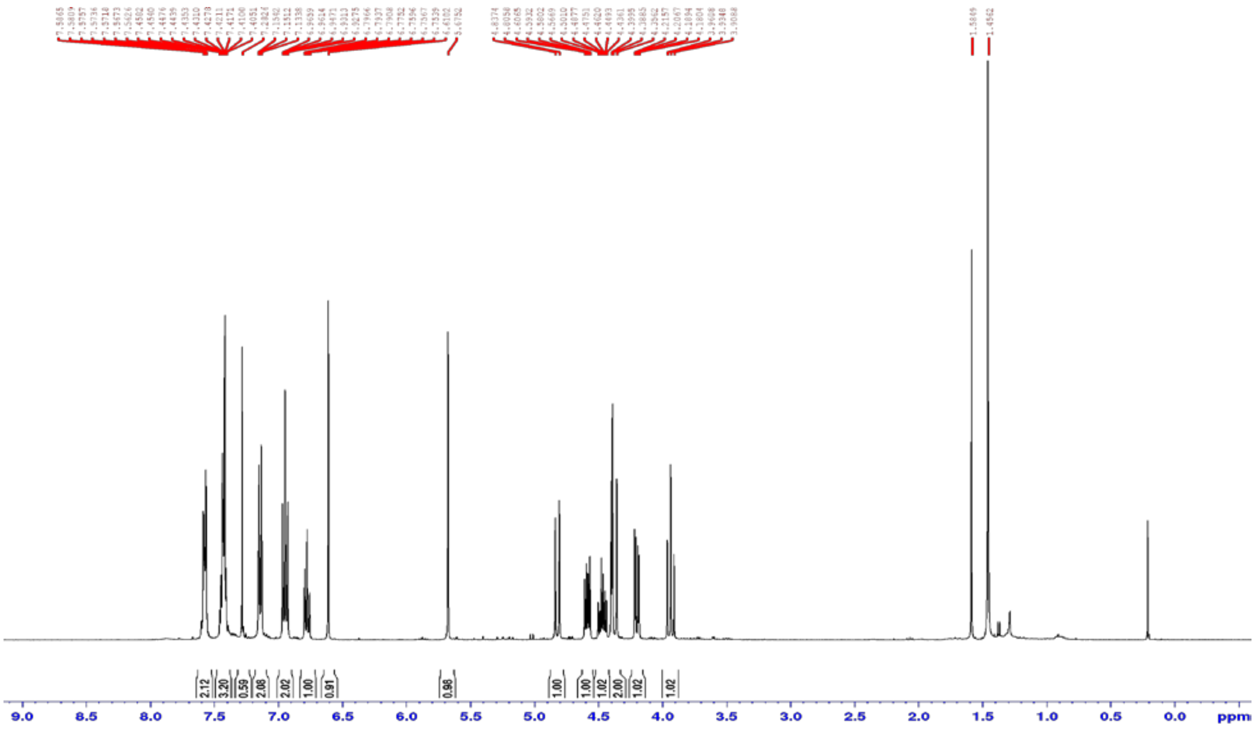

Figure. ^{1}H NMR ($CDCl_3$, 400 MHz) spectrum of **S1**.

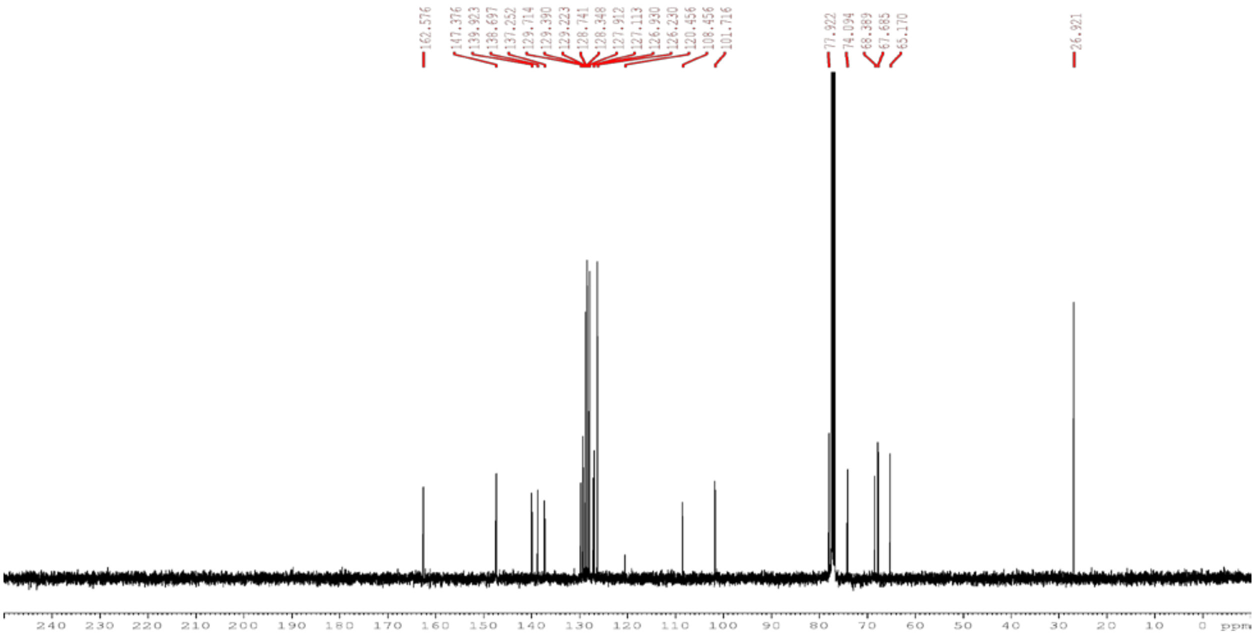

Figure. ^{13}C NMR ($CDCl_3$, 400 MHz) spectrum of **S1**.

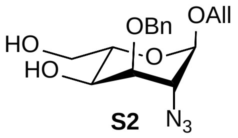

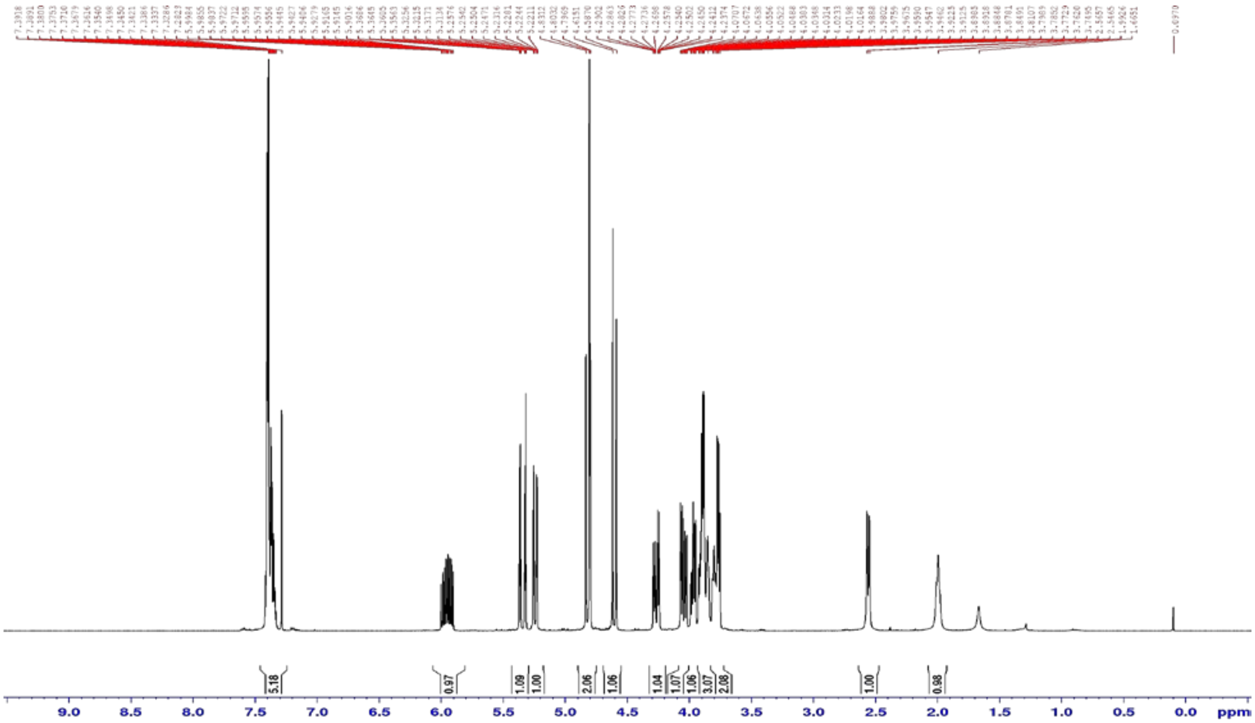

Figure. ^{1}H NMR ($CDCl_3$, 400 MHz) spectrum of **S2**.

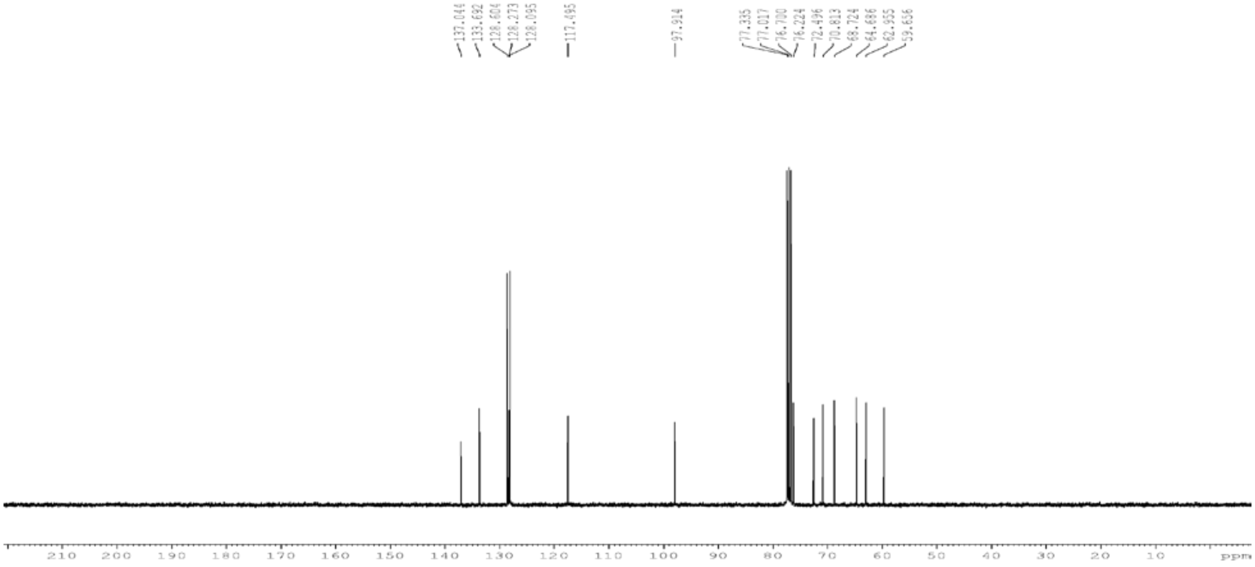

Figure. ^{13}C NMR ($CDCl_3$, 400 MHz) spectrum of **S2**.

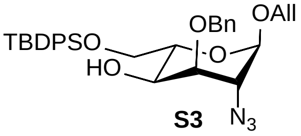

S3

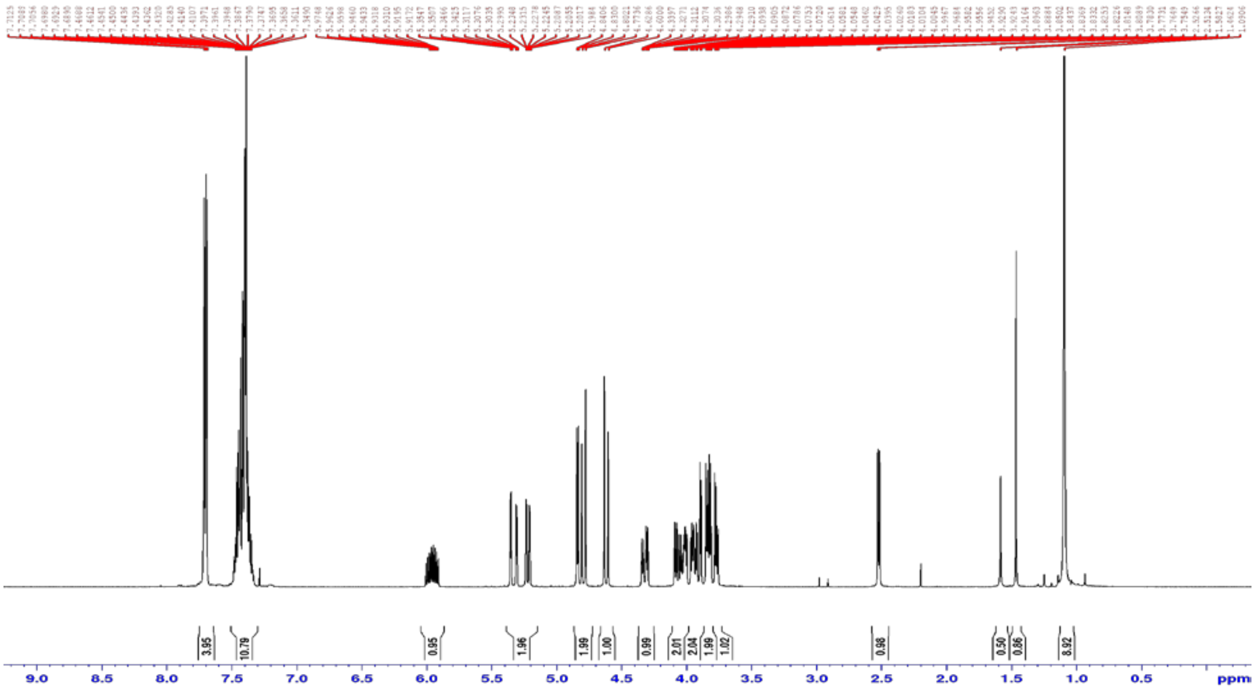

Figure. ^{1}H NMR ($CDCl_3$, 400 MHz) spectrum of **S3**.

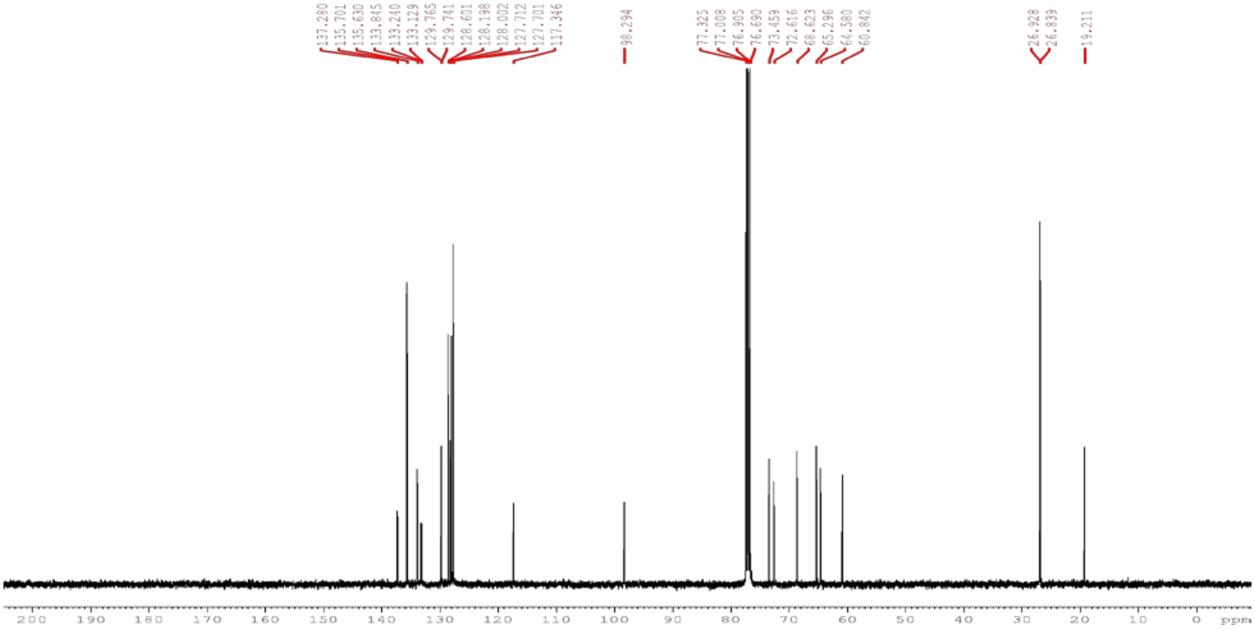

Figure. ^{13}C NMR ($CDCl_3$, 400 MHz) spectrum of **S3**.

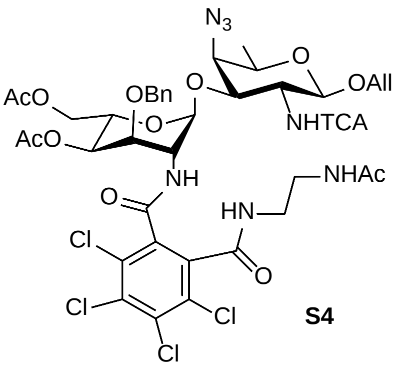

S4

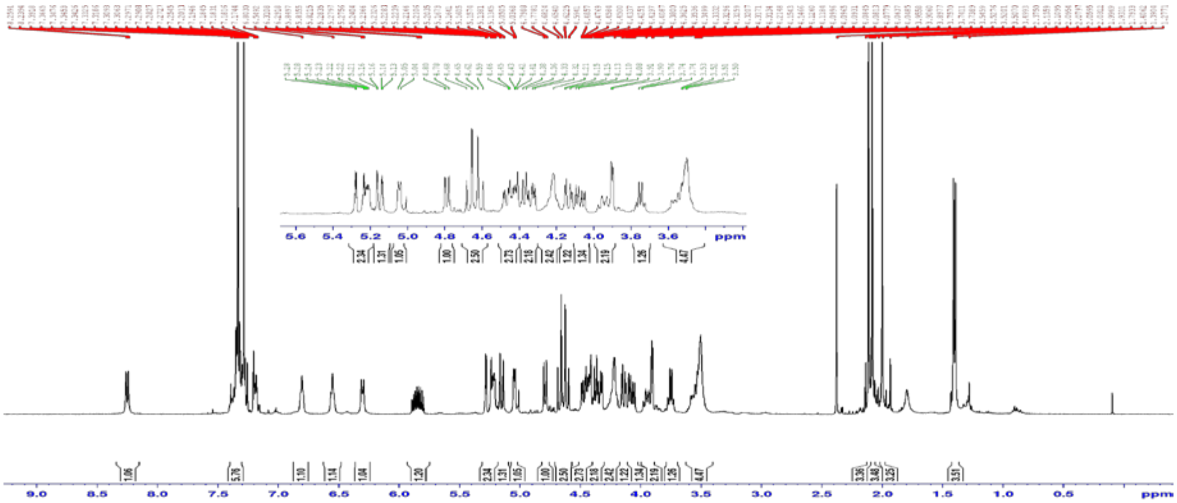

Figure. ^{1}H NMR ($CDCl_3$, 400 MHz) spectrum of **S4**.

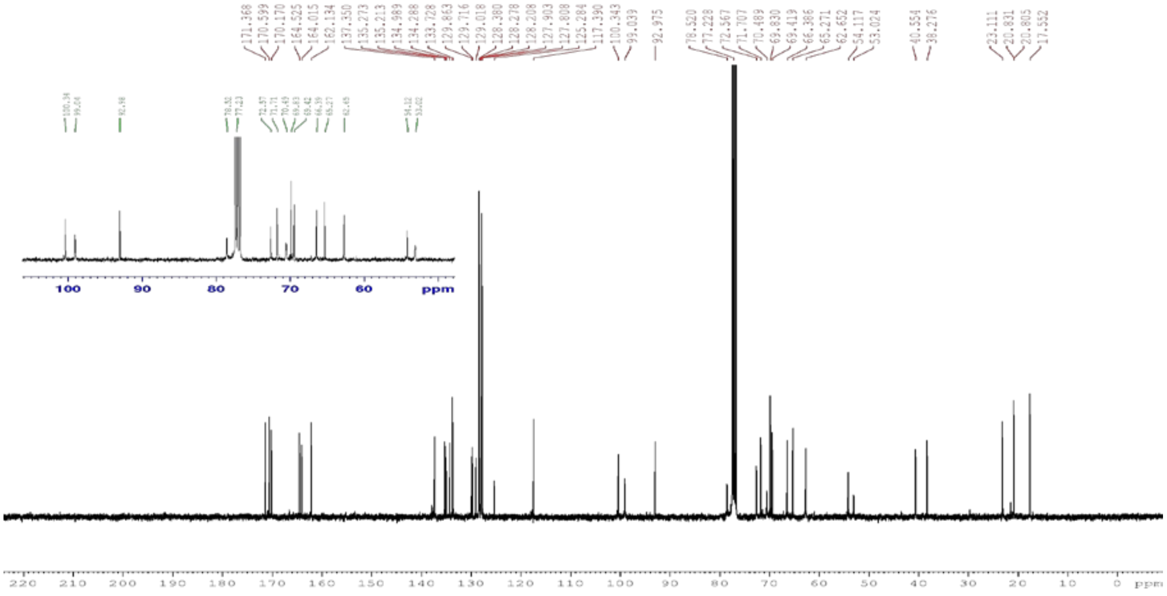

Figure. ^{13}C NMR ($CDCl_3$, 400 MHz) spectrum of **S4**.

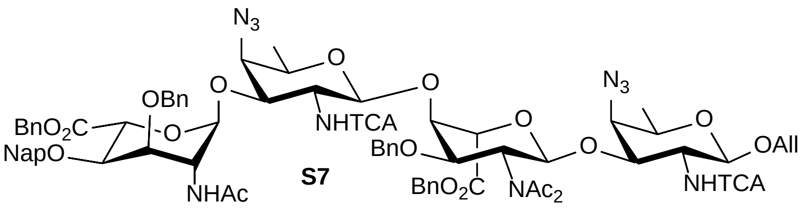

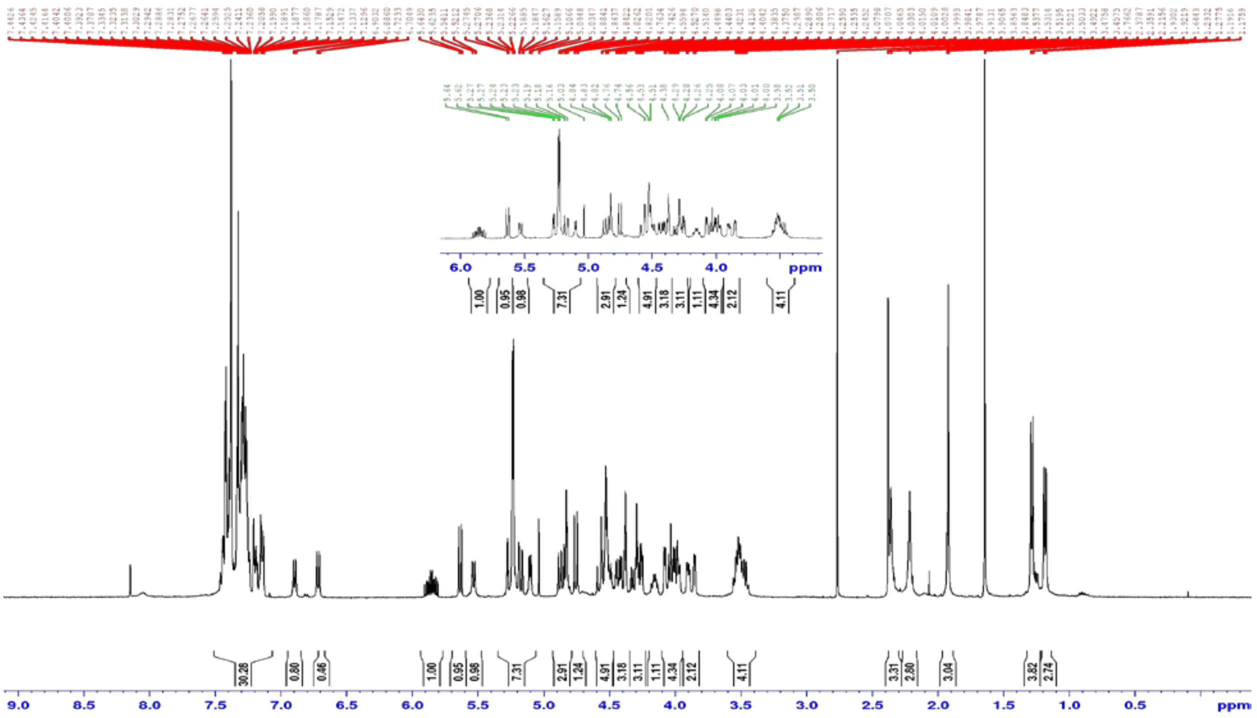

Figure. ^{1}H NMR ($CDCl_3$, 400 MHz) spectrum of **S7**.

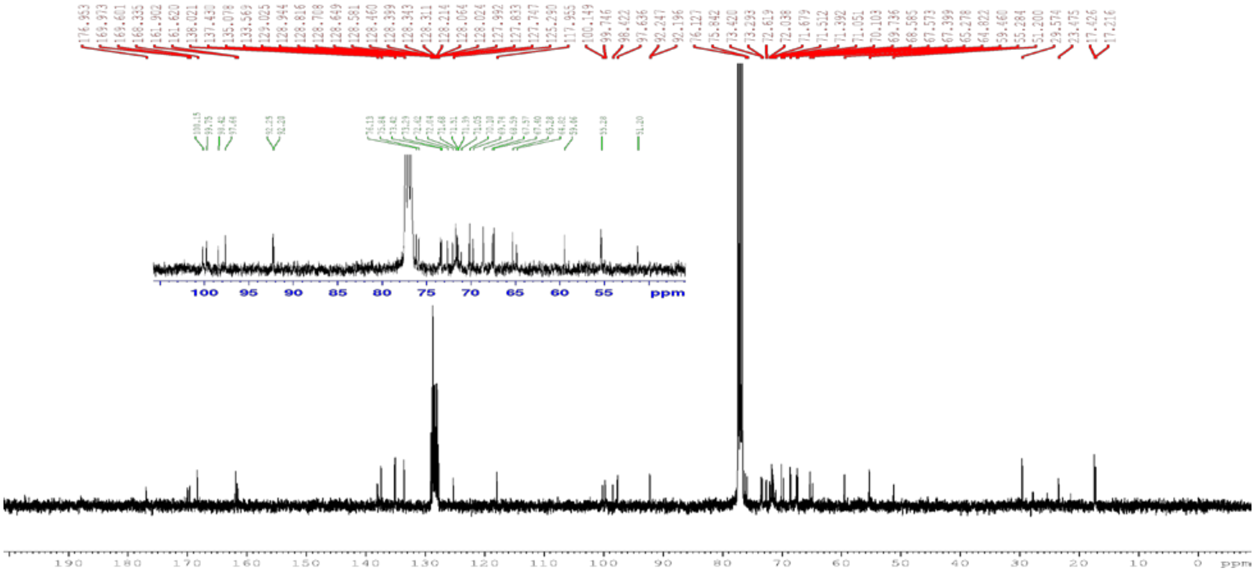

Figure. ^{13}C NMR ($CDCl_3$, 400 MHz) spectrum of **S7**.

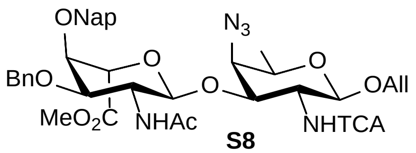

S8

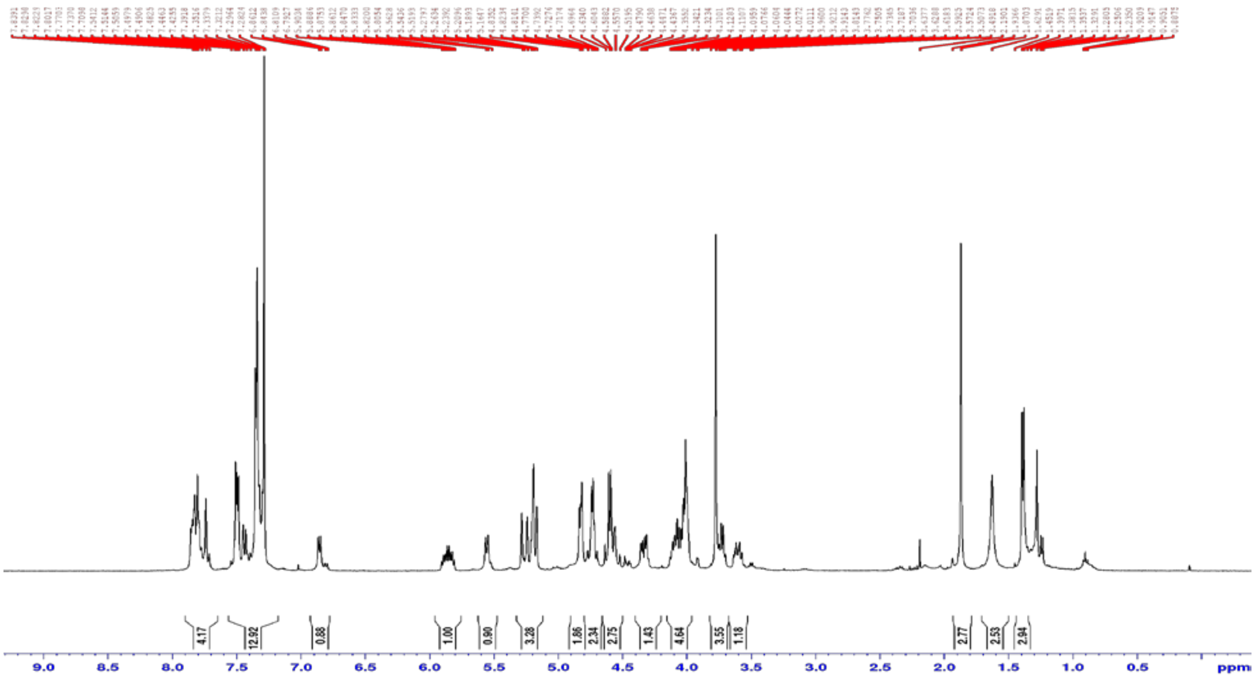

Figure. ^{1}H NMR ($CDCl_3$, 400 MHz) spectrum of **S8**.

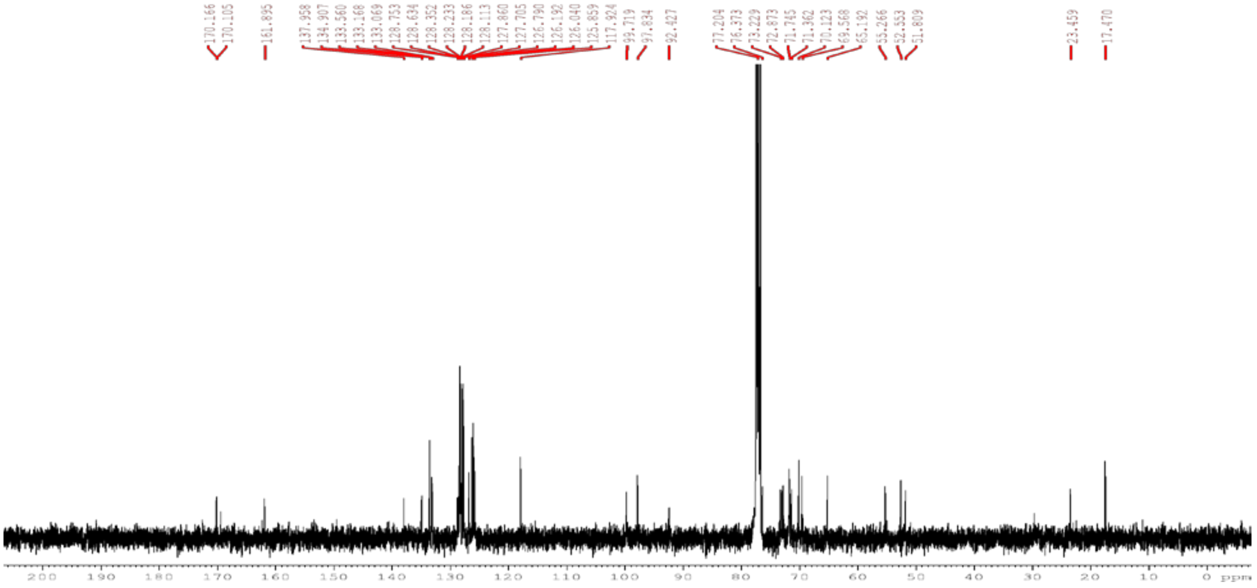

Figure. ^{13}C NMR ($CDCl_3$, 400 MHz) spectrum of **S8**.

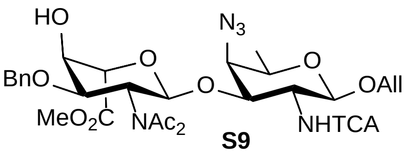

S9

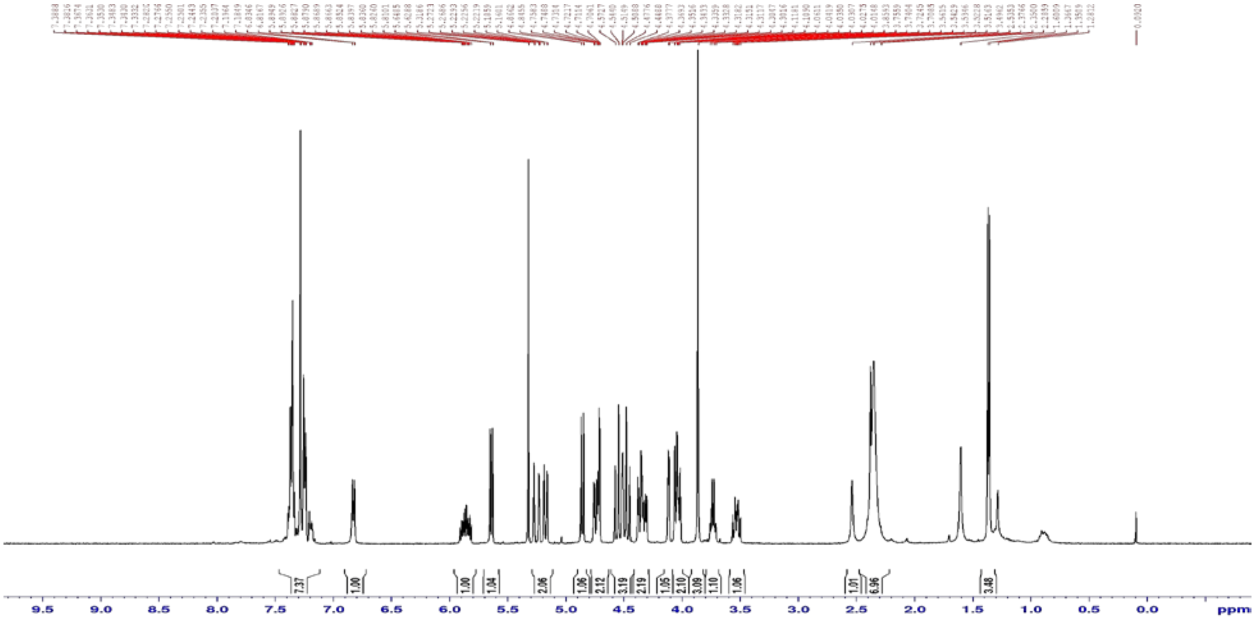

Figure. ^{1}H NMR ($CDCl_3$, 400 MHz) spectrum of **S9**.

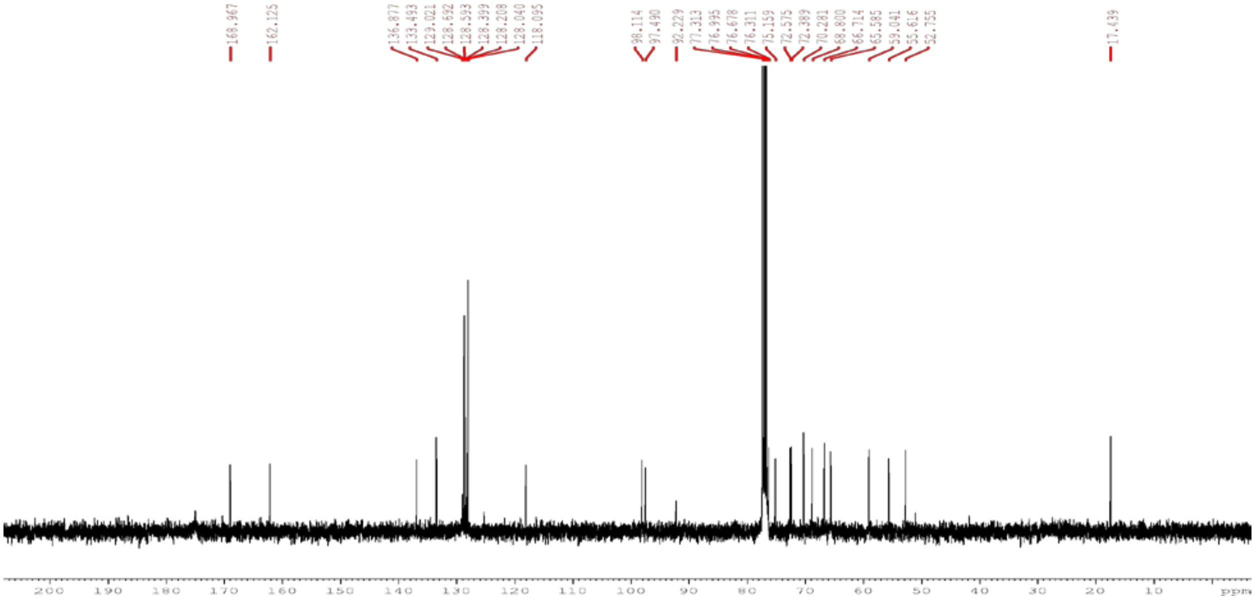

Figure. ^{13}C NMR ($CDCl_3$, 400 MHz) spectrum of **S9**.

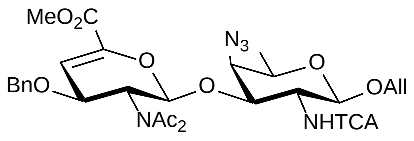

S10

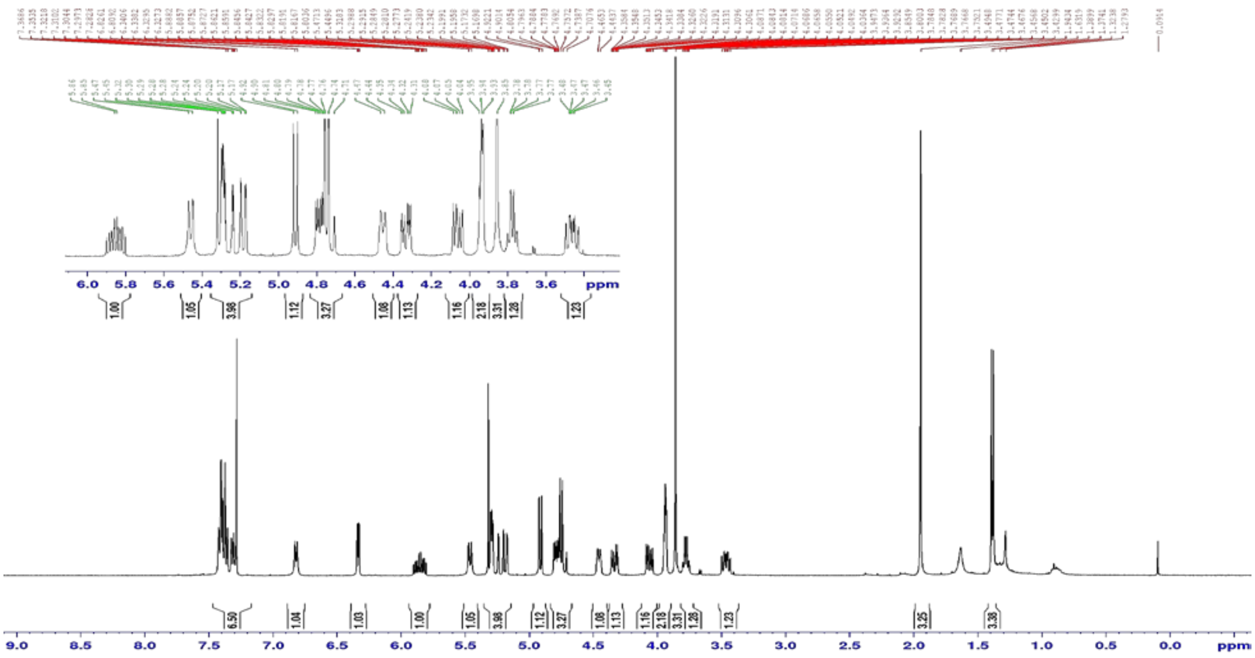

Figure. ^{1}H NMR ($CDCl_3$, 400 MHz) spectrum of **S10**.

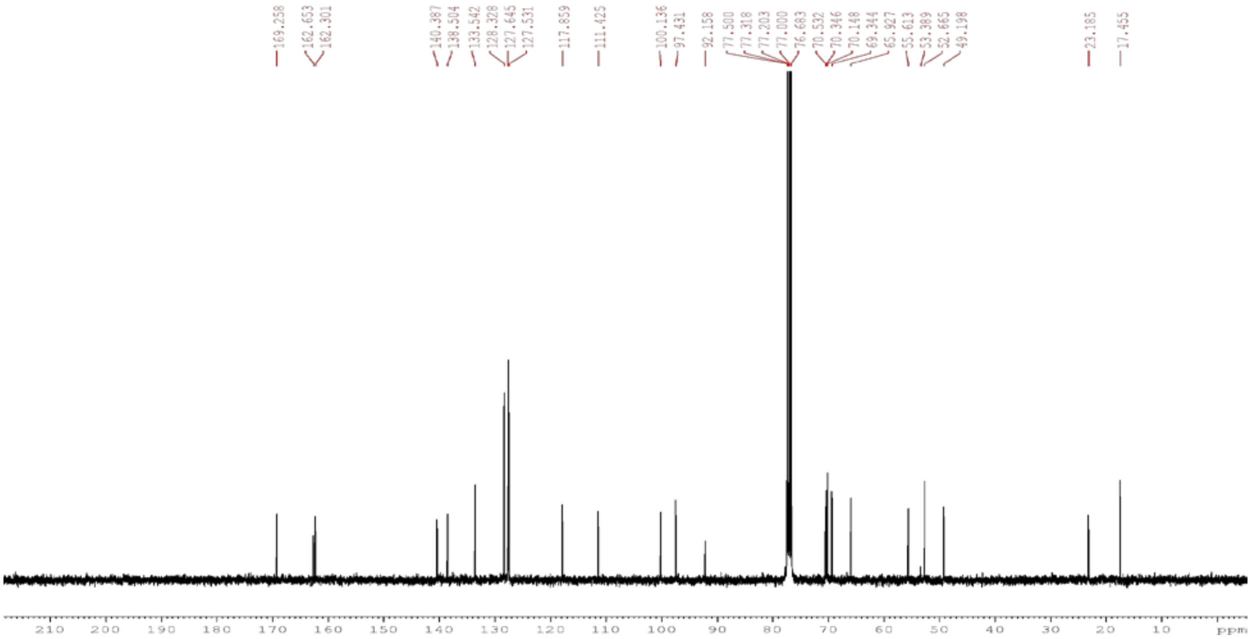

Figure. ^{13}C NMR ($CDCl_3$, 400 MHz) spectrum of **S10**.
